# Distinct hydrogen atom transfer and radical capture reactivity of copper(iii) OH/F complexes enables site-selective C(sp^3^)–H ^18^F-fluorination

**DOI:** 10.1039/d5sc06381g

**Published:** 2025-11-04

**Authors:** Joshua A. Queener, Angela Asor, Margaret A. P. Ball, Jinghua Tang, Jinda Fan, Shiyu Zhang

**Affiliations:** a Department of Chemistry and Biochemistry, The Ohio State University Columbus OH 43210 USA zhang.8941@osu.edu; b Department of Chemistry, Michigan State University East Lansing MI 48824 USA fanjinda@msu.edu; c Department of Radiology, Michigan State University East Lansing MI 48824 USA; d Institute for Quantitative Health Science & Engineering, Michigan State University East Lansing MI 48824 USA

## Abstract

High-valent metal intermediates play a key role in C(sp^3^)–H functionalization reactions in both enzymatic catalysis and organometallic chemistry. Despite its generality, this strategy often requires a single metal complex to efficiently mediate both hydrogen atom transfer and radical capture—a combination challenging to achieve. To overcome this limitation, we propose a decoupled approach, where separate high-valent metal complexes independently perform hydrogen atom transfer (HAT) and radical capture (RC). As a proof of concept, we leveraged the complementary reactivity of copper(iii) hydroxide (efficient for HAT) and copper(iii) fluoride (efficient for RC) to develop a decoupled ^18^F-fluorination protocol. The distinct reactivity of copper(iii) hydroxide and copper(iii) fluoride not only enables precise control over the C–H activation process but also preserves the valuable [^18^F]fluoride for radical capture, preventing its consumption during HAT. With this mechanistic insight, we achieved the selective fluorination of α-ethereal, benzylic, and allylic C–H bonds, facilitating the synthesis of a series of ^18^F-labeled organic molecules.

## Abstract 2

A decoupled HAT and radical capture strategy using formal copper(iii) complexes enables selective C–H ^18^F-fluorination. The distinct selectivity arises from asynchronicity PCET, contrasting the conventional C–H activation selectivity based on BDFE.

## Graphical abstract

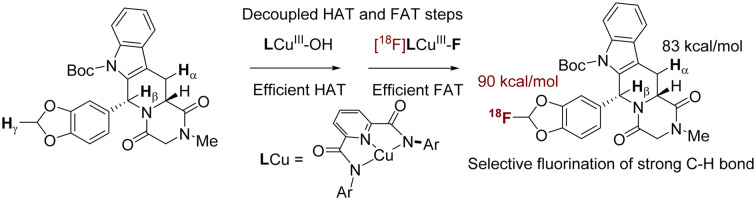

High-valent metal complexes are prototypical intermediates in the activation and functionalization of C–H bonds.^1–4^ Synthetic high-valent metal complexes featuring a diverse range of functional groups (M–FG, FG = functional groups), *e.g.*, hydroxide, superoxo, carboxylate, halides, nitrite, and nitrate, have been shown to activate alkyl C–H bonds.^5–14^ In some cases, the M–FG complexes not only activate the C–H bond *via* hydrogen atom transfer (HAT) but also perform sequential radical capture (RC) to install the FG group on the C(sp^3^)–H position (Scheme 1A). For instance, studies by Tolman,^15–20^ McDonald,^21–25^ and our group,^26–28^ have demonstrated that formal copper(iii) and nickel(iii) complexes can perform C–H halogenation, cyanation, and nitration. These transformations typically proceed with two equivalents of M–FG complexes – the first equivalent performs HAT, the second equivalent captures the R˙ generated from the first step (Scheme 1A).

**Scheme 1:**
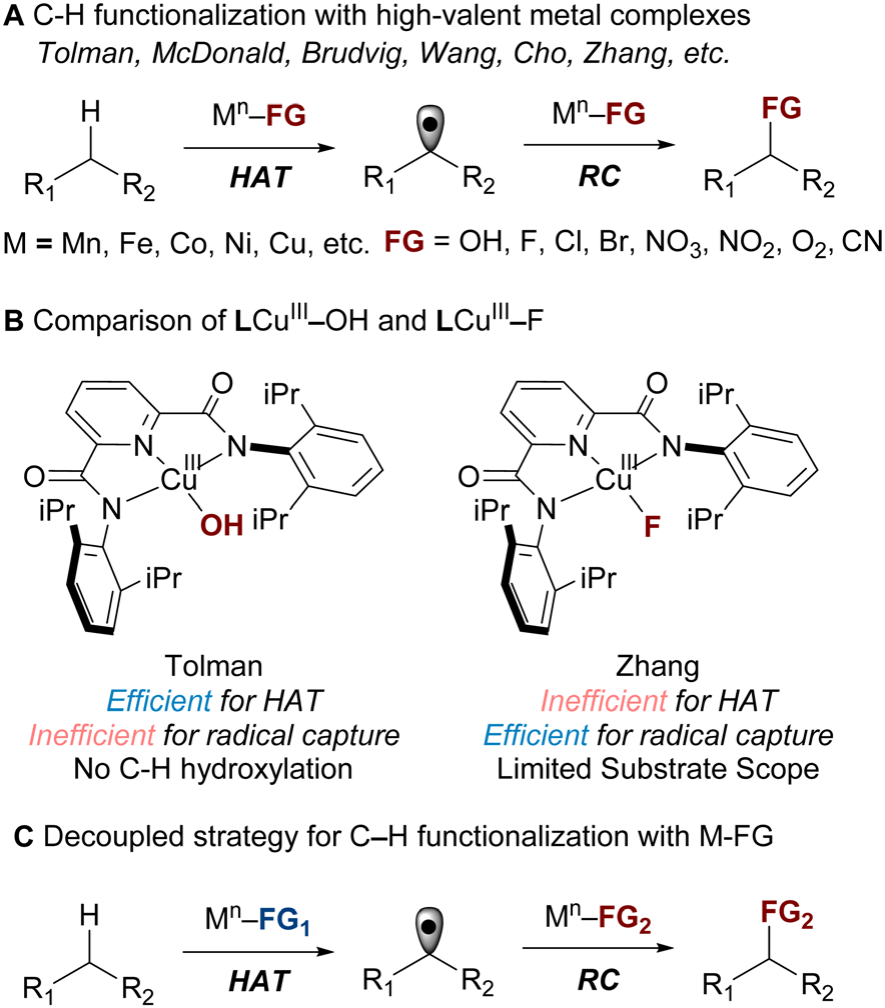


Despite its generality, this strategy often requires a single metal complex to efficiently mediate both HAT and RC, a combination challenging to achieve. In many cases, the HAT and RC steps are not well synchronized, resulting in poor overall C–H functionalization reactivity. For instance, the LCu^III^–F (L = bis(2,6-diisopropylphenyl)pyridinedicarbox-amide) reported by us is efficient at RC, but the HAT process is sluggish, limiting the scope of C–H fluorination.^26,29^ Conversely, the LCu^III^–OH complex reported by Tolman excels in HAT but is inefficient at RC, explaining why it has not been shown to perform C–H hydroxylation (Scheme 1B).

Another limitation is that functionalizing one equivalent of the C–H substrate requires two equivalents of the LCu^III^–FG complex, one for HAT and another for RC (Scheme 1A).^26,29^ Consequently, applying this method to ^18^F-fluorination (fluorine-18 is a short-lived radionuclide with *t*_1/2_ = 109.7 min) would result in the loss of one equivalent of valuable fluorine-18 for HAT.

To address these issues, we consider the possibility of a decoupled HAT and RC process, where HAT is performed with one high-valent metal complex (M–FG_1_) and RC is performed with another (M–FG_2_, Scheme 1C). To ensure that M–FG_1_ and M–FG_2_ perform their corresponding tasks without interfering with each other, it is essential to understand how the identity of the functional groups (FG_1_*vs.* FG_2_) influences their relative rates and selectivity of HAT and RC.

As a proof-of-concept for this decoupled C–H functionalization strategy, we target the C–H ^18^F-fluorination of the C(sp^3^)–H bond. Organic molecules containing fluorine-18 are valuable for positron emission tomography (PET), a highly sensitive bioimaging technique used to diagnose cancers, neurological disorders, and cardiovascular diseases.^30–34^ Traditional ^18^F-labeling methods rely on the substitution of pre-existing functional groups with fluorine-18, *e.g.*, I,^35–41^ Cl,^42^ Br,^43,44^ S,^45,46^ NR_2_,^47,48^ OR,^49–56^ CO_2_R,^57^ BR_2_,^58–63^ and SnR_3_.^64,65^ However, recent efforts have shifted toward direct incorporation of fluorine-18 to C–H bonds.^66–68^ Previous studies by Hooker, Groves,^6,8^ Sanford, Scott,^35,69,70^ Nicewicz, and Li^71,72^ demonstrated the use of the readily available [^18^F]fluoride anion for ^18^F-fluorination of both C(sp^3^)–H and C(sp^2^)–H bonds (Scheme 2).

**Scheme 2:**
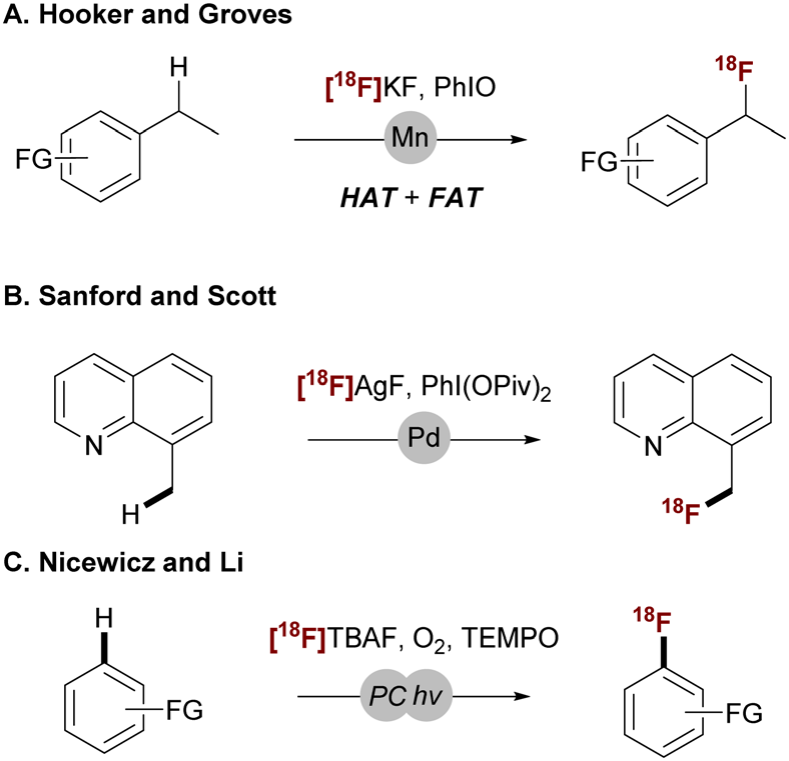
C–H ^18^F-fluorination using [^18^F]F^−^ sources.

Despite these advancements, the selectivity of C(sp^3^)–H ^18^F-fluorination is often governed by the bond dissociation free energy (BDFE) of the C(sp^3^)–H bonds^6,8^ or the presence of a directing group.^35,69,70^ Developing an alternative approach that decouples the HAT and RC steps would allow for independent tuning of each process. This strategy could enable precise control over C–H activation through the steric and electronic properties of the metal complex, without compromising the efficiency of fluorine-18 atom transfer, ultimately providing alternative chemoselectivity in C–H ^18^F-fluorination.

Herein, we investigate the distinct reactivity of LCu^III^–OH and LCu^III^–F. We found that LCu^III^–OH exhibits significantly higher efficiency in HAT (>400 times faster than LCu^III^–F), while LCu^III^–F demonstrates better performance in RC. Leveraging the contrasting reactivity of LCu^III^–OH and LCu^III^–F, we developed a ^18^F-fluorination strategy, utilizing a simple mixture of LCu^III^–OH and [^18^F]LCu^III^–F complexes. This decoupled strategy allows tuning of C–H activation selectivity *via* steric control, polarity matching, and HAT asynchronicity,^27,29^ without interfering with the efficiency of fluorine-18 atom transfer. We demonstrate the utility of this approach by labelling a broad range of C(sp^3^)–H substrates, including pharmaceutical compounds, with fluorine-18, achieving C–H fluorination selectivity distinct from previously reported systems.

Our investigation began with the evaluation of the HAT rates of LCu^III^–OH and LCu^III^–F complexes. The copper(ii) precursors ([LCu^II^–OH]^−^ and [LCu^II^–F]^−^) have been isolated previously, and the corresponding copper(iii) species can be generated *in situ* using chemical oxidants, NAr_3_[PF_6_] (tris(*p*-bromophenylammoniumyl)hexafluorophosphate).^15,73^ Addition of dihydroanthracene (DHA), a model hydrogen atom donor, to LCu^III^–OH or LCu^III^–F complexes at −30 °C led to the consumption of the copper complex, as revealed by UV-vis spectroscopy (Fig. 1A and B). The reaction between LCu^III^–F and DHA was confirmed to generate anthracene as the product, as evidenced by GC-MS analysis (Fig. S12–S14), indicating that the consumption of LCu^III^–F is due to HAT. Notably, Tolman *et al.* previously reported that the reaction of LCu^III^–OH with DHA also affords anthracene.^15^ Therefore, it is reasonable to infer that both LCu^III^–OH and LCu^III^–F undergo the same HAT reaction mechanism with DHA under these conditions.

**Fig. 1:**
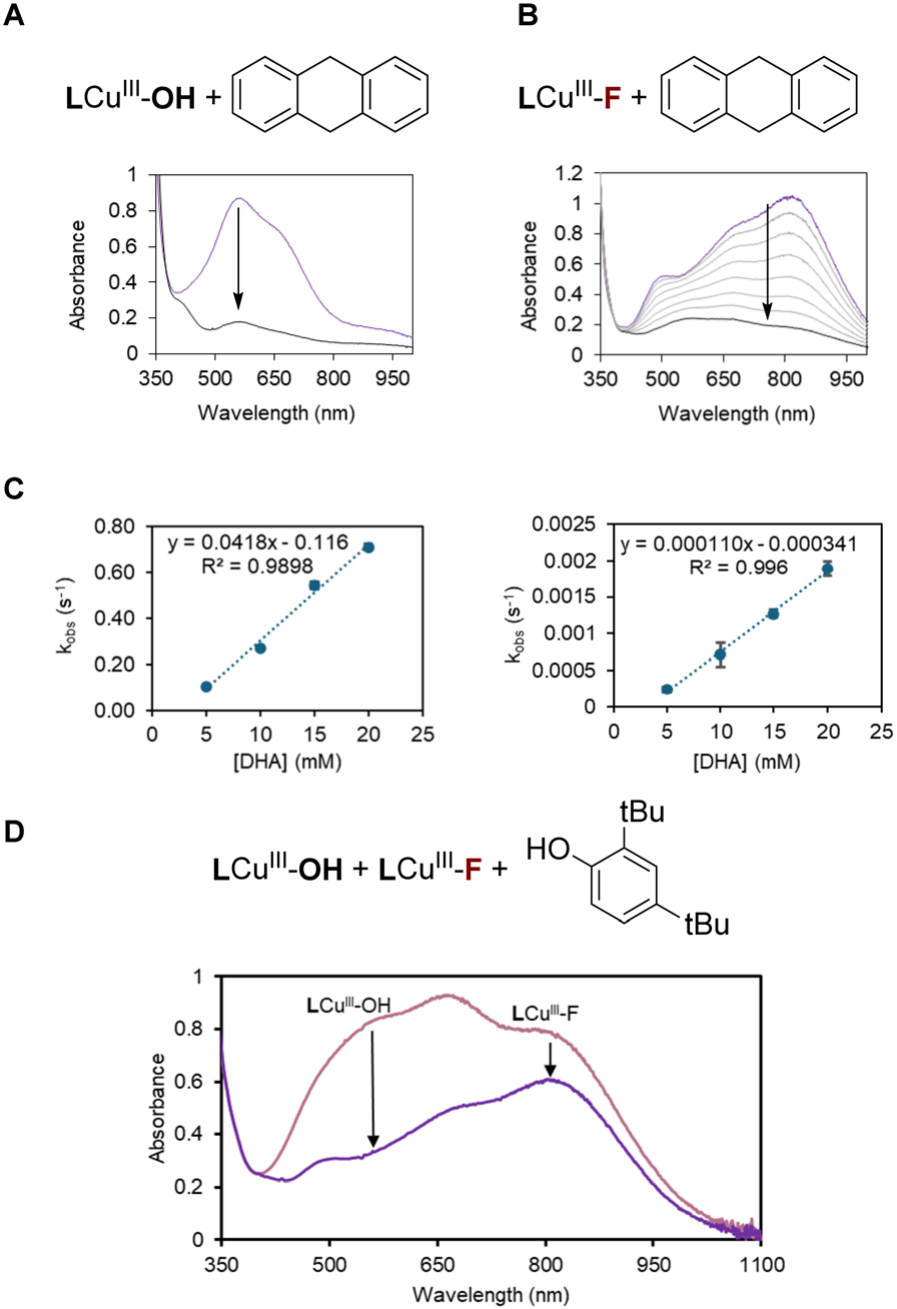
HAT kinetics of (A) LCu^III^–OH and (B) LCu^III^–F with 100 equivalents of DHA monitored by UV-vis spectrometry in dichloroethane at −30 °C. (C) The plots of *k*_obs_ (s^−1^) for each complex with varying equivalents of DHA shows the faster HAT rate of LCu^III^–OH than LCu^III^–F. (D) UV-vis spectra of the reaction between the 1 : 1 mixture of LCu^III^–OH and LCu^III^–F with 1 equivalent of 2,4-di-*tert*-butylphenol monitored by UV-vis spectrometry in DCE at −30 °C after 1 minute.

The second-order rate constants (*k*_HAT_) were calculated by monitoring the decay of ligand-to-metal charge transfer (LMCT) bands of LCu^III^–OH (560 nm) and LCu^III^–F (820 nm, Fig. 1C) at varying equivalents of DHA. The LCu^III^–OH complex shows a HAT rate about 400 times faster than that of LCu^III^–F (0.04(1) *vs.* 0.0001(1) M^−1^ s^−1^, Fig. 1C), suggesting that LCu^III^–OH would perform HAT preferentially in the presence of LCu^III^–F.

To confirm this hypothesis, we investigated the competitive HAT reactivity between LCu^III^–OH and LCu^III^–F in solution. Surprisingly, the addition of DHA to a 1 : 1 mixture of LCu^III^–OH and LCu^III^–F resulted in the consumption of both complexes at approximately equal rates (Fig. S15). This observation contrasts with our findings above that LCu^III^–OH exhibits a HAT rate 400 times faster than LCu^III^–F when the HAT reaction is evaluated in separate solutions. We attribute this apparent discrepancy to the fact that DHA donates two hydrogen atoms sequentially, with the second being more easily abstracted and transferred non-selectively, leading to concurrent consumption of both copper(iii) species.

To avoid complications from secondary H-atom transfer, we used 2,4-di-*tert*-butylphenol as the HAT donor in the competitive HAT studies. HAT from 2,4-di-*tert*-butylphenol yields phenoxy radicals that dimerize cleanly to form the C–C phenol dimer, allowing accurate evaluation of competitive HAT reactivity. Indeed, GC-MS analysis confirmed that both LCu^III^–OH and LCu^III^–F oxidize 2,4-di-*tert*-butylphenol to produce the phenol dimer (3,3′,5,5′-tetra(*tert*-butyl)biphenyl-2,2′-diol), as the main product (see the SI).

When a 1 : 1 mixture of LCu^III^–OH and LCu^III^–F is treated with one equivalent of hydrogen atom donor, 2,4-di-*tert*-butylphenol (chosen as a simple single H atom donor), only 5% of LCu^III^–F is consumed before the complete consumption of all LCu^III^–OH, validating the selective HAT activity by LCu^III^–OH under these conditions (Fig. 1D). Additionally, UV-vis analysis of a 1 : 1 mixture of LCu^III^–OH and LCu^III^–F indicates that the two species coexist without detectable side reactions, such as ligand exchange or disproportionation.

Next, we evaluated if LCu^III^–F can capture C-centered radicals to form the desired C–F bonds without interference from LCu^III^–OH. We compared the RC capability of LCu^III^–OH and LCu^III^–F by treating them with 0.5 equivalent of Gomberg's dimer (Scheme 3), which can dissociate into the trityl radical (Ph_3_C˙) in solution, serving as a model for C-centered radicals. The yield of the radical capture product (Ph_3_C–F) was found to be 85% by ^19^F NMR, while only a trace amount (<1%) of the hydroxide transferred product (Ph_3_C–OH) was observed by GC-MS (see the SI). When a 1 : 1 mixture of LCu^III^–OH and LCu^III^–F was treated with 0.5 equivalent of Gomberg's dimer (Scheme 3), the yield of the fluorine atom transfer (FAT) product (Ph_3_C–F) was found to be 32% by ^19^F NMR, and again only a trace amount (<1%) of the hydroxide transferred product (Ph_3_C–OH) was observed by GC-MS (see the SI). The reason for the reduced yield of Ph_3_C–F in the presence of LCu^III^–OH is unclear to us. However, the much higher yield of Ph_3_C–F than Ph_3_C–OH suggests that LCu^III^–F might preferentially capture the alkyl radical generated from HAT. The higher radical capture efficiency observed for LCu^III^–F compared to LCu^III^–OH can be attributed to the higher redox potential of LCu^III^–F (216.4 mV *vs.* Fc^+^/Fc). Because alkyl radical capture is proposed to proceed *via* an electron transfer–halide transfer mechanism, the higher redox potential of LCu^III^–F facilitates oxidation of the alkyl radical and thus promotes efficient radical capture, whereas the lower redox potential of LCu^III^–OH (−135.2 mV *vs.* Fc^+^/Fc) renders it much less effective.^74^

**Scheme 3:**
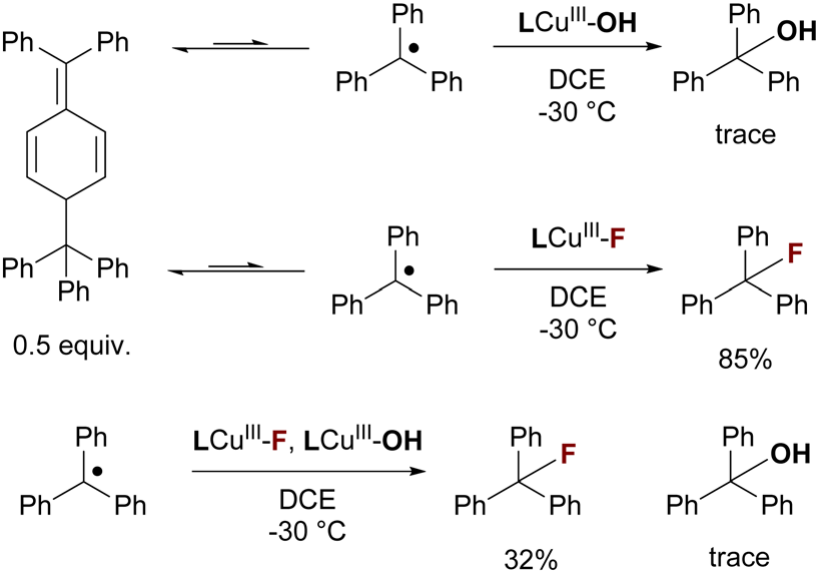
Selectivity of radical capture with LCu^III^–FG.

After establishing that LCu^III^–OH and LCu^III^–F can perform HAT and RC, respectively, we set out to develop a method that combines the HAT and RC steps to achieve C(sp^3^)–H ^18^F-fluorination (Scheme 4). First, we passed an acetonitrile solution of TBAOH·30H_2_O through an ion-exchange cartridge loaded with [^18^F]F^−^ (*ca.* 0.11 GBq, 3 mCi) to generate a mixture of TBAOH and [^18^F]TBAF.

**Scheme 4:**
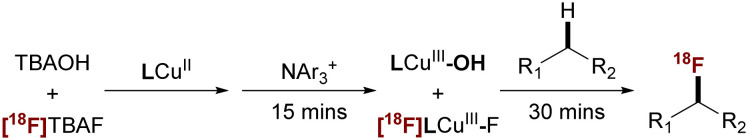
Procedure for ^18^F labeling.

Treatment of the solution mixture of TBAOH and [^18^F]TBAF with one equivalent of LCu^II^–MeCN (with respect to the total TBA concentration) led to a color change to dark blue, consistent with the formation of a mixture of [LCu^II^–OH]^−^ and [^18^F][LCu^II^–F]^−^. A full equivalent of LCu^II^–MeCN, with respect to the total concentration of TBAOH and TBAF, was added to ensure that all nucleophilic ligands (OH^−^ and [^18^F]F^−^) are properly ligated, thereby preventing competitive displacement of [^18^F]F^−^ with OH^−^. This mixture was oxidized to the copper(iii) state using NAr_3_[PF_6_] (*E*_1/2_ = 0.710 V *vs.* Fc/Fc^+^ in dichloroethane DCE) and then transferred to a DCE solution containing C–H substrates at −20 °C for C–H ^18^F-fluorination.

Excitingly, we found that the model C–H substrate 1,3-benzodioxole (1a) was converted to the ^18^F-labeled product in 92% radiochemical conversion (RCC, average of four trials, *n* = 4) under the optimized conditions (Table 1). The entire procedure can be completed within an hour (Scheme 4). The radiochemical conversions were determined by radio-TLC analysis of the reaction mixtures. The identity of the ^18^F-labeled product was confirmed by comparing the retention time of the ^18^F-labeled products with the corresponding ^19^F standards using radio-HPLC (see the SI). The present Cu method also proceeds under ^19^F conditions; however, due to difficulties in separating the fluorinated product from the unreacted starting material, an alternative method using a stoichiometric amount of C–H substrates was developed for isolating the ^19^F standards (see the SI).

**Table 1:** Optimization of ^18^F C–H fluorination conditions

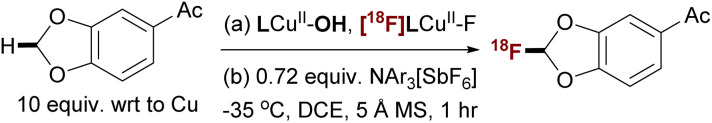		
Entry	Deviation from standard conditions	RCC
1	None	92%
2	No 5 Å MS	18%
3	1.0 equiv. of substrate	23%
4	0.72 equiv. NAr_3_[PF_6_]	86%
5	1.0 equiv. NAr_3_[PF_6_]	25%

The reaction is sensitive to the water introduced by TBAOH·30H_2_O. Without 5 Å molecular sieves as a desiccant, the yield of the reaction drops to 18% (entry 2). Moreover, the use of 0.72 equivalent instead of one equivalent of oxidant (with respect to total copper(ii) concentration) affords the highest radiochemical yield, likely by minimizing oxidation of the fluorinated product. Finally, switching from a PF_6_-based oxidant to an SbF_6_-based oxidant affords more consistent radiochemical yields.

Under the optimal conditions, the substrate scope of C–H fluorination was investigated. As summarized in Table 2, a wide range of 1,3-benzodioxole C–H substrates can be converted to fluorinated products with high RCY. The reaction conditions tolerate many common functional groups, including enolizable ketones (2a), aryl halides (2b), esters (2c, 2f, and 2h), heterocycles (2j, 2m, and 2s), and cyano (2d) and nitro (2l) groups. Additionally, benzylic and allylic C–H substrates can be fluorinated with various efficiencies (2n–2r). Interestingly, substrates 1q and 1r yield the same product through a delocalized allyl radical intermediate, suggesting radical capture selectively occurs at the less sterically hindered position. The potential utility of this method is further demonstrated through the ^18^F-fluorination of Boc-Tadalafil in 74% RCC (2s, Table 2). Although the mono-fluorinated benzodioxole motif is readily accessible *via* our ^18^F-labeling methodology, preliminary stability studies indicate that certain derivatives (*e.g.*, 2d and 2s) are prone to hydrolysis under acidic, basic, and simulated physiological conditions (see the SI). These findings highlight that while the labelling method itself is robust and versatile, the hydrolytic and metabolic instability of some derivatives may limit their direct *in vivo* application. Nevertheless, the methodology provides a general platform to access these fluorinated motifs, which can guide future efforts to design derivatives with enhanced stability for biological imaging applications. As a proof of concept, ^18^F-labeled 2d was isolated with preparative HPLC with 14.5% decay corrected RCY and a molar activity of 1.22 ± 0.42 GBq µmol^−1^ EOS (end of synthesis, Fig. S23–S25).

**Table 2:** Substrate scope for ^18^F C–H fluorination

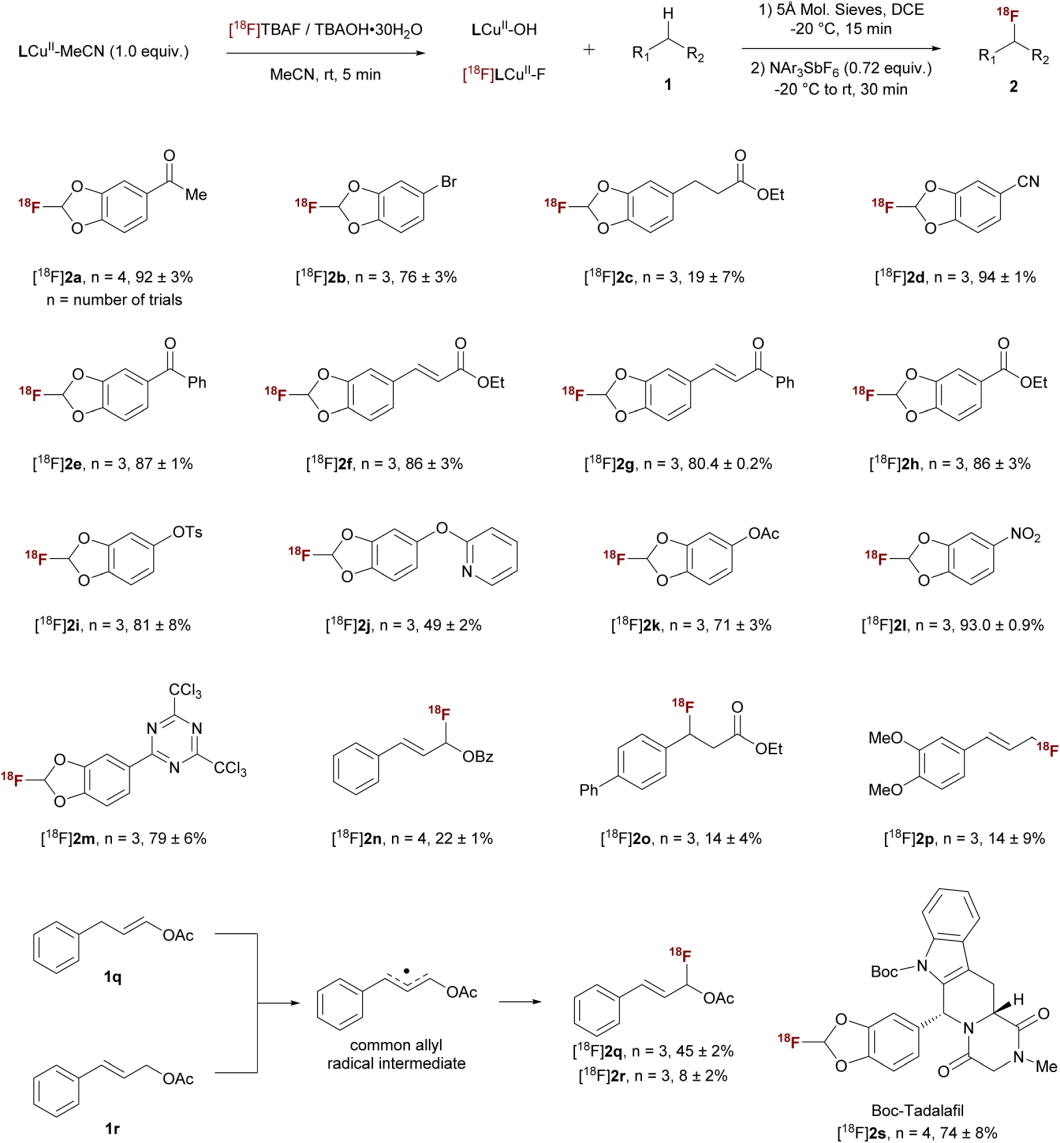

To confirm that the C–H fluorination of 1,3-benzodioxoles still proceeds through the proposed HAT/radical rebound mechanism. We performed a kinetic isotope effect (KIE) study of the reaction between LCu^III^–OH/F and 1a and 3,4-methylenedioxyacetophenone-*d*_2_ (1a-*d*_2_). The KIE values were found to be 11.8 ± 1.8 and 2.95 ± 0.68 for LCu^III^–OH and LCu^III^–F, respectively. These results suggest that the activation of the C–H bond *via* proton-coupled electron transfer (PCET) is the rate-limiting step for both copper complexes.

Notably, 1s contains both a 1,3-benzodioxole motif (Fig. 2B, H_γ_) and other weak benzylic C–H bonds (H_α_ and H_β_), but only the 1,3-benzodioxole C–H bond (C–H_γ_) was activated. This selectivity contrasts the report by Groves *et al.* using manganese(v) oxo fluoride species, which favors C–H positions with low BDFE (Fig. 2A).^6,8^ We attribute this unique selectivity to the selective HAT step by LCu^III^–OH, since HAT is the rate-limiting step as previously reported.^73^ The LCu^III^–OH preferentially activates electron-rich C(sp^3^)–H bonds due to polarity matching. Specifically, the electrophilic nature of formal copper(iii) complexes accelerates C–H activation *via* asynchronous PCET.^27,29,76,77^

**Fig. 2:**
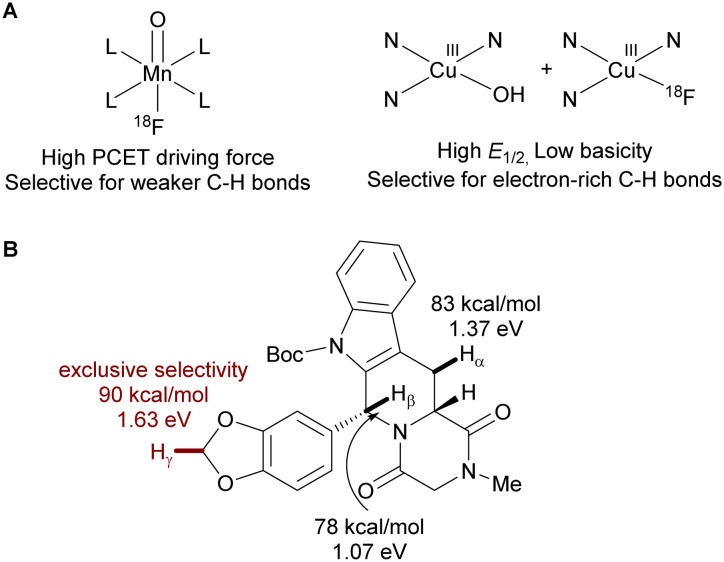
(A) Comparison of selectivity of the O=Mn^V^–F system reported by Groves *et al.* and the Cu^III^ system reported in this work. The selectivity of the O=Mn^V^–F system is dictated by the bond dissociation free energy (BDFE) values of the C–H substrate, while the selectivity of Cu^III^–OH is dictated by the asynchronicity of PCET. (B) Calculated BDFE and asynchronicity factors for three potential C–H fluorination sites.

To evaluate this hypothesis, we calculated the PCET asynchronicity factor *η* of various C–H substrates using density functional theory (DFT, see the SI).^75–77^ The DFT-computed *η* factor shows that PCET from electron-rich benzylic substrates and 1,3-benzodioxole to LCu^III^–OH is highly oxidative and asynchronous (1.445–1.553 V), which is expected to increase the rates of HAT.

We apply this rationale to explain the C–H fluorination selectivity on Boc-Tadalafil 1s, which contains both benzylic (H_α_ and H_β_) and α-ethereal C–H bonds (H_γ_). The calculated BDFE of C_α_–H, C_β_–H, and C_γ_–H shows that the C_γ_–H bond is the strongest (90 kcal mol^−1^). However, the copper(iii) system still selectively fluorinates C_γ_–H over C_α_–H (83 kcal mol^−1^) and C_β_–H (78 kcal mol^−1^) positions (Fig. 2B). This unconventional C–H functionalization selectivity can be explained by the contribution of polarity matching to the barrier of PCET through asynchronicity (*η*), where the greater the asynchronicity the lower the activation barrier. The distinct C–H fluorination selectivity observed here, compared to the work of Groves and Hooker, underscores how new organometallic reagents can expand the scope of C–H ^18^F-fluorination, thereby enhancing the structural diversity of ^18^F-radiotracers.

In summary, we developed a novel ^18^F-labeling methodology through the tandem use of copper(iii) hydroxide as a HAT mediator and copper(iii) fluoride as a FAT agent with high RCCs of up to 94%. The decoupled HAT and FAT reactivity of copper(iii) complexes allows for the activation and fluorination of electron-rich C–H bonds, which are not normally accessible by traditional C–H fluorination methods, which favor C–H bonds with low BDFE, such as benzylic and allylic positions. The tandem use of the HAT reagent and fluorine source, in principle, can provide further practicality and generality to C(sp^3^)–H ^18^F-fluorination, *i.e.*, regioselectivity and stereoselectivity. This will be the subject of our future study.

## Author contributions

S. Z. and J. F. conceived the main idea of the project. J. A. Q., A. A., M. A. P. B., J. T. designed the experiment and performed the data analyses. J. A. Q., S. Z., and J. F. prepared the manuscript.

## Conflicts of interest

There are no conflicts to declare.

## Supplementary Material

SC-017-D5SC06381G-s001

## Data Availability

The datasets supporting this article have been uploaded as part of the supplementary information (SI), including synthesis and characterization information, spectroscopic data, and DFT calculations. Supplementary information is available. See DOI: https://doi.org/10.1039/d5sc06381g.
