# Supplementary material for: Distinct hydrogen atom transfer and radical capture reactivity of copper(iii) OH/F complexes enables site-selective C(sp3)–H 18F-fluorination
Source: Chem Sci. 2025 Nov 4;17(1):299–306. doi: 10.1039/d5sc06381g (PMC12606465; doi:10.1039/d5sc06381g)
Supplement: SC-017-D5SC06381G-s001 [file SC-017-D5SC06381G-s001.pdf]

Supplementary Information (SI) for Chemical Science.
This journal is © The Royal Society of Chemistry 2025

Supporting Information

Distinct Hydrogen Atom Transfer and Radical Capture Reactivity of Copper(III) OH/F Complexes Enables Site-Selective C(sp^3)-H ^{18}F-fluorination

Joshua A. Queener,[a] Angela Asor[b], Margaret A. P. Ball[a], Jinghua Tang,[a] Jinda Fan,[b,c,d]* Shiyu Zhang[a]*

[a]Department of Chemistry and Biochemistry, The Ohio State University, 151 W Woodruff Avenue, Columbus, OH 43210, United States

[b]Department of Chemistry, Michigan State University, 578 S Shaw Lane, East Lansing, MI 48824, United States

[c]Department of Radiology, Michigan State University, 846 Service Road, East Lansing, MI 48824, United States

[d]Institute for Quantitative Health Science & Engineering, Michigan State University, East Lansing, MI, 48824, US

*fanjinda@msu.edu

*zhang.8941@osu.edu

Table of Contents

General Experimental Details 3

General Procedure for the ^{19}F fluorination using TBAF as the fluorine source 4

Charaterization of compounds 6

Procedures for the reactions of trityl radical with LCuOH and LCuF 16

Electrochemical Measurements 20

Kinetic studies of HAT by $LCu^{III}X$ 21

Kinetic Isotope Effect of $LCu^{III}X$ 23

Competition HAT of $LCu^{III}F$ vs $LCu^{III}OH$ 26

Investigating potential ligand exhange reactions between $LCu^{II}OH_2$ and $LCu^{III}F$ 28

Stability of 2d at various pH values 28

Stability of ^{19}F-Tadalafil in biologically compatible solvents 31

Experimental Procedures for Radiochemistry 31

Computational Details 55

NMR Spectra 81

General Experimental Details

All syntheses and experiments were performed under a nitrogen atmosphere in an MBraun glovebox or using standard Schlenk techniques unless otherwise noted. Dichloromethane, tetrahydrofuran, acetonitrile, pentane, and diethyl ether were dried and degassed under nitrogen using a Pure Process Technologies (PPT, Nashua, NH) solvent purification system and stored over 4 Å molecular sieves. Benzene-d_6 and chloroform-d_1 (Cambridge Isotope Laboratories, Inc.) were dried over 4 Å molecular sieves prior to use. All glassware was dried at 120 °C prior to use. [N,N'-bis(2,6-diisopropylphenyl)-2,6-pyridinedicarboxamido] acetonitrilecopper(II) (**L**Cu^{II}MeCN), [TBA]**L**Cu^{II}OH, and [NAr_3]PF_6 were synthesized as previously described.[1,2] All other reagents were obtained from reputable suppliers and used without further purification. NMR spectra were recorded on a Bruker Avance NEO 400 MHz instrument, a Bruker Avance III HD 600 MHz instrument, or a Bruker Ascend 700 MHz instrument and referenced to residual solvent peaks (or α,α,α-trifluorotoluene or fluorobenzene for ^{19}F NMR). NMR multiplicities are reported as follows: singlet (s), doublet (d), triplet (t), quartet (q), multiplet (m), and broad signal (br). UV-vis spectra were collected on an Agilent Cary 60 spectrophotometer outfitted with an Unisoku Unispeks cryostat (−100 °C to + 100 °C). High-resolution mass spectra were recorded on a Bruker MicroTOF (ESI). ESI-MS samples were run in chloroform solvent at concentrations < 1 μM. GC analysis was performed on an Agilent 7890B GC equipped with an HP-5 column and FID detector.

Radiochemistry

All chemicals are analytical grade and used without further purification. Ultrapure water was obtained from a Milli-Q water system. Pre-conditioned Sep-PAK® light QMA cartridges were purchased from Synthra GmbH (Hamburg, Germany) and were flushed with 1 ml of water before use. Merck (Darmstadt, MA) Glass-backed thin layer chromatography (TLC) plates coated with silica gel 60 F_{254} were used for radio-TLC analysis. [^{18}F]Fluoride was produced via the ^{18}O(p,n)^{18}F reaction by proton irradiation (40 μA, 2-5 min) of an [^{18}O] H_2O-containing target in a 11 MeV cyclotron. The [^{18}F]Fluoride (ca. 200 mCi) was delivered to the synthesis module in a bolus of [^{18}O]H_2O by a stream of argon. The aqueous solution of [^{18}F]Fluoride was passed through a QMA cartridge (water preconditioning) to trap [^{18}F]Fluoride before elution into the reaction vessel using an acetonitrile solution of TBAOH•30H_2O before being divided for manual methodology experiments. Radio-TLC was performed using an AR-2000 Radio-TLC Imaging Scanner (Eckert & Ziegler, Valencia, CA, USA) and analyzed using RaPET Lab software. Analytical reversed-phase high-performance liquid chromatography (HPLC) was performed on an Agilent 1260 infinity II system (1260 Infinity II Prime LC, Agilent, Santa Clara, CA, USA) and analyzed using OpenLab CD software. The DAD UV absorbance detector and the CsI(Tl) gamma-ray radiation detector (Carroll & Ramsey Instruments, Fort Collins, CO, USA) were added to the HPLC system. HPLC conditions are listed accordingly for each experiment. All radiochemical conversions (RCC) are decay-corrected and measured by radio-TLC. The identities of the ^{18}F-labeled compounds were confirmed by comparison to an authentic ^{19}F standard. The preparation of fluorinated standards **(1, 2,…)** can be found in section S8-S17.

General Procedure for the ^{19}F fluorination using TBAF as the fluorine source

In an N_2 glovebox, **L**Cu^{II}MeCN (14.7 mg, 0.025 mmol, 1.0 equiv.), [TBA]**L**Cu^{II}OH (20.2 mg, 0.025 mmol, 1.0 equiv.), TBAF•$3H_2O$ (7.9 mg, 0.025 mmol, 1.0 equiv.), powdered 5 Å molecular sieves (20 mg), and C-H substrate (0.25 mmol, 10.0 equiv.) were added to a 10 mL screw cap test tube equipped with a stir bar. Dichloroethane (1.0 ml) was added to dissolve the reagents. The solution was stirred for 10 minutes before being cooled to –35 °C. In a separate vial, a solution of $[NAr_3]PF_6$ (Ar = 4-bromophenyl) (0.05 mM) was prepared in dichloroethane and cooled to −35 °C. Then $[NAr_3]PF_6$ solution (1.0 mL, 0.05 mM, 2.0 equiv.) was added to the reaction vessel. The reaction was stirred for 30 minutes while warming up to room temperature. After the reaction, trifluorotoluene (10 μL, 81.44 μmol) was added to the test tube as internal standard. ^{19}F NMR analysis was performed by taking 200 μL of crude reaction mixture and adding 600 μL of chloroform-d_1.

The C-H starting material and the C-F products have the same retention time on TLC. This property prevents the clean separation of the starting material from the product using traditional methods. Our present method uses 10 equivalents of starting material, the excess amount of C-H starting material remaining after the reaction is completed prevents the clean isolation of the desired C-F products To isolate the ^{19}F products for characterization and use as a standard in HPLC an alternative method that consumes all of the C-H starting material was developed utilizing NFSI.

Table 1. Substrate scope for ^{19}F C-H fluorination.

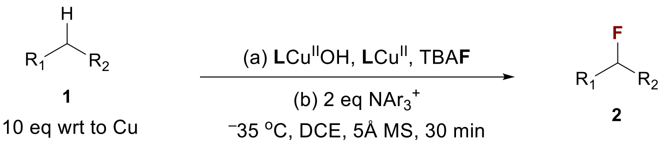

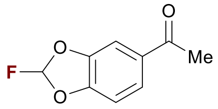

2a, 70%

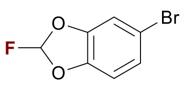

2b, 71%

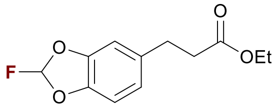

2c, 48%

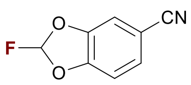

2d, 40%

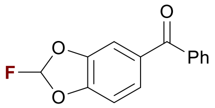

2e, 49%

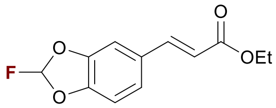

2f, 62%

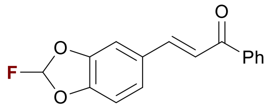

2g, 35%

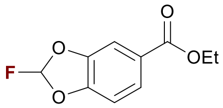

2h, 72%

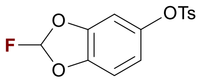

2i, 52%

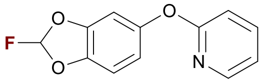

2j, 80%

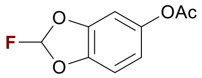

2k, 58%

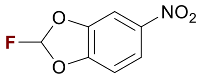

2l, 41%

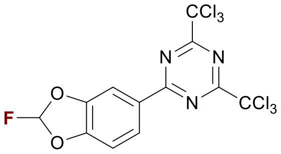

2m, 41%

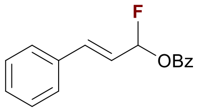

2n, 66%

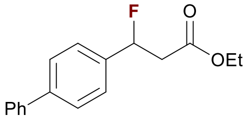

2o, 0%

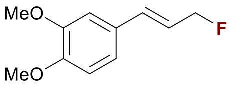

2p, 32%

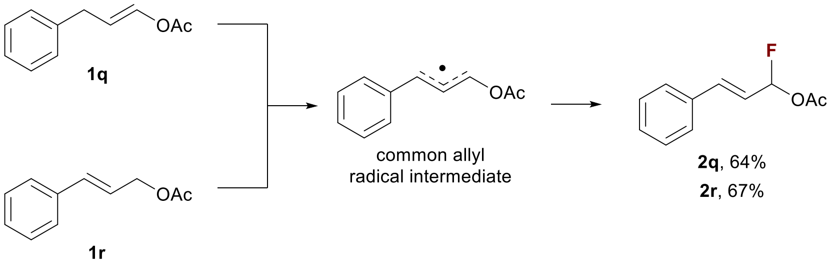

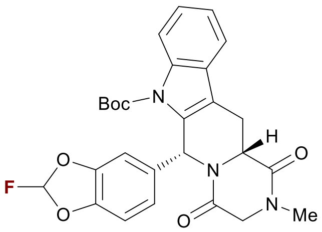

Boc-Tadalafil
2s, 49%

Charaterization of compounds

Preparation of Substrates

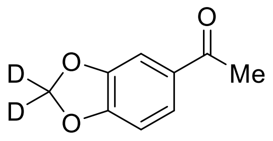

1-(benzo[d][1,3]dioxol-5-yl-2,2-*d*$_2$)ethan-1-one (1a-*d*$_2$) The title compound was synthesized according to a published prodedure; spectral data is consistent with reported literature values.[3]

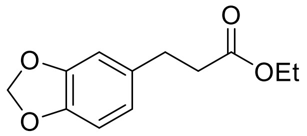

3-Benzo[1,3]dioxol-5-yl-propionic acid ethyl ester (1c) To a dry round bottom flask equipped with a stir bar, 3-(1,3 benzodioxol-5-yl)propionic acid (970 mg, 5.0 mmol, 1.0 equiv.) was added. The solid was dissolved in ethanol (60 mL). Five drops of concentrated sulfuric acid were added as a catalyst. The reaction was heated to reflux overnight. After cooling, the reaction was dried in vacuo and then redissolved in chloroform (40 mL). The chloroform was washed with H_2O (20 mL, three times). The organic phases were combined and dried with $MgSO_4$ and dried in vacuo. The crude product was purified using column chromatography (7:1 hexane: ethyl acetate) to yield **1c** as a yellow oil (0.786 g, 70.8%).

^{1}H NMR (400 MHz, CDCl$_3$) δ 6.79 – 6.57 (m, 1H), 5.92 (s, 1H), 4.13 (q, *J* = 7.1 Hz, 1H), 2.86 (t, *J* = 7.7 Hz, 1H), 2.57 (t, *J* = 7.7 Hz, 1H), 1.24 (t, *J* = 7.1 Hz, 1H)

^{13}C NMR (101 MHz, CDCl$_3$) δ 173.06, 141.10, 139.84, 139.36, 128.89, 127.36, 127.26, 127.15, 60.61, 36.04, 30.75, 14.37.

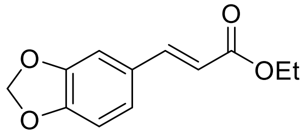

Ethyl (E)-3-(benzo[d][1,3]dioxol-5-yl)acrylate (1f) The title compound was synthesized according to a published procedure; spectral data is consistent with reported literature values.[4]

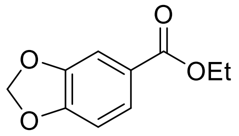

Ethyl 1,3-benzodioxole-5-carboxylate (1h) The title compound was synthesized according to a published procedure; spectral data is consistent with reported literature values.[5]

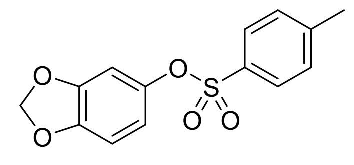

3,4-Methylenedioxyphenyl tosylate (1i) The title compound was synthesized according to a published procedure; spectral data is consistent with reported literature values.[6]

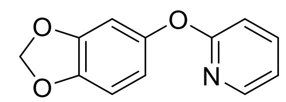

2-(Benzo[d][1,3]dioxol-5-yloxy)pyridine (1j) The title compound was synthesized according to a published procedure; spectral data is consistent with reported literature values.[7]

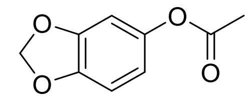

3,4-Methylenedioxyphenyl acetate (1k) The title compound was synthesized according to a published procedure; spectral data is consistent with reported literature values.[8]

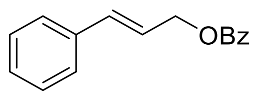

Cinnamyl benzoate (1n) The title compound was synthesized according to a published procedure; spectral data is consistent with reported literature values.[9]

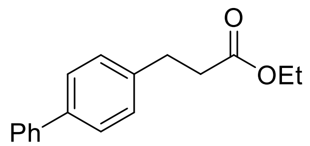

ethyl 3-([1,1'-biphenyl]-4-yl)propanoate (1o) To a dry round bottom flask equipped with a stir bar, 3-(4-biphenylyl)propionic acid (500 mg, 2.2 mmol, 1.0 equiv.) was added. The compound was dissolved in ethanol (60 mL). Five drops of concentrated sulfuric acid were added as a catalyst. The reaction was heated to reflux overnight. After cooling, the reaction was dried in vacuo and then redissolved in chloroform (40 mL). The chloroform was washed with H_2O (20 mL, three times). The organic phases were combined and dried with $MgSO_4$ and dried in vacuo. The crude product was purified using column chromatography (7:1 hexane: ethyl acetate) to yield **1o** as a colorless oil (342 mg, 61.0%).

^{1}H NMR (400 MHz, $CDCl_3$) δ 6.79 – 6.57 (m, 1H), 5.92 (s, 1H), 4.13 (q, J = 7.1 Hz, 1H), 2.86 (t, J = 7.7 Hz, 1H), 2.57 (t, J = 7.7 Hz, 1H), 1.24 (t, J = 7.1 Hz, 1H)

^{13}C NMR (101 MHz, $CDCl_3$) δ 172.99, 147.77, 146.07, 134.55, 121.26, 108.95, 108.38, 100.97, 60.56, 36.42, 30.89, 14.37.

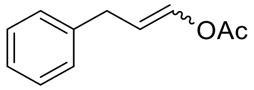

3-Phenylprop-1-en-1-yl acetate (1q) The title compound was synthesized according to a published procedure; spectral data is consistent with reported literature values.[10]

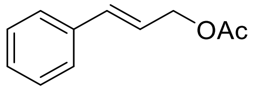

Cinnamyl acetate (1r) The title compound was synthesized according to a published procedure; spectral data is consistent with reported literature values.[11]

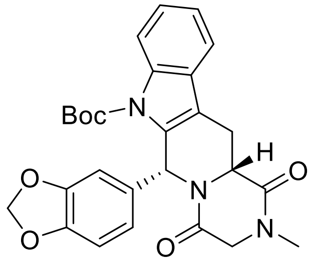

Tert-butyl(6R,12aR)-6-(benzo[d][1,3]dioxol-5-yl)-2-methyl-1,4-dioxo-1,3,4,6,12,12a-hexahydropyrazino[1',2':1,6]pyrido[3,4-b]indole-7(2H)-carboxylate (Boc-Tadalafil, 1s) The title compound was synthesized according to a published procedure; spectral data is consistent with reported literature values.[12]

Synthesis and characterization of ^{19}F-fluorinated product as radio-HPLC standard

The isolation methods are not optimized. These methods were developed due to the inability to separate unreacted C-H starting material from the C-F product under conditions with excess substrate.

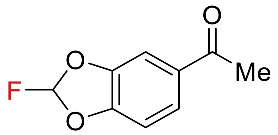

1-(2-Fluorobenzo[d][1,3]dioxol-5-yl)ethan-1-one (2a) To a 10 mL screw cap test tube equipped with a stir bar, N-fluorobenzenesulfonimide (118 mg, 0.375 mmol, 1.5 equiv.) and 1-(benzo[d][1,3]dioxol-5-yl)ethan-1-one (41 mg, 0.25 mmol, 1.0 equiv.) were added. The solids were dissolved in acetonitrile (2 mL). The reaction vessel was sealed and heated to 80 °C for 18 hours. After cooling, the reaction was dried under N_2 flow. The final product was isolated via preparative thin-layer chromatography (8:1 hexane:ethyl acetate) to yield **2a** as a colorless oil (2 mg, 4 %).

^{1}H NMR (400 MHz, $CDCl_3$) δ 7.72 (d, J = 8.3 Hz, 1H), 7.67 (s, 1H), 7.18 (d, $^2J_{H\text{-}F}$ = 88.5 Hz, 1H), 7.10 (d, J = 8.2 Hz, 1H), 2.58k (s, 3H).

^{13}C NMR (176 MHz, $CDCl_3$) δ 196.09, 147.91 (d, $^3J_{C\text{-}F}$ = 3.7 Hz), 144.82 (d, $^3J_{C\text{-}F}$ = 3.1 Hz), 133.45, 125.47, 119.95 (d, $^1J_{C\text{-}F}$ = 277.7 Hz), 109.5, 109.25, 26.70.

^{19}F NMR (376 MHz, $CDCl_3$) δ −70.98 (d, $^2J_{H\text{-}F}$ = 88.5 Hz).

HRMS (ESI) (m/z): $[M+H-HF]^+$ Calculated for $C_9H_8FO_3$: 183.0452; found: 183.0449.

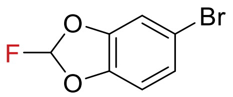

5-Bromo-2-fluorobenzo[d][1,3]dioxole (2b) To a 10 mL screw cap pressure tube equipped with a stir bar, N-fluorobenzenesulfonimide (118 mg, 0.375 mmol, 1.5 equiv.) and 5-bromobenzo[d][1,3]dioxole (31 µL, 0.25 mmol, 1.0 equiv.) were added. The solids were dissolved in acetonitrile (2 mL). The reaction vessel was sealed and heated to 60 °C for 48 hours. The reaction was brought into an N_2 glovebox, extracted with hexane (5 mL, three times), and dried in vacuo to yield **2b** as a yellow oil (5 mg, 9%).

^{1}H NMR (700 MHz, CDCl$_3$) δ 7.20 (d, J = 1.8 Hz, 1H), 7.15 (dd, J = 8.4, 1.8 Hz, 1H), 7.11 (d, $^2J_{H\text{-}F}$ = 88.8 Hz, 1H), 6.92 (d, J = 8.4 Hz, 1H).

^{13}C NMR (176 MHz, CDCl$_3$) δ 145.06, 143.58 (d, $^3J_{C\text{-}F}$ = 3.8 Hz), 126.00, 119.82 (d, $^1J_{C\text{-}F}$ = 277.5 Hz), 114.89, 113.53, 110.86.

^{19}F NMR (565 MHz, CDCl$_3$) δ −70.84 (d, $^2J_{H\text{-}F}$ = 88.8 Hz).

HRMS (ESI) (m/z): $[M+H\text{-}HF]^+$ Calculated for $C_7H_4BrO_2$: 198.9389; found: 198.9389.

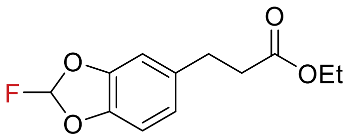

Ethyl 3-(2-fluorobenzo[d][1,3]dioxol-5-yl)propanoate (2c) To a 10 mL screw cap test tube equipped with a stir bar, N-fluorobenzenesulfonimide (118 mg, 0.375 mmol, 1.5 equiv.), copper(II) triflate (10 mg, 0.0275 mmol, 0.11 equiv.) and ethyl 3-(benzo[d][1,3]dioxol-5-yl)propanoate (55.56 mg, 0.25 mmol, 1.0 equiv.) were added. The solids were dissolved in acetonitrile (2 mL). The reaction was stirred for 2 hours followed by the addition of tetrakis(acetonitrile)copper(I) hexafluorophosphate (93.2 mg, 0.25 mmol, 1.0 equiv.) and triethylamine (100 µL, 2.8 equiv.). The reaction was stirred for 30 min before being extracted with hexane (5 mL, three times) and dried in vacuo to yield **2c** as a yellow oil (3 mg, 5%).

^{1}H NMR (700 MHz, CDCl$_3$) δ 7.09 (d, $^2J_{H\text{-}F}$ = 90.0 Hz, 1H), 6.94 (d, J = 8.0 Hz, 1H), 6.91 (s, 1H), 6.84 (d, J = 7.9 Hz, 1H), 4.12 (q, J = 7.1 Hz, 2H), 2.93 (t, J = 7.7 Hz, 2H), 2.59 (t, J = 7.8 Hz, 2H), 1.23 (t, J = 7.2 Hz, 3H).

^{13}C NMR (176 MHz, CDCl$_3$) δ 172.78, 144.27 (d, $^3J_{C\text{-}F}$ = 3.1 Hz), 142.61 (d, $^3J_{C\text{-}F}$ = 3.1 Hz), 136.11, 122.79, 119.59 (d, $^1J_{C\text{-}F}$ = 275.3 Hz), 109.88, 109.45, 60.66, 36.30, 30.88, 14.35.

^{19}F NMR (565 MHz, CDCl$_3$) δ −70.75 (d, $^2J_{H\text{-}F}$ = 90.3 Hz).

HRMS (ESI) (m/z): $[M+H\text{-}HF]^+$ Calculated for $C_{12}H_{13}O_4$: 221.0808 ; found: 221.0808.

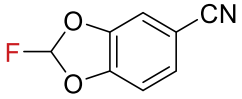

2-Fluorobenzo[d][1,3]dioxole-5-carbonitrile (2d) To a 10 mL screw cap test tube equipped with a stir bar, N-fluorobenzenesulfonimide (118 mg, 0.375 mmol, 1.5 equiv.) and benzo[d][1,3]dioxole-5-carbonitrile (36.8 mg, 0.25 mmol, 1.0 equiv.) were added. The solids were dissolved in acetonitrile (2 mL). The reaction vessel was sealed and heated to 80 °C for 24 hours. After cooling, the reaction was dried under N_2 flow. The final product was isolated via preparative thin-layer chromatography (8:1 hexane:ethyl acetate) to yield **2d** as a white solid (5 mg, 10%).

^{1}H NMR (600 MHz, CDCl$_3$) δ 7.41 (dd, J = 8.2, 1.6 Hz, 1H), 7.31 (d, J = 1.6 Hz, 1H), 7.21 (d, $^2J_{H\text{-}F}$ = 80.8 Hz, 1H), 7.13 (d, J = 1.5 Hz, 1H).

^{13}C NMR (151 MHz, CDCl$_3$) δ 147.75 (d, $^3J_{C\text{-}F}$ = 3.7 Hz), 144.59 (d, $^3J_{C\text{-}F}$ = 3.7 Hz), 129.22, 119.93 (d, $^1J_{C\text{-}F}$ = 279.9 Hz), 118.27, 113.12, 110.60, 107.03.

^{19}F NMR (376 MHz, CDCl$_3$) δ −71.07 (d, $^2J_{H\text{-}F}$ = 87.2 Hz).

HRMS (ESI) (m/z): $[M+H]^+$ Calculated for $C_8H_5FNO_2$: 166.0298 ; found: 166.0299.

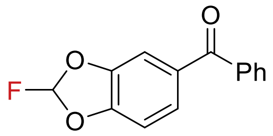

(2-Fluorobenzo[d][1,3]dioxol-5-yl)(phenyl)methanone (2e) To a 10 mL screw cap test tube equipped with a stir bar, N-fluorobenzenesulfonimide (118 mg, 0.375 mmol, 1.5 equiv.) and benzo[d][1,3]dioxol-5-yl(phenyl)methanone (56.6 mg, 0.25 mmol, 1.0 equiv.) were added. The solids were dissolved in acetonitrile (2 mL). The reaction vessel was sealed and heated to 80 °C for 24 hours. After cooling, the reaction was dried under N_2 flow. The final product was isolated via preparative thin-layer chromatography (10:1 hexane:ethyl acetate) to yield **2e** as a colorless oil (6 mg, 10%).

^{1}H NMR (600 MHz, CDCl$_3$) δ 7.78 – 7.74 (m, 2H), 7.63 – 7.54 (m, 3H), 7.52 – 7.46 (m, 2H), 7.21 (d, $^2J_{H\text{-}F}$ = 88.5 Hz, 1H), 7.12 (d, J = 8.1 Hz, 1H).

^{13}C NMR (151 MHz, CDCl$_3$) δ 195.03, 147.60 (d, $^3J_{C\text{-}F}$ = 3.6 Hz), 144.52 (d, $^3J_{C\text{-}F}$ = 3.4 Hz), 137.77, 133.47, 132.54, 129.97, 128.51, 127.36, 119.96 (d, $^1J_{C\text{-}F}$ = 277.8 Hz), 111.42, 109.13.

^{19}F NMR (565 MHz, CDCl$_3$) δ −70.87 (d, $^2J_{H\text{-}F}$ = 88.1 Hz).

HRMS (ESI) (m/z): $[M+H]^+$ Calculated for $C_{14}H_{10}FO_3$: 245.0608 ; found: 245.0607.

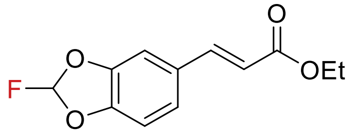

Ethyl (E)-3-(2-fluorobenzo[d][1,3]dioxol-5-yl)acrylate (2f) To a 10 mL screw cap pressure tube equipped with a stir bar, N-fluorobenzenesulfonimide (118 mg, 0.375 mmol, 1.5 equiv.) and ethyl (E)-3-(benzo[d][1,3]dioxol-5-yl)acrylate (47.5 mg, 0.25 mmol, 1.0 equiv.) were added. The solids were dissolved in acetonitrile (2 mL). The reaction vessel was sealed and heated to 80 °C for 24 hours. The reaction vessel was brought back into an N_2 glovebox followed by the addition of tetrakis(acetonitrile)copper(I) hexafluorophosphate (93.2 mg, 0.25 mmol, 1.0 equiv.) and triethylamine (100 µL, 2.8 equiv.). The reaction was stirred for 30 min before being extracted with hexane (5 mL, three times) and dried in vacuo to yield **2f** as a colorless oil (6 mg, 10%).

^{1}H NMR (700 MHz, CDCl$_3$) δ 7.63 (d, J = 15.9 Hz, 1H), 7.25 (s, 1H), 7.19 (dd, J = 8.2, 1.7 Hz, 1H), 7.15 (d, $^2J_{H\text{-}F}$ = 88.9 Hz, 1H), 7.05 (d, J = 8.2 Hz, 1H), 6.33 (d, J = 15.9 Hz, 1H), 4.26 (q, J = 7.2 Hz, 2H), 1.34 (t, J = 7.1 Hz, 3H).

^{13}C NMR (176 MHz, CDCl$_3$) δ 167.01, 145.76 (d, $^3J_{C\text{-}F}$ = 3.8 Hz), 145.06 (d, $^3J_{C\text{-}F}$ = 3.6 Hz), 143.76, 130.51, 124.98, 120.61, 119.82 (d, $^1J_{C\text{-}F}$ = 277.1 Hz), 117.88, 109.91, 108.06, 60.73, 14.48.

^{19}F NMR (565 MHz, CDCl$_3$) δ −70.94 (d, $^2J_{H\text{-}F}$ = 88.1 Hz).

HRMS (ESI) (m/z): $[M+H]^+$ Calculated for $C_{12}H_{12}FO_4$: 239.0714 ; found: 239.0714.

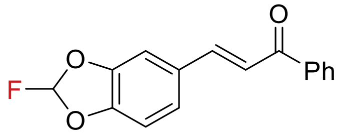

(E)-3-(2-fluorobenzo[d][1,3]dioxol-5-yl)-1-phenylprop-2-en-1-one (2g) To a 10 mL screw cap test tube equipped with a stir bar, N-fluorobenzenesulfonimide (118 mg, 0.375 mmol, 1.5 equiv.) and (E)-3-(benzo[d][1,3]dioxol-5-yl)-1-phenylprop-2-en-1-one (63.1 mg, 0.25 mmol, 1.0 equiv.) were added. The solids were dissolved in acetonitrile (2 mL). The reaction vessel was sealed and heated to 80 °C for 24 hours. The reaction vessel was brought back into an N_2 glovebox followed by the addition of tetrakis(acetonitrile)copper(I) hexafluorophosphate (93.2 mg, 0.25 mmol, 1.0 equiv.) and triethylamine (100 µL, 2.8 equiv.). The reaction was stirred for 30 min before being extracted with pentane (5 mL, three times) and dried in vacuo to yield **2g** as a colorless oil (5 mg, 7%).

^{1}H NMR (600 MHz, CDCl$_3$) δ 8.04 – 7.99 (m, 2H), 7.78 (d, J = 15.6 Hz, 1H), 7.63 – 7.57 (m, 1H), 7.55 – 7.49 (m, 2H), 7.44 (d, J = 15.6 Hz, 1H), 7.39 (d, J = 1.7 Hz, 1H), 7.31 (dd, J = 8.1, 1.7 Hz, 1H), 7.17 (d, $^2J_{H-F}$ = 88.8 Hz, 1H), 7.09 (d, J = 8.2 Hz, 1H).

^{13}C NMR (176 MHz, CDCl$_3$) δ 190.34, 146.04 (d, $^3J_{C-F}$ = 3.4 Hz), 145.06 (d, $^3J_{C-F}$ = 3.2 Hz), 144.05, 138.31, 133.01, 130.95, 128.82, 128.62, 125.69, 121.55, 119.86 (d, $^1J_{C-F}$ = 277.1 Hz), 110.02, 108.26.

^{19}F NMR (565 MHz, CDCl$_3$) δ −70.95 (d, $^2J_{H-F}$ = 88.9 Hz).

HRMS (ESI) (m/z): $[M+H]^+$ Calculated for $C_{16}H_{12}FO_3$: 271.0765 ; found: 271.0762.

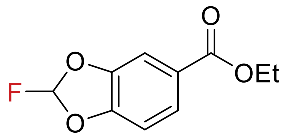

Ethyl 2-fluorobenzo[d][1,3]dioxole-5-carboxylate (2h) To a 10 mL screw cap test tube equipped with a stir bar, N-fluorobenzenesulfonimide (118 mg, 0.375 mmol, 1.5 equiv.) and ethyl benzo[d][1,3]dioxole-5-carboxylate (40.4 µl, 0.25 mmol, 1.0 equiv.) were added. The solids were dissolved in acetonitrile (2 mL). The reaction vessel was sealed and heated to 80 °C for 24 hours. After cooling, the reaction was dried under N_2 flow. The final product was isolated via preparative thin-layer chromatography (20:1 hexane:ethyl acetate) to yield **2h** as a colorless oil (6 mg, 11%).

^{1}H NMR (700 MHz, CDCl$_3$) δ 7.37 (d, J = 8.4 Hz, 1H), 6.80 (s, 1H), 6.71 (d, $^2J_{H-F}$ = 88.7 Hz, 1H), 6.62 (d, J = 8.4 Hz, 1H), 3.91 (q, J = 7.1 Hz, 2H), 0.93 (t, J = 7.1 Hz, 3H).

^{13}C NMR (176 MHz, CDCl$_3$) δ 165.68, 147.72 (d, $^3J_{C-F}$ = 3.0 Hz), 144.32 (d, $^3J_{C-F}$ = 3.1 Hz), 126.25, 126.22, 119.93 (d, $^1J_{C-F}$ = 277.5 Hz), 110.97, 109.24, 61.37, 14.46.

^{19}F NMR (565 MHz, CDCl$_3$) δ −70.88 (d, $^2J_{H-F}$ = 88.5 Hz).

HRMS (ESI) (m/z): $[M+H]^+$ Calculated for $C_{10}H_{10}FO_4$: 213.0557 ; found: 213.0557.

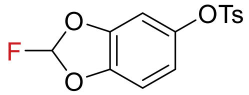

2-Fluorobenzo[d][1,3]dioxol-5-yl 4-methylbenzenesulfonate (2i) To a 10 mL screw cap test tube equipped with a stir bar, N-fluorobenzenesulfonimide (118 mg, 0.375 mmol, 1.5 equiv.) and benzo[d][1,3]dioxol-5-yl 4-methylbenzenesulfonate (73.1 mg, 0.25 mmol, 1.0 equiv.) were added. The solids were dissolved in acetonitrile (2 mL). The reaction vessel was sealed and heated to 80 °C for 48 hours. After cooling, the reaction was dried under N_2 flow. The final product was isolated via preparative thin-layer chromatography (7:1 hexane:ethyl acetate) to yield **2i** as a colorless oil (5 mg, 6%).

^{1}H NMR (700 MHz, CDCl$_3$) δ 7.71 (d, J = 8.3 Hz, 2H), 7.33 (d, J = 8.0 Hz, 2H), 7.11 (d, $^2J_{H-F}$ = 88.8 Hz, 1H), 6.90 (d, J = 8.6 Hz, 1H), 6.77 (d, J = 2.3 Hz, 1H), 6.59 (dd, J = 8.6, 2.3 Hz, 1H), 2.46 (s, 3H).

^{13}C NMR (176 MHz, CDCl$_3$) δ 145.66, 144.79, 144.42 (d, $^3J_{C-F}$ = 3.1 Hz), 142.91 (d, $^3J_{C-F}$ = 2.7 Hz), 131.99, 129.88, 128.59, 120.06 (d, $^1J_{C-F}$ = 277.8 Hz), 116.92, 109.24, 105.56, 21.76.

^{19}F NMR (565 MHz, CDCl$_3$) δ −70.76 (d, $^2J_{H-F}$ = 88.4 Hz).

HRMS (ESI) (m/z): $[M+H-HF]^+$ Calculated for $C_{14}H_{11}O_5S$: 291.0321 ; found: 291.0321.

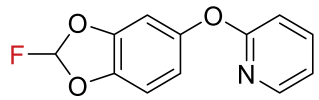

2-((2-Fluorobenzo[d][1,3]dioxol-5-yl)oxy)pyridine (2j) To a 10 mL screw cap test tube equipped with a stir bar, N-fluorobenzenesulfonimide (118 mg, 0.375 mmol, 1.5 equiv.), copper(II) triflate (10 mg, 0.0275 mmol, 0.11 equiv.) and 2-(benzo[d][1,3]dioxol-5-yloxy)pyridine (53.4 mg, 0.25 mmol, 1.0 equiv.) were added. The solids were dissolved in acetonitrile (2 mL). The reaction was brought out of the glovebox and heated to 60 °C for 2 hours. After cooling, the reaction was dried under N_2 flow. The final product was isolated via preparative thin-layer chromatography (4:1 hexane:ethyl acetate) to yield **2j** as a white solid (4 mg, 7%)

^{1}H NMR (700 MHz, $CDCl_3$) δ 8.18 (dd, J = 5.2, 2.0 Hz, 1H), 7.72 – 7.62 (m, 1H), 7.13 (d, $^2J_{H\text{-}F}$ = 89.6 Hz, 1H), 7.03 (d, J = 8.5 Hz, 1H), 7.00 (ddd, J = 7.3, 5.0, 0.9 Hz, 1H), 6.92 (d, J = 8.2, 1H), 6.90 (d, J = 2.4 Hz, 2H), 6.80 (dd, J = 8.5, 2.3 Hz, 2H).

^{13}C NMR (176 MHz, $CDCl_3$) δ 163.88, 149.64, 147.80, 144.73 (d, $^3J_{C\text{-}F}$ = 3.0 Hz), 141.15 (d, $^3J_{C\text{-}F}$ = 2.9 Hz), 139.69, 124.98, 120.17 (d, $^1J_{C\text{-}F}$ = 276.5 Hz), 118.76, 115.58, 111.57, 109.63, 104.79.

^{19}F NMR (565 MHz, $CDCl_3$) δ −70.34 (d, $^2J_{H\text{-}F}$ = 89.6 Hz).

HRMS (ESI) (m/z): $[M+H]^+$ Calculated for $C_{12}H_9FNO_3$: 234.0561 ; found: 234.0560.

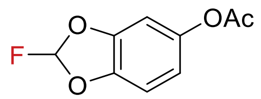

2-Fluorobenzo[d][1,3]dioxol-5-yl acetate (2k) To a 10 mL screw cap test tube equipped with a stir bar, N-fluorobenzenesulfonimide (118 mg, 0.375 mmol, 1.5 equiv), copper(II) triflate (10 mg, 0.0275 mmol, 0.11 equiv.) and benzo[d][1,3]dioxol-5-yl acetate (48 mg, 0.25 mmol, 1.0 equiv.) were added. The solids were dissolved in acetonitrile (2 mL). The reaction was stirred for 1 hour followed by the addition of tetrakis(acetonitrile)copper(I) hexafluorophosphate (93.2 mg, 0.25 mmol, 1.0 equiv.) and triethylamine (100 μL, 2.8 equiv.). The reaction was stirred for 30 min before being extracted with pentane (5 mL, three times) and dried in vacuo to yield **2k** as a yellow oil (4 mg, 8%).

^{1}H NMR (700 MHz, $CDCl_3$) δ 7.13 (d, $^2J_{H\text{-}F}$ = 89.2 Hz, 1H), 7.01 (s, 1H), 6.85 (d, J = 2.3 Hz, 1H), 6.73 (dd, J = 8.5, 2.3 Hz, 1H), 2.29 (s, 3H).

^{13}C NMR (176 MHz, $CDCl_3$) δ 169.68, 146.14, 141.91, 120.14 (d, $^1J_{C\text{-}F}$ = 277.1 Hz), 115.81, 109.39, 104.90, 21.15.

^{19}F NMR (565 MHz, $CDCl_3$) δ −70.65 (d, $^2J_{H\text{-}F}$ = 88.9 Hz).

HRMS (ESI) (m/z): $[M+H-HF]^+$ Calculated for $C_9H_7O_4$: 179.0338 ; found: 179.0339.

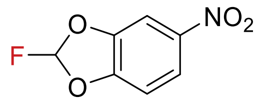

2-Fluoro-5-nitrobenzo[d][1,3]dioxole (2l) To a 10 mL screw cap test tube equipped with a stir bar, N-fluorobenzenesulfonimide (118 mg, 0.375 mmol, 1.5 equiv.) and 5-nitrobenzo[d][1,3]dioxole (41.8 mg, 0.25 mmol, 1.0 equiv.) were added. The solids were dissolved in acetonitrile (2 mL). The reaction vessel was sealed and heated to 80 °C for 4 days. After cooling, the reaction was dried under N_2 flow. The final

product was isolated via preparative thin-layer chromatography (7:1 hexane:ethyl acetate) to yield **2l** as a white solid (3 mg, 6%).

^{1}H NMR (700 MHz, CDCl$_3$) δ 8.08 (dd, J = 8.8, 2.1 Hz, 1H), 7.94 (d, J = 2.3 Hz, 1H), 7.26 (d, $^2J_{H-F}$ = 85.3 Hz, 1H), 7.15 (d, J = 8.7 Hz, 1H).

^{13}C NMR (176 MHz, CDCl$_3$) δ 149.10, 144.71 (d, $^3J_{C-F}$ = 3.4 Hz), 144.11, 120.73, 120.66 (d, $^1J_{C-F}$ = 280.1 Hz), 109.24, 106.12.

^{19}F NMR (565 MHz, CDCl$_3$) δ −70.97 (d, $^2J_{H-F}$ = 86.6 Hz).

HRMS (ESI) (m/z): $[M+H]^+$ Calculated for $C_7H_5FNO_4$: 186.0197 ; found: 186.0197.

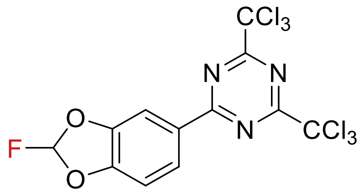

2-(2-Fluorobenzo[d][1,3]dioxol-5-yl)-4,6-bis(trichloromethyl)-1,3,5-triazine (2m) To a 10 mL screw cap test tube equipped with a stir bar, N-fluorobenzenesulfonimide (118 mg, 0.375 mmol, 1.5 equiv.), copper(II) OTf (10 mg, 0.0275 mmol, 0.11 equiv.) and 2-(benzo[d][1,3]dioxol-5-yl)-4,6-bis(trichloromethyl)-1,3,5-triazine (108.9 mg, 0.25 mmol, 1.0 equiv.) were added. The solids were dissolved in acetonitrile (2 mL). The reaction was brought out of the glovebox and heated to 60 °C for 8 hours. After cooling, the reaction was dried under N_2 flow. The final product was isolated via preparative thin-layer chromatography (10:1 hexane:ethyl acetate) to yield **2m** as a white solid (5 mg, 4%).

^{1}H NMR (700 MHz, CDCl$_3$) δ 8.55 (dd, J = 8.4, 1.7 Hz, 1H), 8.36 (d, J = 1.7 Hz, 1H), 7.25 (d, $^2J_{H-F}$ = 88.0 Hz, 1H), 7.24 (d, J = 8.4 Hz, 1H).

^{13}C NMR (176 MHz, CDCl$_3$) δ 175.28, 173.89, 149.56 (d, $^3J_{C-F}$ = 3.2 Hz), 145.30 (d, $^3J_{C-F}$ = 3.0 Hz), 129.31, 127.44, 120.14 (d, $^1J_{C-F}$ = 278.5 Hz), 111.05, 110.24, 95.00.

^{19}F NMR (565 MHz, CDCl$_3$) δ −70.94 (d, $^2J_{H-F}$ = 87.7 Hz).

HRMS (ESI) (m/z): $[M+H]^+$ Calculated for $C_{12}H_5Cl_6FN_3O_2$: 453.8462 ; found: 453.8465.

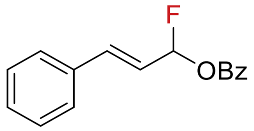

(E)-1-fluoro-3-phenylallyl benzoate (2n) Benzoic acid (61.05 mg, 0.5 mmol, 1.0 equiv.) and cinnamaldehyde (0.188 mL, 1.5 mmol, 3.0 equiv.) were added to a N_2-filled round bottom flask and dissolved in dichloromethane (5 mL). The reaction was cooled to 0 °C in an ice bath. Diethyl amino sulfur trifluoride (DAST) (0.132 mL, 1.0 mmol, 2.0 equiv.) was added dropwise. The reaction was allowed to warm up to room temperature and reacted for 4 hours. The reaction mixture was then poured over an ice / $NaHCO_3$ solution, and the organic phase was collected and washed with saturated $NaHCO_3$ solution (15 mL, three times). The aqueous layers were combined and extracted with dichloromethane (30 mL, three times). The organic layers were combined and dried with $NaSO_4$ and dried in vacuo. The resulting oil was brought into an N_2 glovebox and the product was isolated via preparative thin-layer chromatography (10:1 hexane:ethyl acetate) to yield **2n** as a yellow oil (5 mg, 4%).

^{1}H NMR (700 MHz, CDCl$_3$) δ 8.14 (d, *J* = 6.8, 2H), 7.66 – 7.59 (m, 1H), 7.52 – 7.44 (m, 4H), 7.36 (dt, J = 28.4, 7.2 Hz, 3H), 7.08 (dd, J = 55.4, 5.5 Hz, 1H), 6.97 (dd, J = 16.1, 3.5 Hz, 1H), 6.41 (ddd, J = 16.0, 9.1, 5.9 Hz, 1H).

^{13}C NMR (176 MHz, CDCl$_3$) δ 164.56, 136.61 (d, $^3J_{C\text{-}F}$ = 11.1 Hz), 135.01, 134.05, 130.28, 129.28, 128.92, 128.72, 127.31, 121.46 (d, $^2J_{C\text{-}F}$ = 24.2 Hz), 102.57 (d, $^1J_{C\text{-}F}$ = 217.4 Hz).

^{19}F NMR (565 MHz, CDCl$_3$) δ −122.23 (ddd, J = 54.9, 9.2, 3.9 Hz).

HRMS (ESI) (m/z): $[M+H-HF]^+$ Calculated for $C_{16}H_{13}O_2$: 237.0910 ; found: 237.0911.

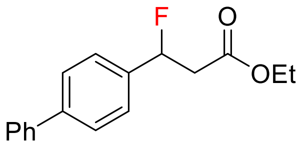

Ethyl 3-([1,1'-biphenyl]-4-yl)-3-fluoropropanoate (2o) In an N_2 glovebox, Selectfluor (177 mg, 0.5 mmol, 2.0 equiv.), ethyl 3-([1,1'-biphenyl]-4-yl)propanoate (63.5 mg, 0.25 mmol, 1.0 equiv.) and 9-fluorenone (9 mg, 0.05 mmol, 0.2 equiv.) were added to a 10 mL screw cap test tube and dissolved in acetonitrile (2 mL). The reaction mixture was brought out of the box and placed in front of an 11 watt light bulb for 4 hours. The reaction was dried and the final product was isolated via preparative thin layer chromatography (10:1 hexane:ethyl acetate) yielding **2o** as a clear oil (5 mg, 8 %)

^{1}H NMR (700 MHz, CDCl$_3$) δ 7.65 – 7.57 (m, 4H), 7.48 – 7.43 (m, 4H), 7.39 – 7.35 (m, 1H), 5.97 (ddd, J = 46.9, 9.0, 4.3 Hz, 1H), 4.25 – 4.17 (m, 2H), 3.07 (ddd, J = 16.0, 13.4, 9.1 Hz, 1H), 2.84 (ddd, J = 31.9, 16.0, 4.3 Hz, 1H), 1.28 (t, J = 7.2 Hz, 3H).

^{13}C NMR (176 MHz, CDCl$_3$) δ 169.80 (d, $^3J_{C\text{-}F}$ = 5.1 Hz), 141.97, 140.63, 137.76 (d, $^2J_{C\text{-}F}$ = 19.7 Hz), 128.99, 127.73, 127.55, 127.29, 126.30 (d, $^3J_{C\text{-}F}$ = 6.2 Hz), 90.65 (d, $^1J_{C\text{-}F}$ = 171.8 Hz), 61.18, 42.60 (d, $^2J_{C\text{-}F}$ = 27.3 Hz), 14.31.

^{19}F NMR (376 MHz, CDCl$_3$) δ −172.66 (ddd, J = 46.2, 32.1, 13.5 Hz).

HRMS (ESI) (m/z): $[M+H-HF]^+$ Calculated for $C_{17}H_{17}O_2$: 253.1223 ; found 253.1223.

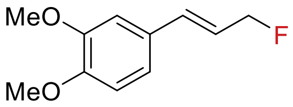

(E)-4-(3-fluoroprop-1-en-1-yl)-1,2-dimethoxybenzene (2p) In an N_2 glovebox, (E)-3-(3,4-dimethoxyphenyl)allyl 4-nitrobenzoate (34.33 mg, 0.1 mmol, 1.0 equiv.), triphenylphosphine (4 mg, 0.015 mmol, 0.15 equiv.), tris(dibenzylideneacetone)dipalladium(0) (3 mg, 0.005 mmol, 0.05 zequiv.) and TBAF•3H_2O (78 mg, 0.25 mmol, 2.5 equiv.) were added to a 20 mL screw top vial and dissolved in tetrahydrofuran (2 mL). The reaction was allowed to stir for 1 hour before being brought out of the glovebox. The reaction was quenched with saturated NH_4Cl solution (5 mL) and extracted with diethyl ether (5 mL, three times). The organic layers were combined and dried with $NaSO_4$ and dried in vacuo. The final product was isolated via preparative thin-layer chromatography (6:1 hexane:ethyl acetate, 5% triethylamine) to yield **2p** as a clear oil (6 mg, 30%).

^{1}H NMR (400 MHz, CDCl$_3$) δ 6.96 (d, J = 7.3 Hz, 2H), 6.83 (d, J = 8.8 Hz, 1H), 6.64 (dd, J = 15.9, 5.6 Hz, 1H), 6.25 (ddt, J = 15.8, 10.9, 6.4 Hz, 1H), 5.01 (ddd, J = 47.2, 6.3, 1.3 Hz, 2H), 3.91 (s, 3H), 3.89 (s, 3H).

^{13}C NMR (176 MHz, CDCl$_3$) δ 149.57, 149.23, 134.81 (d, $^2J_{C\text{-}F}$ = 12.4 Hz), 121.68, 121.59, 120.39 (d, $^3J_{C\text{-}F}$ = 2.5 Hz), 111.22, 109.17, 83.81 (d, $^1J_{C\text{-}F}$ = 161.9 Hz), 56.07, 55.99.

^{19}F NMR (376 MHz, CDCl$_3$) δ −208.35 (tdd, J = 47.1, 11.0, 5.5 Hz).

HRMS (ESI) (m/z): $[M+H]^+$ Calculated for $C_{11}H_{14}FO_2$: 197.0972 ; found 197.0973.

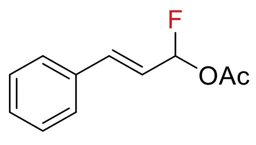

(E)-1-fluoro-3-phenylallyl acetate (2q, 2r) Acetic acid (0.032 mL, 0.5 mmol, 1.0 equiv.) and cinnamaldehyde (0.188 mL, 1.5 mmol, 3.0 equiv.) were added to a N_2-filled round bottom flask and dissolved in dichloromethane (5 mL). The reaction was cooled to 0 °C in an ice bath. Diethyl amino sulfur trifluoride (DAST) (0.132 mL, 1.0 mmol, 2.0 equiv.) was added dropwise. The reaction was allowed to warm up to room temperature and reacted for 4 hours. The reaction mixture was then poured over an ice / $NaHCO_3$ solution, and the organic phase was collected and washed with saturated $NaHCO_3$ solution (15 mL, three times). The aqueous layers were combined and extracted with dichloromethane (30 mL). The organic layers were combined and dried with $NaSO_4$ and dried in vacuo. The resulting oil was brought into an N_2 glovebox and the product was isolated via preparative thin-layer chromatography (10:1 hexane:ethyl acetate) to yield (**2q, 2r**) as a yellow oil (4 mg, 1%)

^{1}H NMR (700 MHz, $CDCl_3$) δ 7.47 – 7.41 (m, 2H), 7.38 – 7.30 (m, 3H), 6.87 – 6.83 (m, 1H), 6.84 (dd, J = 74.6, 4.8 Hz, 1H), 6.25 (ddd, J = 16.2, 9.0, 6.1 Hz, 1H), 2.18 (s, 3H).

^{13}C NMR (176 MHz, $CDCl_3$) δ 168.98, 136.53 (d, $^3J_{C-F}$ = 11.2 Hz), 134.97, 129.27, 128.91, 127.27, 121.32 (d, $^2J_{C-F}$ = 24.1 Hz), 101.98 (d, $^1J_{C-F}$ = 216.5 Hz), 21.08.

^{19}F NMR (377 MHz, $CDCl_3$) δ −121.94 (ddd, J = 55.0, 8.9, 3.6 Hz).

HRMS (ESI) (m/z): $[M+H-HF]^+$ Calculated for $C_{11}H_{11}O_2$: 175.0753 ; found: 175.0753.

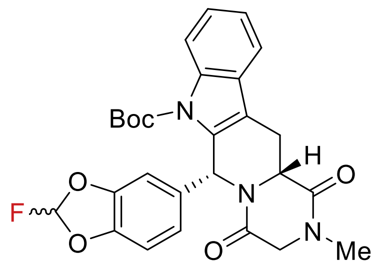

Tert-butyl (6R,12aR)-6-(2-fluorobenzo[d][1,3]dioxol-5-yl)-2-methyl-1,4-dioxo-1,3,4,6,12,12a-hexahydropyrazino[1',2':1,6]pyrido[3,4-b]indole-7(2H)-carboxylate (2s) To a 10 ml screw cap test tube equipped with a stir bar, N-fluorobenzenesulfonimide (118 mg, 0.375 mmol, 1.5 equiv.), copper(II) OTf (10 mg, 0.0275 mmol, 0.11 equiv.) and **1z** (Boc-Tadalafil) (122 mg, 0.25 mmol, 1.0 equiv.) were added. The solids were dissolved in acetonitrile (2 mL). The reaction was stirred for 1 hour followed by the addition of tetrakis(acetonitrile)copper(I) hexafluorophosphate (93.2 mg, 0.25 mmol, 1.0 equiv.) and triethylamine (100 µl, 2.8 equiv.). The reaction was stirred for 30 min before being extracted with pentane (5 mL, three times) and dried in vacuo to yield **2s** as a white solid (4 mg, 3%).

^{1}H NMR (400 MHz, $CDCl_3$) δ 8.12 – 8.02 (m, 1H), 7.60 (dd, J = 6.5, 2.3 Hz, 1H), 7.37 – 7.28 (m, 3H), 7.07 (dd, J = 4.3, 1.8 Hz, 1H), 7.04 (dd, J = 89.8, 3.5 Hz, 1H), 6.99 (ddd, J = 8.4, 4.2, 1.9 Hz, 1H), 6.85 (d, J = 8.3 Hz, 1H), 4.26 (dt, J = 10.5, 4.6 Hz, 1H), 4.14 (d, J = 17.2 Hz, 1H), 3.94 (d, J = 17.3 Hz, 1H), 3.71 (dt, J = 16.4, 4.5 Hz, 1H), 3.16 – 3.06 (m, 1H), 3.04 (s, 4H), 1.59 (s, 8H).

^{13}C NMR (176 MHz, $CDCl_3$) δ 166.59 (dd, J = 22.8, 12.3 Hz), 149.73, 143.46, 136.08 (d, J = 5.7 Hz), 134.06, 127.58, 125.21 (d, J = 3.0 Hz), 124.99, 123.35 (d, J = 3.6 Hz), 123.28 (d, J = 7.1 Hz), 119.64 (d, $^1J_{C-F}$ = 278.1 Hz), 118.71 (d, J = 3.9 Hz), 116.16, 116.09, 115.18, 115.13, 110.20, 110.06, 108.99, 85.01, 55.59, 55.42, 55.26, 52.61 (d, J = 7.9 Hz), 46.25, 33.78, 28.26, 23.17, 23.02.

^{19}F NMR (377 MHz, $CDCl_3$) δ −70.36 (d, J = 89.8 Hz), −70.71 (d, J = 89.6 Hz).

HRMS (ESI) (m/z): $[M+H]^+$ Calculated for $C_{27}H_{26}FN_3O_6$: 508.1878 ; found: 508.1878.

Synthesis of $NAr_3[SbF_6]$ Adapted from a literature procedure.[13] Inside the glovebox, nitrosonium hexafluoroantimonate ($[NO]SbF_6$, 2.126 g, 8 mmol, 1 equiv.) was added to a 250 mL round bottom flask with DCM (30 mL). The $[NO]SbF_6$ suspension was then cooled to −78 °C. A solution of tris(4-bromophenyl)amine (3.8561 g, 8 mmol, 1 equiv.) in DCM (60 mL) was added dropwise to the suspension over the course of 1 hour. After the complete addition of tris(4-bromophenyl)amine the headspace of the flask was evacuated for 3 minutes. Pentane was added until the flask was completely filled to precipitate the product. The resulting solids were collected via vacuum filtration and washed with diethyl ether (50 mL, three times) to yield blue solids (5.15 g, 90% yield). The UV-vis spectrum matches the literature report. The isolated solid can be stored indefinitely at −35 °C under nitrogen atmosphere. The compound is moisture sensitive.

Procedures for the reactions of trityl radical with LCuOH and LCuF

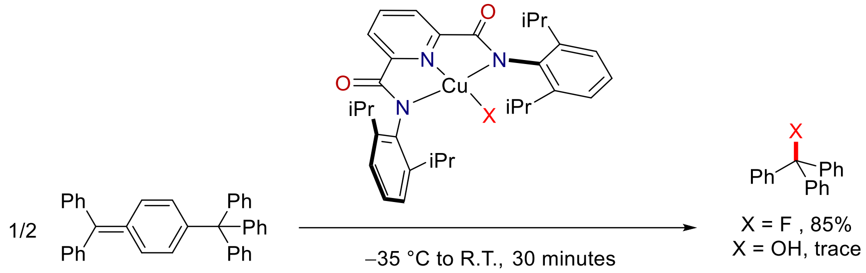

*Procedure for radical capture with **L**Cu^{III}F:* A $CDCl_3$ solution of [TBA]**L**Cu^{II}F (0.250 mL, 10 mM) was added to an NMR tube inside an N_2 glovebox and cooled to –35 °C. A cold solution of $[NAr_3]PF_6$ (0.125 mL, 20 mM) precooled at –35 °C was added to generate **L**Cu^{III}F. The NMR tube was inverted once quickly and stored at –35 °C for 30 minutes. Then a cold solution of Gomberg's dimer (0.250 mL, 5 mM) at –35 °C was added. The tube was inverted once quickly and allowed to sit at room temperature for 30 minutes prior to NMR analysis. ^{19}F NMR was used to quantify the yield of Ph_3C-F due to its instability on silica gel columns.

*Procedure for radical capture with **L**Cu^{III}OH:* A $CDCl_3$ solution of [TBA]**L**Cu^{II}OH (0.250 mL, 10 mM) was added to an NMR tube inside an N_2 glovebox and cooled to –35 °C. A cold solution of $[NAr_3]PF_6$ (0.125 mL, 20 mM) precooled at –35 °C was added to generate **L**Cu^{III}OH. The NMR tube was inverted once quickly and stored at –35 °C for 30 minutes. Then a cold solution of Gomberg's dimer (0.250 mL, 5 mM) at –35 °C was added. The tube was allowed to sit at room temperature for 30 minutes. ^{1}H NMR analysis of the reaction mixture did not show a peak, consistent with the formation of Ph_3C-OH. The reaction mixture was then analyzed with GC-MS. A GC-MS calibration curve was used to quantify the yield of Ph_3C-OH.

*Procedure for radical capture with **L**Cu^{III}F and **L**Cu^{III}OH:* A $CDCl_3$ solution of [TBA]**L**Cu^{II}F (0.125 mL, 20 mM) and a $CDCl_3$ solution of [TBA]**L**Cu^{II}OH (0.125 mL, 20 mM) were added to an NMR tube inside an N_2 glovebox and cooled to –35 °C. A cold solution of $[NAr_3]PF_6$ (0.250 mL, 20 mM) precooled at –35 °C was added to generate **L**Cu^{III}F and **L**Cu^{III}OH. The NMR tube was inverted once quickly and stored at –35 °C for 30 minutes. Then a cold solution of Gomberg's dimer (0.125 mL, 10 mM) at –35 °C was added. The tube was inverted once quickly and allowed to sit at room temperature for 30 minutes prior to NMR analysis. ^{19}F NMR was used to quantify the yield of Ph_3C-F due to its instability on silica gel columns. A GC-MS calibration curve was used to quantify the yield of Ph_3C-OH.

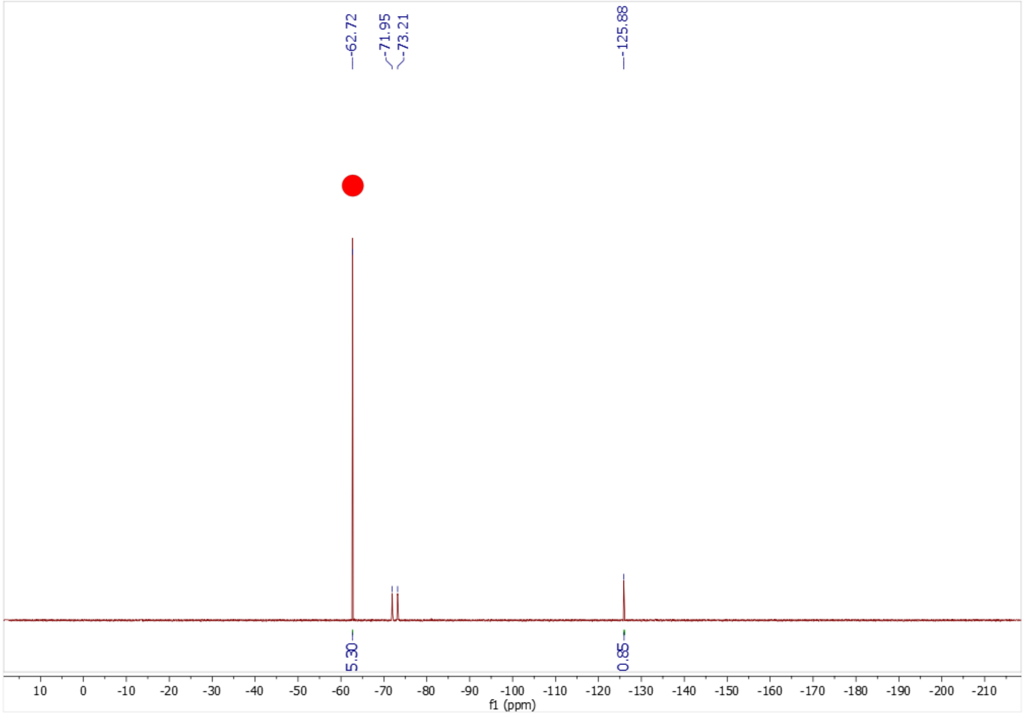

Figure S1. $^{19}F\{^{1}H\}$ NMR (565 MHz, CDCl3) of the reaction between **L**$Cu^{III}F$ (generated from [TBA]**L**$Cu^{II}F$ and $[NAr_3]PF_6$ at −35 °C) and 0.5 equivalents of Gomberg's dimer, revealing the formation of trityl fluoride at 125.9 ppm.[14] A ^{19}F NMR yield of 85% was obtained. The red dot indicates trifluorotoluene, the internal reference standard.

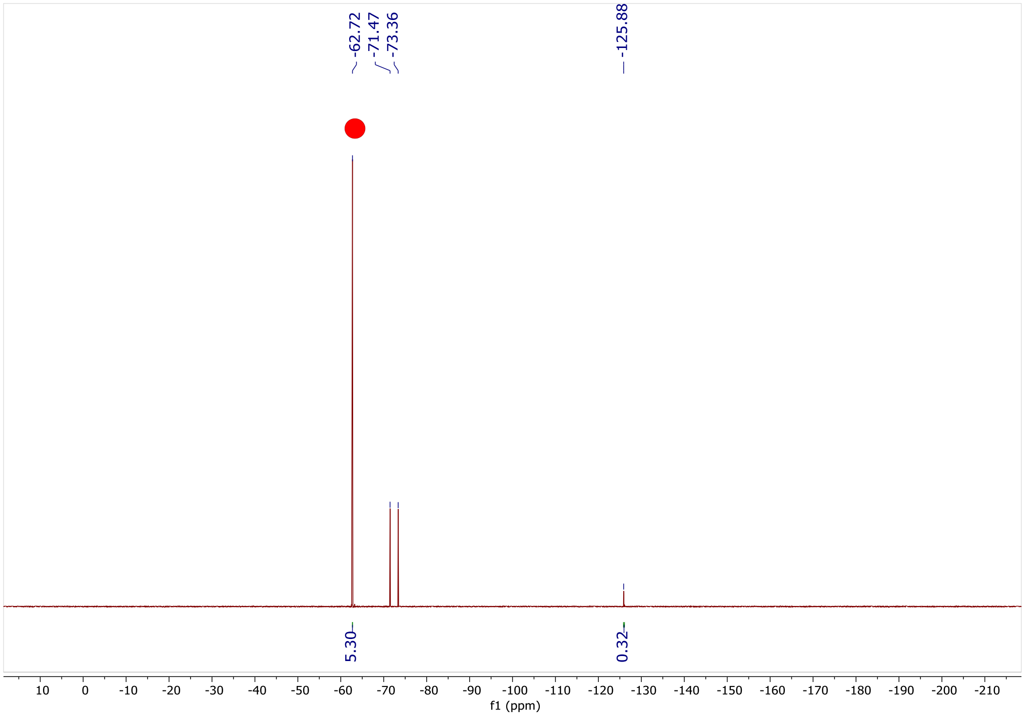

Figure S2. $^{19}F\{^1H\}$ NMR (565 MHz, CDCl3) of the reaction between **L**$Cu^{III}F$ and **L**$Cu^{III}OH$ and 0.5 equivalents of Gomberg's dimer, revealing the formation of trityl fluoride at 125.9 ppm.[14] A ^{19}F NMR yield of 32% was obtained. The red dot indicates trifluorotoluene, the internal reference standard.

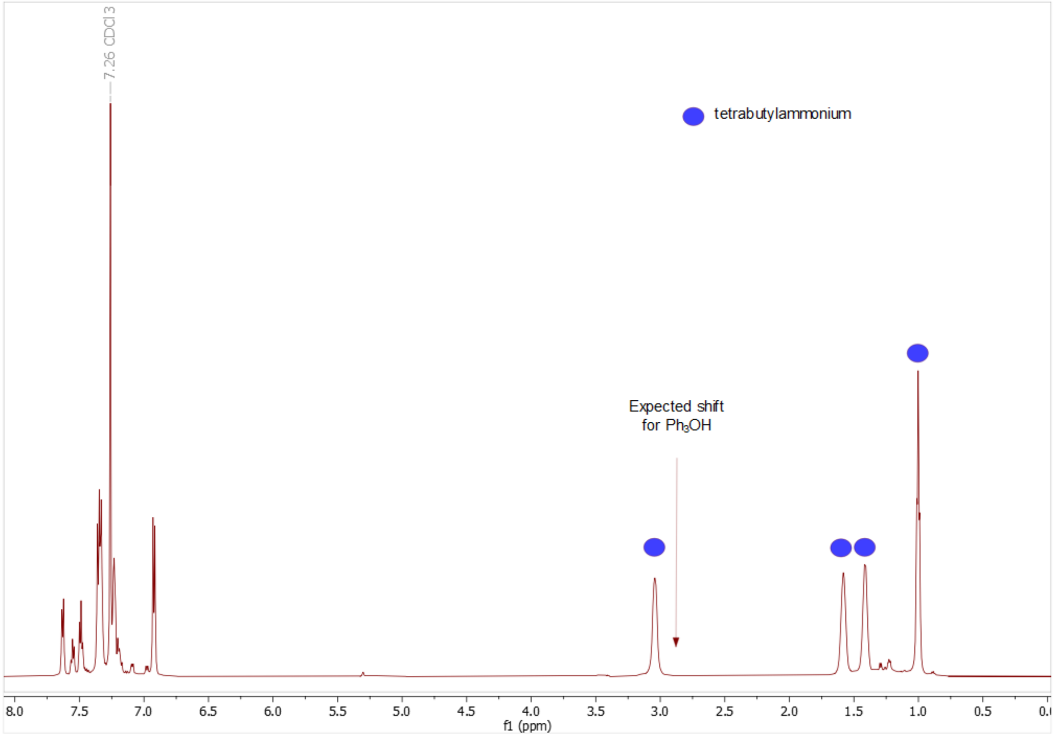

Figure S3. ^1H NMR (600 MHz, CDCl3) of the reaction between **L**$Cu^{III}OH$ (generated from [TBA]**L**$Cu^{II}OH$ and $[NAr_3]PF_6$ at −35 °C) and 0.5 equivalents of Gomberg's dimer, the ^1H NMR shift does not match the reported literature value for trityl hydroxide.[15]

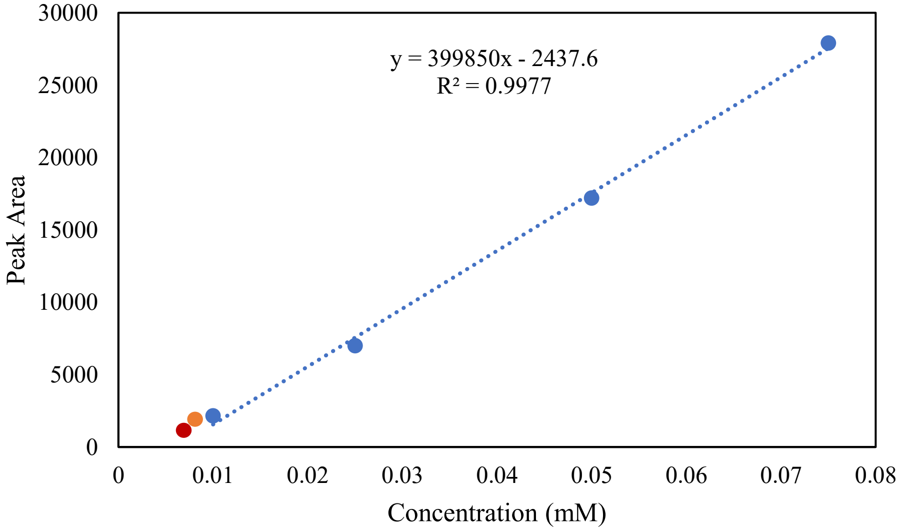

Figure S4. GC-MS calibration curve of Ph_3C-OH standard for the analysis of radical capture by **L**Cu^{III}OH. Analysis of the reaction mixture of **L**Cu^{III}OH with Gomberg's dimer shows a peak of Ph_3C-OH with an area of 1153.1 (indicated by the red dot), suggesting a concentration of 0.0089 mM. This concentration suggests the yield of Ph_3C-OH is ca. 0.89%. Analysis of the competition reaction between **L**Cu^{III}F and **L**Cu^{III}OH shows a peak of Ph_3C-OH with an area of 1478.3 (indicated by the orange dot), suggesting a concentration of 0.0098 mM. This concentration suggests the yield of Ph_3C-OH is ca. 0.97%.

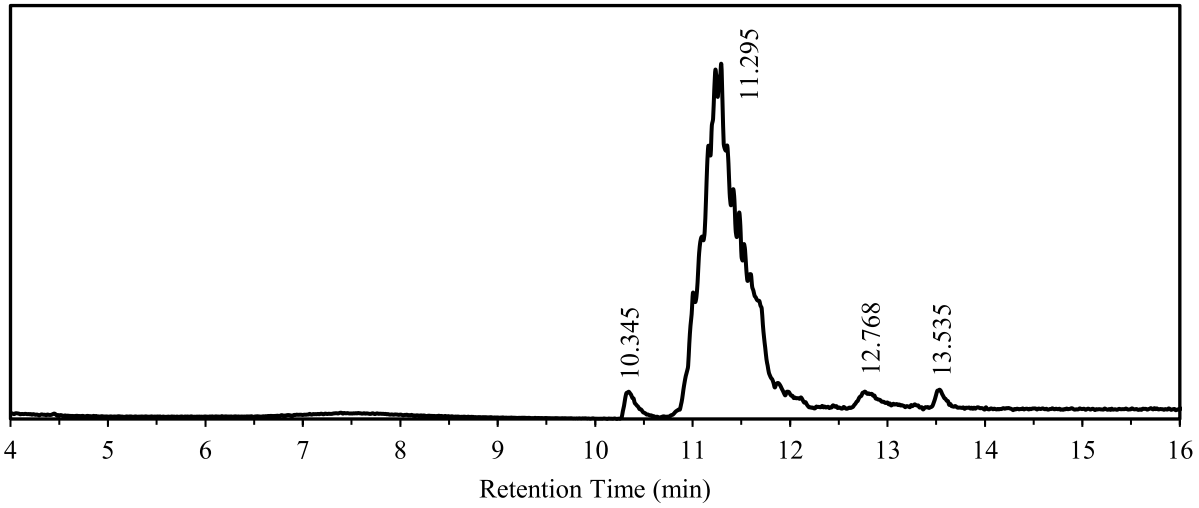

Figure S5. GC chromatogram of the reaction between **L**Cu^{III}OH (generated from [TBA]**L**Cu^{II}OH and [NAr_3]PF_6 at −35 °C) and 0.5 equivalents of Gomberg's dimer. The peak at 13.535 matches the standard of Ph_3OH used to generate the calibration curve.

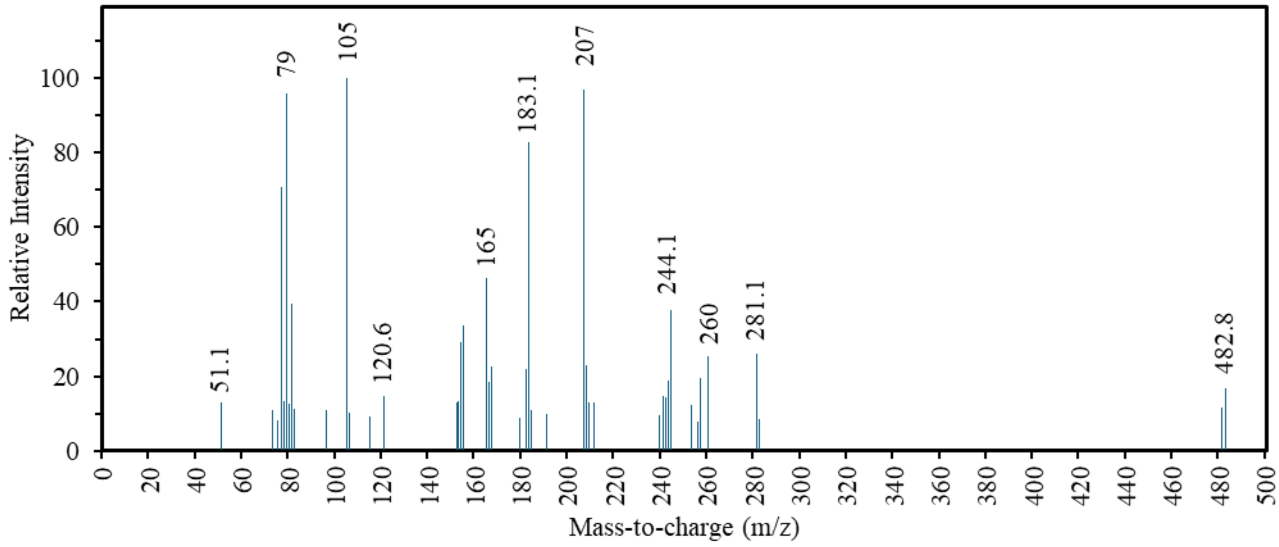

Figure S6. Mass-spectrometry of the peak at 13.535 minutes in the chromatogram of the reaction between **L**Cu^{III}OH (generated from [TBA]**L**Cu^{II}OH and [NAr_3]PF_6 at −35 °C) and 0.5 equivalents of Gomberg's dimer.

Electrochemical Measurements

Cyclic voltammograms were recorded under a nitrogen atmosphere with a Biologic SP-150 potentiostat using a three-electrode system comprised of a glassy carbon working electrode, platinum wire counter electrode, and nonaqueous silver nitrate reference electrode. All cyclic voltammograms were recorded at room temperature with a scan rate of 100 mV/s and internally referenced to ferrocene/ferrocenium. The CV solution was prepared by dissolving the copper complex (1.0 mM) in an electrolyte solution consisting of 0.1 M electrochemical grade tetrabutylammonium perchlorate in dichloroethane.

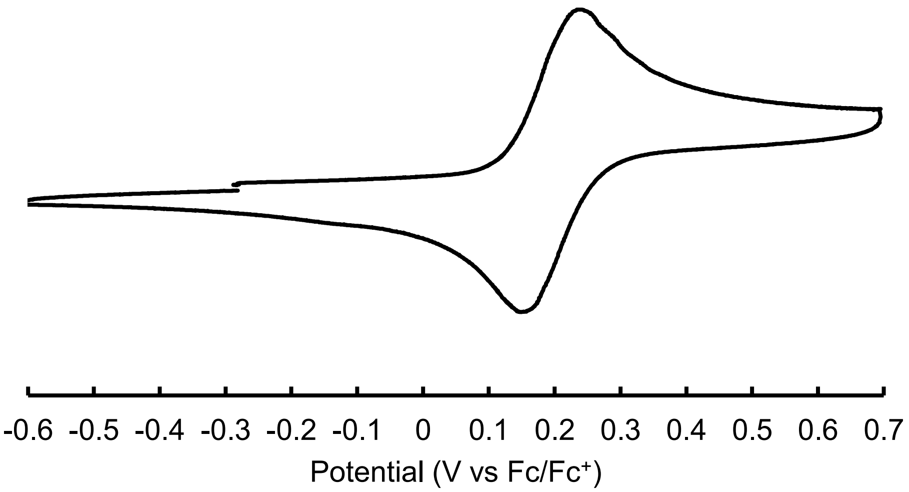

Figure S7. Cyclic voltammogram of [TBA]**L**$Cu^{II}F$ in 1,2-dichloroethane at 100 mV/s. $E_{1/2}$ = 216.4 mV vs Fc/Fc^+

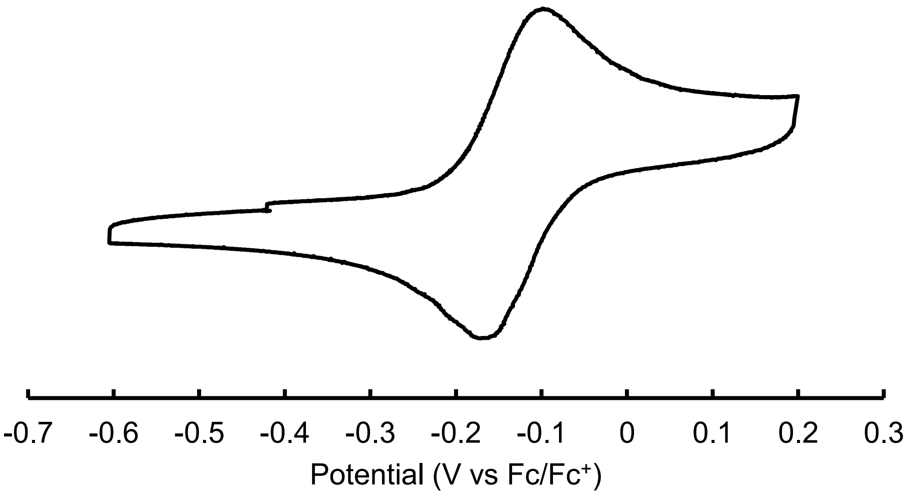

Figure S8. Cyclic voltammogram of [TBA]**L**$Cu^{II}OH$ in 1,2-dichloroethane at 100 mV/s. $E_{1/2}$ = -135.2 mV vs Fc/Fc^+

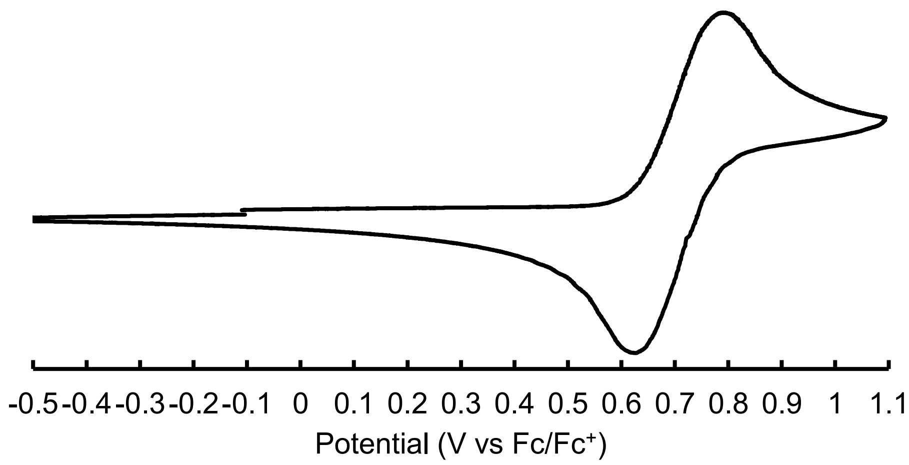

Figure S9. Cyclic voltammogram of tris(4-bromophenyl) amine in 1,2-dichloroethane at 100 mV/s. $E_{1/2}$ = 710.5 mV vs Fc/Fc$^+$

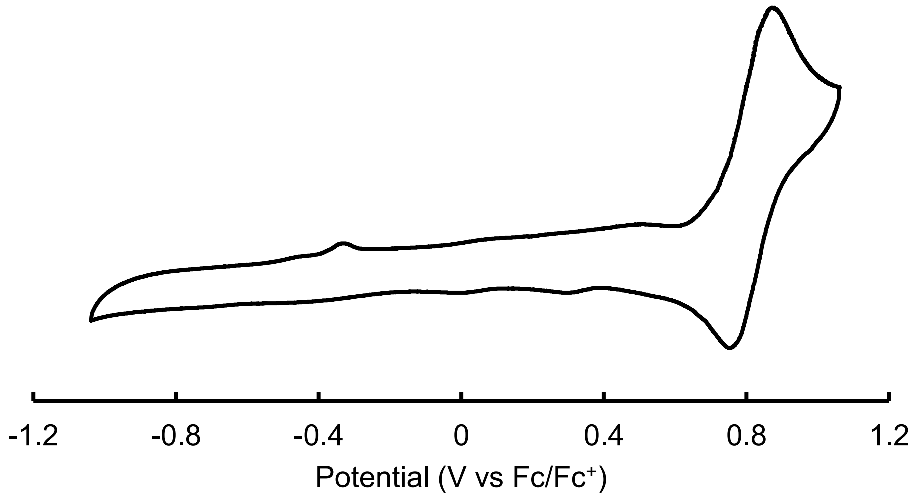

Figure S10. Cyclic voltammogram of **L**Cu^{II}OH$_2$ in 1,2-dichloroethane at 100 mV/s. $E_{1/2}$ = 815.5 mV vs Fc/Fc$^+$

Kinetic studies of HAT by LCu^{III}X

UV-vis spectra were collected on an Agilent Cary 60 spectrophotometer outfitted with a Unisoku Unispeks cryostat (−100 °C to + 100 °C). **L**Cu^{III}F or **L**Cu^{III}OH was generated in situ by the reaction of [**L**Cu^{II}X]$^-$ (X = OH or F) with one equiv. of oxidant [NAr$_3$]PF$_6$, according to the literature procedure.[1] A dichloroethane solution of [TBA]**L**Cu^{II}X (0.1 mL, 3.0 mM) was diluted to 2.800 mL in a quartz cuvette under a nitrogen atmosphere. The cuvette was sealed with a septum and cooled to −30 °C in the UV-Vis spectrometer. A dichloroethane solution of the oxidant [NAr$_3$]PF$_6$ (Ar = 4-bromophenyl) (0.1 mL, 3.0 mM, 1 equiv.) was injected into the cuvette, and the corresponding optical features of **L**Cu^{III}X were observed. Then, a solution

of the C-H substrate DHA at varying concentrations (0.1 mL, 150 – 600 mM, 50 – 200 equivalents) was injected into the cuvette by syringe. The decay of the absorption maximum of **L**Cu^{III}F at 820 nm or **L**Cu^{III}OH at 560 nm was monitored.

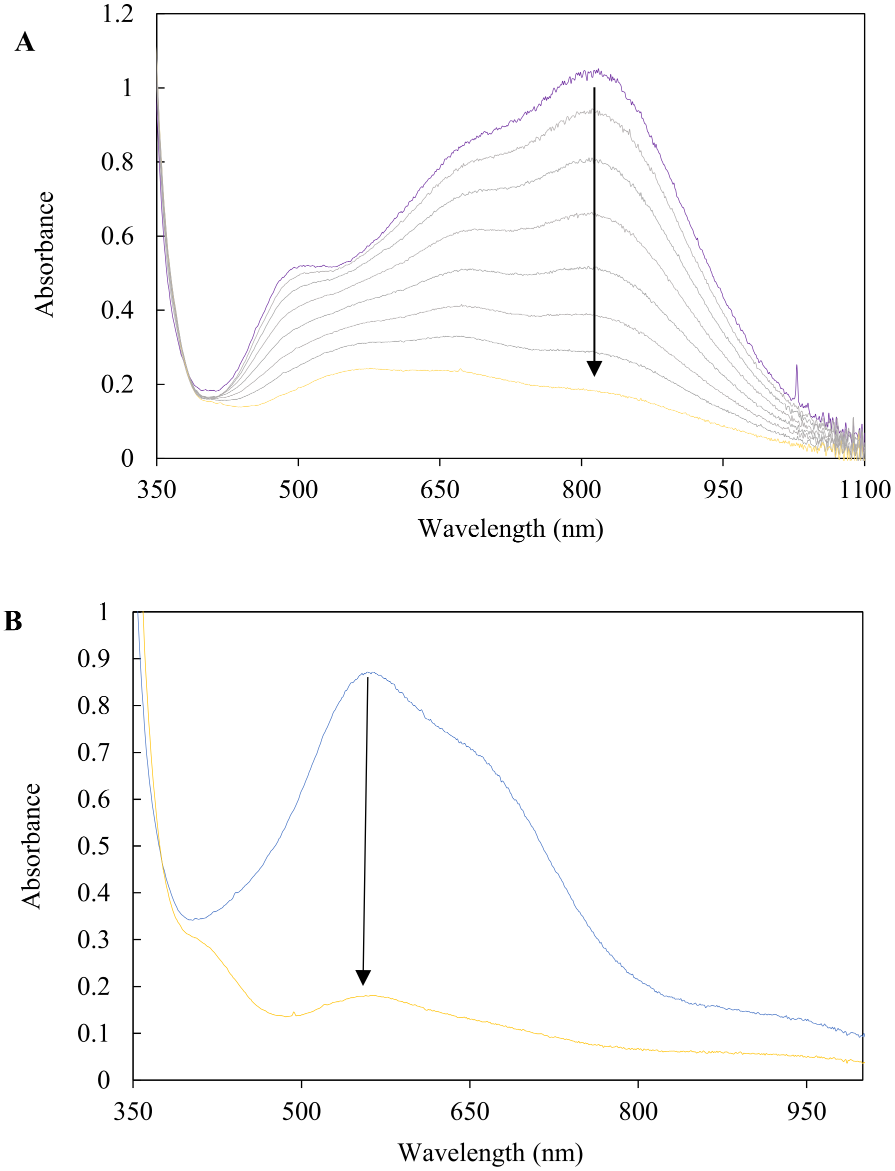

Figure S11. Representative UV-vis traces of the reaction between **L**Cu^{III}F (purple, **A**) and **L**Cu^{III}OH (blue, **B**) with an excess amount of DHA at a 30-second time interval.

Table S2. Rates (k_{obs}, s^{-1}) from the reaction of **L**Cu^{III}F with varying equivalents of 9,10-dihydroanthracene at -30 ºC in DCE. Used to plot the linear relationship between pseudo-first-order rate constant (k_{obs}, three replicates) We assume that 1 equivalent of DHA donoates 2 equivalents of H atoms, with the first HAT step being rate limiting.

[DHA]	Trial 1	Trial 2	Trial 3	Average	Std. Dev.

5 mM	0.000286	0.000239	0.000217	0.000247	0.0000288
10 mM	0.000577	0.000630	0.000946	0.000717	0.000163
15 mM	0.00132	0.00129	0.00120	0.00127	0.0000507
20 mM	0.00204	0.00181	0.00183	0.00189	0.000103

Table S3. Rates (k_{obs}, s^{-1}) from the reaction of **L**$Cu^{III}OH$ with varying equivalents of 9,10-dihydroanthracene at -30 ºC in DCE. Used to plot the linear relationship between pseudo-first-order rate constant (k_{obs}, three replicates). We assume that 1 equivalent of DHA donoates 2 equivalents of H atoms, with the first HAT step being rate limiting.

[DHA]	Trial 1	Trial 2	Trial 3	Average	Std. Dev.
5 mM	0.099	0.103	0.107	0.103	0.00268
10 mM	0.271	0.264	0.277	0.271	0.00523
15 mM	0.522	0.566	0.543	0.544	0.0177
20 mM	0.727	0.710	0.689	0.709	0.0151

Kinetic Isotope Effect of L$Cu^{III}X$

UV-vis spectra were collected on an Agilent Cary 60 spectrophotometer outfitted with a Unisoku Unispeks cryostat (−100 °C to + 100 °C). **L**$Cu^{III}F$ or **L**$Cu^{III}OH$ was generated in situ by the reaction of [**L**$Cu^{II}X]^{-}$ (X = OH or F) with one equiv. of oxidant $[NAr_3]PF_6$, according to the literature procedure.[1] A dichloroethane solution of [TBA]**L**$Cu^{II}X$ (0.1 mL, 3.0 mM) was diluted to 2.800 mL in a quartz cuvette under a nitrogen atmosphere. The cuvette was sealed with a septum and cooled to −30 °C in the UV-Vis spectrometer. A dichloroethane solution of the oxidant $[NAr_3]PF_6$ (Ar = 4-bromophenyl) (0.1 mL, 3.0 mM, 1 equiv.) was injected into the cuvette, and the corresponding optical features of **L**$Cu^{III}X$ were observed. Then, a solution of **1a** or **1a-*d*₂** (0.1 mL, 300 mM, 100 equivalents) was injected into the cuvette by syringe. The decay of the absorption maximum of **L**$Cu^{III}F$ at 820 nm or **L**$Cu^{III}OH$ at 560 nm was monitored.

Table S4. Rate constants (k_{obs}, s^{-1}) for the reaction of **L**$Cu^{III}F$ with **1a** and **1a-d_2** at 20 ºC in DCE were used to calculate the KIE of the HAT. These rate constants do not account for the subsequent radical capture reactions. The HAT rates were calculated calculated by substracting the self-decay rate of **L**$Cu^{III}F$ in DCE at 20 ºC. The calculated HAT rate for **1a** (k_{obs}, s^{-1}) is 0.000119 ± 0.0000411. Given that the HAT rate for **1a-d_2** is very close to the self-decay of **L**$Cu^{III}F$ at this temperature, a precise KIE cannot be reliably determined. However, by assuming a non-zero contribution from self-decay, we can estimate a minimum KIE of 2.95 ± 0.68.

Reaction	Trial 1	Trial 2	Trial 3	Average	Std. Dev.
**1a**	0.000165	0.000138	0.000234	0.000179	0.0000404
**1a-d_2**	0.0000584	0.0000595	0.0000642	0.0000607	0.00000252
$Cu^{III}F$ Self-Decay	0.0000491	0.0000657	0.0000646	0.0000597	0.00000762

Table S5. Rate constants (k_{obs}, s^{-1}) for the reaction of **L**$Cu^{III}OH$ with **1a** and **1a-d_2** at 0 ºC in DCE were used to calculate the KIE of the HAT. The HAT rates were calculated by subtracting the self-decay rate of

LCu^{III}F in DCE at 0 ºC. The calculated HAT rate for **1a** (k_{obs}, s^{-1}) is 0.000151 ± 0.0000159, and for **1a-*d*$_2$** (k_{obs}, s^{-1}), it is 0.0000135 ± 0.00000165, giving a KIE of 11.8 ± 1.8.

Reaction	Trial 1	Trial 2	Trial 3	Average	Std. Dev.
**1a**	0.000148	0.000156	0.000185	0.000163	0.0000159
**1a-*d*$_2$**	0.0000250	0.0000243	0.0000279	0.0000257	0.00000156
Cu^{III}OH Self-Decay	0.0000121	0.0000116	0.0000129	0.0000122	0.000000535

Procedure for quantification of anthracene produced from oxidation of DHA with LCu^{III}X

A dichloroethane solution of [TBA]**L**Cu^{II}F (0.1 mL, 3.0 mM) was diluted to 2.800 mL in a quartz cuvette under a nitrogen atmosphere. The cuvette was sealed with a septum and cooled to −30 °C in the UV-Vis spectrometer. A dichloroethane solution of the oxidant $[NAr_3]PF_6$ (Ar = 4-bromophenyl) (0.1 mL, 3.0 mM, 1 equiv.) was injected into the cuvette, and the corresponding optical features of **L**Cu^{III}F were observed. Then, a solution of the C-H substrate DHA (0.1 mL, 30 mM, 10 equivalents) was injected into the cuvette by syringe. The decay of the absorption maximum of **L**Cu^{III}F at 820 nm was monitored. After the reaction was completed the solution was filtered through silica prior to GC-MS analysis. A GC-MS calibration curve of anthracene was used to quantify the product.

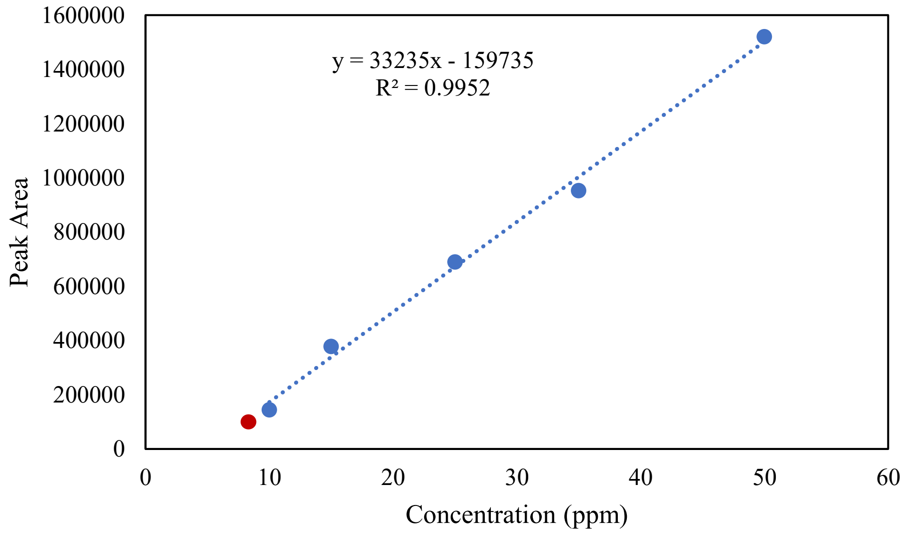

Figure S12. GC-MS calibration curve of anthracene for the analysis of HAT by **L**Cu^{III}F. Analysis of the reaction mixture of **L**Cu^{III}F with DHA shows a peak of anthracene with a peak area of 103486.2 (indicated by the red dot), giving a concentration of 7.92 ppm. This concentration suggests the yield of anthracene is 89%, considering a 2:1 **L**Cu^{III}-X to DHA stoichiometric ratio). A GCMS analysis of the DHA starting material shows that the DHA is not contaminated with anthracene. The analogous study with **L**Cu^{III}OH and anthracene was previously reported by Tolman.[2]

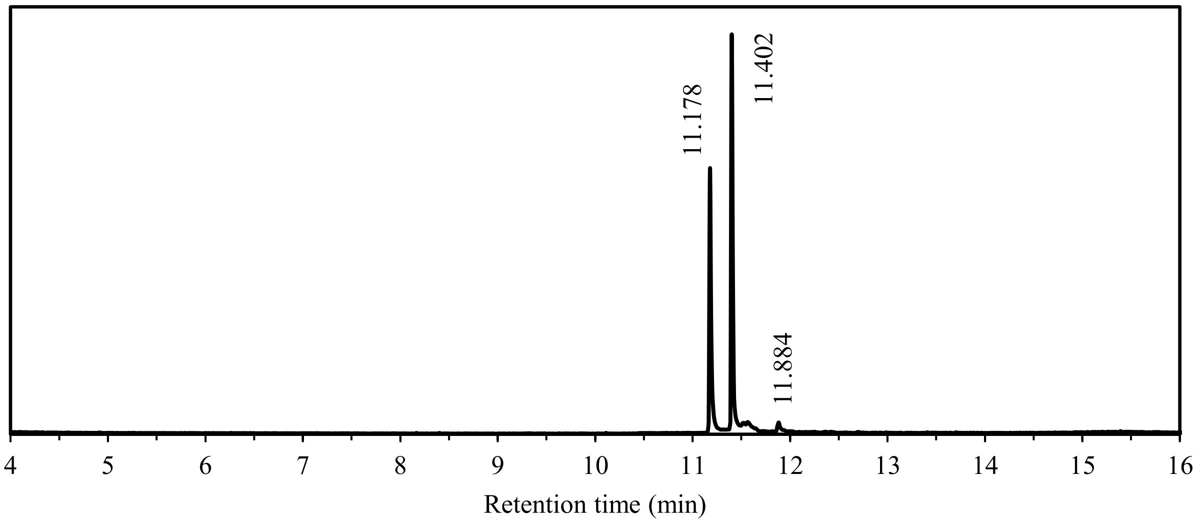

Figure S13. GC chromatogram of the reaction between **L**Cu^{III}F (generated from [TBA]**L**Cu^{II}F and [NAr$_3$]PF$_6$ at −35 °C) and DHA. The peak at 11.884 min matches the standard of anthracene. The peak at 11.178 min is benzophenone internal standard and the peak at 11.402 min is from DHA.

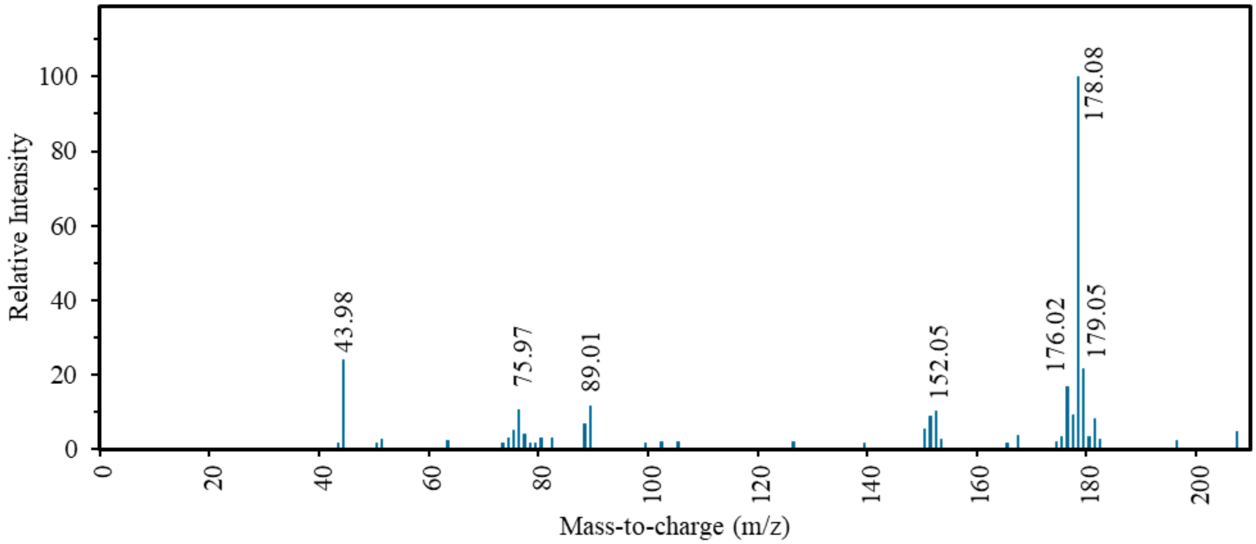

Figure S14. Mass-spectrometry of the peak at 11.884 minutes in the chromatogram corresponding to anthracene generated from the HAT reaction of **L**Cu^{III}F (generated from [TBA]**L**Cu^{II}F and [NAr$_3$]PF$_6$ at −35 °C) with DHA.

Competition HAT of LCu^{III}F vs LCu^{III}OH

UV-vis spectra were collected on an Agilent Cary 60 spectrophotometer outfitted with a Unisoku Unispeks cryostat (−100 °C to + 100 °C). **L**Cu^{III}F and **L**Cu^{III}OH were generated in situ by the reaction of [**L**Cu^{II}X]$^{-}$ (X = OH or F) with one equiv. of oxidant [NAr$_3$]PF$_6$, according to the literature procedure.[1] A dichloroethane solution of [TBA]**L**Cu^{II}F (0.1 mL, 3.0 mM) and [TBA]**L**Cu^{II}OH (0.1 mL, 3.0 mM) was diluted to 2.700 mL in a quartz cuvette under a nitrogen atmosphere. The cuvette was sealed with a septum and cooled to −30 °C in the UV-Vis spectrometer. A dichloroethane solution of the oxidant [NAr$_3$]PF$_6$ (Ar = 4-bromophenyl) (0.2 mL, 3.0 mM, 2 equiv.) was injected into the cuvette, and the corresponding optical features of **L**Cu^{III}F and **L**Cu^{III}OH were observed. Then, a solution of the C-H substrate DHA (0.1 mL, 3 mM, 1 equivalent) was injected into the cuvette by syringe. The decay of the absorption maximum of **L**Cu^{III}F at 820 nm and **L**Cu^{III}OH at 560 nm was monitored.

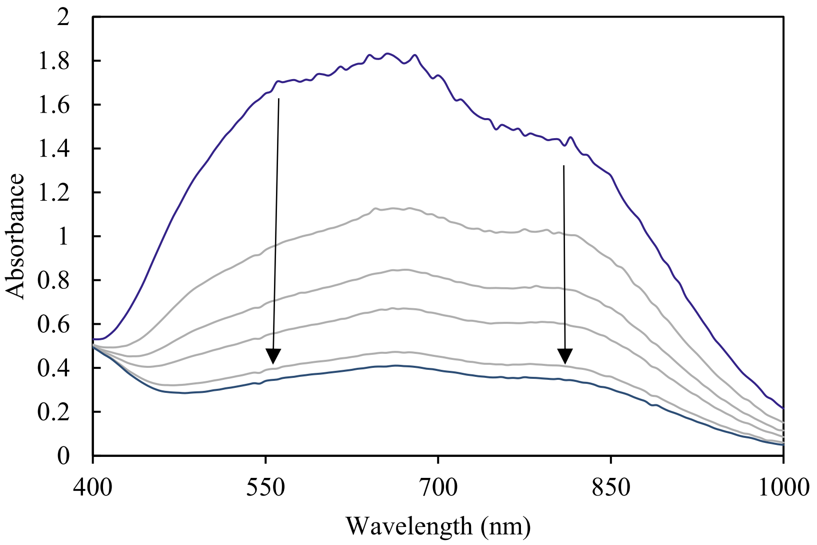

Figure S15. Representative UV-vis traces of the competition experiment between **L**Cu^{III}F and **L**Cu^{III}OH with DHA.

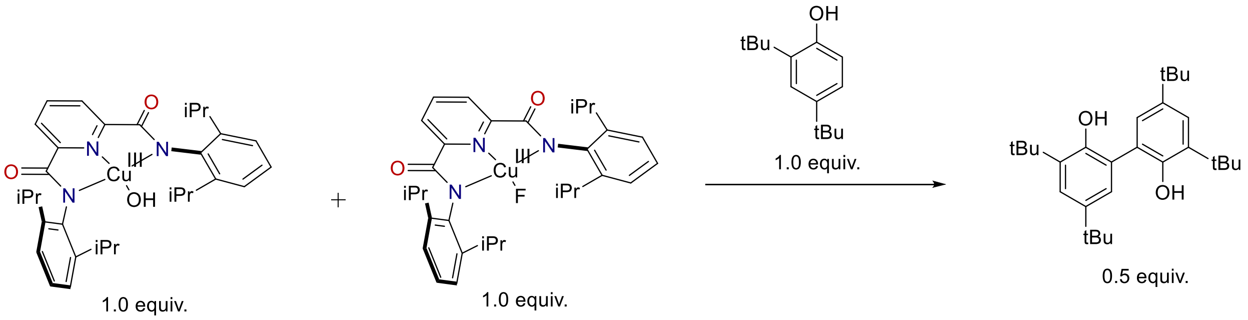

UV-vis spectra were collected on an Agilent Cary 60 spectrophotometer outfitted with a Unisoku Unispeks cryostat (−100 °C to + 100 °C). **L**Cu^{III}F and **L**Cu^{III}OH were generated in situ by the reaction of [**L**Cu^{II}X]$^{-}$ (X = OH or F) with one equiv. of oxidant [NAr$_3$]PF$_6$, according to the literature procedure.[1] A dichloroethane solution of [TBA]**L**Cu^{II}F (0.1 mL, 3.0 mM) and [TBA]**L**Cu^{II}OH (0.1 mL, 3.0 mM) was diluted to 2.700 mL in a quartz cuvette under a nitrogen atmosphere. The cuvette was sealed with a septum

and cooled to −30 °C in the UV-Vis spectrometer. A dichloroethane solution of the oxidant $[NAr_3]PF_6$ (Ar = 4-bromophenyl) (0.2 mL, 3.0 mM, 2 equiv.) was injected into the cuvette, and the corresponding optical features of **L**Cu^{III}F and **L**Cu^{III}OH were observed. Then, a solution of the model H-atom donor 2,4-di-*tert*-butylphenol (0.1 mL, 3.0 mM, 1 equivalent) was injected into the cuvette by syringe. The decay of the absorption maximum of **L**Cu^{III}F at 820 nm and **L**Cu^{III}OH at 560 nm was monitored. A deconvolution was performed using the known abs of each complex individually to solve for the amount of each complex left in the solution at various time points. GC-MS analysis of the reaction mixture after completion showed only 3,3',5,5'-tetra(*tert*-butyl)biphenyl-2,2'-diol indicating complete consumption of the starting phenol.

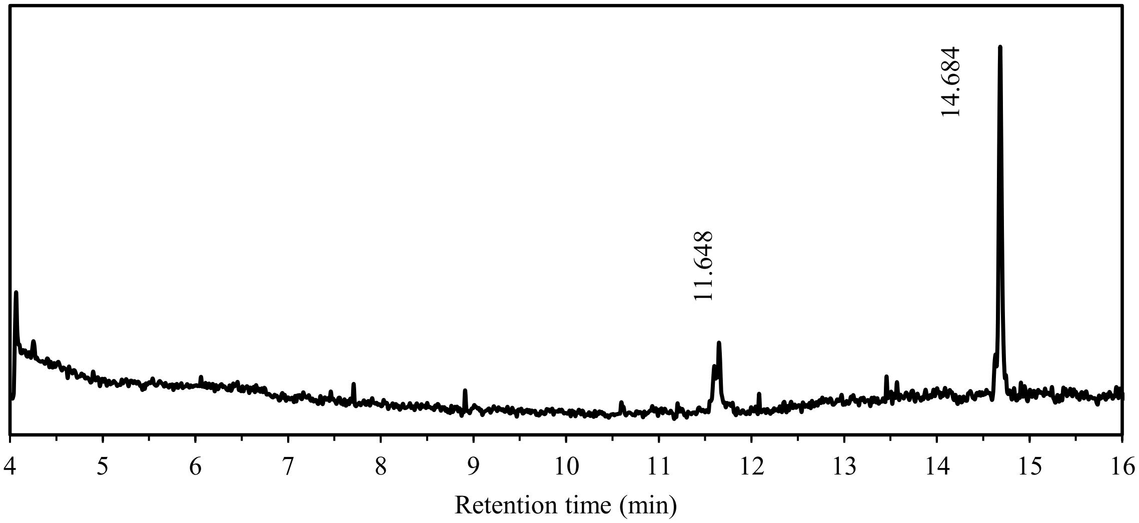

Figure S16. GC chromatogram of the competition reaction of **L**Cu^{III}F and **L**Cu^{III}OH (generated from [TBA]**L**Cu^{II}X and $[NAr_3]PF_6$ at −35 °C) and 2,4-di-*tert*-butylphenol. The peak at 14.684 mins matches the standard of 3,3',5,5'-tetra(*tert*-butyl)biphenyl-2,2'-diol. The starting material is not observed in GC-MS.

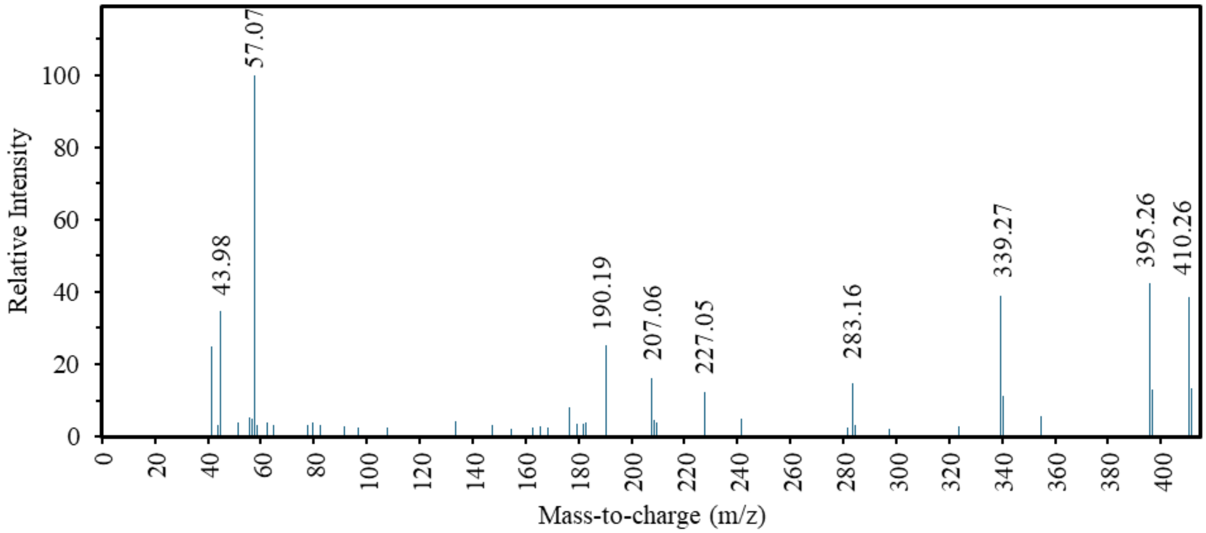

Figure S17. Mass-spectrometry of the peak at 14.684 mins in the chromatogram corresponding to 3,3',5,5'-tetra(*tert*-butyl)biphenyl-2,2'-diol generated from the competition HAT reaction of **L**Cu^{III}F and **L**Cu^{III}OH (generated from [TBA]**L**Cu^{II}X and [NAr$_3$]PF$_6$ at −35 °C) with 2,4-di-*tert*-butylphenol.

Investigating potential ligand exhange reactions between LCu^{II}OH$_2$ and LCu^{III}F

UV-vis spectra were collected on an Agilent Cary 60 spectrophotometer outfitted with a Unisoku Unispeks cryostat (−100 °C to + 100 °C). **L**Cu^{III}F and was generated in situ by the reaction of [**L**Cu^{II}F]$^{-}$ with one equiv. of oxidant [NAr$_3$]PF$_6$, according to the literature procedure.[1] A dichloroethane solution of [TBA]**L**Cu^{II}F (0.1 mL, 3.0 mM) was diluted to 2.800 mL in a quartz cuvette under a nitrogen atmosphere. The cuvette was sealed with a septum and cooled to −30 °C in the UV-Vis spectrometer. A dichloroethane solution of the oxidant [NAr$_3$]PF$_6$ (Ar = 4-bromophenyl) (0.2 mL, 3.0 mM, 2 equiv.) was injected into the cuvette, and the corresponding optical features of **L**Cu^{III}F were observed. Then, a solution of the **L**Cu^{II}OH$_2$ (0.1 mL, 3.0 mM, 1 equivalent) was injected into the cuvette by syringe.

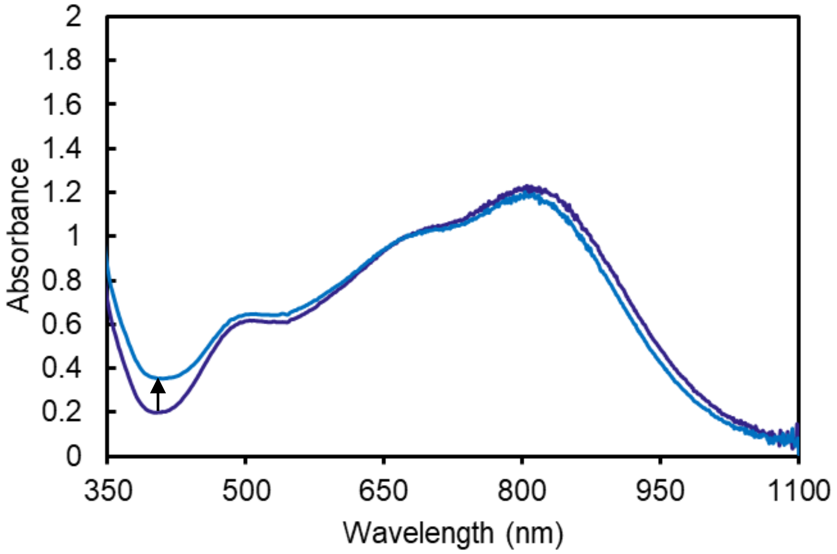

Figure S18. UV-vis traces of a mixture of **L**Cu^{III}F and **L**Cu^{II}OH$_2$. The purple trace corresponds to **L**Cu^{III}F, addition of **L**Cu^{II}OH$_2$ only causes a minor change in the spectra, resulting in the blue trace. The optical features of **L**Cu^{III}F are unchanged, indicating that there is no reaction between the two species.

Stability of 2d at various pH values

2d (3 mg, 0.018 mmol) and hexafluorobenzene (5 uL, 0.043 mmol) were added to a 20 mL scintillation vial and then dissolved in DMSO (50 uL). Then water (600 uL) at the desired pH (5, 7 or 9) was added to the vial along with D_2O (50 uL). The resulting solution was added to an NMR tube and was monitored using ^{19}F NMR for 1.5 hours. **2d** hydrolyzed completely in this time.

Table S6. Timepoints of the percent of 2d remaining in solution at different pH values.

	Percentage of **2d** remaining		
Time (min)	pH 5	pH 7	pH 9
0	100	100	100
15	44	45	48
30	22	20	25
45	8	9	11
60	3	3	5
75	0	1	0
90	0	0	0

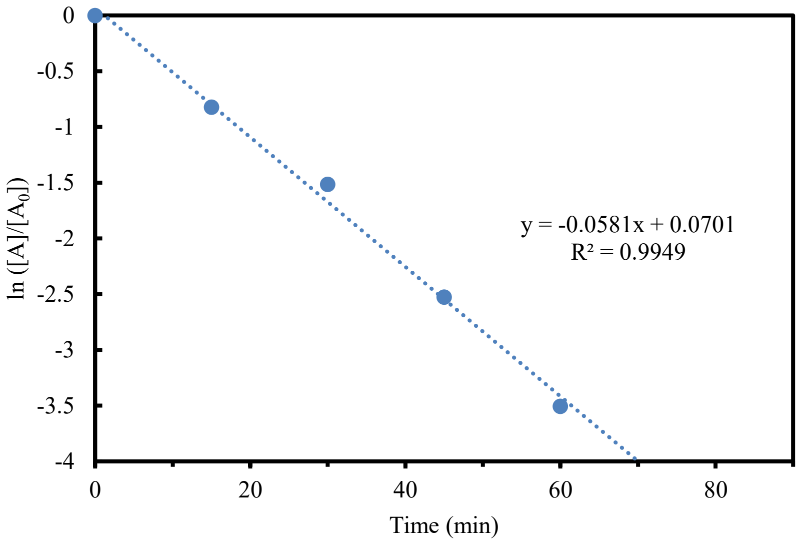

Figure S19. Pseudo-first order decay of **2d** at pH 5 obtained from ^{19}F NMR monitoring of the hydrolysis of **2d** in water at room temp. The rate of decay for **2d** (k_{obs}, min^{-1}) is 0.0581 at pH 5.

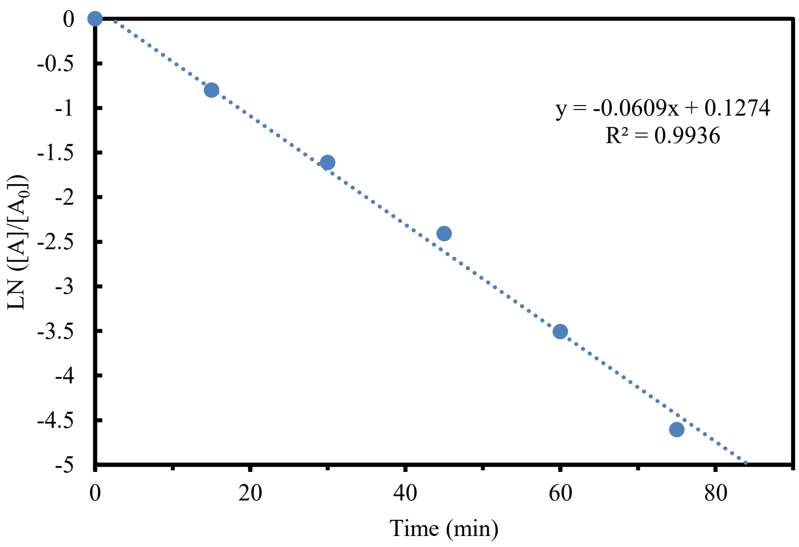

Figure S20. Pseudo-first order decay of **2d** at pH 7 obtained from ^{19}F NMR monitoring of the hydrolysis of **2d** in water at room temp. The rate of decay for **2d** (k_{obs}, min^{-1}) is 0.0609 at pH 7.

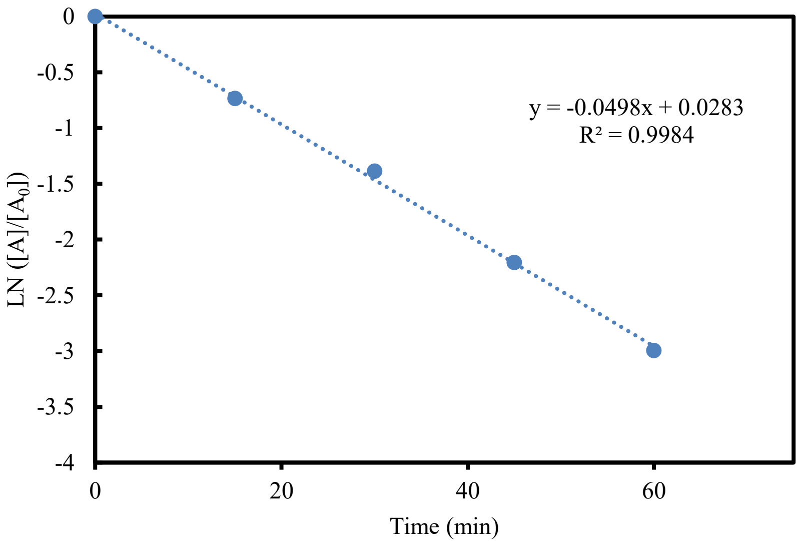

Figure S21. Pseudo-first order decay of **2d** at pH 9 obtained from ^{19}F NMR monitoring of the hydrolysis of **2d** in water at room temp. The rate of decay for **2d** (k_{obs}, min^{-1}) is 0.0498 at pH 9.

Stability of 2d in biologically relevant conditions.

2d (4 mg, 0.024 mmol) and hexafluorobenzene (5 uL, 0.043 mmol) were added to a 20 mL scintillation vial and then dissolved in DMSO (50 uL). Then Simulated Body Fluid (SBF, prepared as reported by Kokubo and Takadama[16,17]) was added to the vial along with D_2O (50 uL). The resulting solution was added to an NMR tube and an initial ^{19}F NMR timepoint was taken at room temp confirming the presence of **2d**. The NMR tube was then placed in a 37 °C oil bath and timepoints were taken at 5 minutes and 10 minutes. The ^{19}F signal corresponding to **2d** completely disappears after 5 minutes upon heating.

Stability of ^{19}F-Tadalafil in other biologically compatible solvents

To gain insight into the potential radiotracers prepared in this study, we studied the stability of the fluorinated Boc-Tadalafil **2s** in a biologically compatible solvent DMSO. ^{19}F-Boc-Tadalafil was synthesized and brought out of the glovebox. Under open air, **2s** (3 mg, 0.006 mmol) was dissolved in DMSO-d_6 (600 uL) with trifluorotoluene (0.47 mg, 0.4 µL, 0.003 mmol) as the internal standard. The resulting solution was monitored using ^{19}F NMR for 24 hours. During this time, **2s** was found to be stable in DMSO-d_6.

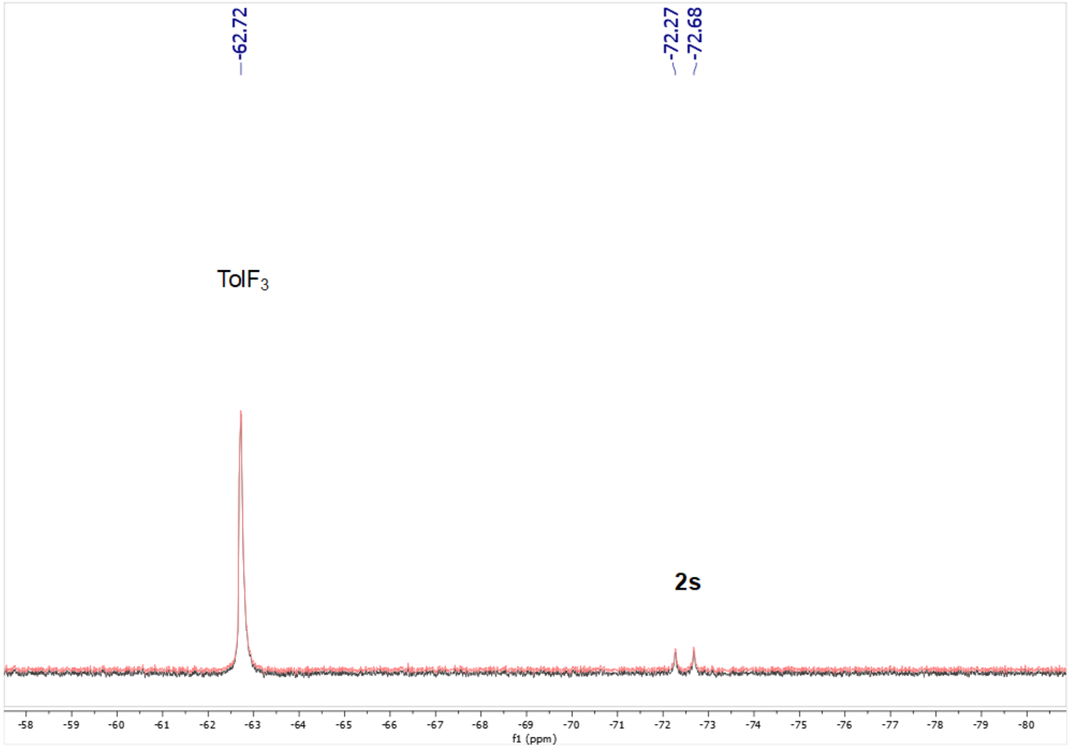

Figure S22. Overlay of NMR traces of ^{19}F-Boc-Tadalafil (**2s-^{19}F**) at the initial time point (red) and after 24 hours (black). The compound (**2s-^{19}F**) is shown to be stable for 24 hours in DMSO-d_6.

Attempt to deprotect the Boc group of 2s

2s (5 mg, 0.01 mmol, 1 equiv.) was weighed out into a 20 mL scintillation vial and dissolved in DCM (2 mL). Trifluoroacetic acid (100 uL, 1.3 mmol, 130 equiv.) was added and the reaction was stirred at 40 °C for 10 minutes. The reaction was then dried in vacuo and the resulting solids were dissoved in chloroform-d_1 for ^{19}F NMR analysis. The peaks corresponding to **2s** had completely disappeared indicating the decomposition of the compound under these conditions.

Experimental Procedures for Radiochemistry

General procedure for the synthesis of ^{18}F-radiolabeled substrates

TBAOH•30H_2O (140 mg, 0.175 mmol, 7.0 equiv.) was weighed out into a 5 mL vial and dissolved in MeCN (2 mL). A QMA-light Sep-Paks ion exchange cartridge was preconditioned by passing DI water (3 mL). Target rinse (20 mCi) was then passed through the QMA-light Sep-Pak to trap the ^{18}F-fluoride. The QMA cartridge was then rinsed with the prepared solution of TBAOH•30H_2O into a 20 mL septum cap vial

containing **L**Cu^{II}MeCN (102.9 mg, 0.175 mmol, 7.0 equiv.). The resulting solution was then dried under N_2 flow at 60 °C. The resulting solids were dissolved in dichloroethane (7.0 mL) and 1 mL of the copper solution was added to seven separate 10 mL septum cap test tubes under N_2 containing powdered 5 Å molecular sieves (40 mg), and C-H substrate (0.25 mmol, 10.0 equiv.). The test tubes were then fitted with N_2 lines and cooled to −20 °C before being stirred for 15 minutes. Then [NAr_3]SbF_6 solution in DCE (0.72 mL, 0.025 mM, 0.72 equiv.) was added to each test tube. The reactions were then stirred for 30 minutes at room temp. After completion the reaction mixtures were analyzed by radio-TLC and radio-HPLC.

General HPLC conditions

Column: Gemini-NX 5u C18 110A, 250 x 4.6 mm

Solvent A: H_2O, 0.1% TFA   Solvent B: MeCN

Gradient:

Time [min]	A[%]	B[%]	Flow [mL/min]	Max. Pressure Limit [bar]
0.00	60.0	40.0	1.000	400.00
5.00	60.0	40.0	1.000	400.00
5.50	50.0	50.0	1.000	400.00
8.50	50.0	50.0	1.000	400.00
8.75	5.0	95.0	1.000	400.00
10.00	5.0	95.0	1.000	400.00
18.00	60.0	40.0	1.000	400.00

Flow rate: 1.000 mL/min

Procedure for the isolation of ^{18}F-radiolabeled product

TBAOH•30H_2O (140 mg, 0.175 mmol, 7.0 equiv.) was weighed out into a 5 mL vial and dissolved in MeCN (2 mL). A QMA-light Sep-Paks ion exchange cartridge was preconditioned by passing DI water (3 mL). Target rinse (20 mCi) was then passed through the QMA-light Sep-Pak to trap the ^{18}F-fluoride. The QMA cartridge was then rinsed with the prepared solution of TBAOH•30H_2O into a 20 mL septum cap vial containing **L**Cu^{II}MeCN (102.9 mg, 0.175 mmol, 7.0 equiv.). The resulting solution was then dried under N_2 flow at 60 °C. The resulting solids were dissolved in dichloroethane (7.0 mL) and 1 mL of the copper solution was added to a 10 mL septum cap test tube under N_2 containing powdered 5 Å molecular sieves (40 mg), and C-H substrate (0.025 mmol, 1.0 equiv.). The test tubes were then fitted with N_2 lines and cooled to −20 °C before being stirred for 15 minutes. Then [NAr_3]SbF_6 solution in DCE (0.72 mL, 0.025 mM, 0.72 equiv.) was added to each test tube. The reactions were then stirred for 30 minutes at room temp. After completion the reaction mixtures were analyzed by radio-TLC and radio-HPLC.

After radio-TLC analysis, the reaction mixture was filtered through a frit and then a PTFE disk to remove the molecular sieves. The filtrate was then dried and redissolved in MeCN (1 mL) before being loaded to preparative HPLC for purification. Analytical HPLC was subsequently performed on the isolated product. The product [^{18}F]**2d** was obtained in 14.5% decay-corrected radiochemical yield (RCY) with a molar activity of 1.22 ± 0.42 GBq/µmol. This molar activity lets us estimate the eluted solution generally contains a mixture of $^{18}F^-$ and $^{19}F^-$ in a ratio of approximately 1:2000, meaning the total concentration of fluoride

anions ($^{18}F^{-}$ + $^{19}F^{-}$) is about 2 nmol. Our standard reaction conditions include 0.25 mmol of TBAOH. Assuming complete elution and participation of all fluoride ions, the resulting OH^{-} to F^{-} ratio in the reaction mixture is approximately 10^5:1.

To avoid saturating the prep-HPLC with C-H starting material the substrate loading was dropped from 10 equivalents to 1 equivalent.

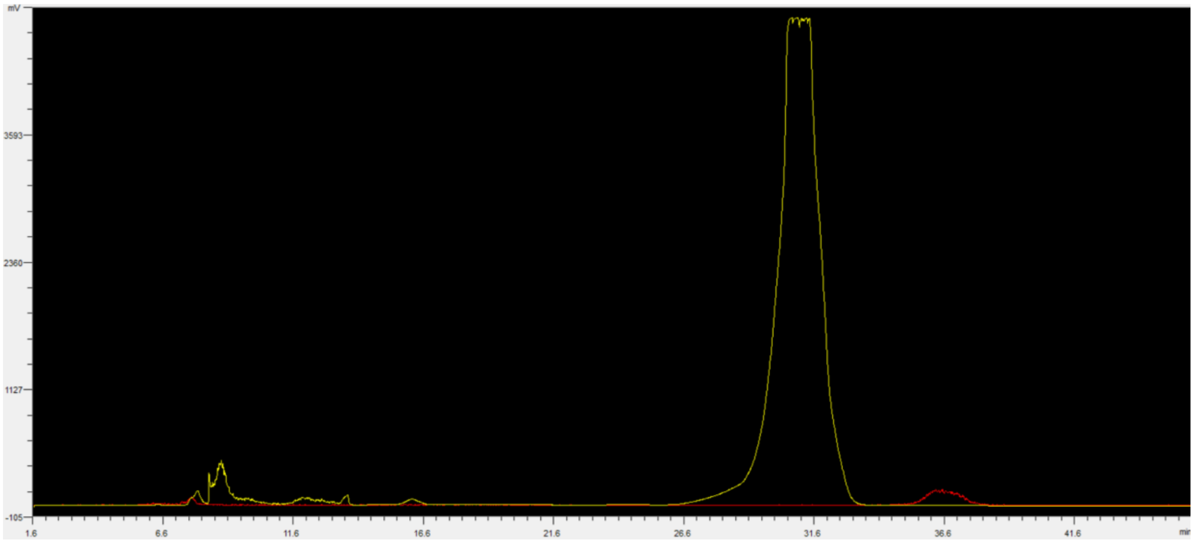

Figure S23. Representative HPLC chromatogram of the isolation of [^{18}F]**2d** from the preparative HPLC. The yellow trace corresponds to the UV detector, while the red trace corresponds to the radio detector.

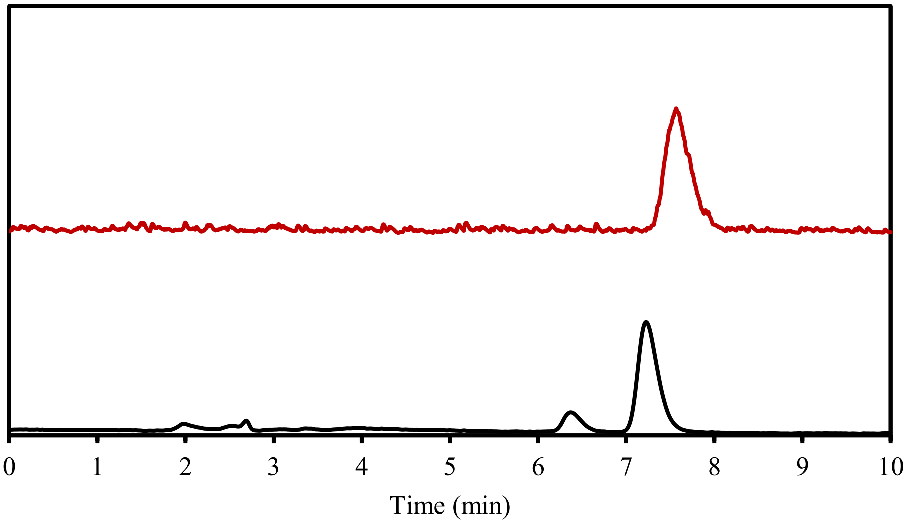

Figure S24. HPLC chromatogram of the isolation of [^{18}F]**2d** from the analytical HPLC. The black trace corresponds to the UV detector, while the red trace corresponds to the radio detector. In the UV, the peak at 6.4 mins corresponds to the starting material **1d** while the peak at 7.3 mins corresponds to the product [^{18}F]**2d.** We note that complete separation of the non-labeled starting material **1d** from the [^{18}F]**2d** remains challenging, which is a known issue in ^{18}F radio-C–H fluorination chemistry. As a result, the isolated sample contains some unlabeled starting material. Nevertheless, the radiochemical purity of the HPLC-purified product is greater than 99%, as indicated by the radio detector trace (red).

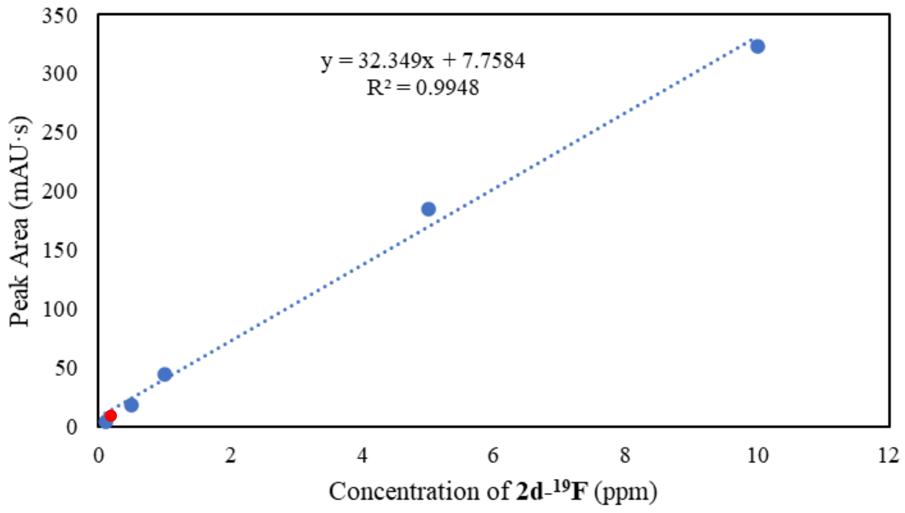

Figure S25. HPLC calibration curve of **2d** standard for the analysis of radiochemical isolation. The peak area measured for the isolation was 2.61 indicated by the red dot. The tracer amount used was 0.000487 GBq / mL for a 25 mL sample.

Preparative HPLC conditions

Column: Agilent Zorbax SB-C18, 5 um, 9.4 x 250 mm

Solvent A: H_2O (75%) Solvent B: MeCN (25%)Flow rate: 1.000 mL/min

Analytical HPLC conditions

Column: Gemini-NX 5u C18 110A, 250 x 4.6 mm

Solvent A: H_2O (60%) Solvent B: MeCN (40%)

Flow rate: 1.000 mL/min

Radio-TLC and Radio-HPLC analysis and characterization for ^{18}F-labeled substrates

General information: All the ^{18}F-labeling C-H substrates were performed using the general procedure described above. Three to four replicated experiments were used to calculate RCCs. The radioactive HPLC peak and cold standard are offset by ca. 0.5 min due to the distance between the UV and radio detector. The red HPLC traces are the radio signal for the crude radiolabeled products. All HPLC analysis was performed using the reported standard procedure.

1-(2-(fluoro-^{18}*F*)benzo[d][1,3]dioxol-5-yl)ethan-1-one ([^{18}F]2a)

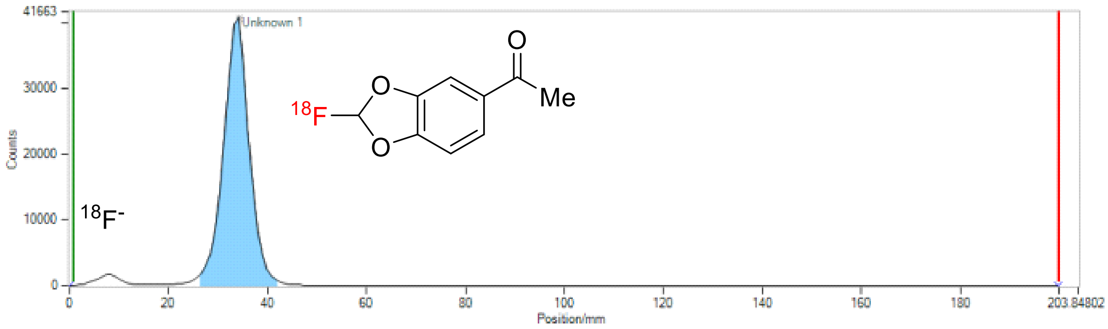

Figure S26. A representative Radio-TLC trace for the quantification of [^{18}F]**2a**.

Table S7. Average radiochemical yields for [^{18}F]**2a**.

Trial number	RCC [^{18}F]**2a**
1	93.69
2	87.15
3	91.94
4	94.98
**Average**	91.94 ± 2.97 % n = 4

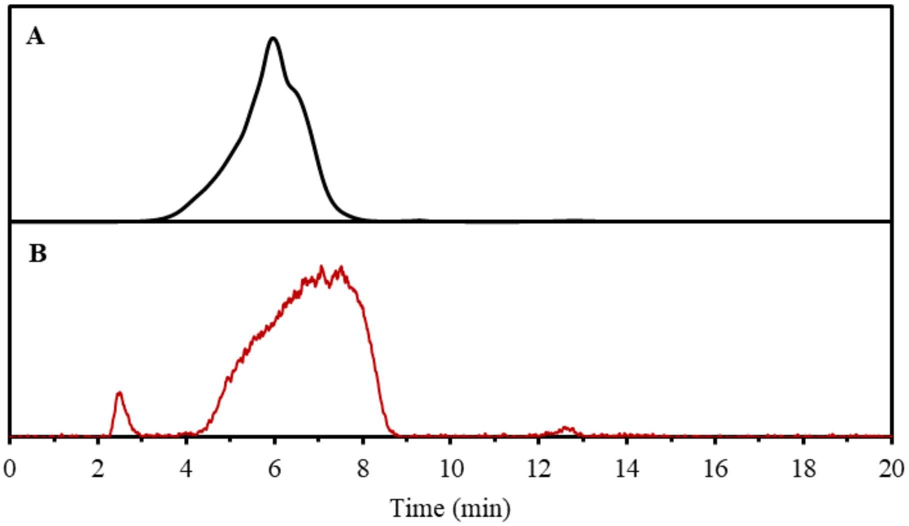

Figure S27. HPLC traces of standard (**A**) and ^{18}F-product (**B**) for **2a**

5-bromo-2-(fluoro-^{18}*F*)benzo[d][1,3]dioxole ([^{18}F]2b)

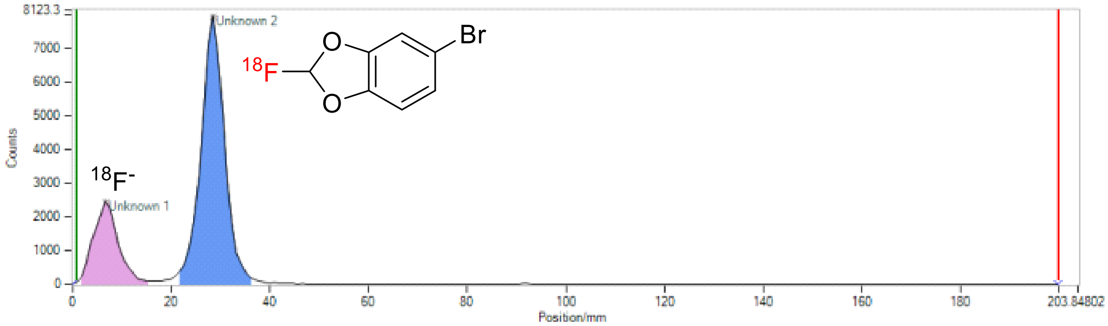

Figure S28. A representative Radio-TLC trace for the quantification of [^{18}F]**2b**.

Table S8. Average radiochemical conversions for [^{18}F]**2b**.

Trial number	RCC [^{18}F]**2b**
1	72.78
2	75.08
3	80.32
**Average**	76.06 ± 3.16 % n = 3

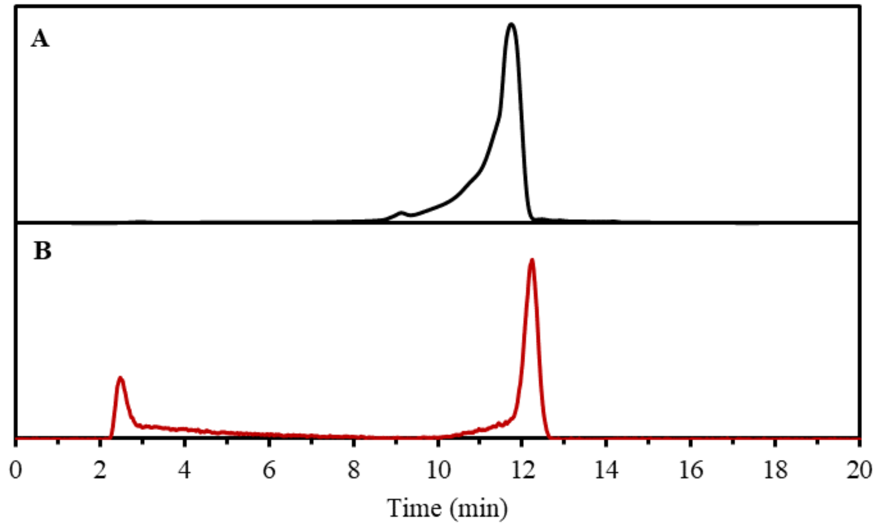

Figure S29. HPLC traces of standard (**A**) and ^{18}F-product (**B**) for **2b**.

ethyl 3-(2-(fluoro-^{18}*F*)benzo[d][1,3]dioxol-5-yl)propanoate ([^{18}F]2c)

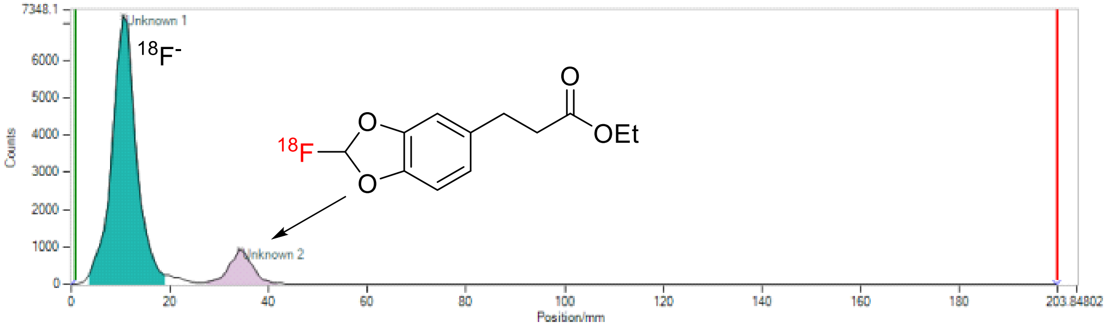

Figure S30. A representative Radio-TLC trace for the quantification of [^{18}F]**2c**.

Table S9. Average radiochemical conversions for [^{18}F]**2c**.

Trial number	RCC [^{18}F]**2c**
1	20.16
2	24.86
3	10.61
**Average**	18.54 ± 7.26 % n = 3

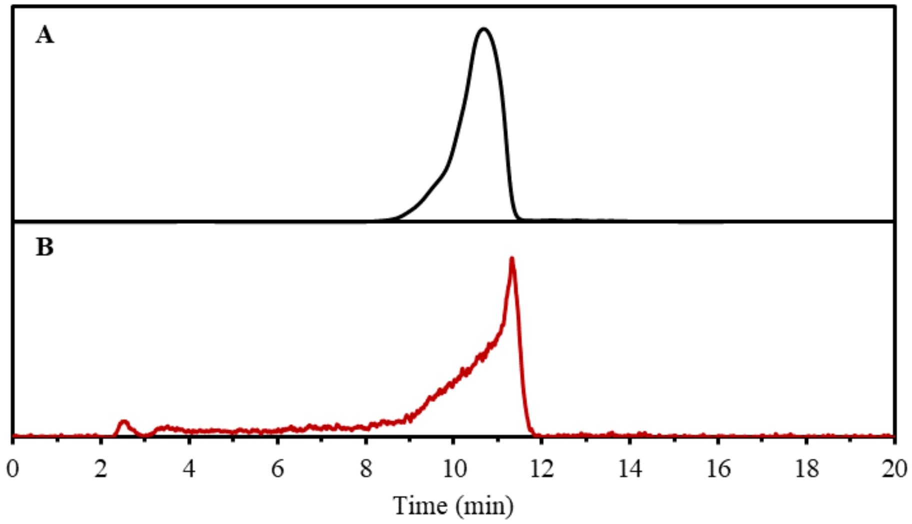

Figure S31. HPLC traces of standard (**A**) and ^{18}F-product (**B**) for **2c**.

2-(fluoro-^{18}*F*)benzo[d][1,3]dioxole-5-carbonitrile ([^{18}F]2d)

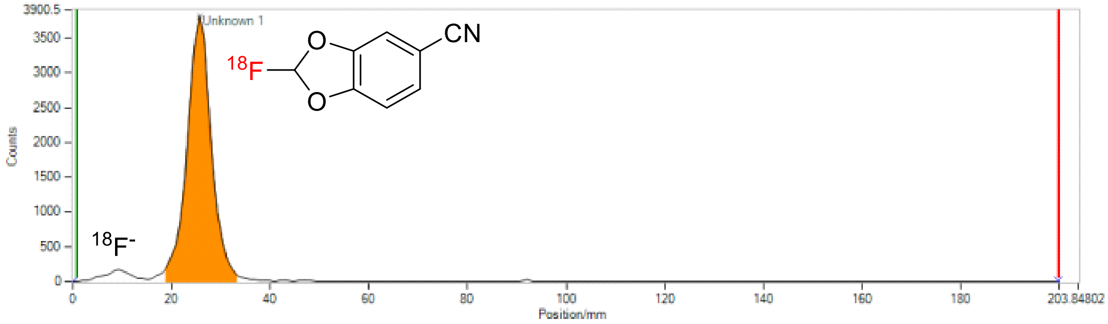

Figure S32. A representative Radio-TLC trace for the quantification of [^{18}F]**2d**.

Table S10. Average radiochemical conversions for [^{18}F]**2d**.

Trial number	RCC [^{18}F]**2d**
1	93.85
2	94.96
3	93.60
4	92.08
**Average**	93.62 ± 1.03 % n = 4

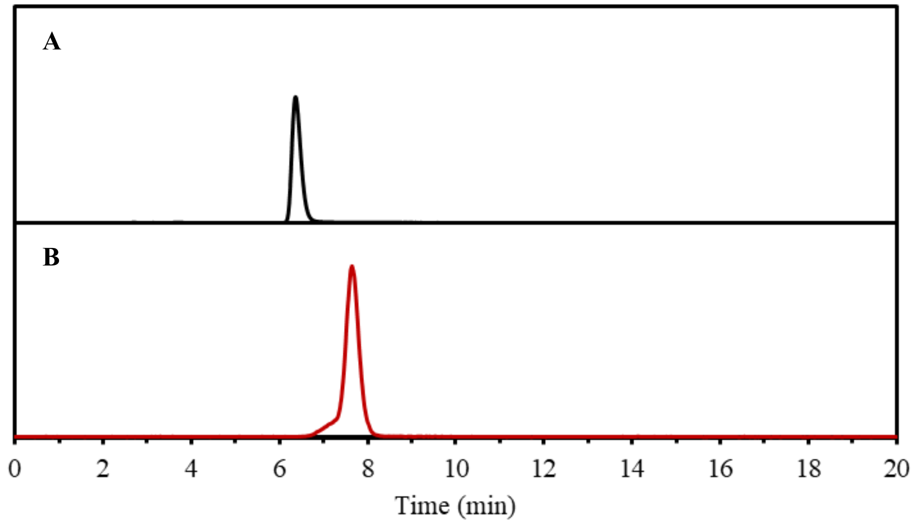

Figure S33. HPLC traces of standard (**A**) and ^{18}F-product (**B**) for **2d**.

(2-(fluoro-^{18}*F*)benzo[d][1,3]dioxol-5-yl)(phenyl)methanone ([^{18}F]2e)

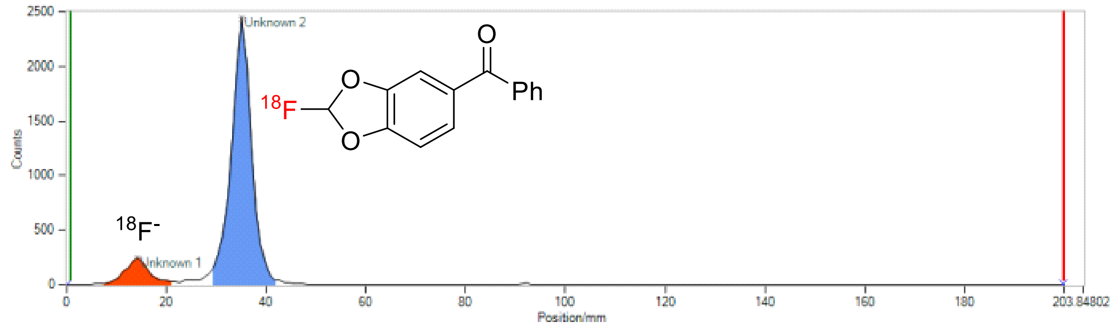

Figure S34. A representative Radio-TLC trace for the quantification of [^{18}F]**2e**.

Table S11. Average radiochemical conversions for [^{18}F]**2e**.

Trial number	RCC [^{18}F]**2e**
1	87.24
2	85.35
3	88.64
**Average**	87.08 ± 1.35 % n = 3

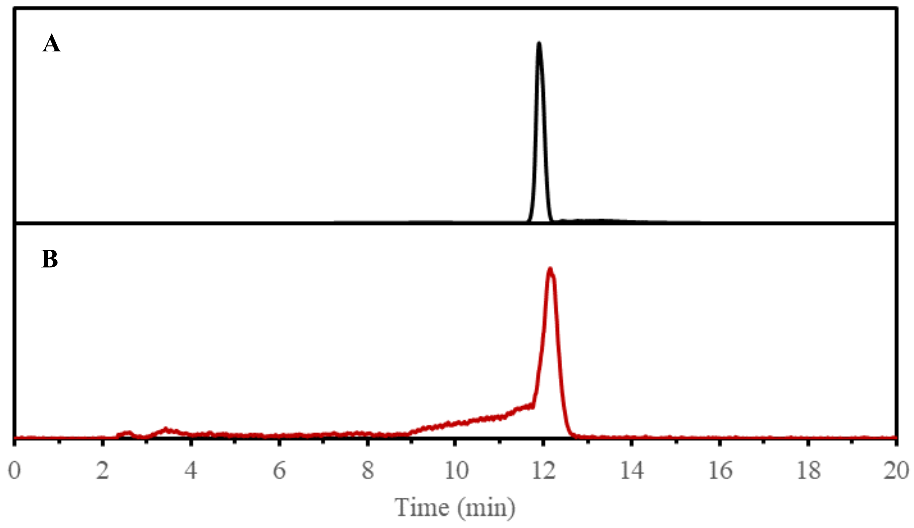

Figure S35. HPLC traces of standard (**A**) and ^{18}F-product (**B**) for **2e**.

ethyl (E)-3-(2-(fluoro-^{18}*F*)benzo[*d*][1,3]dioxol-5-yl)acrylate ([^{18}F]2f)

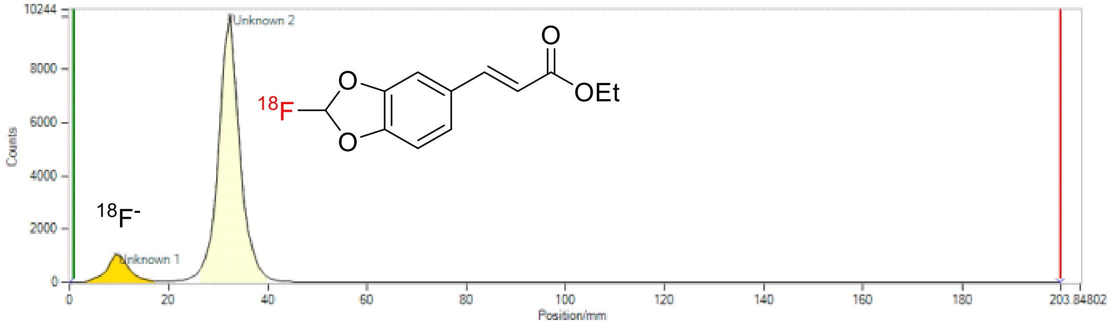

Figure S36. A representative Radio-TLC trace for the quantification of [^{18}F]**2f**.

Table S12. Average radiochemical conversions for [^{18}F]**2f**.

Trial number	RCC [^{18}F]**2f**
1	87.40
2	88.24
3	82.29
**Average**	85.98 ± 2.63 % n = 3

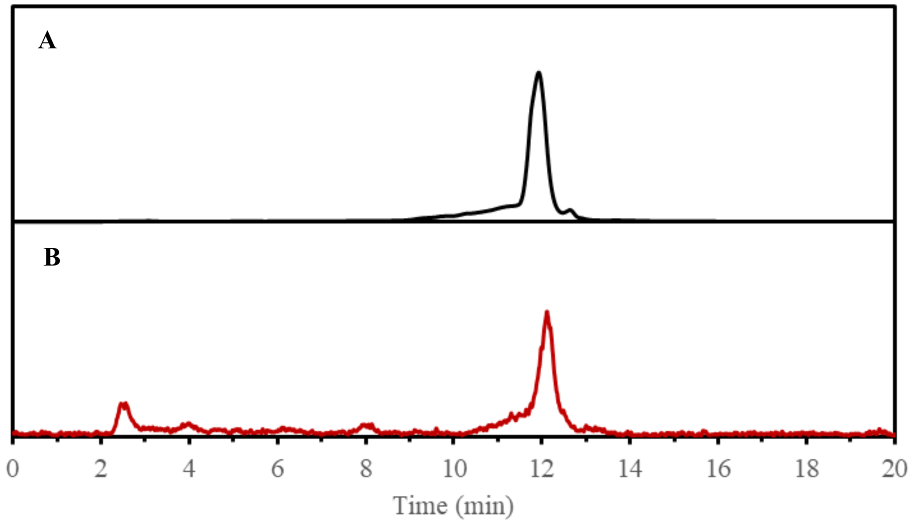

Figure S37. HPLC traces of standard (**A**) and ^{18}F-product (**B**) for **2f**.

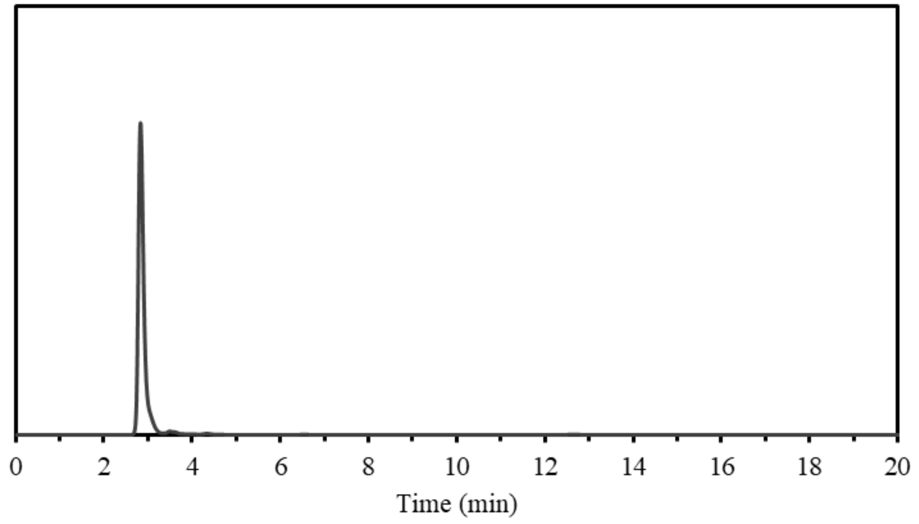

Figure S38. HPLC trace of **2f** standard using pure MeCN as the eluent.

(E)-3-(2-(fluoro-[18]*F*)benzo[*d*][1,3]dioxol-5-yl)-1-phenylprop-2-en-1-one ([^{18}F]2g)

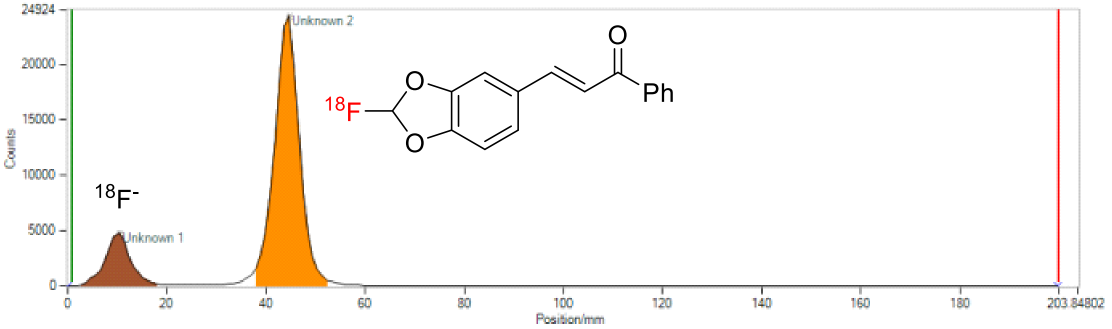

Figure S39. A representative Radio-TLC trace for the quantification of [^{18}F]**2g**.

Table S13. Average radiochemical conversions for [^{18}F]**2g**.

Trial number	RCC [^{18}F]**2g**
1	80.35
2	80.59
3	80.19
**Average**	80.38 ± 0.16 % n = 3

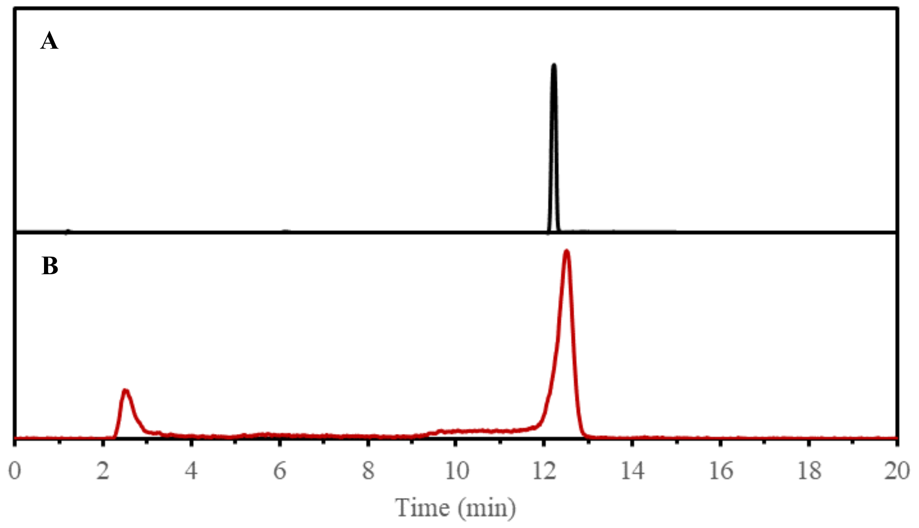

Figure S40. HPLC traces of standard (**A**) and ^{18}F-product (**B**) for **2g**.

ethyl 2-(fluoro-^{18}*F*)benzo[d][1,3]dioxole-5-carboxylate ([^{18}F]2h)

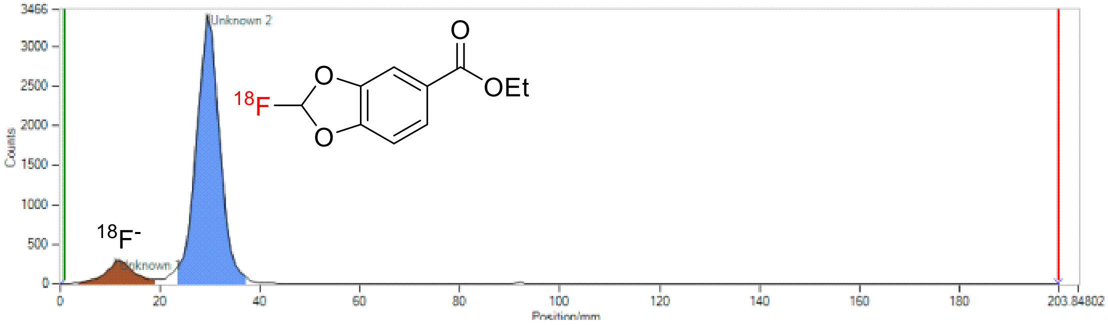

Figure S41. A representative Radio-TLC trace for the quantification of [^{18}F]**2h**.

Table S14. Average radiochemical conversions for [^{18}F]**2h**.

Trial number	RCC [^{18}F]**2h**
1	87.21
2	88.44
3	82.63
**Average**	86.09 ± 2.50 % n = 3

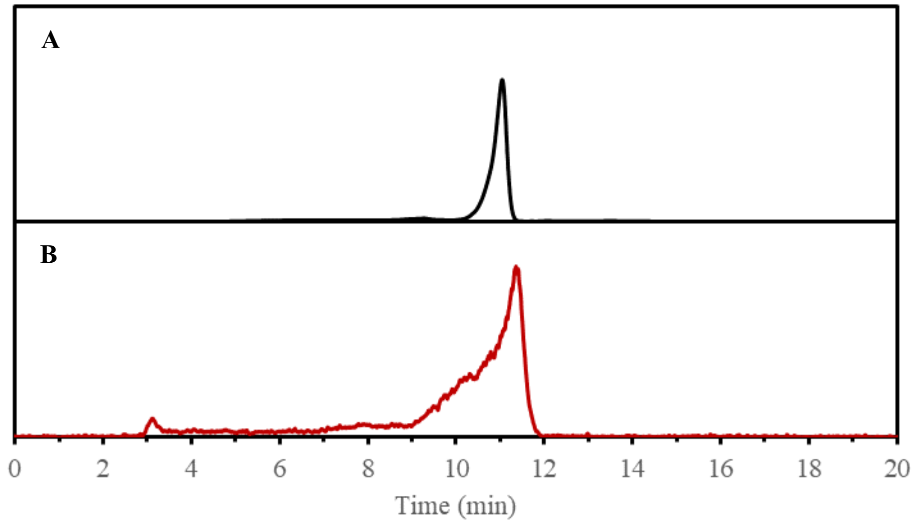

Figure S42. HPLC traces of standard (**A**) and ^{18}F-product (**B**) for **2h**.

2-(fluoro-^{18}*F*)benzo[d][1,3]dioxol-5-yl 4-methylbenzenesulfonate ([^{18}F]2i)

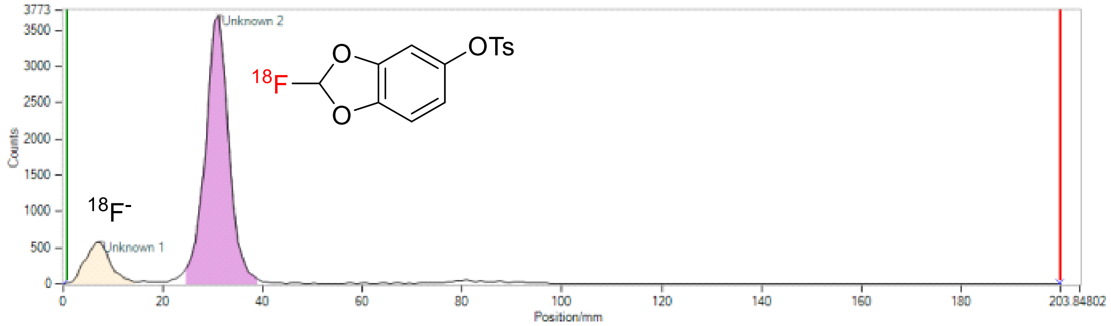

Figure S43. A representative Radio-TLC trace for the quantification of [^{18}F]**2i**.

Table S15. Average radiochemical conversions for [^{18}F]**2i**.

Trial number	RCC [^{18}F]**2i**
1	80.09
2	72.02
3	91.98
**Average**	81.36 ± 8.19 % n = 3

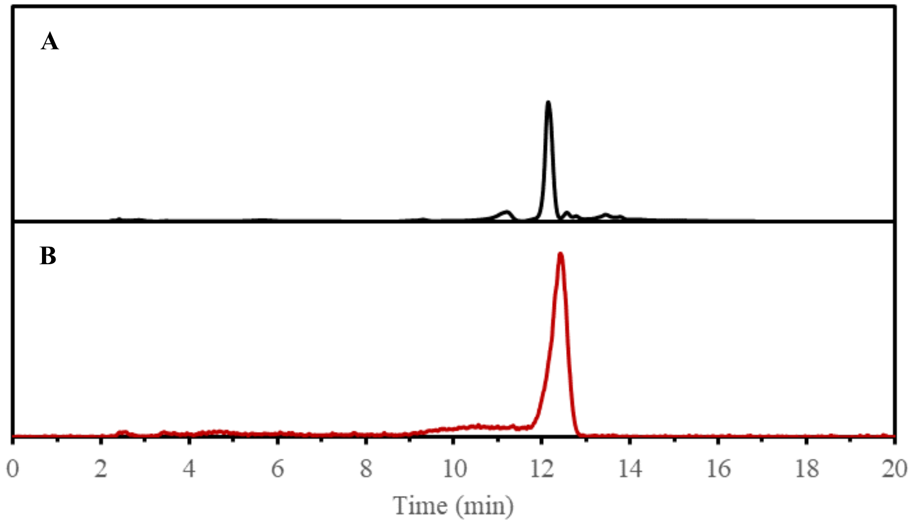

Figure S44. HPLC traces of standard (**A**) and ^{18}F-product (**B**) for **2i**.

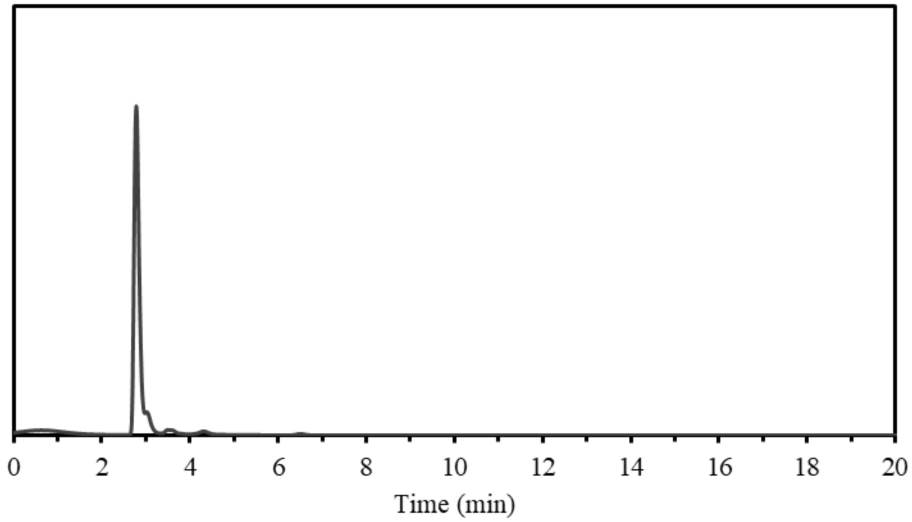

Figure S45. HPLC trace of **2i** standard using pure MeCN as the eluent.

2-((2-(fluoro-^{18}*F*)benzo[d][1,3]dioxol-5-yl)oxy)pyridine ([^{18}F]2j)

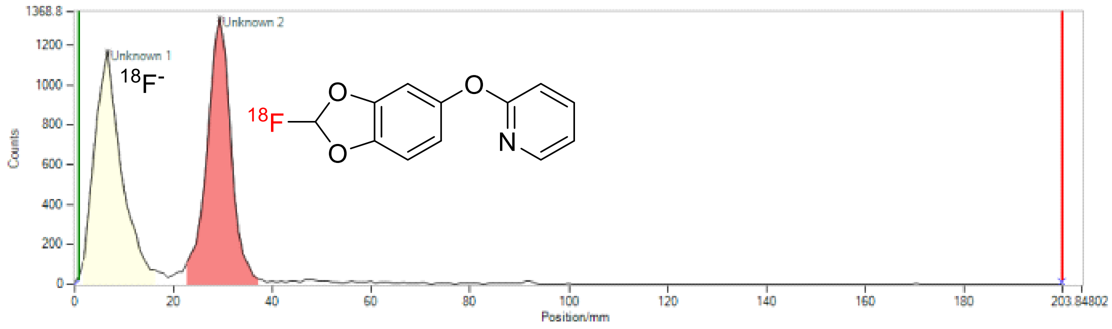

Figure S46. A representative Radio-TLC trace for the quantification of [^{18}F]**2j**.

Table S16. Average radiochemical conversions for [^{18}F]**2j**.

Trial number	RCC [^{18}F]**2j**
1	52.14
2	47.97
3	47.17
**Average**	49.09 ± 2.18 % n = 3

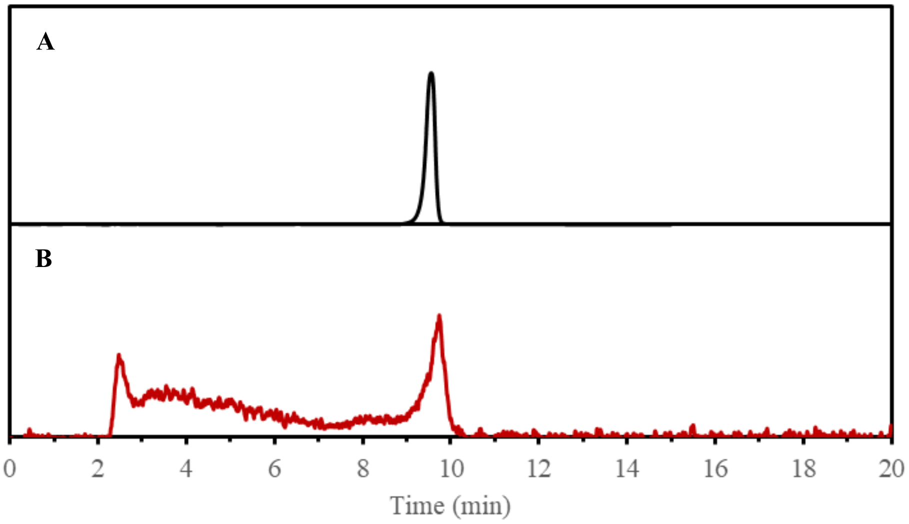

Figure S47. HPLC traces of standard (**A**) and ^{18}F-product (**B**) for **2j**.

2-(fluoro-^{18}*F*)benzo[*d*][1,3]dioxol-5-yl acetate ([^{18}F]2k)

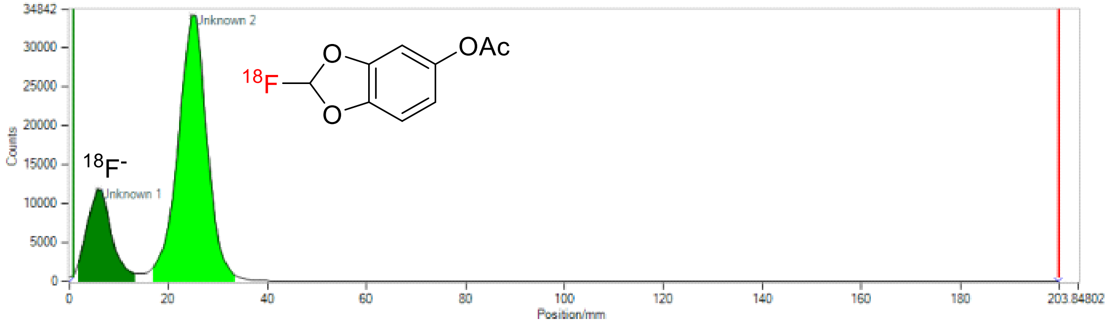

Figure S48. A representative Radio-TLC trace for the quantification of [^{18}F]**2k**.

Table S17. Average radiochemical conversions for [^{18}F]**2k**.

Trial number	RCC [^{18}F]**2k**
1	74.79
2	72.61
3	66.69
**Average**	71.36 ± 3.42 n = 3

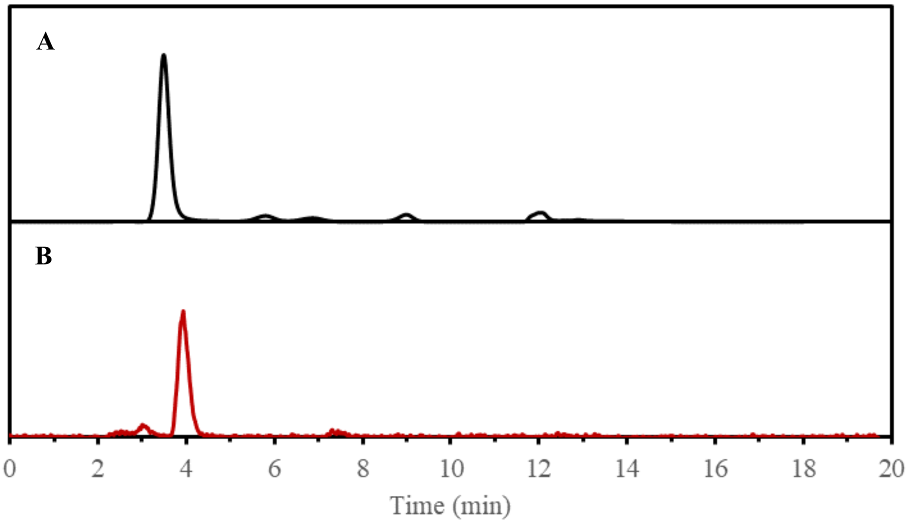

Figure S49. HPLC traces of standard (**A**) and ^{18}F-product (**B**) for **2k**.

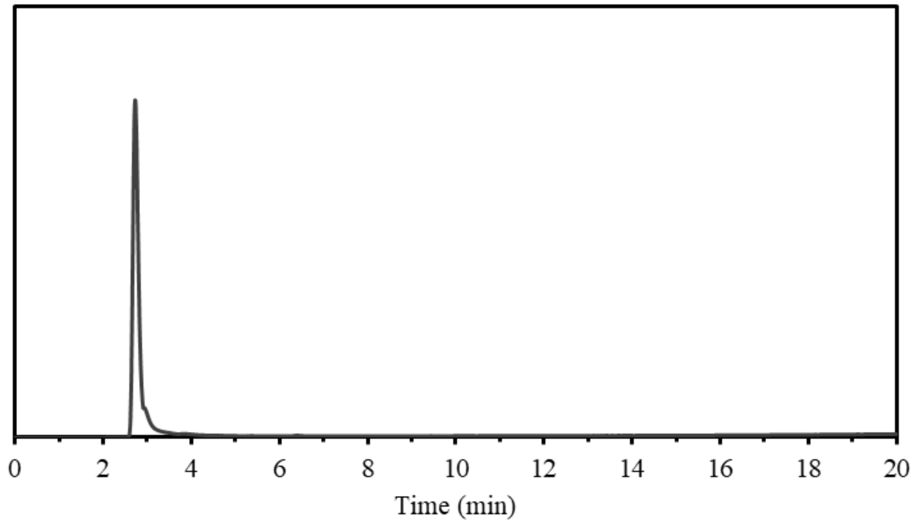

Figure S50. HPLC trace of **2k** standard using pure MeCN as the eluent.

2-(fluoro-[18]*F*)-5-nitrobenzo[d][1,3]dioxole ([18F]2l)

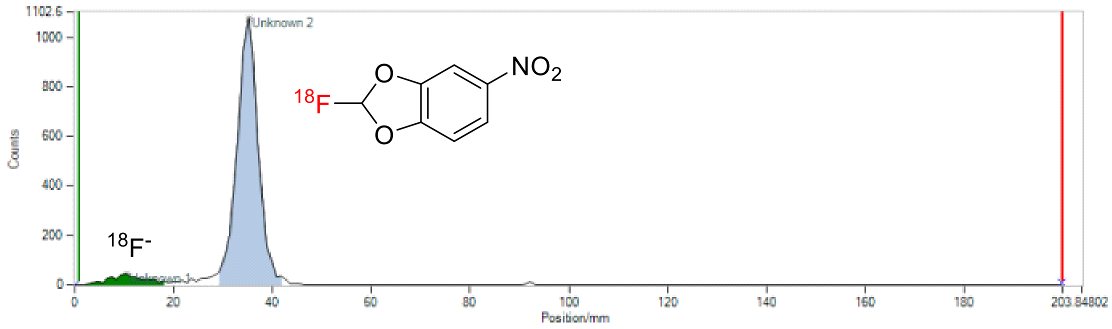

Figure S51. A representative Radio-TLC trace for the quantification of [^{18}F]**2l**.

Table S18. Average radiochemical conversions for [^{18}F]**2l**.

Trial number	RCC [^{18}F]**2l**
1	93.90
2	93.38
3	91.85
**Average**	93.04 ± 0.87 % n = 3

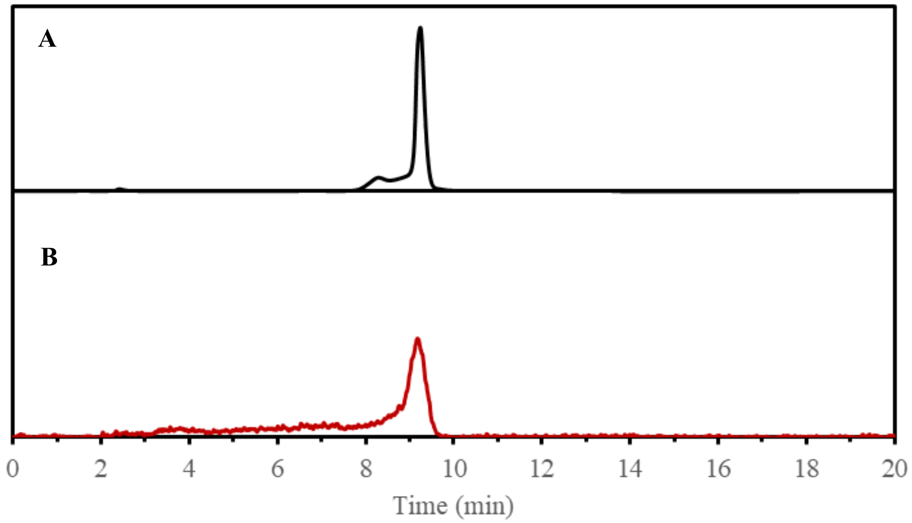

Figure S52. HPLC traces of standard (**A**) and ^{18}F-product (**B**) for **2l**.

2-(2-(fluoro-[18]*F*)benzo[*d*][1,3]dioxol-5-yl)-4,6-bis(trichloromethyl)-1,3,5-triazine ([^{18}F]2m)

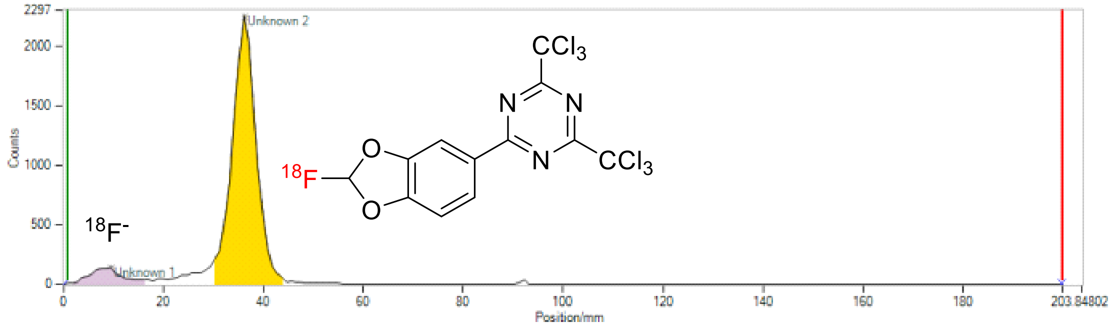

Figure S53. A5 representative Radio-TLC trace for the quantification of [^{18}F]**2m**.

Table S19. Average radiochemical conversions for [^{18}F]**2m**.

Trial number	RCC [^{18}F]**2m**
1	83.95
2	80.83
3	70.85
**Average**	78.54 ± 5.59 n = 3

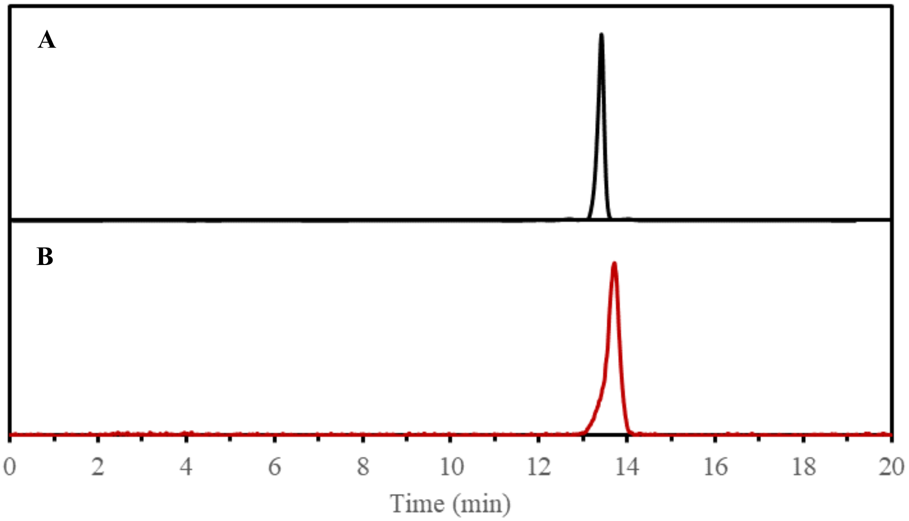

Figure S54. HPLC traces of standard (**A**) and ^{18}F-product (**B**) for **2m**.

(*E*)-1-(fluoro-^{18}*F*)-3-phenylallyl benzoate ([^{18}F]2n)

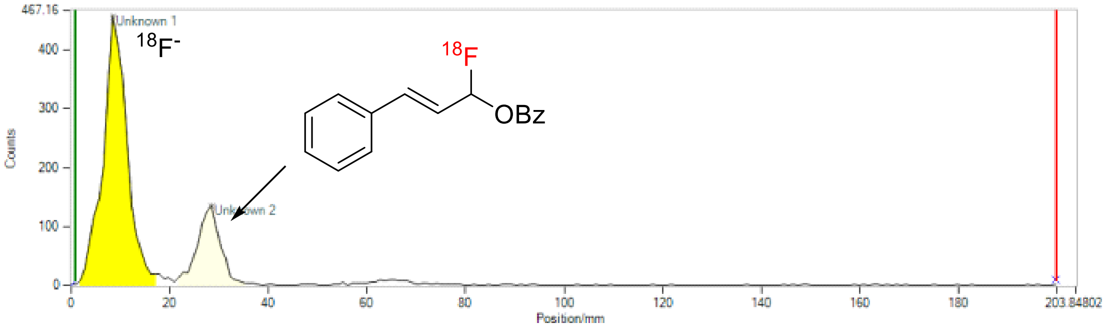

Figure S55. A representative Radio-TLC trace for the quantification of [^{18}F]**2n**.

Table S20. Average radiochemical conversions for [^{18}F]**2n**.

Trial number	RCC [^{18}F]**2n**
1	24.74
2	22.34
3	20.84
4	21.45
**Average**	22.34 ± 1.48 n = 4

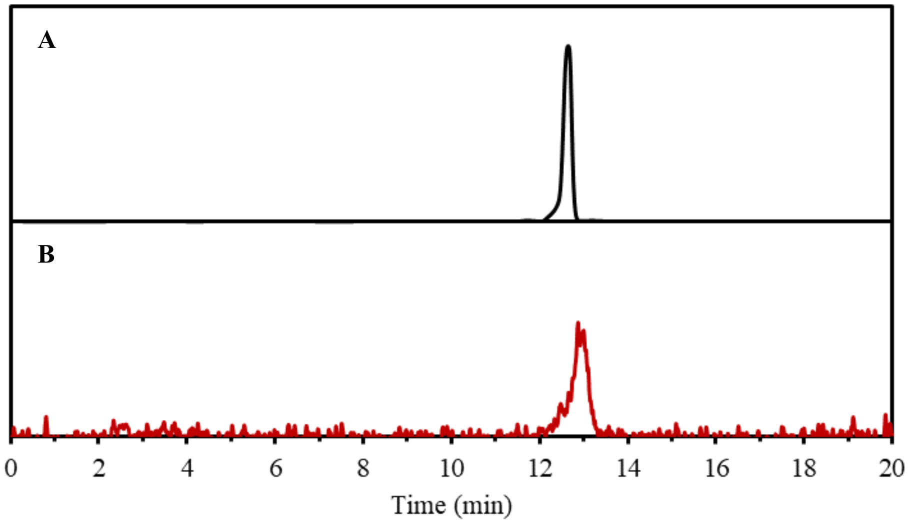

Figure S56. HPLC traces of standard (**A**) and ^{18}F-product (**B**) for **2n**.

Ethyl 3-([1,1'-biphenyl]-4-yl)-3-(fluoro-^{18}*F*)propanoate ([^{18}F]2o)

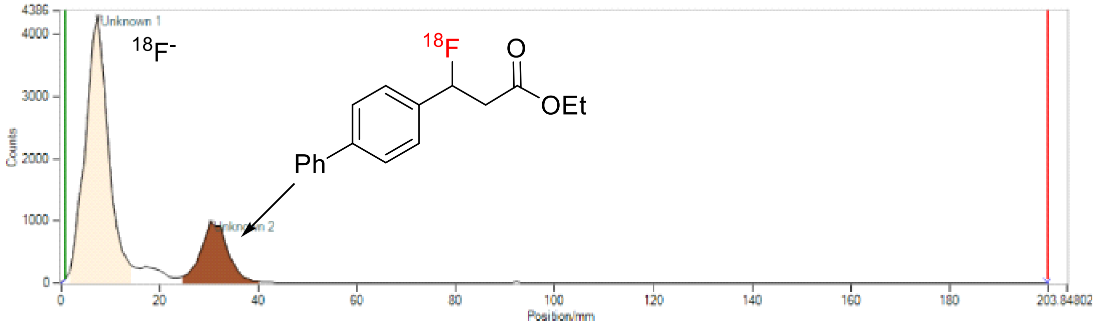

Figure S57. A representative Radio-TLC trace for the quantification of [^{18}F]**2o**.

Table S21. Average radiochemical conversions for [^{18}F]**2o**.

Trial number	RCC [^{18}F]**2o**
1	19.97
2	12.24
3	11.12
**Average**	14.44 ± 3.93 n = 3

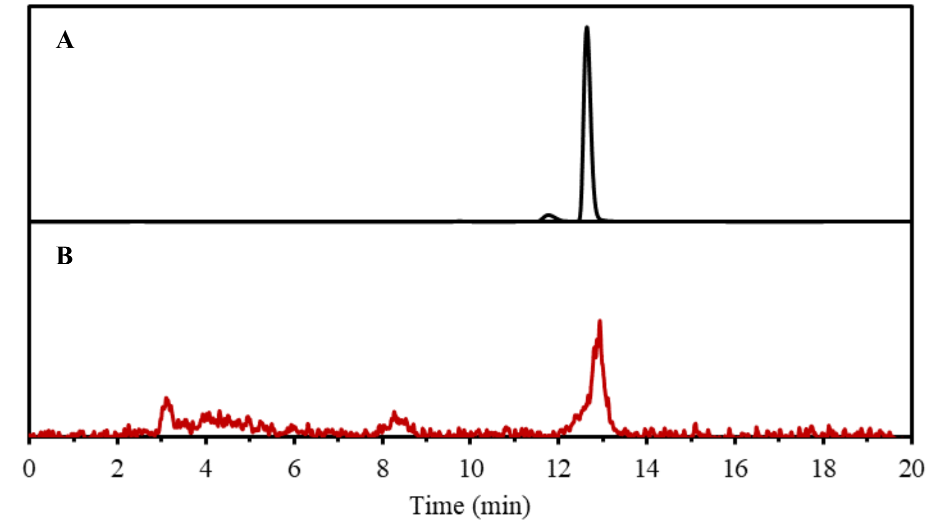

Figure S58. HPLC traces of standard (**A**) and ^{18}F-product (**B**) for **2o**.

(*E*)-4-(3-(fluoro-^{18}*F*)prop-1-en-1-yl)-1,2-dimethoxybenzene ([^{18}F]2p)

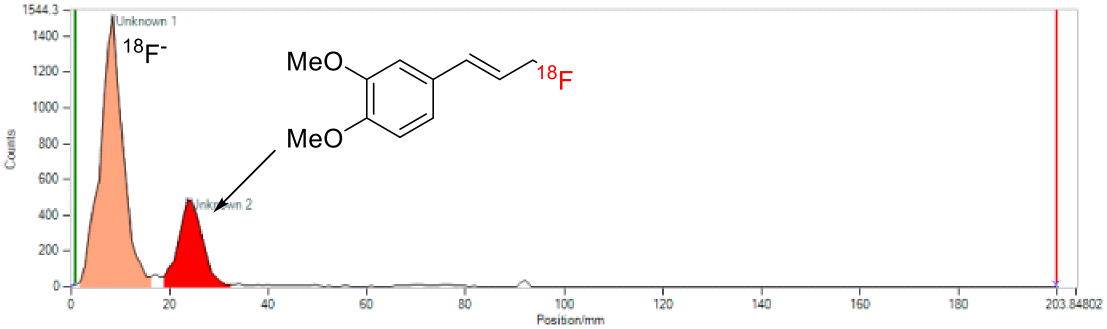

Figure S59. A representative Radio-TLC trace for the quantification of [^{18}F]**2p**.

Table S22. Average radiochemical conversions for [^{18}F]**2p**.

Trial number	RCC [^{18}F]**2p**
1	6.42
2	11.82
3	24.28
**Average**	14.17 ± 9.16 n = 3

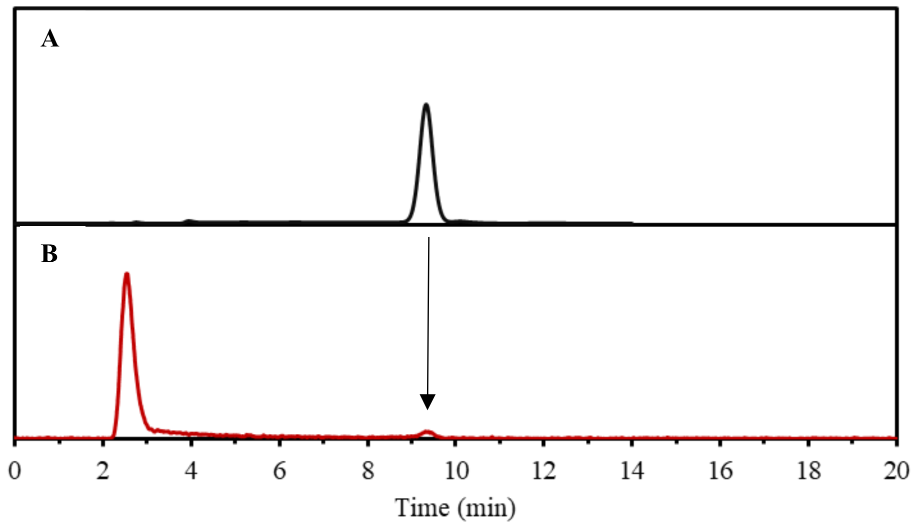

Figure S60. HPLC traces of standard (**A**) and ^{18}F-product (**B**) for **2p**.

(*E*)-1-(fluoro-^{18}*F*)-3-phenylallyl acetate ([^{18}F]2q)

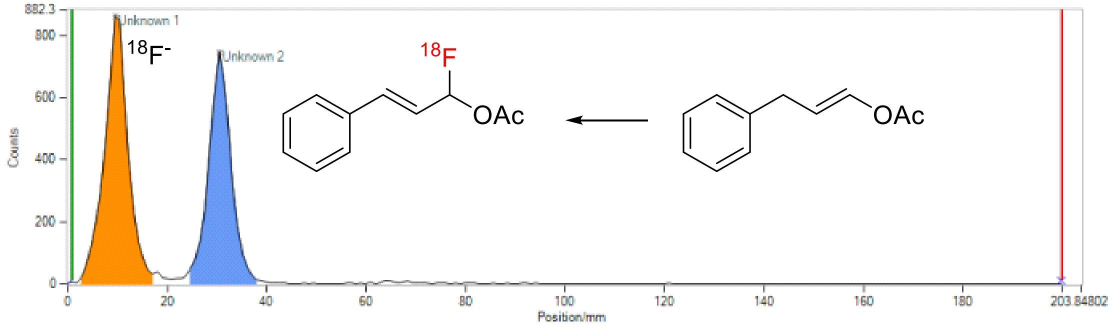

Figure S61. A representative Radio-TLC trace for the quantification of [^{18}F]**2q**.

Table S23. Average radiochemical conversions for [^{18}F]**2q**.

Trial number	RCC [^{18}F]**2q**
1	43.29
2	49.22
3	44.46
4	43.14
**Average**	45.03 ± 2.47 n = 4

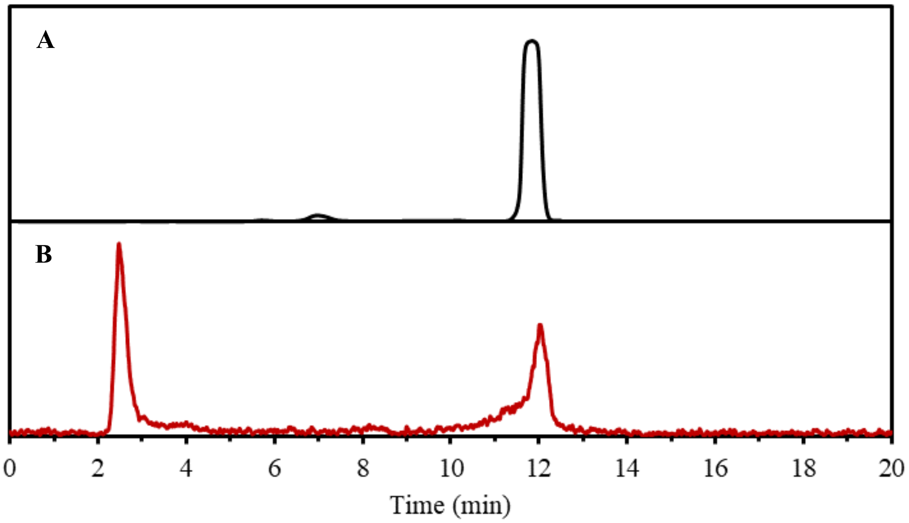

Figure S62. HPLC traces of standard (**A**) and ^{18}F-product (**B**) for **2q**.

(*E*)-1-(fluoro-^{18}*F*)-3-phenylallyl acetate ([^{18}F]2r)

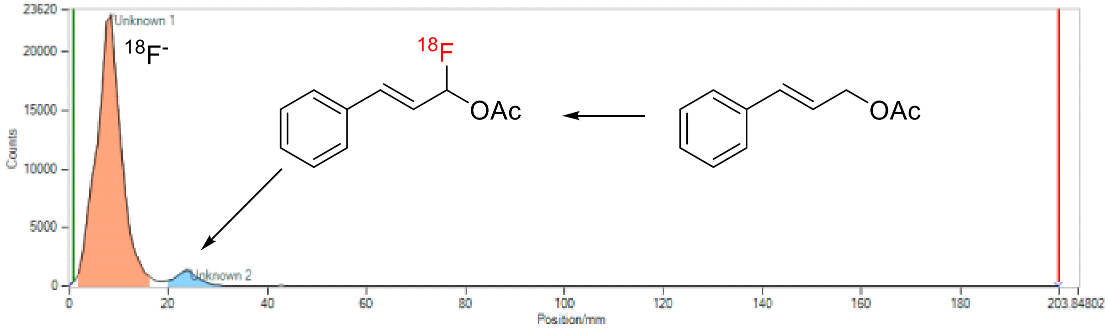

Figure S63. A representative Radio-TLC trace for the quantification of [^{18}F]**2r**.

Table S24. Average radiochemical conversions for [^{18}F]**2r**.

Trial number	RCC [^{18}F]**2r**
1	8.23
2	10.12
3	6.75
**Average**	8.37 ± 1.69 n = 3

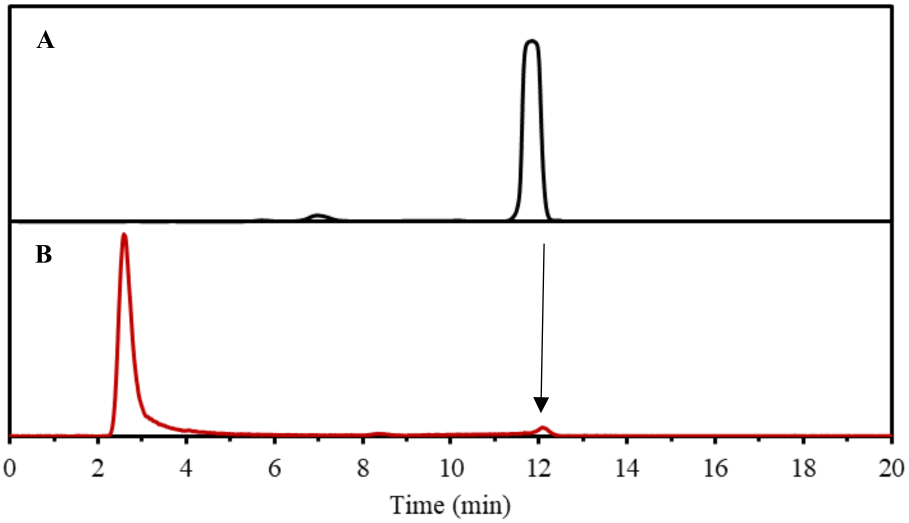

Figure S64. HPLC traces of standard (**A**) and ^{18}F-product (**B**) for **2r**.

tert-butyl (6*R*,12a*R*)-6-(2-(fluoro-^{18}*F*)benzo[*d*][1,3]dioxol-5-yl)-2-methyl-1,4-dioxo-1,3,4,6,12,12a-hexahydropyrazino[1',2':1,6]pyrido[3,4-*b*]indole-7(2*H*)-carboxylate ([^{18}F]2s)

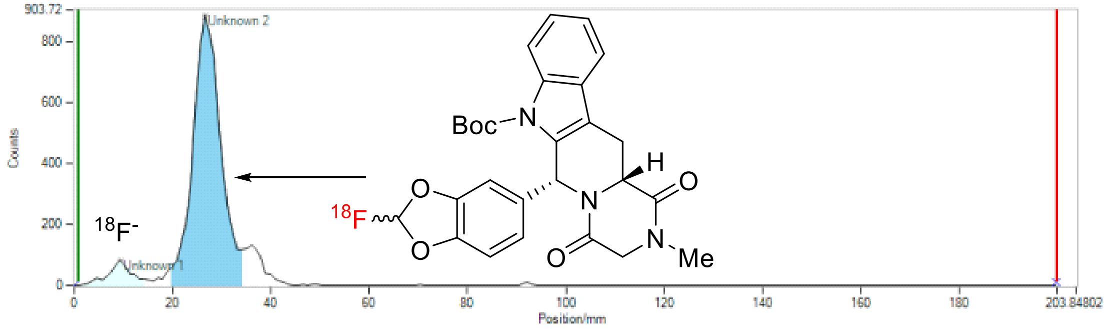

Figure S65. A representative Radio-TLC trace for the quantification of [^{18}F]**2s**.

Table S25. Average radiochemical conversions for [^{18}F]**2s**.

Trial number	RCC [^{18}F]**2s**
1	81.48
2	65.61
3	82.86
4	65.1
**Average**	73.76 ± 8.42 n = 4

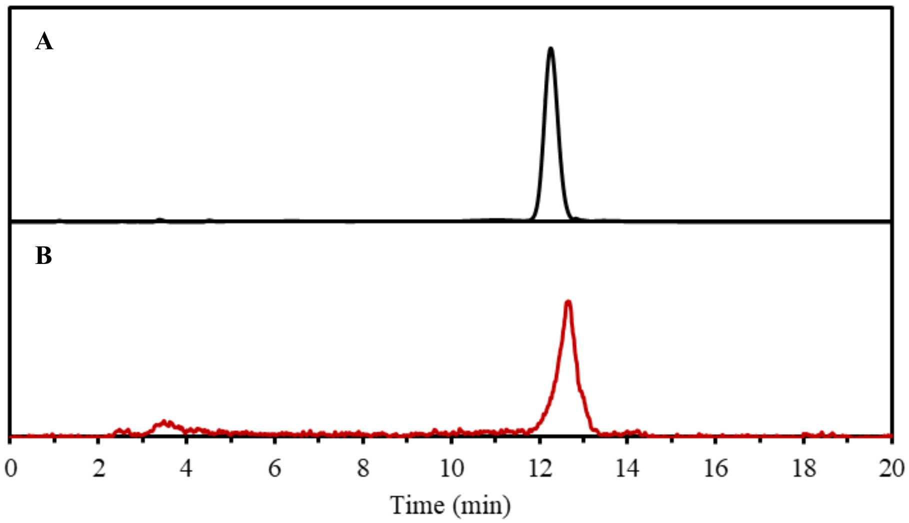

Figure S66. HPLC traces of standard (**A**) and ^{18}F-product (**B**) for **2s**.

Computational Details

DFT calculations were performed in ORCA[18] or Gaussian[19]. Geometry optimizations were performed at the B3LYP/6-31G(d) (SDD for Cu, Mn) level of theory with D3BJ dispersion correction. A frequency calculation was then performed on the optimized structures at the same level of theory, confirming the absence of imaginary frequencies. A single point energy calculation on the optimized structure was then performed at B3LYP/def2-TZVP (SDD for Cu, Mn) level of theory, with CPCM solvent modeling (dichloromethane) and D3BJ dispersion correction. Calculation of asynchronicity factor was performed as previously reported for related formally copper(III) complexes.[20,21]

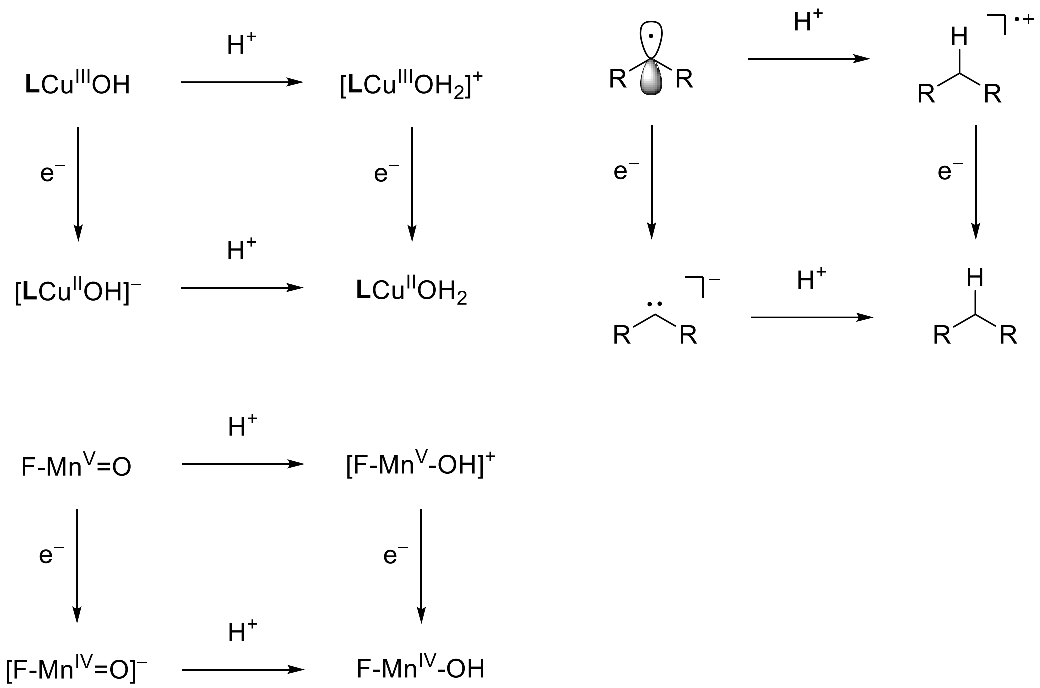

Asynchronicity Factor (η) $= 1/\sqrt{2}\,[-G(\mathrm{Cu^{II}OH^{-}}) + G(\mathrm{Sub^{-}}) + G(\mathrm{Cu^{III}OH_2^{+}}) - G(\mathrm{SubH^{+}})$

Asynchronicity Factor (η) $= 1/\sqrt{2}\,[-G(\mathrm{F\text{-}Mn^{IV}{=}O^{-}}) + G(\mathrm{Sub^{-}}) + G(\mathrm{F\text{-}Mn^{V}\text{-}OH^{+}}) - G(\mathrm{SubH^{+}})$

Table S26. Gibbs energies of species for the calculation of asynchronicity factor.

**Compound**	***G* (hartree)**
**L**$Cu^{III}OH_2$+	-1789.520652
**L**$Cu^{II}OH$	-1789.727899
1,3-benzodioxole•+	-420.3733063
1,3-benzodioxole−	-420.0326147
p-ethylanisole•+	-424.8854909
p-ethylanisole−	-424.5504335
4-chloro(ethylbenzene)•+	-769.9585711
4-chloro(ethylbenzene)−	-769.6597
F-Mn^{IV}=O	-4175.296387
F-Mn^{V}-OH	-4175.671936
Tadalafil•+	-1659.793995
Tadalafil− (H_a)	-1659.46288
Tadalafil− (H_b)	-1659.478232
Tadalafil− (H_g)	-1659.449133

Table S27. Calculated asynchronicity factor for the HAT reaction between **L**$Cu^{III}OH$ and C-H substrates.

**Compound**	***η*** **(V)**
1,3-benzodioxole	1.553
p-ethylanisole	1.445
4-chloro(ethylbenzene)	0.748
Tadalafil (H_a)	1.37
Tadalafil (H_b)	1.07
Tadalafil (H_g)	1.63

Table S28. Calculated asynchronicity factor for the HAT reaction between F-Mn^V=O and C-H substrates.

**Compound**	***η*** **(V)**
1,3-benzodioxole	-0.671
p-ethylanisole	-0.779
4-chloro(ethylbenzene)	-1.475

Cartesian coordinates of complexes and C-H substrates used in computations

Complex – Level of theory optimized

Charge, spin multiplicity

L$Cu^{III}OH_2$+ – B3LYP/6-31G(d) D3BJ (SDD for Cu)

1, 1

29	6.901473000	5.458450000	3.759180000
8	5.811806000	6.019392000	5.250280000
8	5.056741000	4.135925000	0.500467000
8	10.831370000	5.995458000	4.023070000
7	8.578983000	5.842724000	4.574064000
7	7.927063000	5.079288000	2.281885000
7	5.458406000	4.886620000	2.664413000
6	4.149712000	4.807334000	3.171133000
6	7.293580000	4.652639000	1.189520000
6	5.802449000	4.519197000	1.386037000

6	9.421566000	4.562234000	0.098182000
1	10.023348000	4.354437000	-0.781139000
6	10.042567000	5.012374000	1.271686000
1	11.113434000	5.168794000	1.345029000
6	9.242851000	5.266104000	2.378651000
6	8.559196000	5.356919000	6.949115000
6	3.617810000	3.557377000	3.582524000
6	2.134171000	5.949192000	3.865028000
1	1.549821000	6.854829000	3.986683000
6	8.509130000	8.129978000	7.475907000
1	8.512538000	9.188778000	7.707979000
6	8.622396000	7.710429000	6.149863000
6	8.680371000	3.868071000	6.641966000
1	9.341580000	3.781459000	5.771996000
6	9.671298000	5.749275000	3.744865000
6	4.023461000	7.331019000	2.850275000
1	5.110570000	7.227438000	2.927155000
6	8.391791000	7.205047000	8.515112000
1	8.292686000	7.555923000	9.538192000
6	8.624729000	6.312492000	5.897082000
6	8.726620000	8.696003000	4.994784000
1	9.450887000	8.279786000	4.285279000
6	8.426120000	5.833513000	8.256104000
1	8.378343000	5.134817000	9.084002000
6	7.374360000	8.828697000	4.266958000
1	7.015700000	7.852574000	3.933817000
1	7.466072000	9.477038000	3.389094000
1	6.610320000	9.250501000	4.929190000
6	4.404131000	2.267055000	3.436050000
1	5.444087000	2.532230000	3.214162000
6	4.406172000	1.438752000	4.732057000

1	4.758268000	2.027196000	5.586957000
1	5.064419000	0.570374000	4.620612000
1	3.406923000	1.060906000	4.973524000
6	9.315372000	3.065200000	7.786247000
1	10.260704000	3.511737000	8.109642000
1	9.518124000	2.042612000	7.451685000
1	8.652516000	2.996290000	8.656328000
6	3.686673000	7.550875000	1.362073000
1	3.988475000	6.693836000	0.752025000
1	4.193051000	8.446522000	0.984630000
1	2.607588000	7.689711000	1.231438000
6	3.418652000	6.020231000	3.324983000
6	2.318793000	3.542976000	4.096551000
1	1.873880000	2.599047000	4.394730000
6	8.033200000	4.375071000	0.047031000
1	7.521528000	4.022591000	-0.842044000
6	1.584683000	4.720101000	4.237481000
1	0.579019000	4.682041000	4.645884000
6	3.622214000	8.540473000	3.703191000
1	2.555938000	8.773270000	3.611313000
1	4.176171000	9.425772000	3.374219000
1	3.846047000	8.375171000	4.763523000
6	9.244711000	10.078860000	5.409984000
1	8.521396000	10.616294000	6.034047000
1	9.419515000	10.689088000	4.518040000
1	10.188313000	10.005799000	5.960155000
6	3.867424000	1.444875000	2.247585000
1	2.832110000	1.134102000	2.428917000
1	4.471119000	0.540765000	2.109182000
1	3.894638000	2.029291000	1.324006000
6	7.325771000	3.247636000	6.249416000

1	6.598033000	3.336630000	7.065919000
1	7.435197000	2.184711000	6.012215000
1	6.904241000	3.736780000	5.365600000
1	6.217526000	5.926599000	6.136371000
1	4.887668000	5.691693000	5.241971000

LCu^{II}OH$_2$ – B3LYP/6-31G(d) D3BJ (SDD for Cu)

−1, 2

29	6.731583000	5.482412000	3.945017000
8	5.634772000	6.044774000	5.327707000
8	5.111437000	3.966761000	0.471914000
8	10.828277000	5.879379000	4.094712000
7	8.562236000	5.913874000	4.689075000
7	7.853577000	4.987200000	2.431185000
7	5.331717000	4.752249000	2.673242000
6	3.950855000	4.696088000	2.963110000
6	7.263266000	4.497361000	1.341214000
6	5.757526000	4.375345000	1.453160000
6	9.416950000	4.309799000	0.312132000
1	10.042054000	4.040314000	-0.536113000
6	10.006458000	4.816488000	1.476012000
1	11.076222000	4.956347000	1.583424000
6	9.171953000	5.151962000	2.539312000
6	9.036959000	5.515461000	7.051487000
6	3.309465000	3.463318000	3.197009000
6	1.877198000	5.867273000	3.389842000
1	1.318759000	6.795064000	3.480711000
6	8.821960000	8.267955000	7.546873000
1	8.732577000	9.333177000	7.746771000
6	8.691842000	7.802667000	6.232936000
6	9.087486000	4.023404000	6.756259000

1	9.579920000	3.907651000	5.784910000
6	9.620400000	5.699551000	3.879279000
6	3.973384000	7.216667000	2.840974000
1	5.007938000	7.029594000	3.133575000
6	9.061137000	7.389058000	8.599256000
1	9.161176000	7.765853000	9.614916000
6	8.792875000	6.417214000	5.988800000
6	8.393325000	8.765467000	5.094624000
1	8.540041000	8.212880000	4.162194000
6	9.168563000	6.020718000	8.347216000
1	9.354937000	5.340853000	9.173616000
6	6.918532000	9.205705000	5.140210000
1	6.261572000	8.329485000	5.125711000
1	6.674915000	9.843210000	4.279538000
1	6.710938000	9.778693000	6.054068000
6	4.080448000	2.154645000	3.139453000
1	5.137415000	2.407531000	3.016846000
6	3.956037000	1.363644000	4.452289000
1	4.287232000	1.971003000	5.302092000
1	4.572403000	0.455489000	4.414868000
1	2.920659000	1.053699000	4.642738000
6	9.896988000	3.213190000	7.776004000
1	10.906488000	3.621517000	7.902713000
1	9.987356000	2.172844000	7.440134000
1	9.411557000	3.193802000	8.760117000
6	3.954624000	7.568838000	1.343019000
1	4.319910000	6.731300000	0.739420000
1	4.581340000	8.447950000	1.139045000
1	2.932292000	7.796979000	1.011940000
6	3.238545000	5.910108000	3.084407000
6	1.942149000	3.461660000	3.495372000

1	1.435728000	2.513374000	3.663802000
6	8.029919000	4.143032000	0.233218000
1	7.530936000	3.750148000	-0.645416000
6	1.223476000	4.650440000	3.585721000
1	0.160723000	4.631630000	3.818865000
6	3.472486000	8.373158000	3.709858000
1	2.441826000	8.664244000	3.466548000
1	4.105682000	9.256576000	3.560663000
1	3.523690000	8.090587000	4.765731000
6	9.349859000	9.966884000	5.073054000
1	9.229586000	10.600558000	5.960998000
1	9.152663000	10.594421000	4.194397000
1	10.393328000	9.634430000	5.032781000
6	3.658529000	1.315582000	1.921642000
1	2.600772000	1.027637000	1.990220000
1	4.253506000	0.394038000	1.856575000
1	3.806320000	1.896751000	1.007234000
6	7.656726000	3.465213000	6.624641000
1	7.139440000	3.501424000	7.591994000
1	7.669838000	2.422442000	6.281011000
1	7.071663000	4.060050000	5.919584000
1	6.187672000	6.297826000	6.083040000

<u>1,3-Benzodioxole•+ – B3LYP/6-31G(d) D3BJ</u>

1, 2

6	2.072093000	5.787929000	3.426005000
1	1.445159000	6.671135000	3.494714000
6	2.178991000	3.318311000	3.559793000
1	1.728282000	2.344487000	3.710134000
6	1.461549000	4.503579000	3.627673000
1	0.397991000	4.468286000	3.840773000

6	3.423172000	5.936135000	3.148824000
6	3.536882000	3.463869000	3.281524000
6	4.146969000	4.747027000	3.080021000
1	3.892221000	6.900693000	2.995073000
8	4.465047000	2.518612000	3.156358000
8	5.443381000	4.576460000	2.834058000
6	5.714830000	3.165631000	2.867723000
1	6.427499000	2.952750000	3.668516000
1	6.072947000	2.842229000	1.887299000

1,3-Benzodioxole– – B3LYP/6-31G(d) D3BJ

-1, 1

6	1.998281000	5.781234000	3.349773000
1	1.380735000	6.678211000	3.320795000
6	2.195714000	3.362741000	3.593758000
1	1.744699000	2.386599000	3.756375000
6	1.409092000	4.539328000	3.553615000
1	0.330266000	4.463788000	3.684630000
6	3.398632000	5.898933000	3.177548000
6	3.571444000	3.477530000	3.430587000
6	4.173047000	4.745386000	3.222737000
1	3.869359000	6.866846000	3.021617000
8	4.501283000	2.519303000	3.489226000
8	5.501655000	4.629037000	3.142144000
6	5.870402000	3.126726000	3.109878000
1	5.832125000	2.963293000	1.996113000

p-ethylanisole•+ – B3LYP/6-31G(d) D3BJ

1, 2

6	2.330884000	6.117412000	3.497336000
6	2.164725000	3.717937000	3.474183000
1	1.588090000	2.804020000	3.585728000
6	1.551612000	4.930676000	3.649375000
1	0.498680000	4.987307000	3.897725000
6	3.725440000	6.032201000	3.168134000
6	3.557440000	3.610982000	3.144810000
6	4.314143000	4.804676000	2.998294000
1	4.278037000	6.959952000	3.062574000
1	5.366955000	4.743319000	2.749798000
6	4.148867000	2.247576000	2.970561000
6	5.633986000	2.169158000	2.620834000
8	1.869965000	7.337178000	3.636898000
6	0.481462000	7.596544000	3.969267000
1	0.246575000	7.152290000	4.939793000
1	0.404067000	8.680719000	4.015653000
1	-0.165194000	7.198070000	3.183447000
1	3.942008000	1.683074000	3.895549000
1	3.546449000	1.727357000	2.206677000
1	5.853107000	2.663789000	1.668484000
1	6.258451000	2.618778000	3.400262000
1	5.932345000	1.122100000	2.523749000

<u>p-ethylanisole– – B3LYP/6-31G(d) D3BJ</u>

-1, 1

6	1.944204000	6.023180000	2.996707000
6	1.972019000	3.595102000	3.035842000
1	1.419924000	2.654074000	3.046679000
6	1.273151000	4.787377000	3.027663000
1	0.181926000	4.784768000	3.031484000
6	3.344812000	6.017681000	2.985194000

6	3.421682000	3.529090000	3.024257000
6	4.064156000	4.831479000	2.992678000
1	3.869287000	6.974452000	2.956834000
1	5.151136000	4.875701000	2.967710000
6	4.121472000	2.332497000	3.037382000
6	5.619020000	2.253613000	3.022074000
8	1.222670000	7.235763000	2.989989000
6	0.954531000	7.708635000	4.290970000
1	1.881300000	7.903288000	4.856312000
1	0.390628000	8.648656000	4.197078000
1	0.354621000	6.990025000	4.874152000
1	3.562417000	1.397392000	3.054900000
1	6.092385000	2.715446000	2.128221000
1	6.111293000	2.751662000	3.885562000
1	5.958282000	1.206778000	3.039995000

<u>4-chloro(ethylbenzene)•+ – B3LYP/6-31G(d) D3BJ</u>

1, 2

6	2.115135000	5.744190000	3.528010000
6	2.190473000	3.343279000	3.441646000
1	1.712232000	2.371098000	3.519336000
6	1.458349000	4.484461000	3.636730000
1	0.399696000	4.451127000	3.870024000
6	3.507810000	5.831006000	3.222365000
6	3.593113000	3.399164000	3.132817000
6	4.224022000	4.675593000	3.030381000
1	3.974567000	6.807431000	3.147597000
1	5.281093000	4.729537000	2.798071000
6	4.331064000	2.120213000	2.932607000
6	5.820714000	2.200271000	2.606739000
1	4.161973000	1.508428000	3.837256000

1	3.790678000	1.557784000	2.149707000
1	6.003128000	2.738309000	1.670397000
1	6.386079000	2.687642000	3.408177000
1	6.222455000	1.190803000	2.488662000
17	1.230781000	7.169705000	3.765185000

4-chloro(ethylbenzene)– – B3LYP/6-31G(d) D3BJ

-1, 1

6	2.074622000	5.785149000	3.535215000
6	2.151846000	3.375289000	3.386912000
1	1.631865000	2.418007000	3.437626000
6	1.433772000	4.535734000	3.593702000
1	0.366380000	4.487817000	3.803233000
6	3.446970000	5.844090000	3.265610000
6	3.575333000	3.361169000	3.102780000
6	4.174680000	4.684233000	3.057014000
1	3.943852000	6.812144000	3.220478000
1	5.239355000	4.768375000	2.850451000
6	4.288720000	2.194439000	2.896398000
6	5.760146000	2.166566000	2.602858000
1	3.762915000	1.241767000	2.948627000
1	6.048427000	2.711019000	1.678762000
1	6.390530000	2.613278000	3.400753000
1	6.116172000	1.133828000	2.473466000
17	1.138107000	7.290506000	3.803317000

F-Mn^{V}-OH – B3LYP/6-31G(d) D3BJ (SDD for Mn)

1, 1

25	-1.201518000	0.001768000	-0.682238000
6	-1.609254000	-0.074605000	-4.084608000
6	-0.871606000	3.403733000	-0.758688000

6	-1.590515000	0.073804000	2.729672000
6	-0.869403000	-3.399845000	-0.605079000
7	-1.194430000	1.445413000	0.722415000
7	-1.231238000	1.392442000	-2.132457000
7	-1.238208000	-1.443958000	-2.074304000
7	-1.212036000	-1.391083000	0.774916000
6	-1.148311000	-2.778598000	-1.852217000
6	-1.568928000	-1.285879000	-3.412556000
6	-1.331812000	1.175010000	-3.465032000
6	-0.912636000	2.735653000	-1.970764000
6	-0.902155000	-2.732778000	0.610933000
6	-1.314856000	-1.175101000	2.111182000
6	-1.546562000	1.286604000	2.058166000
6	-1.140993000	2.783405000	0.493519000
6	-1.450925000	-3.508165000	-3.061566000
6	-1.741913000	-2.585776000	-4.016997000
6	-1.047485000	2.389821000	-4.193314000
6	-0.757005000	3.345260000	-3.270750000
6	-1.467057000	3.509476000	1.694901000
6	-1.746654000	2.583713000	2.653619000
6	-0.750502000	-3.346674000	1.908519000
6	-1.036306000	-2.391688000	2.835074000
1	-1.471083000	-4.583754000	-3.149857000
1	-2.037005000	-2.763841000	-5.039566000
1	-1.041711000	2.482452000	-5.268738000
1	-0.479304000	4.372742000	-3.448663000
1	-1.510548000	4.584832000	1.778673000
1	-2.054265000	2.761059000	3.672766000
1	-0.477678000	-4.375893000	2.083857000
1	-1.033211000	-2.488557000	3.910208000
6	-1.891828000	-0.058438000	-5.539134000

6	-1.067733000	-0.708582000	-6.466557000
6	-2.995343000	0.649311000	-6.034165000
6	-1.336046000	-0.670347000	-7.831187000
6	-3.283342000	0.697288000	-7.393573000
6	-2.448700000	0.033128000	-8.293884000
6	-0.599357000	4.860666000	-0.738239000
6	-1.433816000	5.788342000	-1.373831000
6	0.506250000	5.356217000	-0.035127000
6	-1.172621000	7.154414000	-1.325342000
6	0.787680000	6.716217000	0.023540000
6	-0.057587000	7.617492000	-0.625989000
6	-1.881132000	0.059953000	4.182472000
6	-2.987552000	-0.644592000	4.675449000
6	-1.061025000	0.713767000	5.110624000
6	-3.280785000	-0.686139000	6.034199000
6	-1.334179000	0.682044000	6.474149000
6	-2.449419000	-0.018781000	6.935130000
6	-0.600206000	-4.857513000	-0.625288000
6	0.503764000	-5.357998000	-1.327957000
6	-1.437827000	-5.780949000	0.012595000
6	0.779566000	-6.719702000	-1.382683000
6	-1.181957000	-7.147883000	-0.032533000
6	-0.068528000	-7.616429000	-0.730883000
8	0.567858000	-0.039843000	-0.726664000
9	-2.964280000	0.026265000	-0.641745000
9	-3.802865000	1.290599000	-5.182655000
9	-4.344644000	1.365690000	-7.837501000
9	-2.712589000	0.072835000	-9.593184000
9	-0.534340000	-1.290571000	-8.693745000
9	0.018935000	-1.368828000	-6.049839000
9	-2.521882000	5.370869000	-2.030367000

9	1.325296000	4.501617000	0.591367000
9	1.852026000	7.159824000	0.687564000
9	0.199332000	8.917817000	-0.576769000
9	-1.983346000	8.017594000	-1.932258000
9	-3.793004000	-1.287531000	3.823975000
9	0.028590000	1.371517000	4.694071000
9	-0.535317000	1.305332000	7.337158000
9	-2.718528000	-0.052752000	8.233400000
9	-4.344379000	-1.351457000	6.476722000
9	1.324202000	-4.509447000	-1.957255000
9	-2.524790000	-5.357733000	0.668043000
9	1.841989000	-7.169121000	-2.045841000
9	-1.995954000	-8.006808000	0.576488000
9	0.183718000	-8.917893000	-0.776948000
1	0.950048000	0.351603000	0.080483000

F-Mn^{IV}=O – B3LYP/6-31G(d) D3BJ (SDD for Mn)

-1, 4

25	-1.965504000	0.041431000	-0.602182000
6	-1.664179000	-0.096721000	-3.955106000
6	-0.813395000	3.395736000	-0.755383000
6	-1.609823000	0.067015000	2.581659000
6	-0.916181000	-3.439594000	-0.578855000
7	-1.177005000	1.453442000	0.625085000
7	-1.312057000	1.407245000	-2.056800000
7	-1.324849000	-1.489196000	-1.987069000
7	-1.297635000	-1.455790000	0.745907000
6	-1.305588000	-2.823899000	-1.799038000
6	-1.762156000	-1.311169000	-3.279049000
6	-1.279919000	1.131779000	-3.370369000
6	-0.831664000	2.687385000	-1.949921000

6	-0.864674000	-2.753277000	0.624186000
6	-1.118283000	-1.143053000	2.032523000
6	-1.802160000	1.214615000	1.833052000
6	-1.205951000	2.779573000	0.452249000
6	-1.766179000	-3.541483000	-2.976793000
6	-2.081574000	-2.588073000	-3.895734000
6	-0.719726000	2.240382000	-4.147356000
6	-0.409011000	3.203280000	-3.246078000
6	-1.854543000	3.450321000	1.564910000
6	-2.255393000	2.466909000	2.411286000
6	-0.334664000	-3.249715000	1.886567000
6	-0.509924000	-2.240528000	2.780296000
1	-1.874537000	-4.614459000	-3.068929000
1	-2.479601000	-2.733734000	-4.890912000
1	-0.560069000	2.248737000	-5.217616000
1	0.049828000	4.165459000	-3.429618000
1	-2.020015000	4.516230000	1.651580000
1	-2.807517000	2.575011000	3.335231000
1	0.124766000	-4.214542000	2.055380000
1	-0.236447000	-2.218095000	3.827753000
6	-1.926224000	-0.081622000	-5.417497000
6	-1.128044000	-0.781180000	-6.327937000
6	-2.975490000	0.680931000	-5.943355000
6	-1.371642000	-0.745583000	-7.698483000
6	-3.234934000	0.731027000	-7.310405000
6	-2.428488000	0.014580000	-8.189914000
6	-0.507149000	4.841409000	-0.736501000
6	-1.208471000	5.773614000	-1.512910000
6	0.482166000	5.346506000	0.118213000
6	-0.921549000	7.135477000	-1.463235000
6	0.778958000	6.704204000	0.185379000

6	0.074246000	7.602150000	-0.610836000
6	-1.910761000	0.066491000	4.032027000
6	-2.851529000	-0.824225000	4.563529000
6	-1.242750000	0.902475000	4.933563000
6	-3.134374000	-0.868710000	5.925482000
6	-1.510728000	0.870984000	6.299669000
6	-2.459605000	-0.017482000	6.795898000
6	-0.592198000	-4.884952000	-0.604549000
6	0.425914000	-5.380315000	-1.429209000
6	-1.285789000	-5.818234000	0.175231000
6	0.743645000	-6.734411000	-1.477444000
6	-0.979291000	-7.176209000	0.145335000
6	0.038786000	-7.635369000	-0.684533000
8	0.083893000	-0.071497000	-0.666864000
9	-3.290617000	1.532060000	-0.548393000
9	-3.776184000	1.373401000	-5.129078000
9	-4.255564000	1.457762000	-7.787394000
9	-2.669494000	0.055930000	-9.507215000
9	-0.587537000	-1.424012000	-8.549874000
9	-0.081735000	-1.498598000	-5.901514000
9	-2.201869000	5.382441000	-2.316679000
9	1.192799000	4.515838000	0.889591000
9	1.743260000	7.153926000	1.001851000
9	0.353511000	8.911310000	-0.556295000
9	-1.611786000	8.004882000	-2.216025000
9	-3.529356000	-1.646450000	3.757263000
9	-0.294862000	1.744468000	4.507762000
9	-0.850852000	1.678098000	7.143889000
9	-2.722983000	-0.053912000	8.108681000
9	-4.052927000	-1.718423000	6.406972000
9	1.140842000	-4.545714000	-2.191196000

9	-2.293480000	-5.428954000	0.964997000
9	1.730661000	-7.177133000	-2.269815000
9	-1.666864000	-8.047366000	0.898515000
9	0.339268000	-8.940382000	-0.720720000

Tadalafil•+ B3LYP/6-31G(d) D3BJ

1, 2

8	0.259297000	14.008263000	5.372950000
7	-0.938789000	12.762594000	8.514733000
8	-2.992984000	11.972358000	9.086098000
7	-1.786324000	13.125035000	5.882612000
7	1.576524000	12.487418000	11.325851000
6	1.707906000	13.422151000	9.255939000
6	2.986165000	13.548376000	9.859987000
6	-2.207281000	12.327504000	8.217734000
6	-2.577651000	14.402109000	12.620372000
8	-2.916522000	16.551070000	13.215888000
6	0.867677000	12.773719000	10.166235000
6	-0.602783000	12.616495000	9.945803000
1	-0.964958000	11.649415000	10.290459000
6	2.898817000	12.981394000	11.163623000
8	-3.393042000	14.339935000	13.698710000
6	0.088177000	12.875447000	7.466935000
1	0.521548000	11.881713000	7.270586000
6	-2.288401000	15.753335000	12.328577000
6	-0.479464000	13.395484000	6.133552000
6	-1.263188000	13.710299000	10.778710000
6	-1.489311000	16.115564000	11.254669000
1	-1.283776000	17.154527000	11.025972000
6	-0.973563000	15.067359000	10.490927000
1	-0.350002000	15.301314000	9.637056000

6	4.204171000	14.096752000	9.404046000
1	4.274746000	14.527167000	8.409934000
6	-2.081571000	13.362722000	11.861643000
1	-2.342407000	12.334459000	12.063504000
6	-2.370191000	13.500404000	4.597625000
1	-2.603107000	12.606684000	4.005971000
1	-3.291987000	14.069482000	4.759855000
1	-1.645876000	14.111190000	4.060517000
6	3.990822000	12.949074000	12.017829000
1	3.921997000	12.524012000	13.008804000
6	-2.591118000	12.283937000	6.750546000
1	-3.635098000	12.605067000	6.692801000
1	-2.568205000	11.230748000	6.426372000
6	5.188146000	13.505342000	11.540182000
1	6.057451000	13.497222000	12.190546000
6	1.230729000	13.807494000	7.900732000
1	0.880767000	14.848111000	7.872748000
1	2.029452000	13.746105000	7.158506000
6	5.297785000	14.071036000	10.255240000
1	6.246649000	14.487624000	9.933950000
6	-3.645974000	15.692032000	14.109382000
1	-3.280130000	15.837635000	15.129424000
1	-4.714757000	15.907171000	14.023922000
6	1.181696000	11.726931000	12.464625000
8	1.830468000	11.749481000	13.484491000
8	0.085118000	11.029031000	12.204513000
6	-0.465306000	10.063039000	13.217764000
6	-0.910680000	10.829915000	14.460962000
6	0.594287000	9.006003000	13.523356000
6	-1.646387000	9.448085000	12.471303000
1	-1.396424000	10.131539000	15.150306000

1	-0.061099000	11.284781000	14.972857000
1	-1.635630000	11.609822000	14.207449000
1	0.946669000	8.538429000	12.597972000
1	0.144396000	8.225835000	14.145916000
1	1.445071000	9.427900000	14.060099000
1	-2.394177000	10.199501000	12.198851000
1	-1.310616000	8.947689000	11.557704000
1	-2.132462000	8.705141000	13.110649000

Tadalafil– (H_g) B3LYP/6-31G(d) D3BJ

-1, 1

8	-0.430998000	14.990834000	5.959018000
7	-0.790191000	12.527096000	8.502166000
8	-2.406620000	10.907651000	8.756365000
7	-1.936673000	13.273448000	6.098259000
7	1.942918000	12.068107000	11.064626000
6	1.806112000	13.346363000	9.198836000
6	3.176374000	13.361858000	9.640913000
6	-1.810768000	11.739282000	8.076462000
6	-3.176964000	14.097327000	11.929755000
8	-3.292635000	15.819035000	13.391151000
6	1.090296000	12.556217000	10.045850000
6	-0.396611000	12.421209000	9.953166000
1	-0.713505000	11.419118000	10.235195000
6	3.241205000	12.565828000	10.815090000
8	-4.445625000	14.060971000	12.329449000
6	0.054355000	13.174498000	7.483778000
1	0.597472000	12.385624000	6.932764000
6	-2.483341000	15.156609000	12.573772000
6	-0.790363000	13.924085000	6.447551000
6	-1.127249000	13.431467000	10.828773000

6	-1.127167000	15.356330000	12.338531000
1	-0.597103000	16.168170000	12.828034000
6	-0.457041000	14.476970000	11.455403000
1	0.607109000	14.618689000	11.290683000
6	4.337485000	13.974446000	9.152189000
1	4.296576000	14.591274000	8.258050000
6	-2.520474000	13.237784000	11.059020000
1	-3.054947000	12.431053000	10.570082000
6	-2.810489000	13.822869000	5.078571000
1	-2.805006000	13.198759000	4.173508000
1	-3.838864000	13.886304000	5.454195000
1	-2.445707000	14.819718000	4.829462000
6	4.439941000	12.370479000	11.501788000
1	4.468981000	11.775122000	12.403451000
6	-2.224580000	11.946139000	6.617607000
1	-3.303416000	11.777774000	6.583890000
1	-1.755401000	11.162084000	5.998510000
6	5.583152000	12.989089000	10.992445000
1	6.528547000	12.856662000	11.512403000
6	1.113905000	14.088572000	8.101685000
1	0.631120000	14.994796000	8.486826000
1	1.796549000	14.403609000	7.308236000
6	5.536234000	13.781959000	9.833480000
1	6.445728000	14.252786000	9.468342000
6	-4.699982000	15.195357000	13.338029000
1	-5.204770000	15.936720000	12.666607000
6	1.583355000	11.432151000	12.263523000
8	2.353067000	11.328391000	13.200745000
8	0.338228000	10.966939000	12.195517000
6	-0.424616000	10.640436000	13.426397000
6	-0.525484000	11.899187000	14.287086000

6	0.238860000	9.467234000	14.150859000
6	-1.791669000	10.241367000	12.876421000
1	-1.178164000	11.697651000	15.143802000
1	0.456225000	12.198818000	14.663313000
1	-0.958513000	12.722931000	13.712217000
1	0.360798000	8.619700000	13.466379000
1	-0.407278000	9.147538000	14.976751000
1	1.215926000	9.745148000	14.547151000
1	-2.254300000	11.081996000	12.353791000
1	-1.698562000	9.397983000	12.183394000
1	-2.444442000	9.944400000	13.704438000

Tadalafil– (H_b) B3LYP/6-31G(d) D3BJ

-1, 1

8	-0.365601000	14.615797000	5.637360000
7	-0.742056000	12.870749000	8.692613000
8	-2.305415000	11.277636000	9.333289000
7	-1.885901000	12.993654000	6.188785000
7	1.985321000	12.704500000	11.235268000
6	1.971894000	13.465008000	9.083690000
6	3.333329000	13.325641000	9.478586000
6	-1.724034000	11.951435000	8.481217000
6	-3.354439000	14.479370000	11.715247000
8	-3.887094000	15.544392000	13.658764000
6	1.143912000	13.104818000	10.136291000
6	-0.272506000	13.184908000	10.030330000
6	3.339103000	12.864493000	10.830658000
8	-4.730418000	14.704926000	11.680023000
6	0.123180000	13.147062000	7.513914000
1	0.551906000	12.187895000	7.177754000
6	-2.849267000	14.974571000	12.909899000
6	-0.718410000	13.685006000	6.357020000

6	-1.166016000	13.790152000	10.970076000
6	-1.486754000	14.935476000	13.157046000
1	-1.062448000	15.358438000	14.063412000
6	-0.667985000	14.353694000	12.185469000
1	0.400974000	14.346415000	12.362069000
6	4.563430000	13.586133000	8.849038000
1	4.585090000	13.947312000	7.823228000
6	-2.576296000	13.898229000	10.740111000
1	-3.038188000	13.505712000	9.847877000
6	-2.772050000	13.305149000	5.086858000
1	-2.812102000	12.479269000	4.361191000
1	-3.790034000	13.494581000	5.451492000
1	-2.385391000	14.197908000	4.593109000
6	4.517901000	12.661326000	11.535303000
1	4.489885000	12.308761000	12.557215000
6	-2.178713000	11.832300000	7.018321000
1	-3.262266000	11.688760000	7.048042000
1	-1.747053000	10.915033000	6.581599000
6	5.731773000	12.916856000	10.877943000
1	6.666956000	12.756791000	11.409151000
6	1.304721000	14.049710000	7.881136000
1	0.915838000	15.058024000	8.088303000
1	1.963107000	14.130552000	7.011543000
6	5.748702000	13.377977000	9.554409000
1	6.702078000	13.578078000	9.068200000
6	-5.055325000	15.038603000	13.026798000
1	-5.396965000	14.127901000	13.553285000
1	-5.834996000	15.806353000	13.033298000
6	1.624875000	11.895115000	12.312301000
8	2.418285000	11.502076000	13.156684000
8	0.322450000	11.622273000	12.268527000

6	-0.362670000	10.851079000	13.301944000
6	-0.174572000	11.507176000	14.672201000
6	0.155454000	9.410448000	13.268578000
6	-1.825002000	10.928356000	12.854913000
1	-0.813783000	11.000425000	15.405640000
1	0.864433000	11.449071000	15.001558000
1	-0.478673000	12.557069000	14.617070000
1	0.010534000	8.985414000	12.269307000
1	-0.402276000	8.798549000	13.987824000
1	1.217777000	9.376712000	13.520550000
1	-2.212830000	11.942225000	12.984631000
1	-1.913481000	10.679964000	11.793389000
1	-2.431220000	10.235466000	13.451655000

Tadalafil– (H_g) B3LYP/6-31G(d) D3BJ

-1, 1

8	-0.026687000	14.289626000	5.462047000
7	-0.964460000	12.550406000	8.497859000
8	-2.973923000	11.541425000	9.000109000
7	-1.952824000	13.198582000	5.989713000
7	1.716871000	12.187972000	11.192846000
6	1.737136000	13.082971000	9.056215000
6	2.973748000	13.443196000	9.750608000
6	-2.180525000	12.049735000	8.199310000
6	-2.738772000	14.390649000	12.211218000
8	-2.649414000	16.360857000	13.335346000
6	1.002429000	12.231961000	9.921402000
6	-0.498026000	12.369896000	9.906584000
1	-0.992002000	11.465578000	10.261031000
6	2.926796000	12.894979000	11.054813000
8	-3.949861000	14.502619000	12.885569000

6	0.023534000	12.805135000	7.384100000
1	0.266170000	11.775386000	7.019244000
6	-1.960905000	15.510714000	12.480400000
6	-0.634783000	13.521208000	6.200805000
6	-1.036595000	13.520226000	10.779963000
6	-0.715210000	15.676078000	11.907279000
1	-0.107628000	16.550871000	12.115499000
6	-0.264764000	14.654482000	11.053688000
1	0.711999000	14.743240000	10.594665000
6	4.074990000	14.205215000	9.358675000
1	4.107921000	14.628602000	8.357706000
6	-2.313831000	13.383084000	11.369912000
1	-2.933122000	12.526465000	11.133511000
6	-2.637425000	13.700066000	4.815929000
1	-2.770737000	12.911321000	4.058873000
1	-3.627872000	14.086334000	5.089021000
1	-2.028232000	14.498627000	4.391630000
6	3.976912000	13.077664000	11.957914000
1	3.923048000	12.649845000	12.949234000
6	-2.583934000	12.122921000	6.729497000
1	-3.667718000	12.267091000	6.715046000
1	-2.383373000	11.141045000	6.264049000
6	5.069582000	13.843760000	11.541680000
1	5.891006000	14.009479000	12.235981000
6	1.244220000	13.510234000	7.844947000
1	1.740819000	14.187003000	7.166054000
6	5.123679000	14.406580000	10.258258000
1	5.987124000	14.999274000	9.963230000
6	-3.754225000	15.582152000	13.793778000
1	-3.528346000	15.183074000	14.797923000
1	-4.652937000	16.206029000	13.818092000

6	1.371303000	11.522347000	12.332724000
8	1.987751000	11.563652000	13.398276000
8	0.233470000	10.800401000	12.131895000
6	-0.487239000	10.232855000	13.260375000
6	-0.884235000	11.337676000	14.246134000
6	0.349579000	9.135220000	13.928228000
6	-1.730094000	9.629928000	12.599925000
1	-1.534145000	10.921671000	15.025994000
1	0.001293000	11.772198000	14.712950000
1	-1.431366000	12.126495000	13.720195000
1	0.648326000	8.388809000	13.183120000
1	-0.245530000	8.631601000	14.700529000
1	1.246557000	9.560455000	14.379566000
1	-2.310619000	10.402820000	12.088730000
1	-1.439931000	8.878229000	11.858234000
1	-2.364443000	9.151869000	13.355597000

NMR Spectra

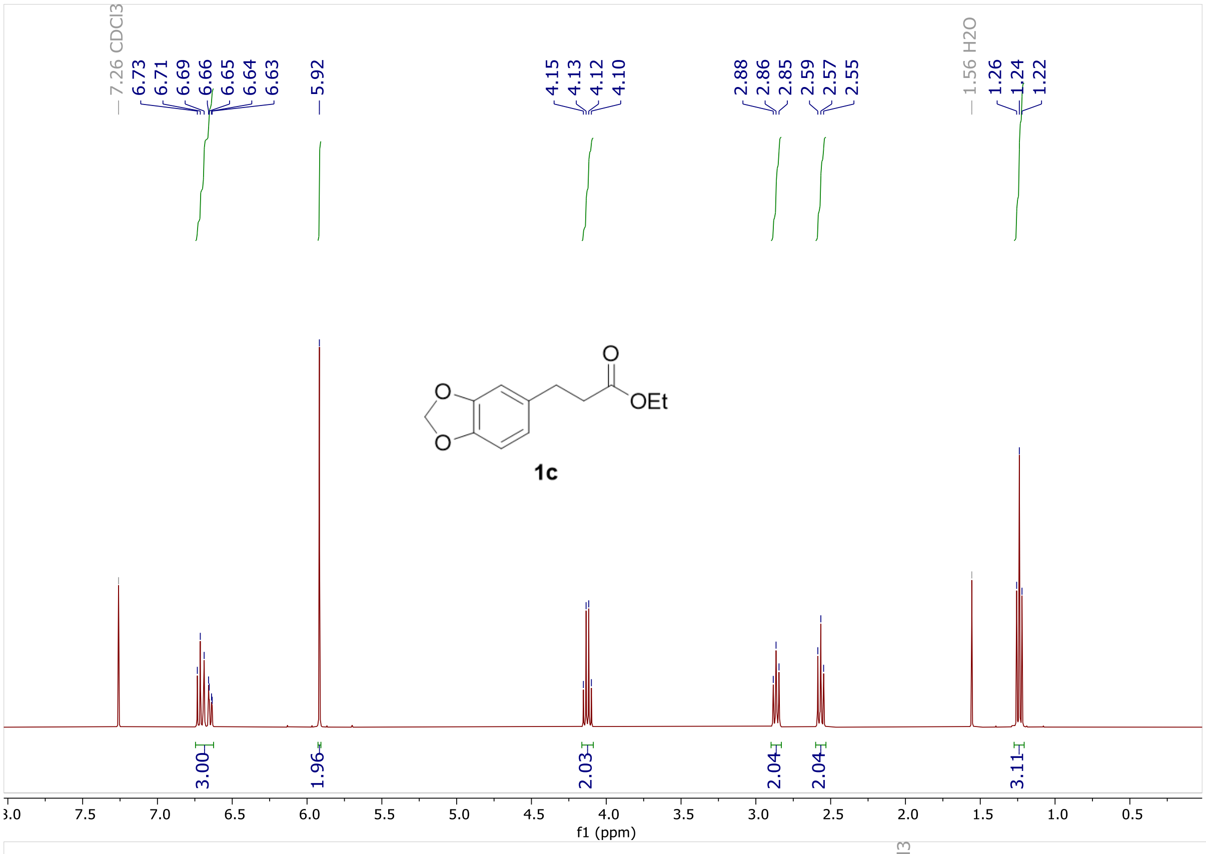
7.26 CDCl3
6.73
6.71
6.69
6.66
6.65
6.64
6.63
5.92
4.15
4.13
4.12
4.10
2.88
2.86
2.85
2.59
2.57
2.55
1.56 H2O
1.26
1.24
1.22
O
OEt
O
O
1c
3.00
1.96
2.03
2.04
2.04
3.11
f1 (ppm)

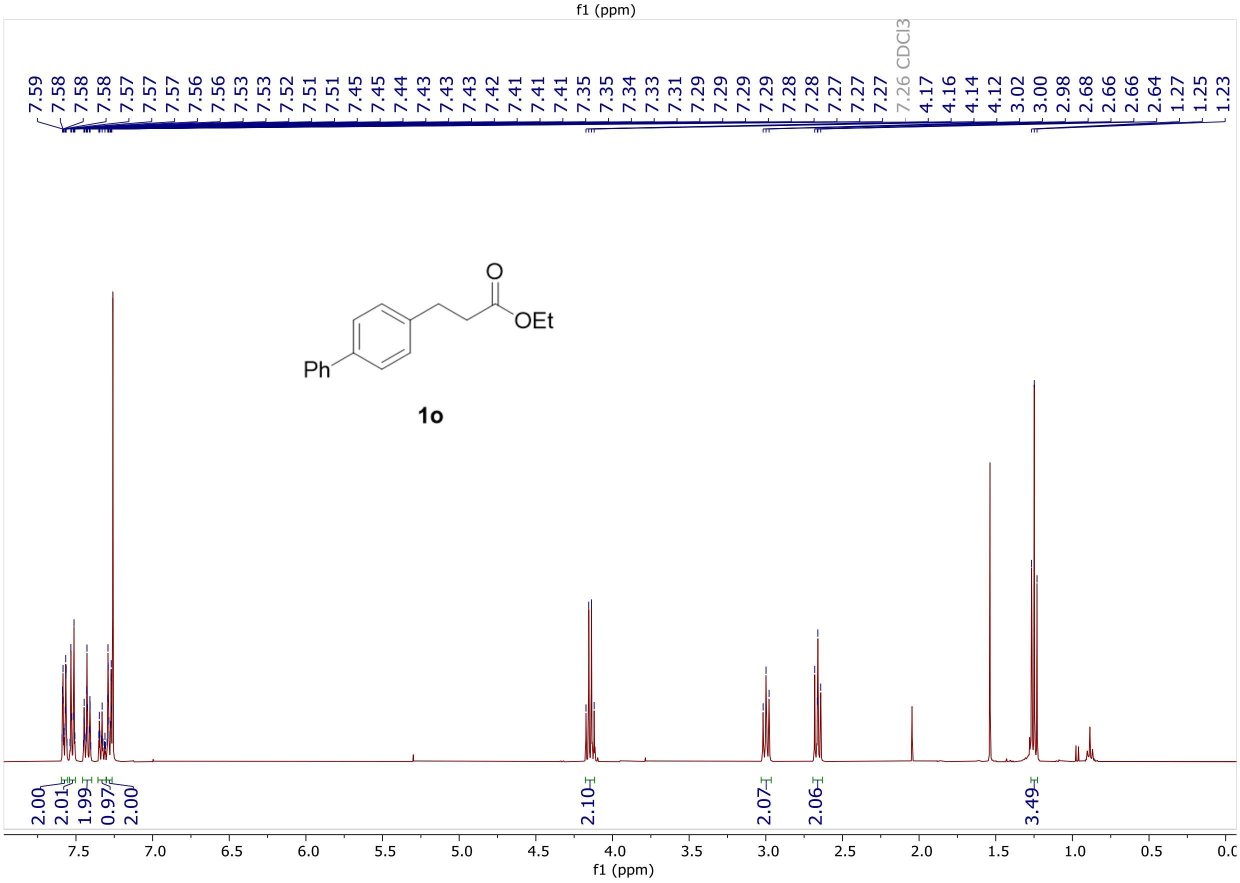
7.59
7.58
7.58
7.57
7.57
7.57
7.56
7.56
7.53
7.52
7.51
7.51
7.45
7.45
7.44
7.43
7.43
7.43
7.42
7.41
7.41
7.41
7.35
7.35
7.34
7.33
7.31
7.29
7.29
7.29
7.28
7.28
7.27
7.27
7.27
7.26 CDCl3
4.17
4.16
4.14
4.12
3.02
3.00
2.98
2.68
2.66
2.64
1.27
1.25
1.23
O
OEt
Ph
1o
2.00
2.01
1.99
0.97
2.00
2.10
2.07
2.06
3.49
f1 (ppm)

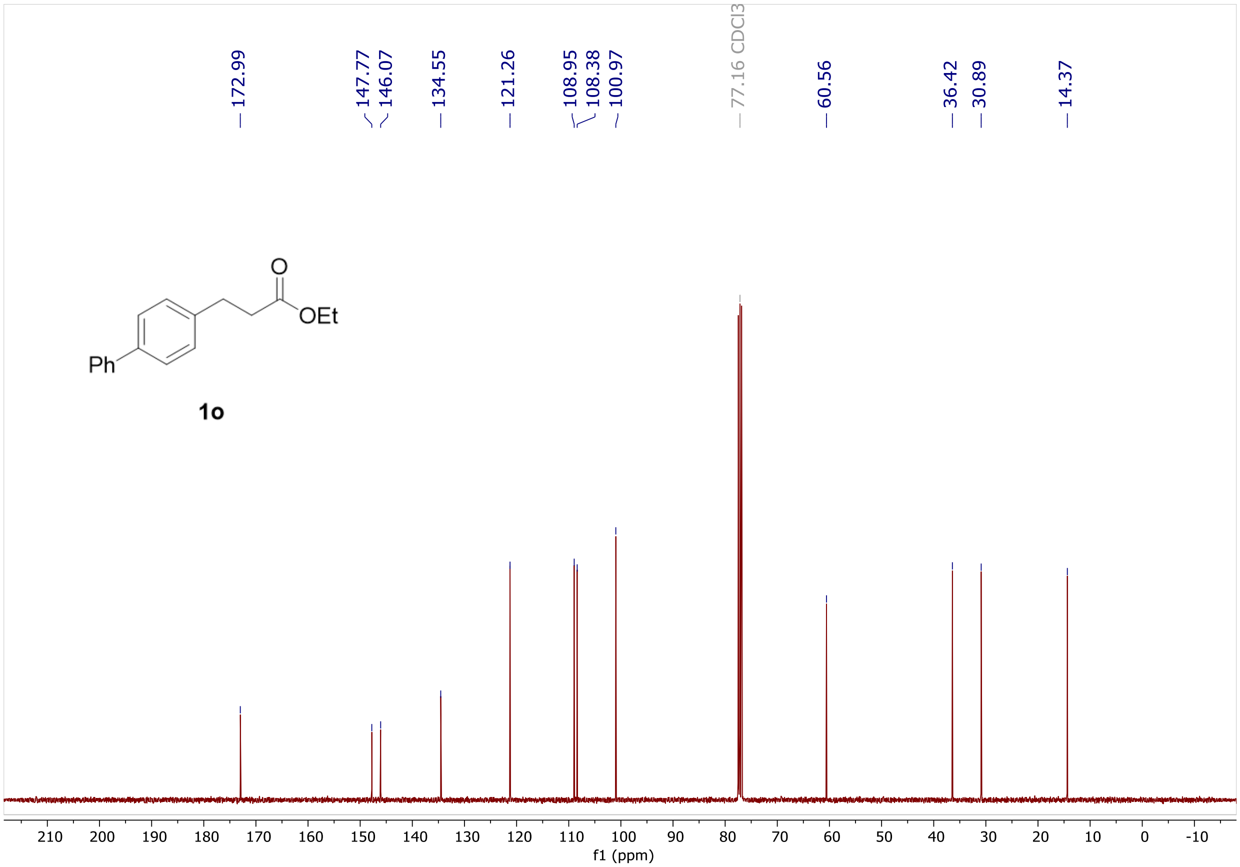

172.99
147.77
146.07
134.55
121.26
108.95
108.38
100.97
77.16 CDCl3
60.56
36.42
30.89
14.37
O
OEt
Ph
1o
210 200 190 180 170 160 150 140 130 120 110 100 90 80 70 60 50 40 30 20 10 0 -10
f1 (ppm)

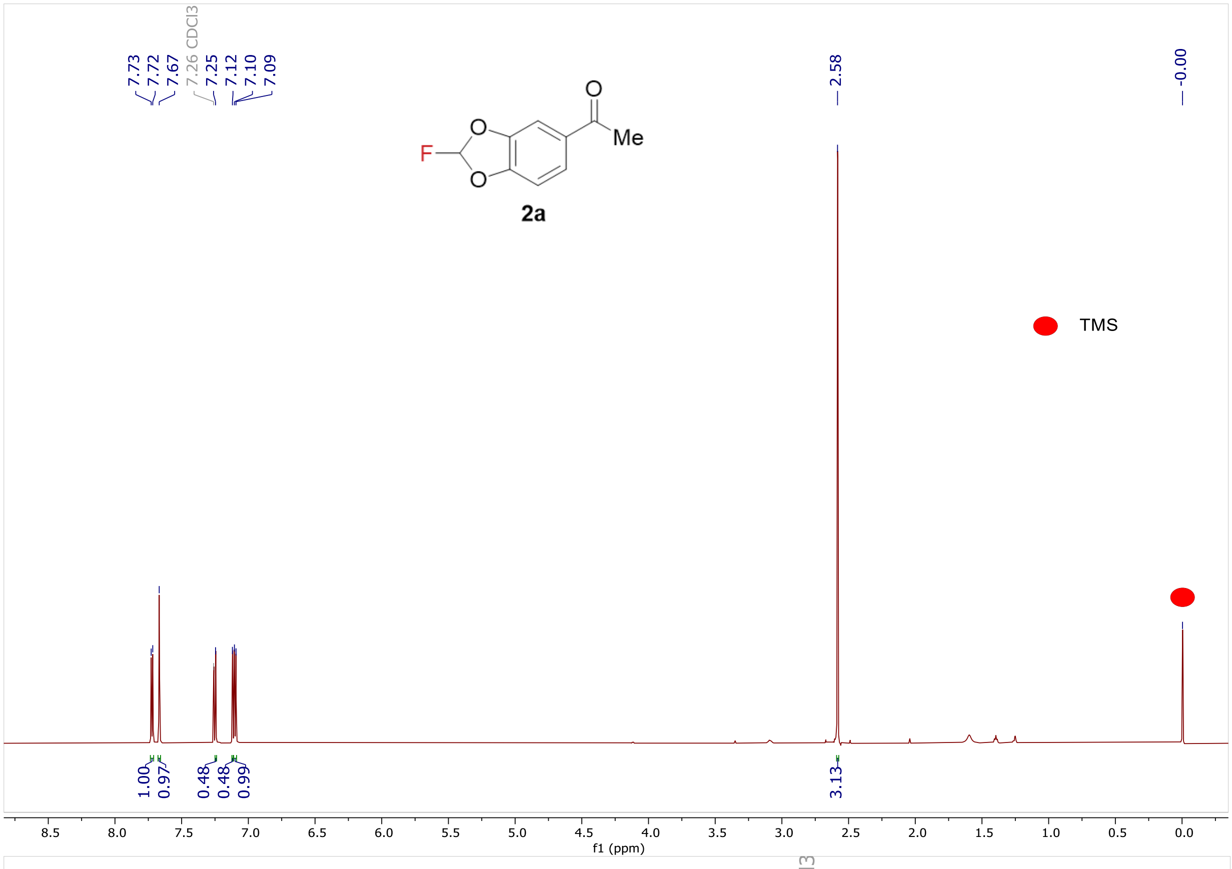
7.73
7.72
7.67
7.26 CDCl3
7.25
7.12
7.10
7.09
2.58
-0.00
F
O
O
O
Me
2a
TMS
1.00
0.97
0.48
0.48
0.99
3.13
f1 (ppm)

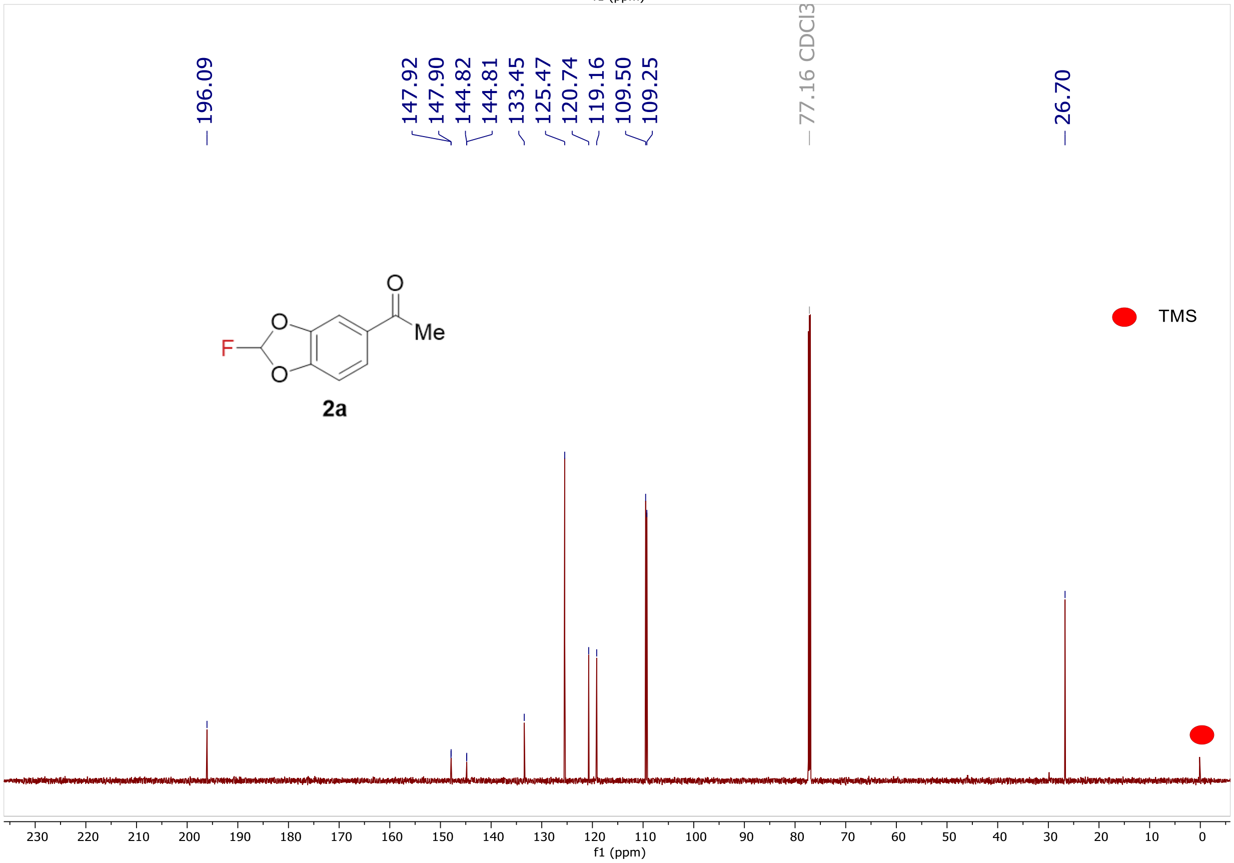
196.09
147.92
147.90
144.82
144.81
133.45
125.47
120.74
119.16
109.50
109.25
77.16 CDCl3
26.70
F
O
O
O
Me
2a
TMS
f1 (ppm)

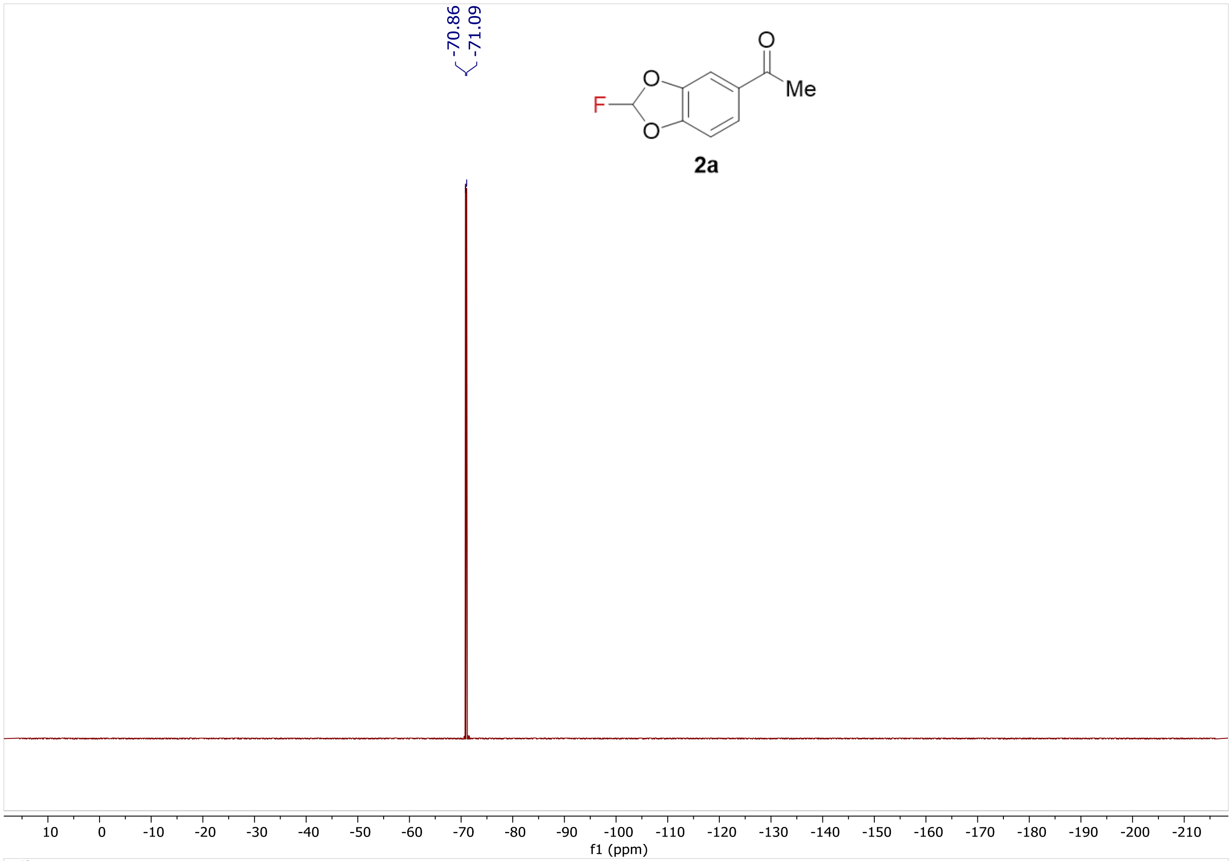
-70.86
-71.09
O
Me
O
F
O
2a
10
0
-10
-20
-30
-40
-50
-60
-70
-80
-90
-100
-110
-120
-130
-140
-150
-160
-170
-180
-190
-200
-210
f1 (ppm)

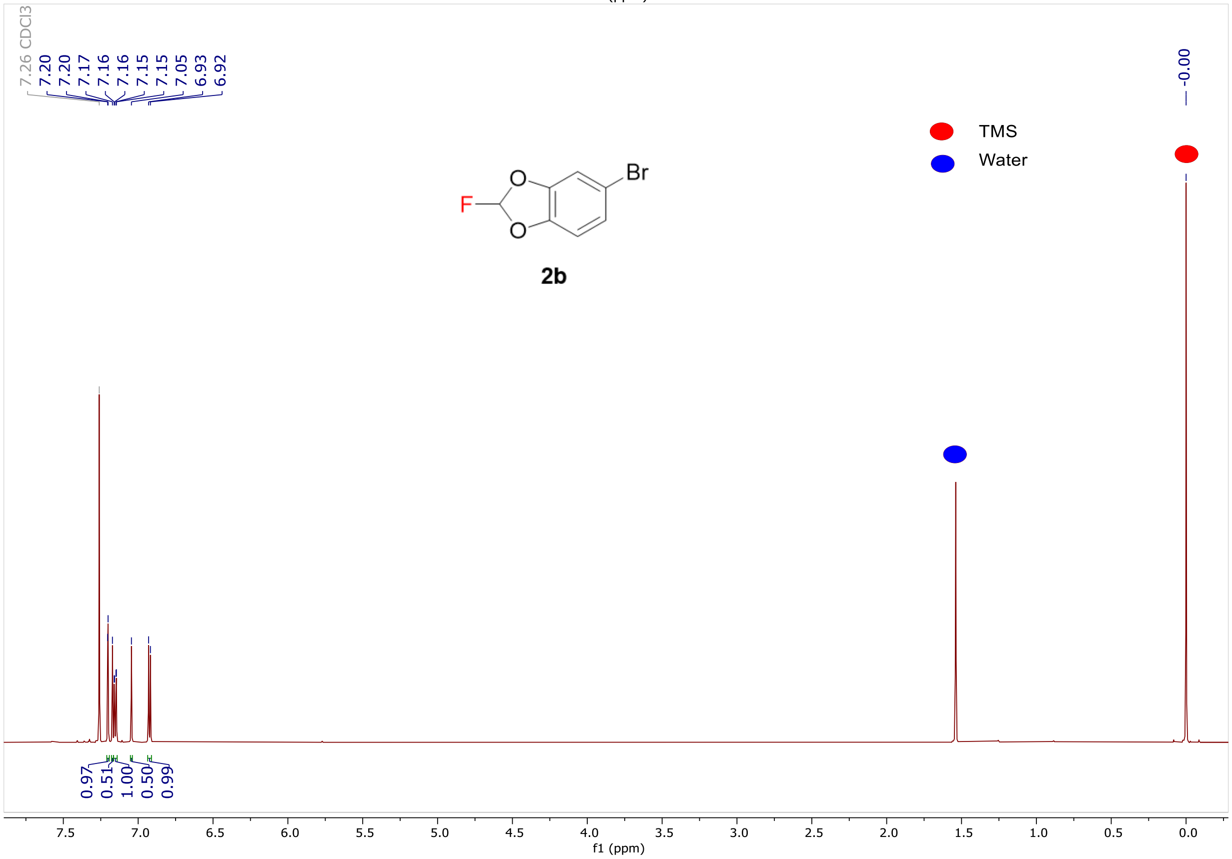
7.26 CDCl3
7.20
7.20
7.17
7.16
7.16
7.15
7.15
7.05
6.93
6.92
-0.00
TMS
Water
Br
O
F
O
2b
0.97
0.51
1.00
0.50
0.99
7.5
7.0
6.5
6.0
5.5
5.0
4.5
4.0
3.5
3.0
2.5
2.0
1.5
1.0
0.5
0.0
f1 (ppm)

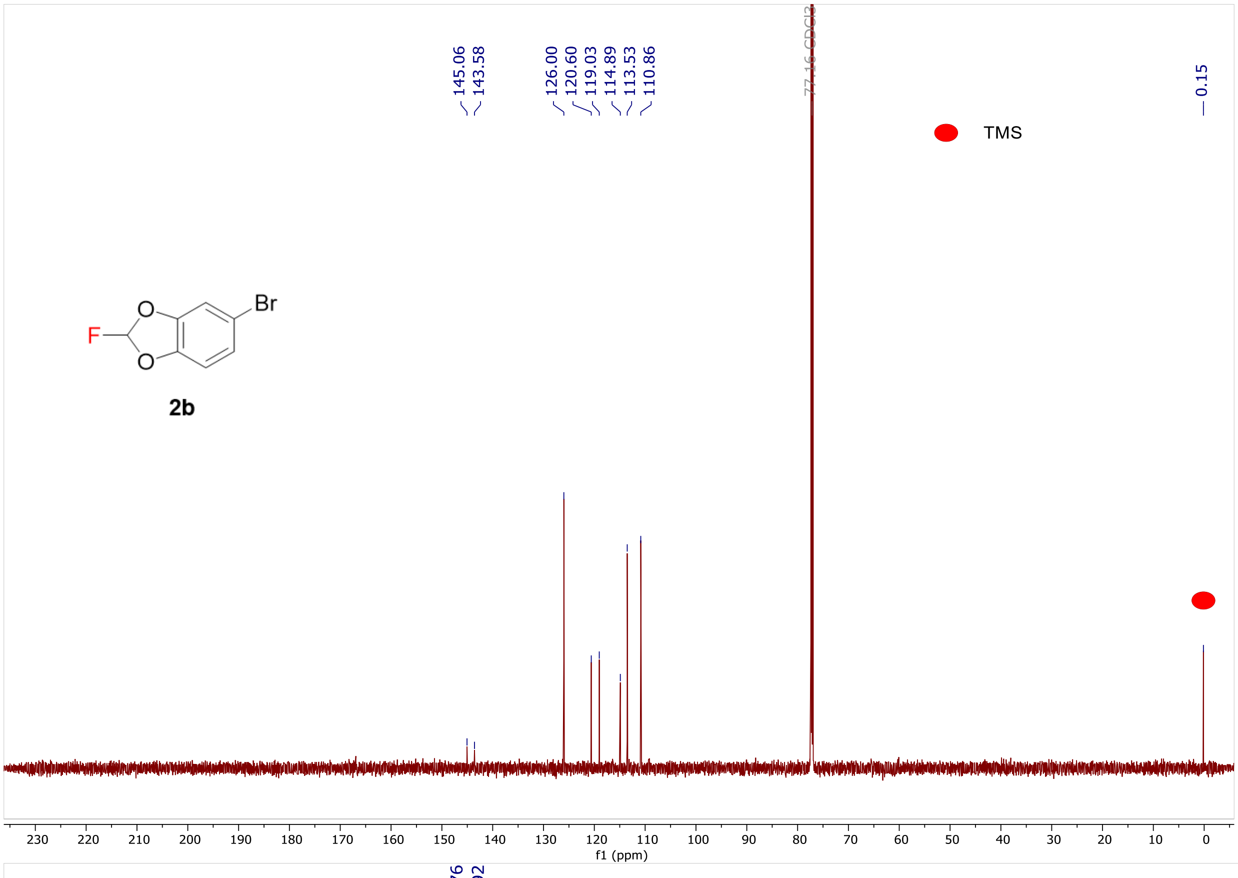
145.06
143.58
126.00
120.60
119.03
114.89
113.53
110.86
77.16 CDCl3
0.15
TMS
Br
F
O
O
2b
f1 (ppm)

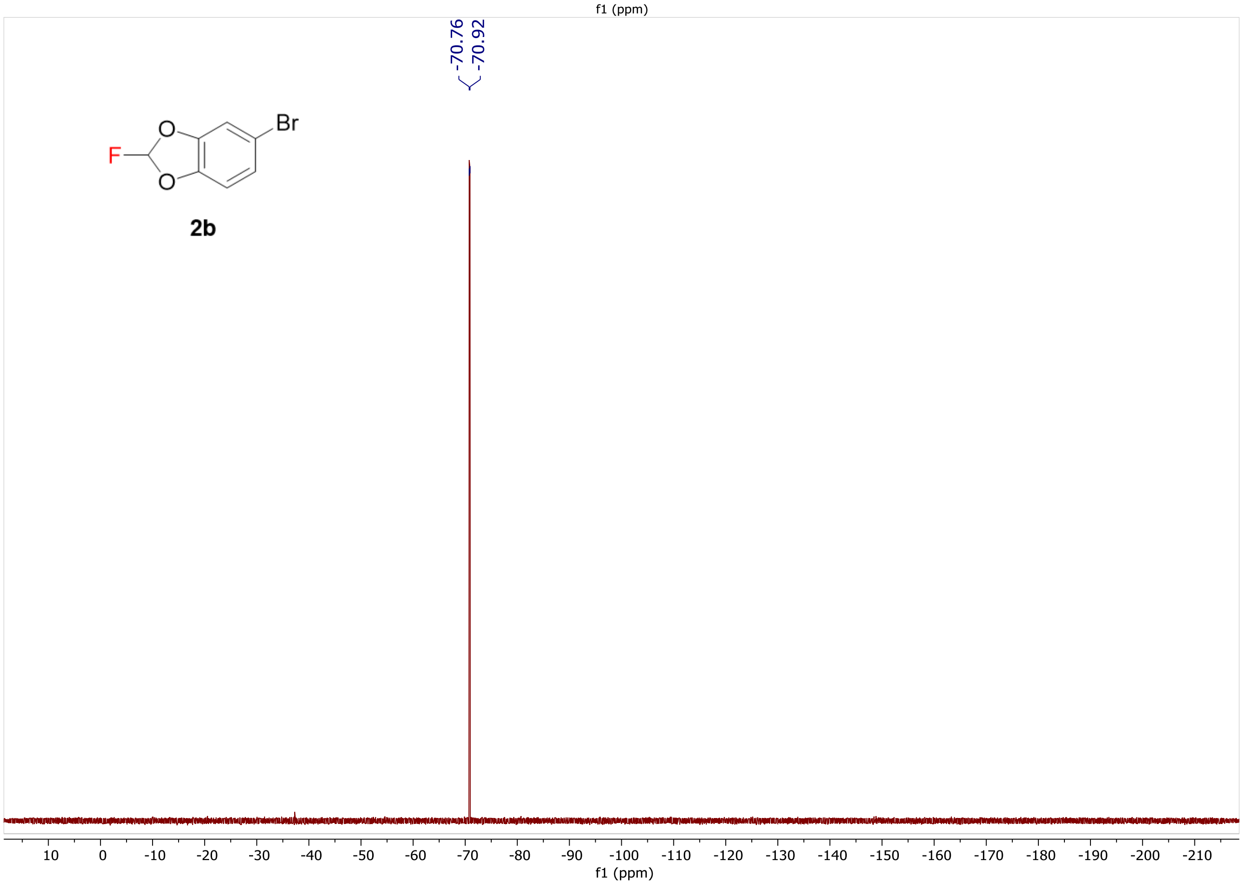
-70.76
-70.92
Br
F
O
O
2b
f1 (ppm)

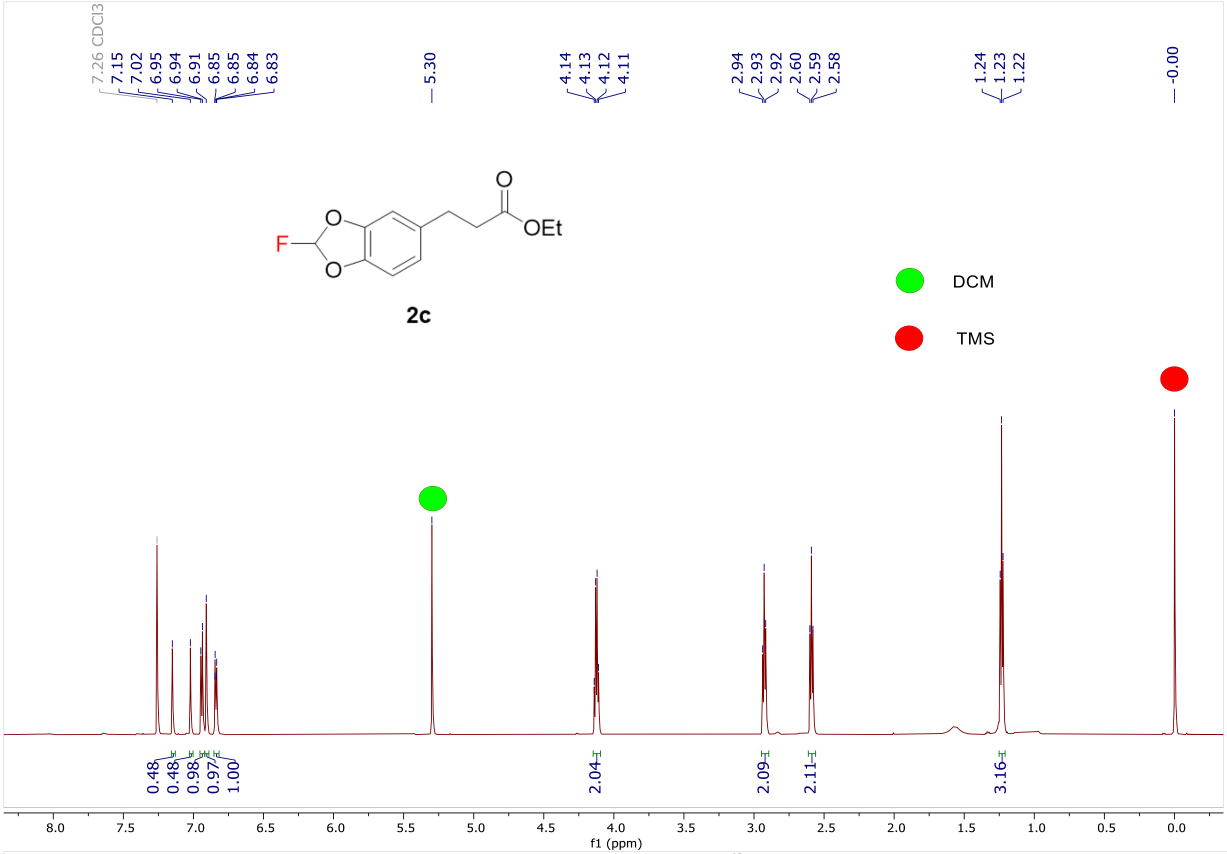
7.26 CDCl3
7.15
7.02
6.95
6.94
6.91
6.85
6.85
6.84
6.83
5.30
4.14
4.13
4.12
4.11
2.94
2.93
2.92
2.60
2.59
2.58
1.24
1.23
1.22
-0.00
O
F
O
O
OEt
2c
DCM
TMS
0.48
0.48
0.98
0.97
1.00
2.04
2.09
2.11
3.16
8.0
7.5
7.0
6.5
6.0
5.5
5.0
4.5
4.0
3.5
3.0
2.5
2.0
1.5
1.0
0.5
0.0
f1 (ppm)

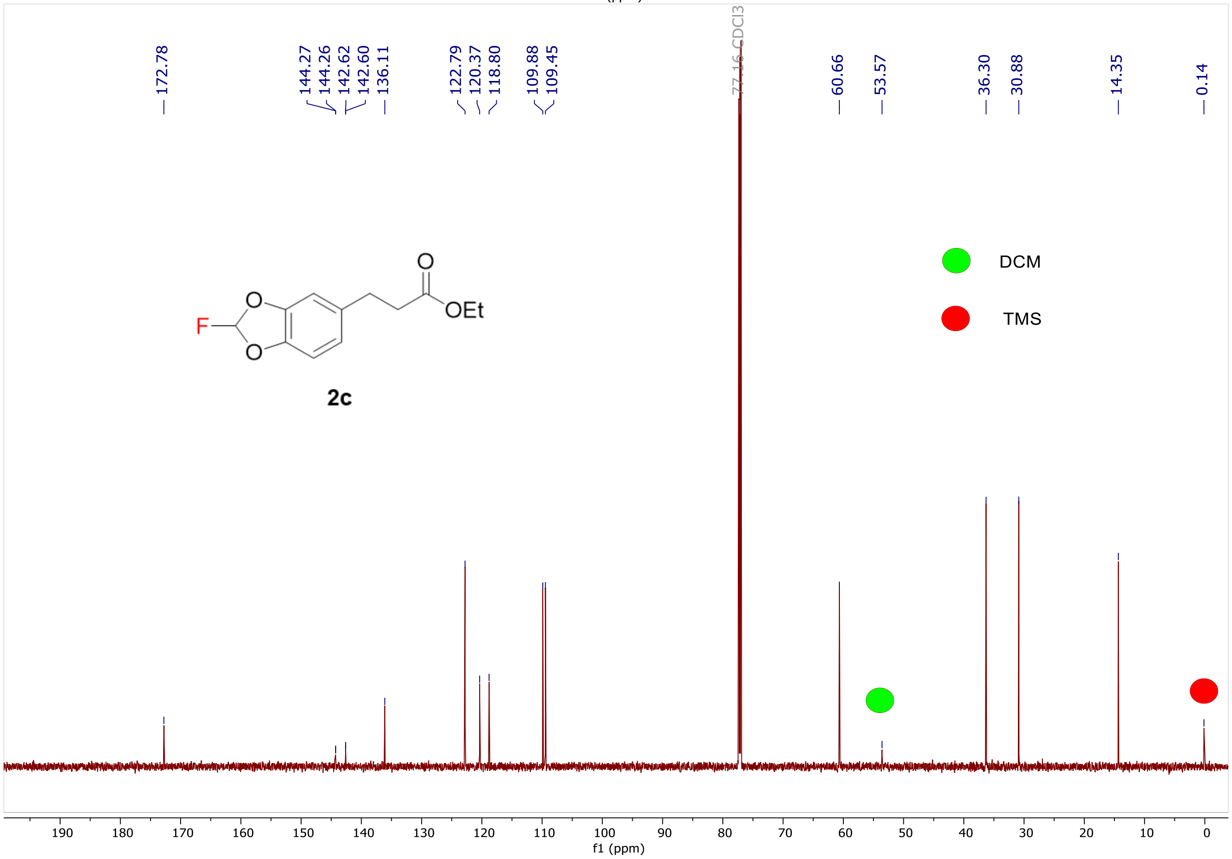
172.78
144.27
144.26
142.62
142.60
136.11
122.79
120.37
118.80
109.88
109.45
77.16 CDCl3
60.66
53.57
36.30
30.88
14.35
0.14
O
F
O
O
OEt
2c
DCM
TMS
190
180
170
160
150
140
130
120
110
100
90
80
70
60
50
40
30
20
10
0
f1 (ppm)

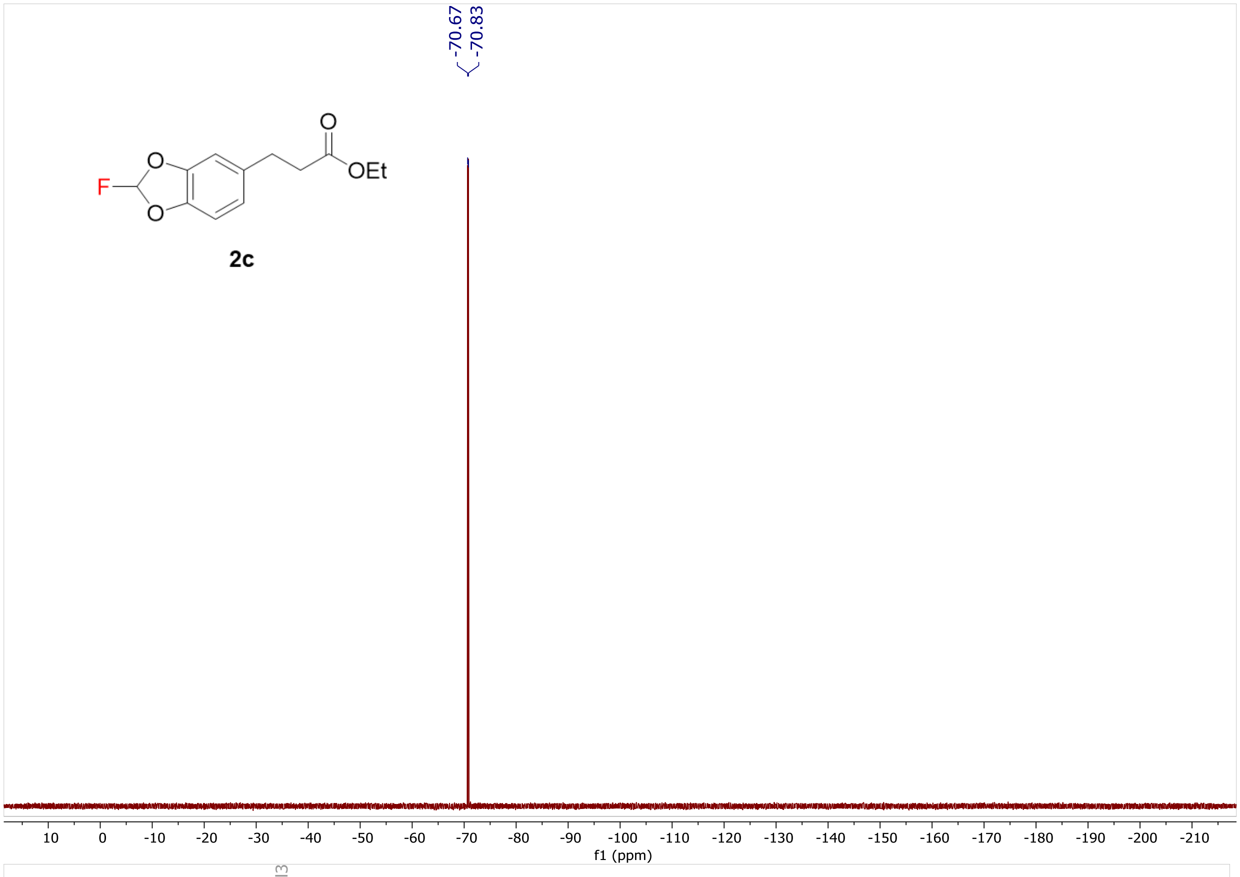
-70.67
-70.83
O
O
F
O
OEt
2c
10
0
-10
-20
-30
-40
-50
-60
-70
-80
-90
-100
-110
-120
-130
-140
-150
-160
-170
-180
-190
-200
-210
f1 (ppm)

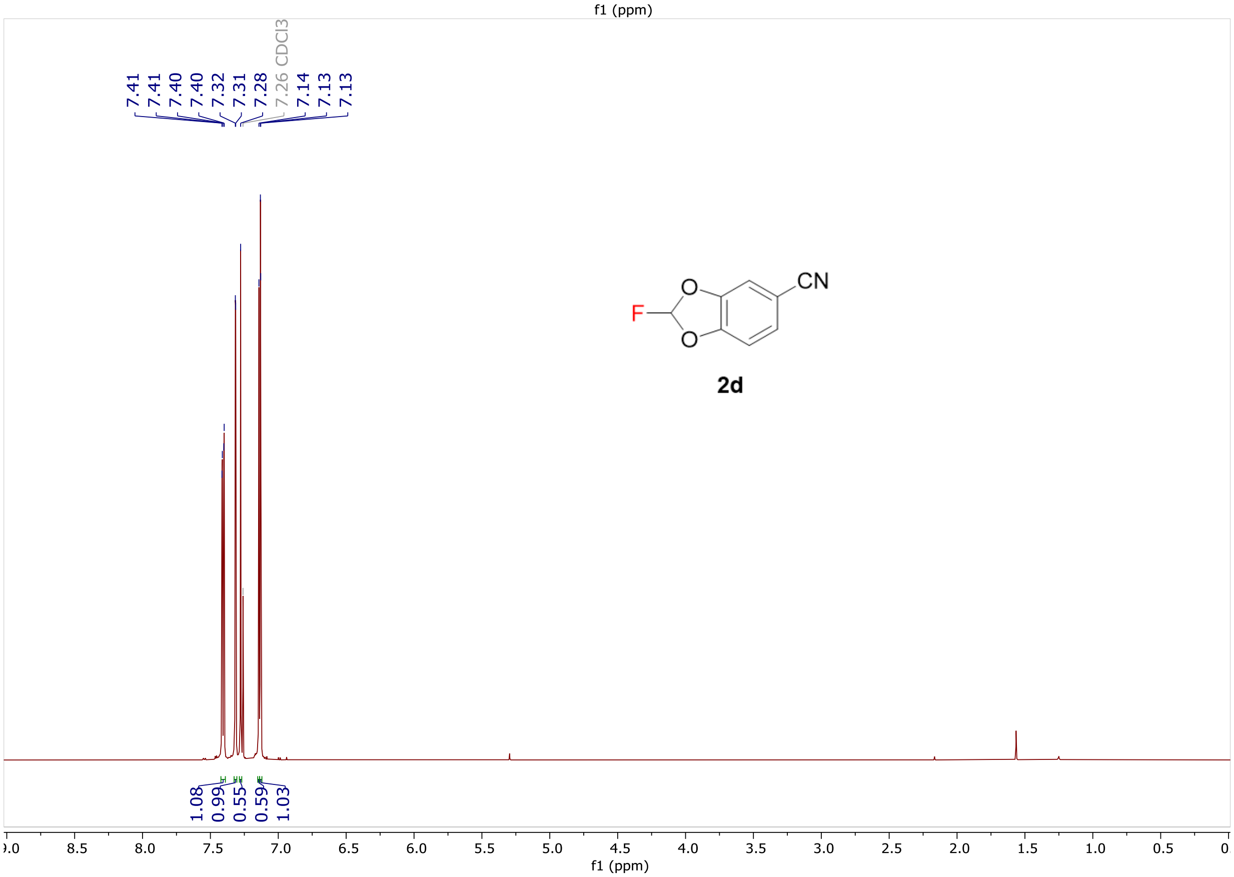
7.41
7.41
7.40
7.40
7.32
7.31
7.28
7.26 CDCl3
7.14
7.13
7.13
F
O
O
CN
2d
1.08
0.99
0.55
0.59
1.03
8.5
8.0
7.5
7.0
6.5
6.0
5.5
5.0
4.5
4.0
3.5
3.0
2.5
2.0
1.5
1.0
0.5
0
f1 (ppm)

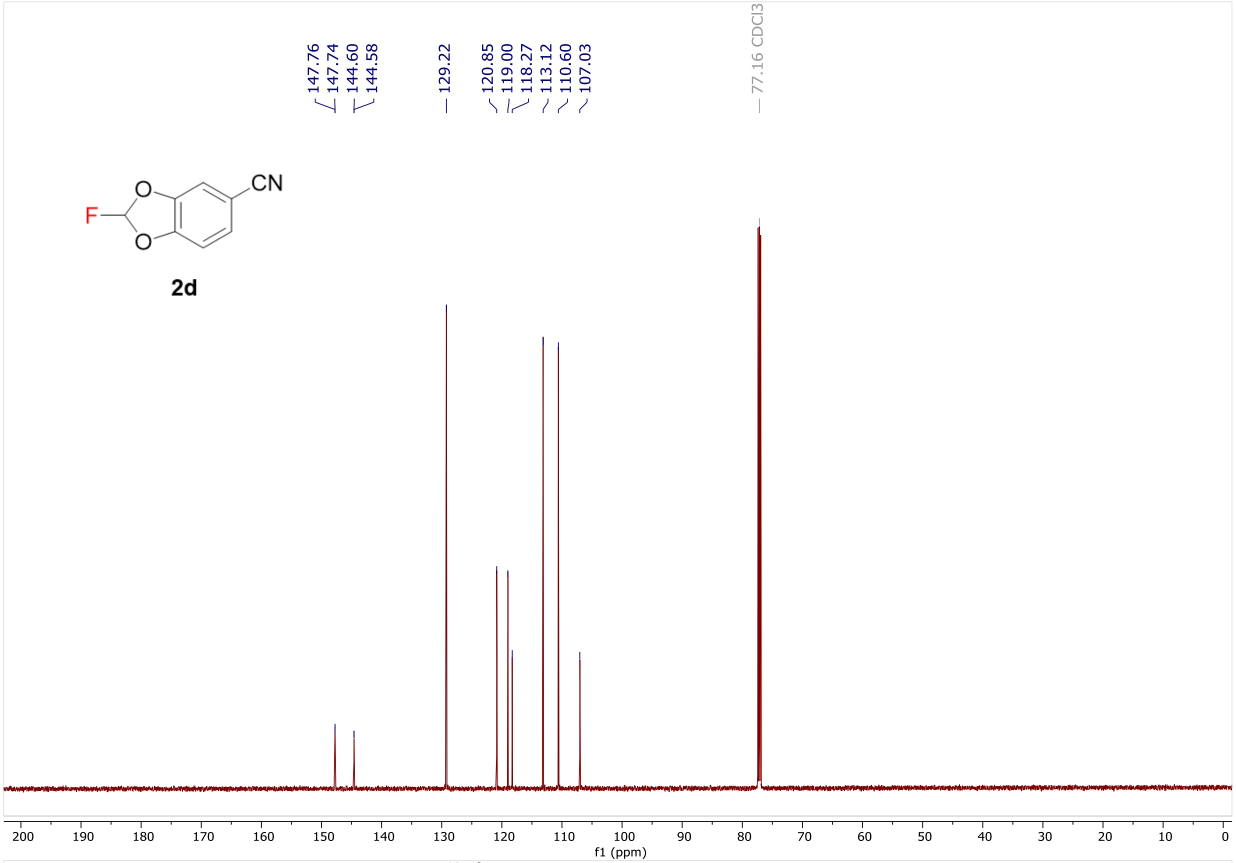

147.76
147.74
144.60
144.58
129.22
120.85
119.00
118.27
113.12
110.60
107.03
77.16 CDCl3
CN
F
O
O
2d
200
190
180
170
160
150
140
130
120
110
100
90
80
70
60
50
40
30
20
10
0
f1 (ppm)

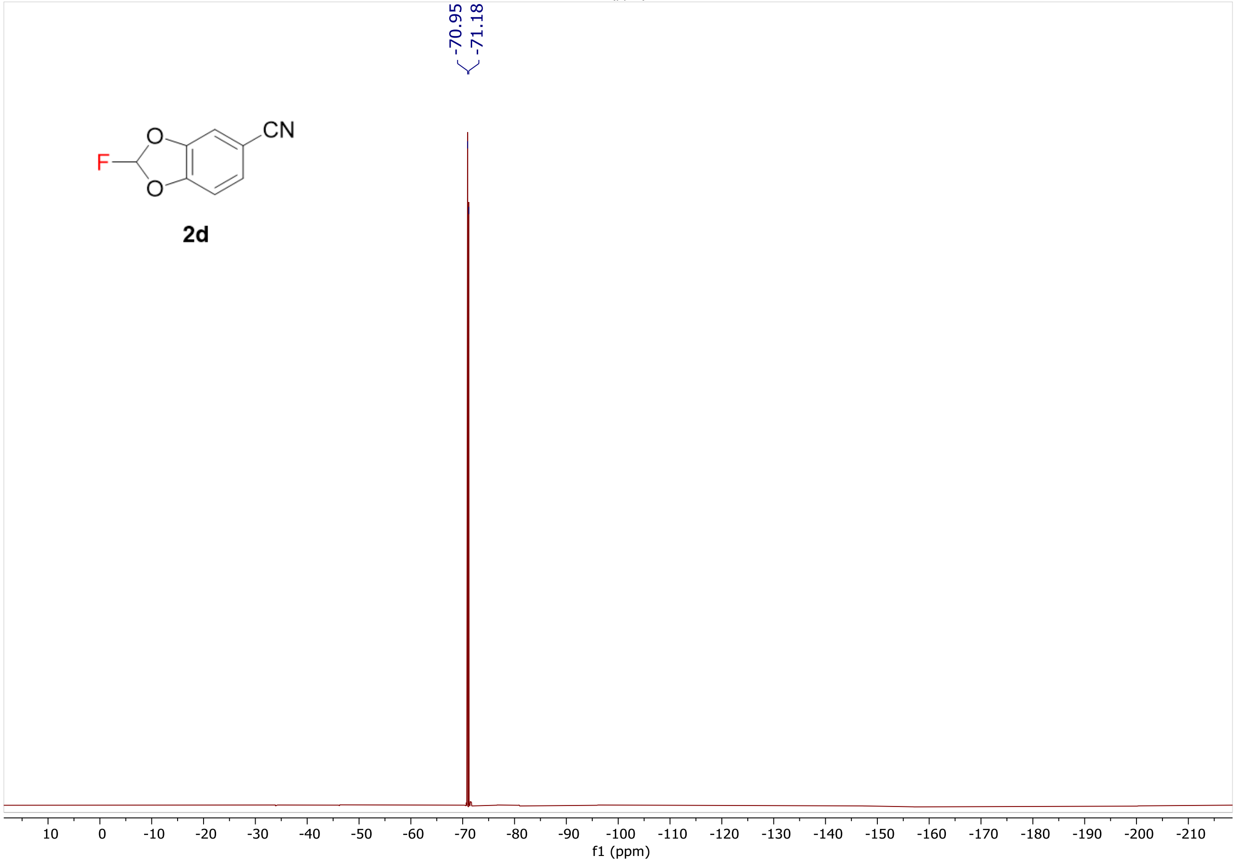

-70.95
-71.18
CN
F
O
O
2d
10
0
-10
-20
-30
-40
-50
-60
-70
-80
-90
-100
-110
-120
-130
-140
-150
-160
-170
-180
-190
-200
-210
f1 (ppm)

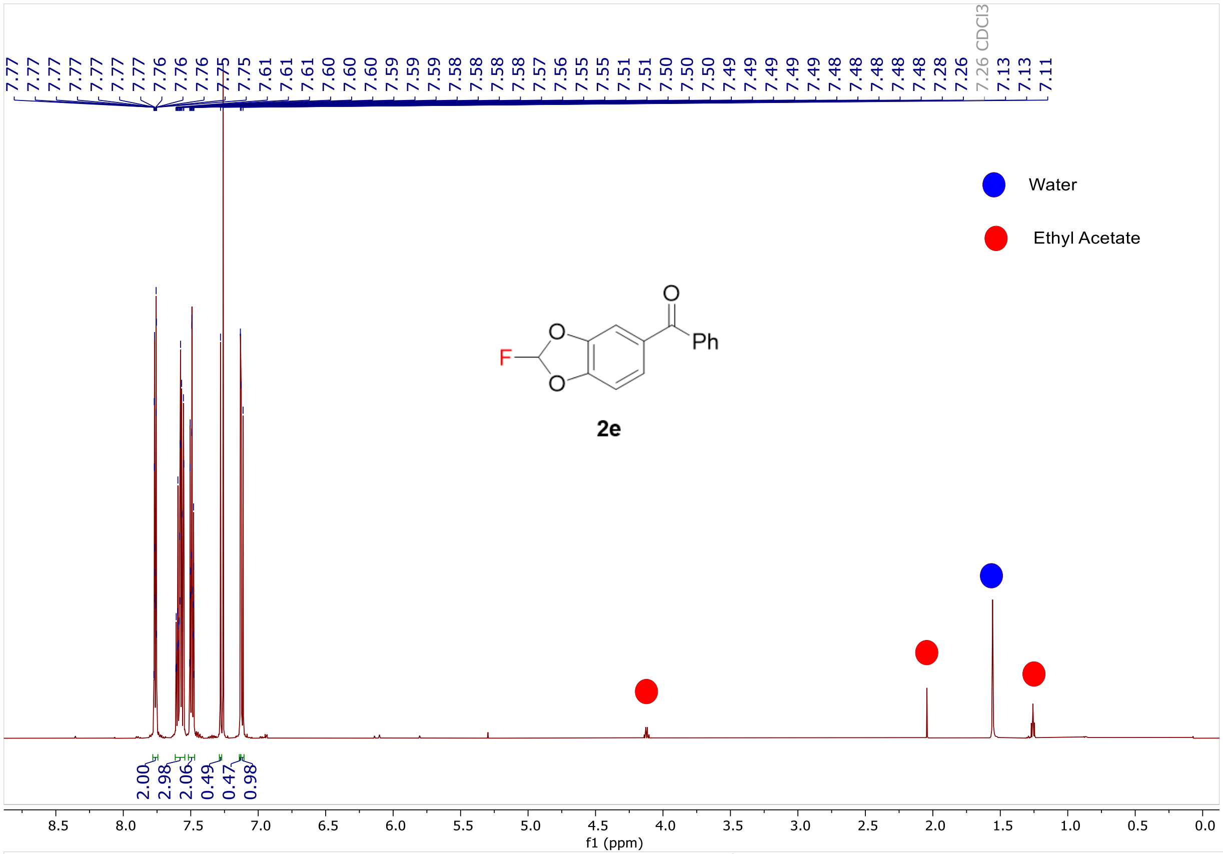

7.77
7.77
7.77
7.77
7.77
7.77
7.76
7.76
7.76
7.75
7.75
7.61
7.61
7.60
7.60
7.59
7.59
7.59
7.58
7.58
7.58
7.58
7.57
7.56
7.55
7.55
7.51
7.50
7.50
7.50
7.49
7.49
7.49
7.49
7.49
7.48
7.48
7.48
7.48
7.48
7.28
7.26
7.26 CDCl3
7.13
7.13
7.11
Water
Ethyl Acetate
O
Ph
F
O
O
2e
2.00
2.98
2.06
0.49
0.47
0.98
8.5
8.0
7.5
7.0
6.5
6.0
5.5
5.0
4.5
4.0
3.5
3.0
2.5
2.0
1.5
1.0
0.5
0.0
f1 (ppm)

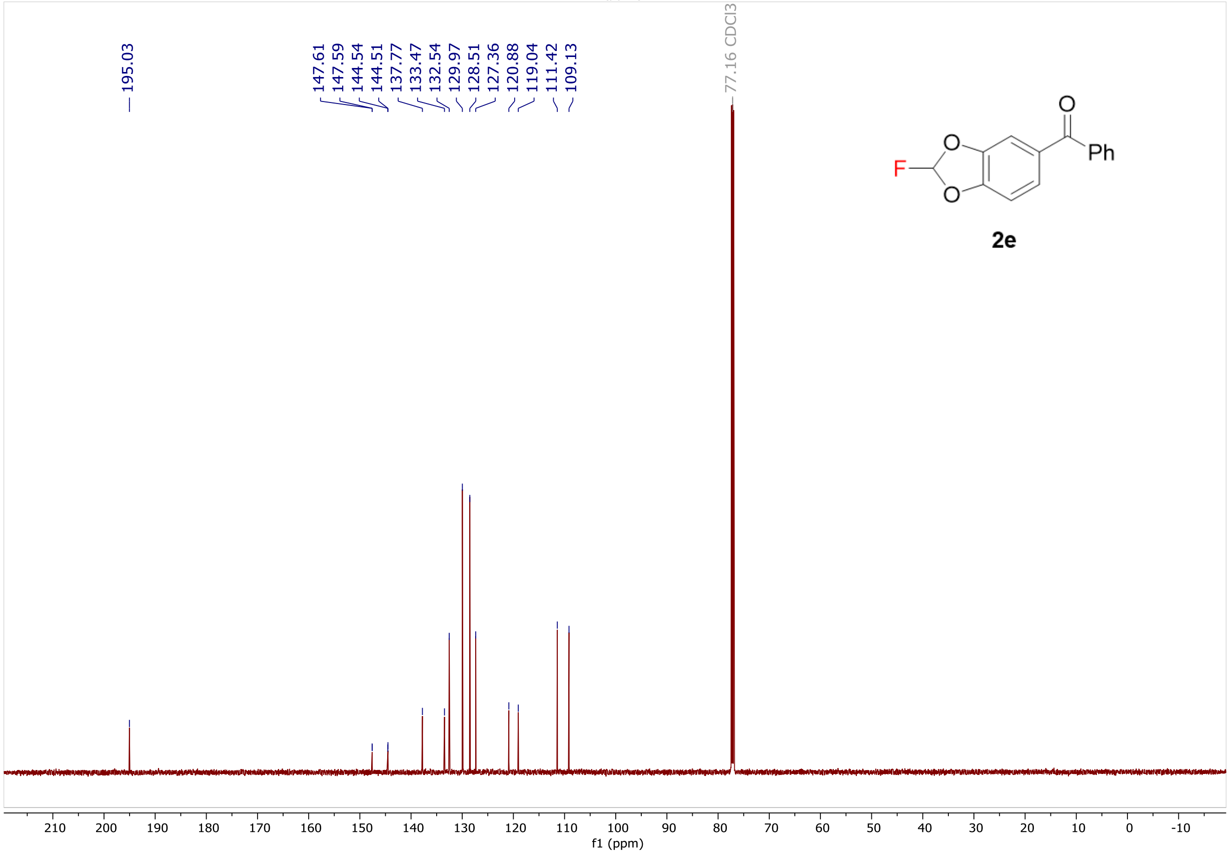

195.03
147.61
147.59
144.54
144.51
137.77
133.47
132.54
129.97
128.51
127.36
120.88
119.04
111.42
109.13
77.16 CDCl3
O
Ph
F
O
O
2e
210
200
190
180
170
160
150
140
130
120
110
100
90
80
70
60
50
40
30
20
10
0
-10
f1 (ppm)

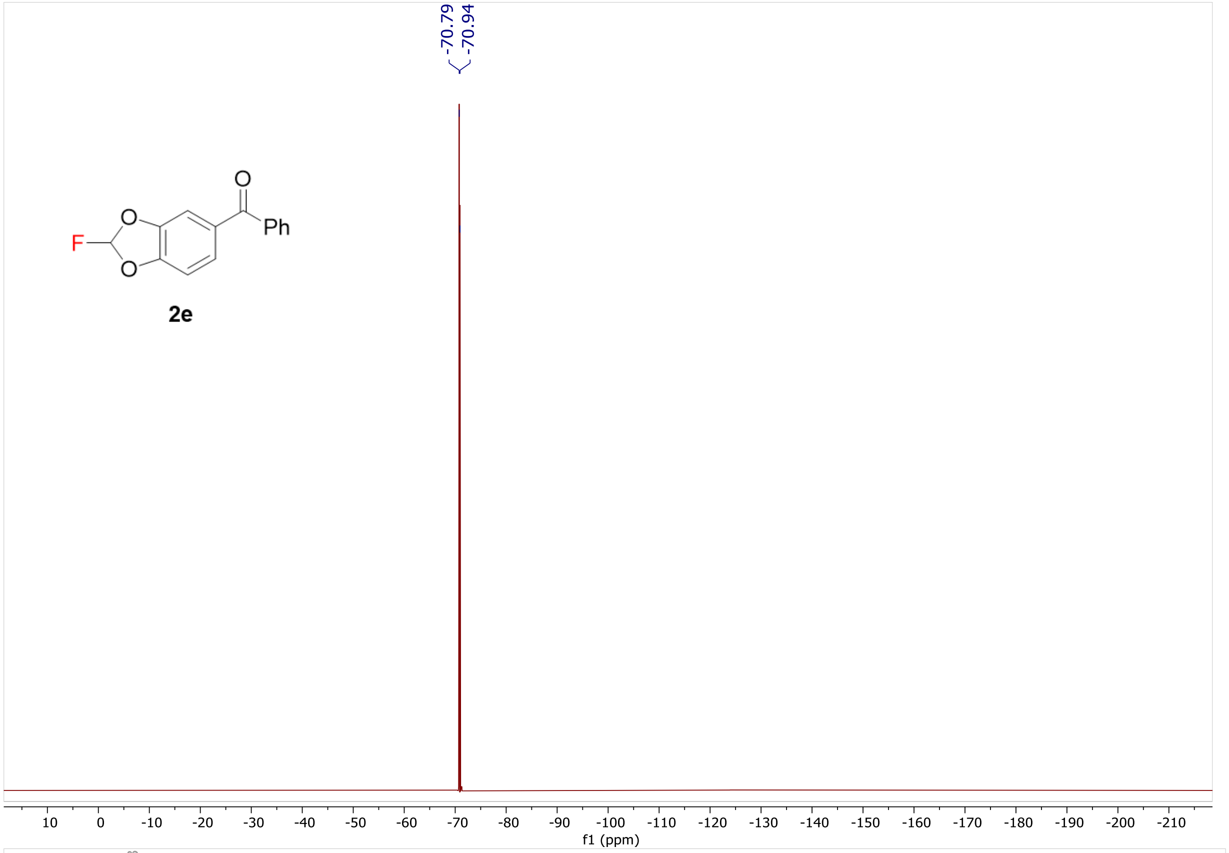
-70.79
-70.94
O
F
O
O
Ph
2e
10 0 -10 -20 -30 -40 -50 -60 -70 -80 -90 -100 -110 -120 -130 -140 -150 -160 -170 -180 -190 -200 -210
f1 (ppm)

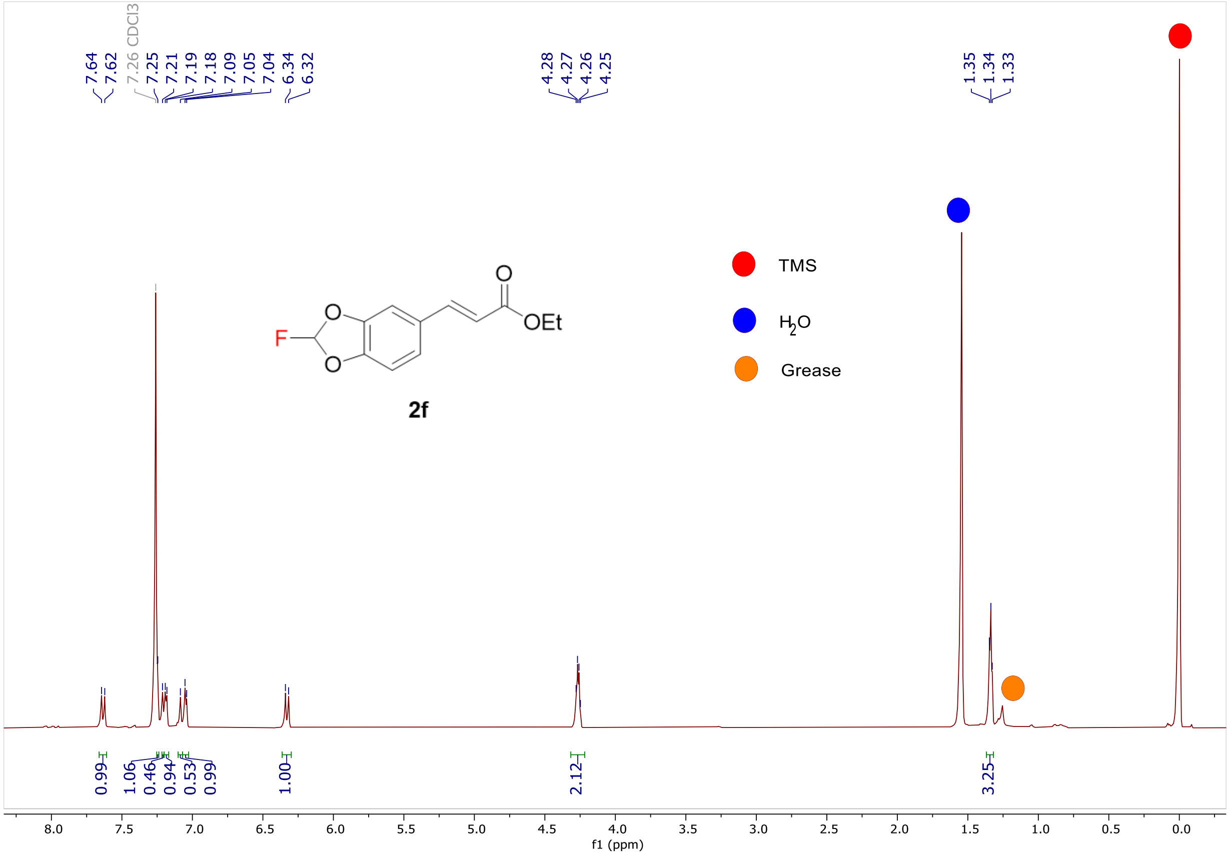
7.64
7.62
7.26 CDCl3
7.25
7.21
7.19
7.18
7.09
7.05
7.04
6.34
6.32
4.28
4.27
4.26
4.25
1.35
1.34
1.33
TMS
H2O
Grease
F
O
O
O
OEt
2f
0.99
1.06
0.46
0.94
0.53
0.99
1.00
2.12
3.25
8.0 7.5 7.0 6.5 6.0 5.5 5.0 4.5 4.0 3.5 3.0 2.5 2.0 1.5 1.0 0.5 0.0
f1 (ppm)

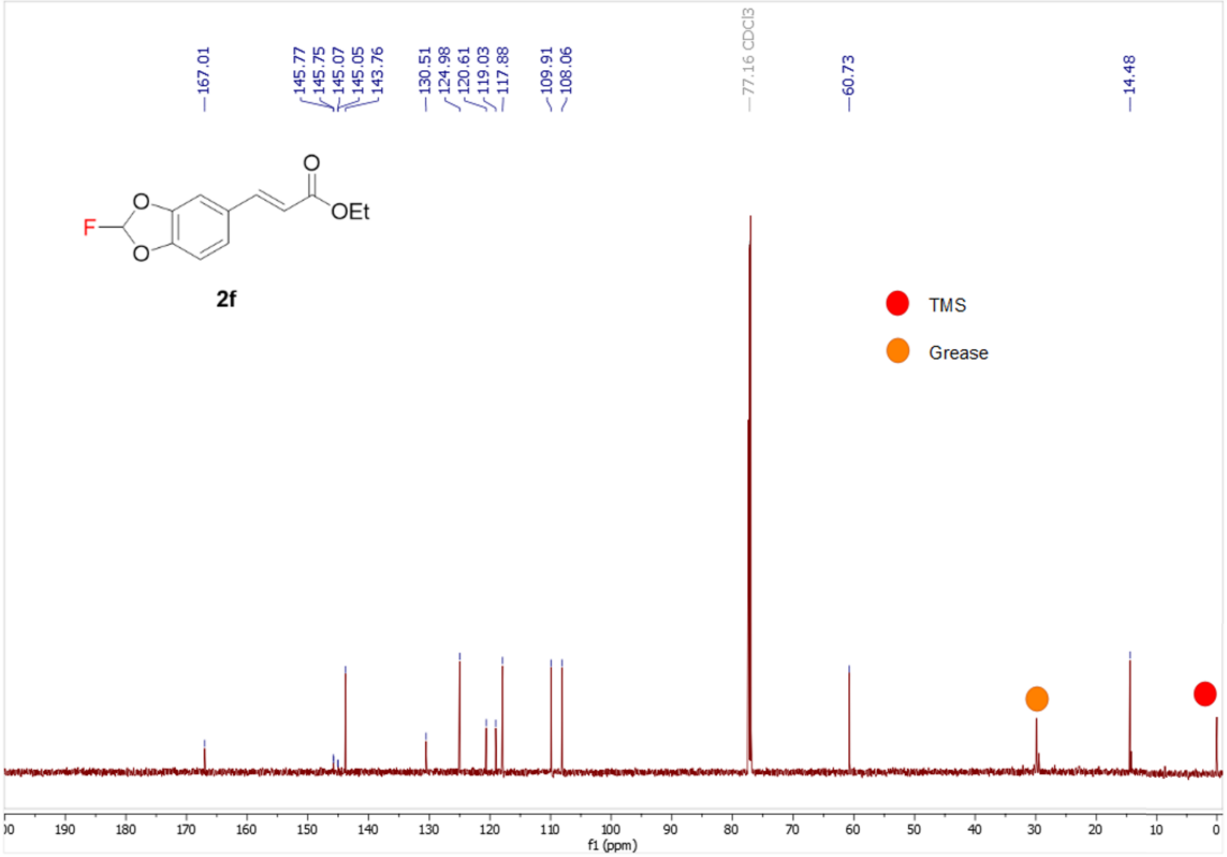

167.01
145.77
145.75
145.07
145.05
143.76
130.51
124.98
120.61
119.03
117.88
109.91
108.06
77.16 CDCl3
60.73
14.48
F
O
O
O
OEt
2f
TMS
Grease
f1 (ppm)

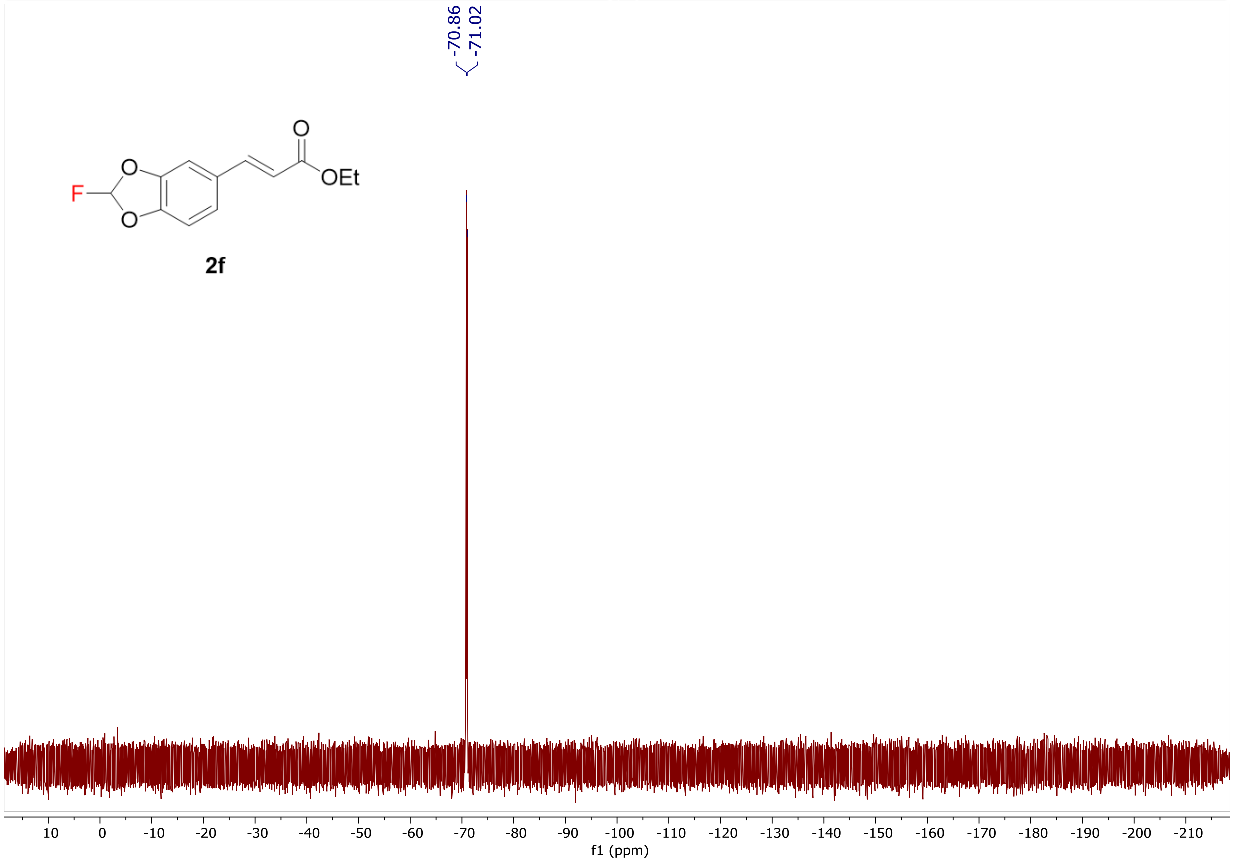

-70.86
-71.02
F
O
O
O
OEt
2f
f1 (ppm)

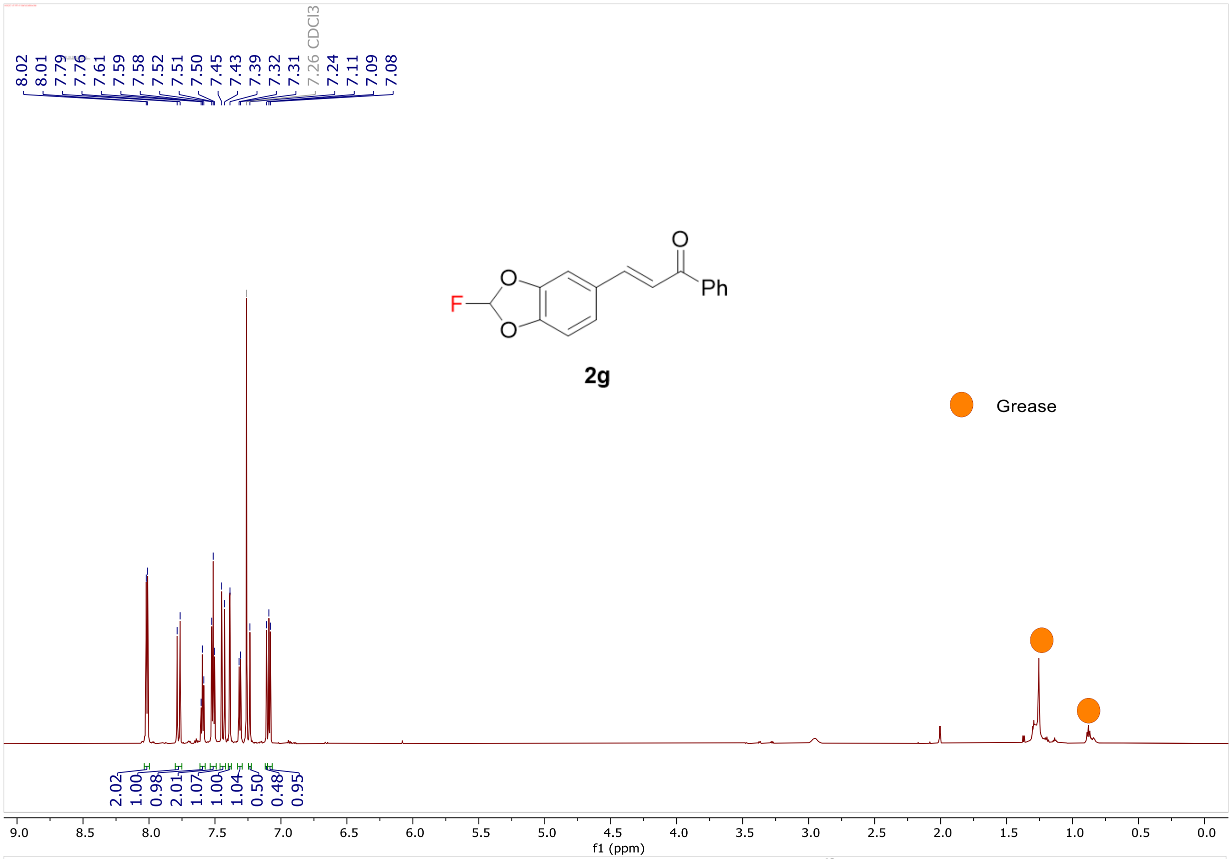

8.02
8.01
7.79
7.76
7.61
7.59
7.58
7.52
7.51
7.50
7.45
7.43
7.39
7.32
7.31
7.26 CDCl3
7.24
7.11
7.09
7.08
F
O
O
O
Ph
2g
Grease
2.02
1.00
0.98
2.01
1.07
1.00
1.04
0.50
0.48
0.95
f1 (ppm)

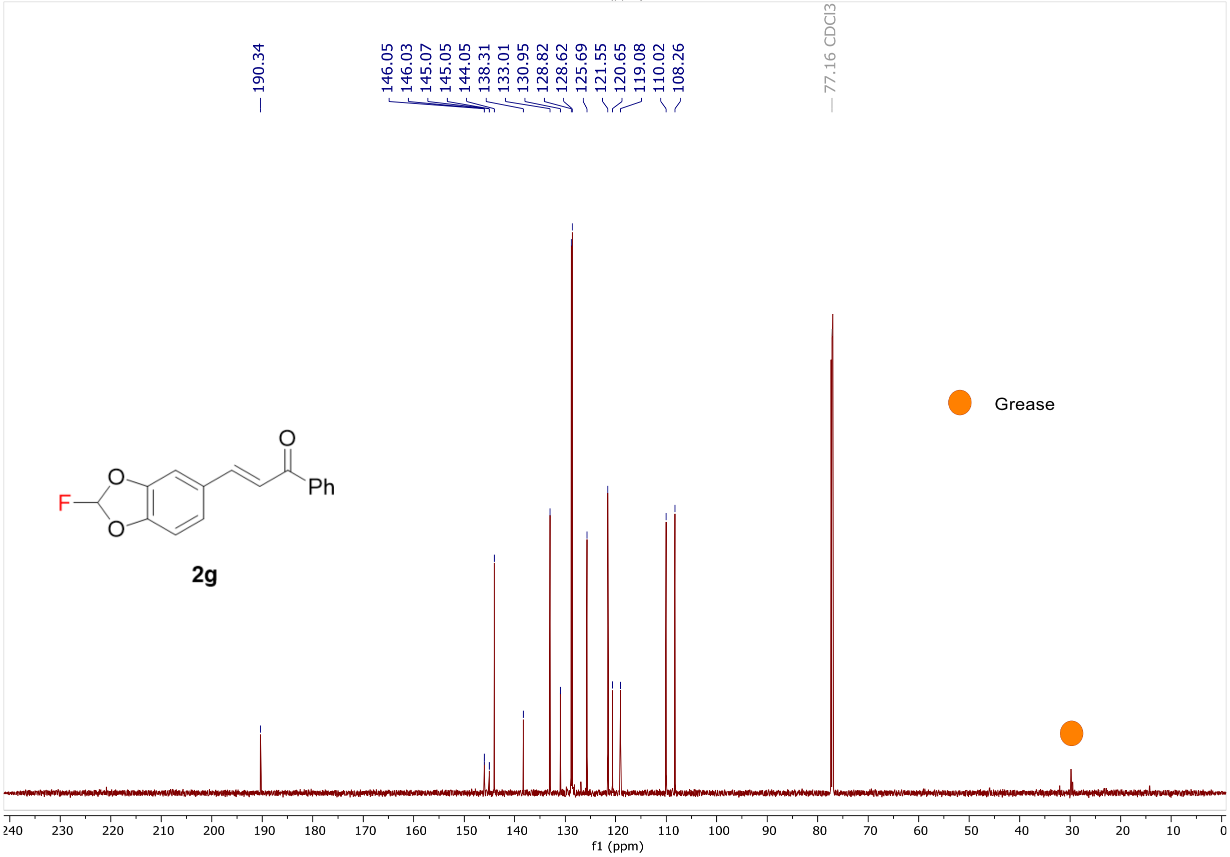

190.34
146.05
146.03
145.07
145.05
144.05
138.31
133.01
130.95
128.82
128.62
125.69
121.55
120.65
119.08
110.02
108.26
77.16 CDCl3
Grease
F
O
O
O
Ph
2g
f1 (ppm)

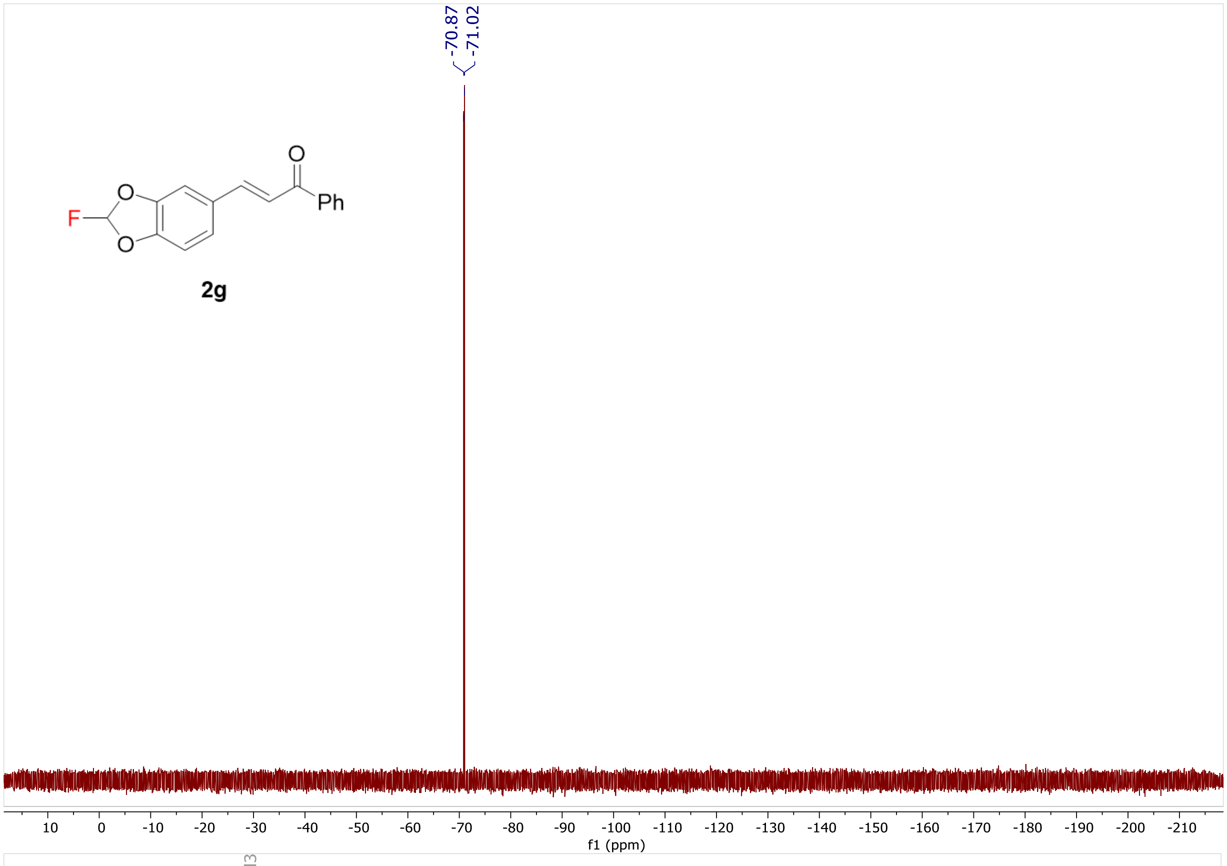
-70.87
-71.02
O
O
O
F
Ph
2g
10 0 -10 -20 -30 -40 -50 -60 -70 -80 -90 -100 -110 -120 -130 -140 -150 -160 -170 -180 -190 -200 -210
f1 (ppm)

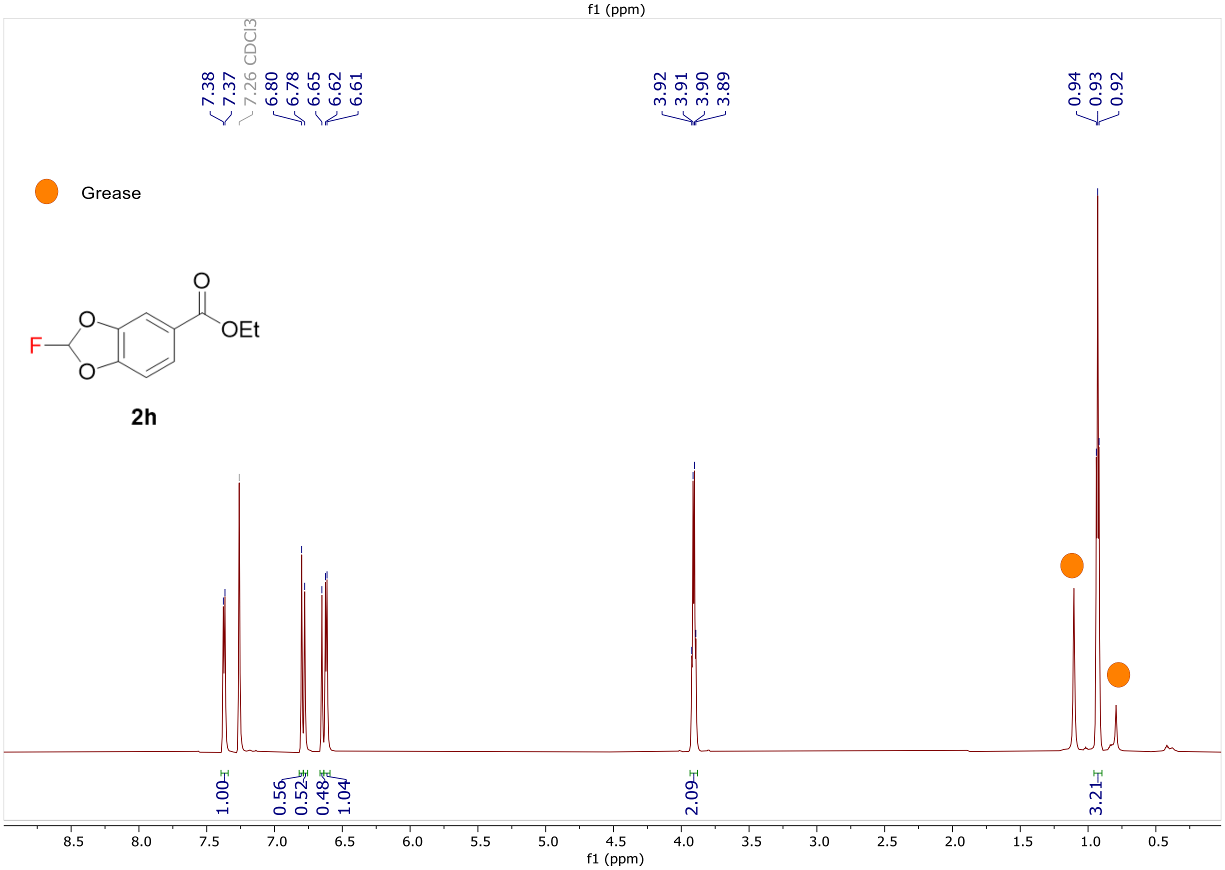
7.38
7.37
7.26 CDCl3
6.80
6.78
6.65
6.62
6.61
3.92
3.91
3.90
3.89
0.94
0.93
0.92
Grease
O
O
O
F
OEt
2h
1.00
0.56
0.52
0.48
1.04
2.09
3.21
8.5 8.0 7.5 7.0 6.5 6.0 5.5 5.0 4.5 4.0 3.5 3.0 2.5 2.0 1.5 1.0 0.5
f1 (ppm)

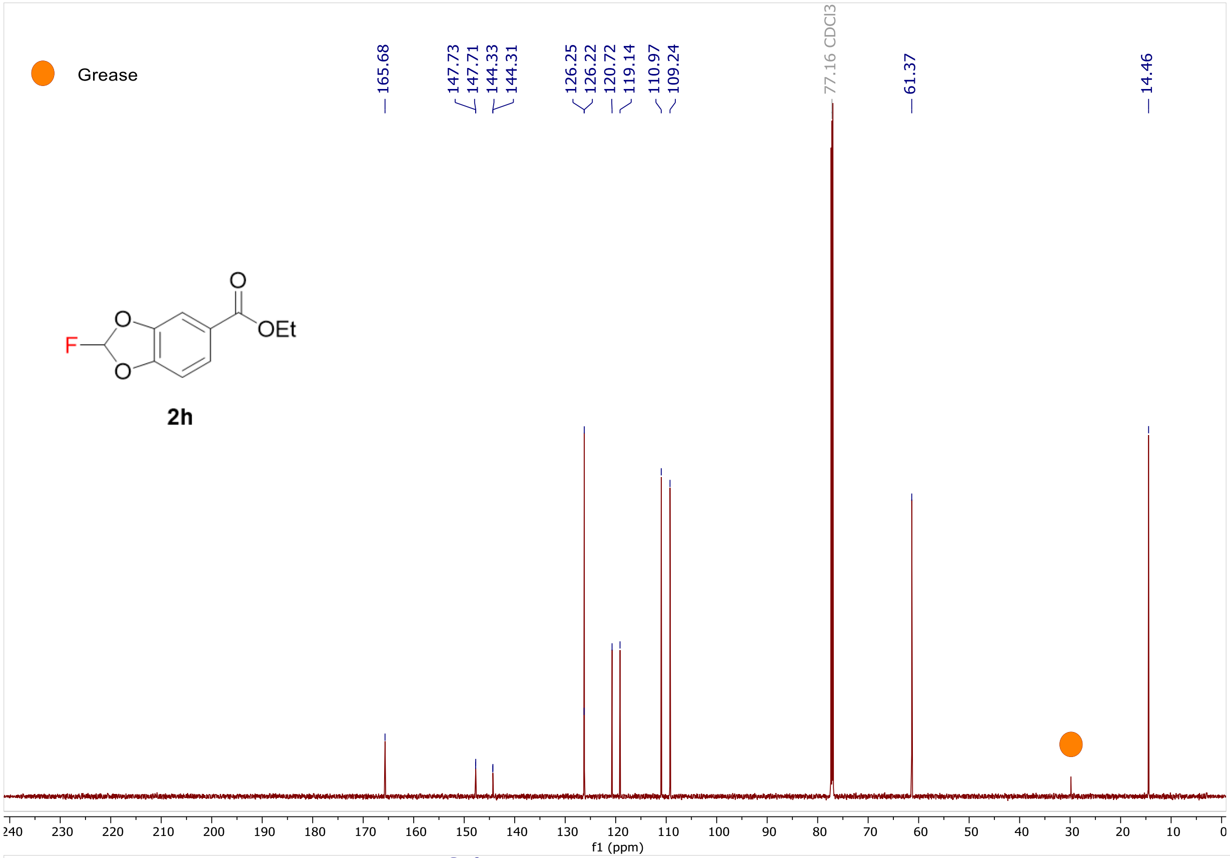
Grease
165.68
147.73
147.71
144.33
144.31
126.25
126.22
120.72
119.14
110.97
109.24
77.16 CDCl3
61.37
14.46
F
O
O
O
OEt
2h
f1 (ppm)

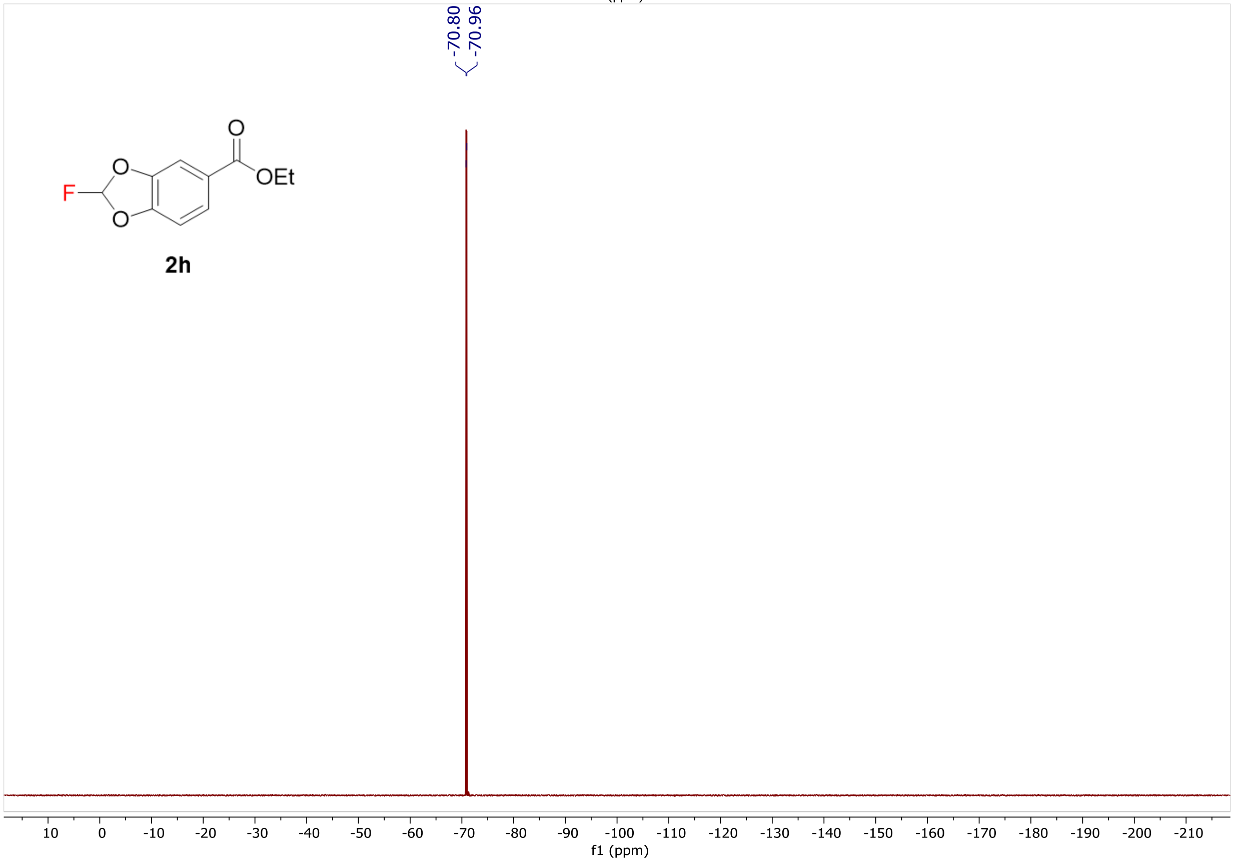
-70.80
-70.96
F
O
O
O
OEt
2h
f1 (ppm)

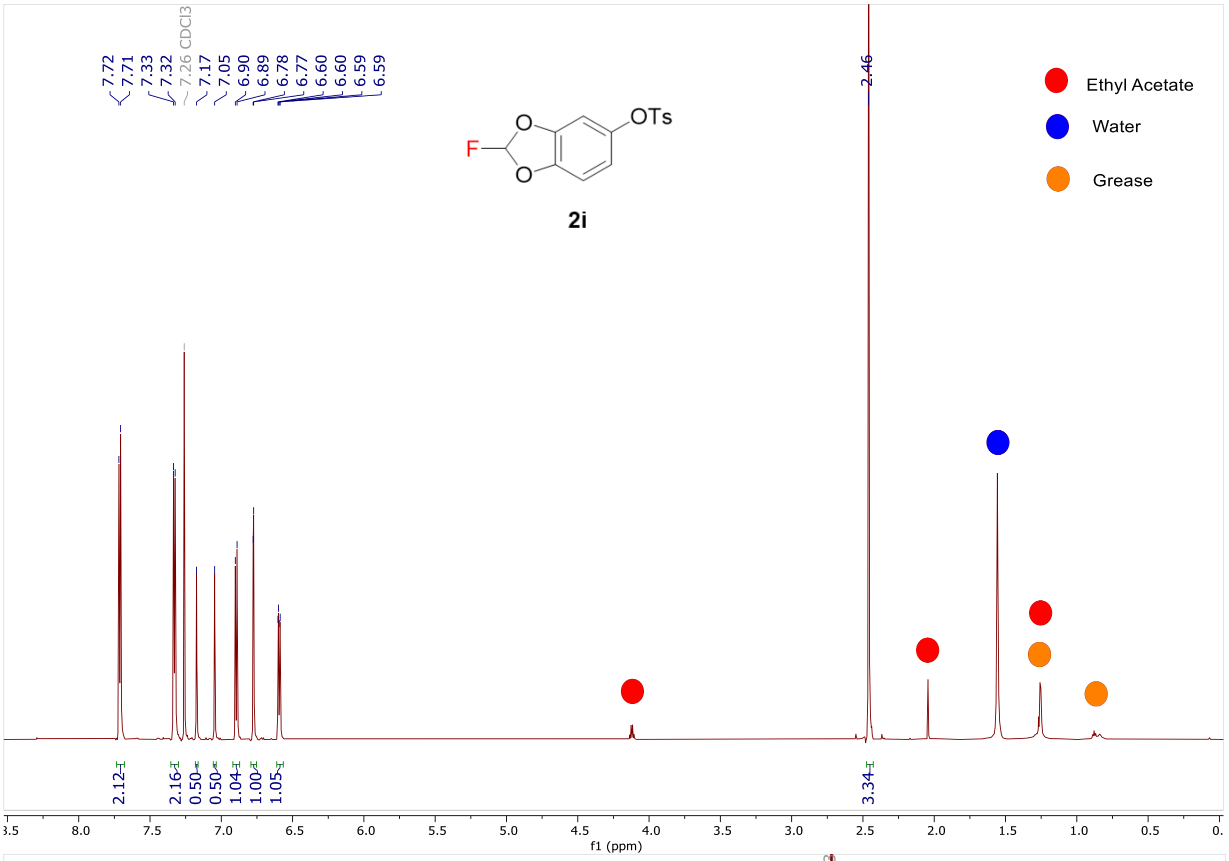
7.72
7.71
7.33
7.32
7.26 CDCl3
7.17
7.05
6.90
6.89
6.78
6.77
6.60
6.60
6.59
6.59
2.46
OTs
F
O
O
2i
Ethyl Acetate
Water
Grease
2.12
2.16
0.50
0.50
1.04
1.00
1.05
3.34
f1 (ppm)

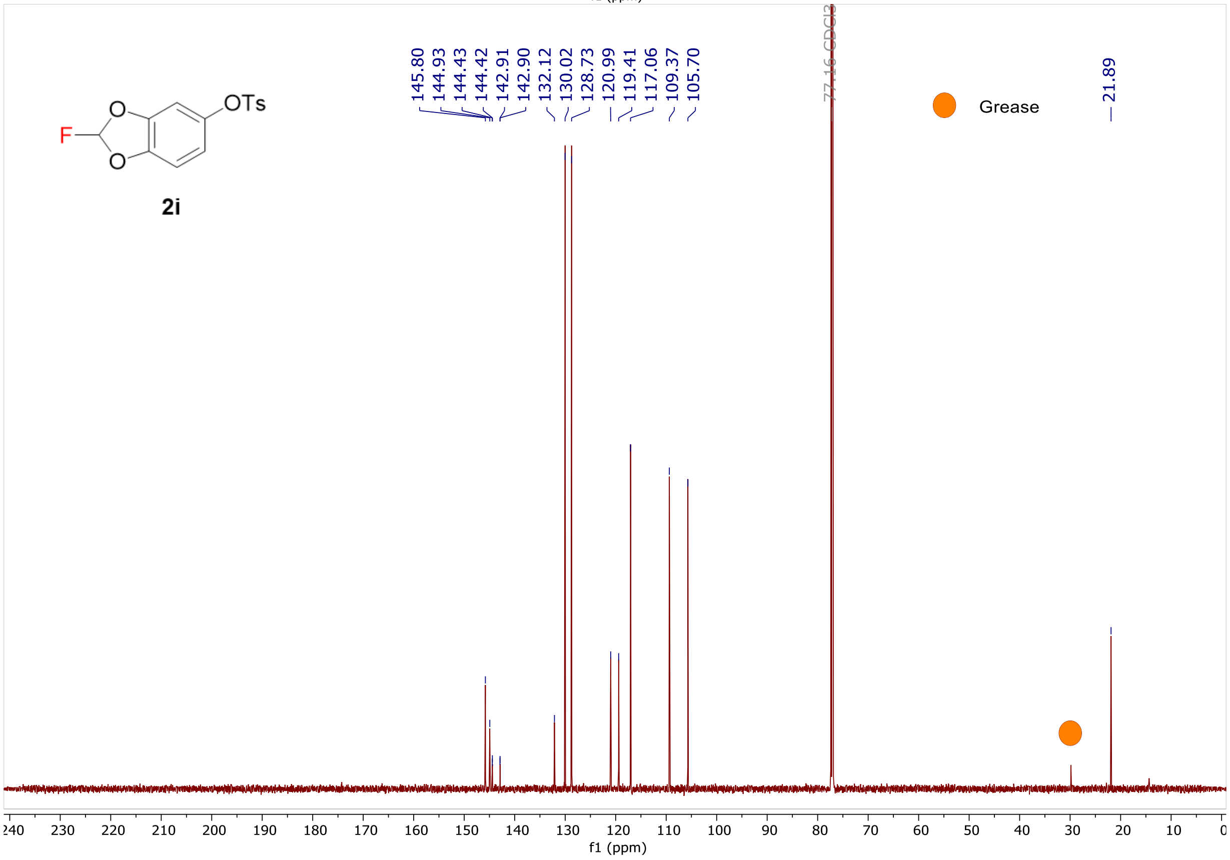
145.80
144.93
144.43
144.42
142.91
142.90
132.12
130.02
128.73
120.99
119.41
117.06
109.37
105.70
77.16 CDCl3
21.89
OTs
F
O
O
2i
Grease
f1 (ppm)

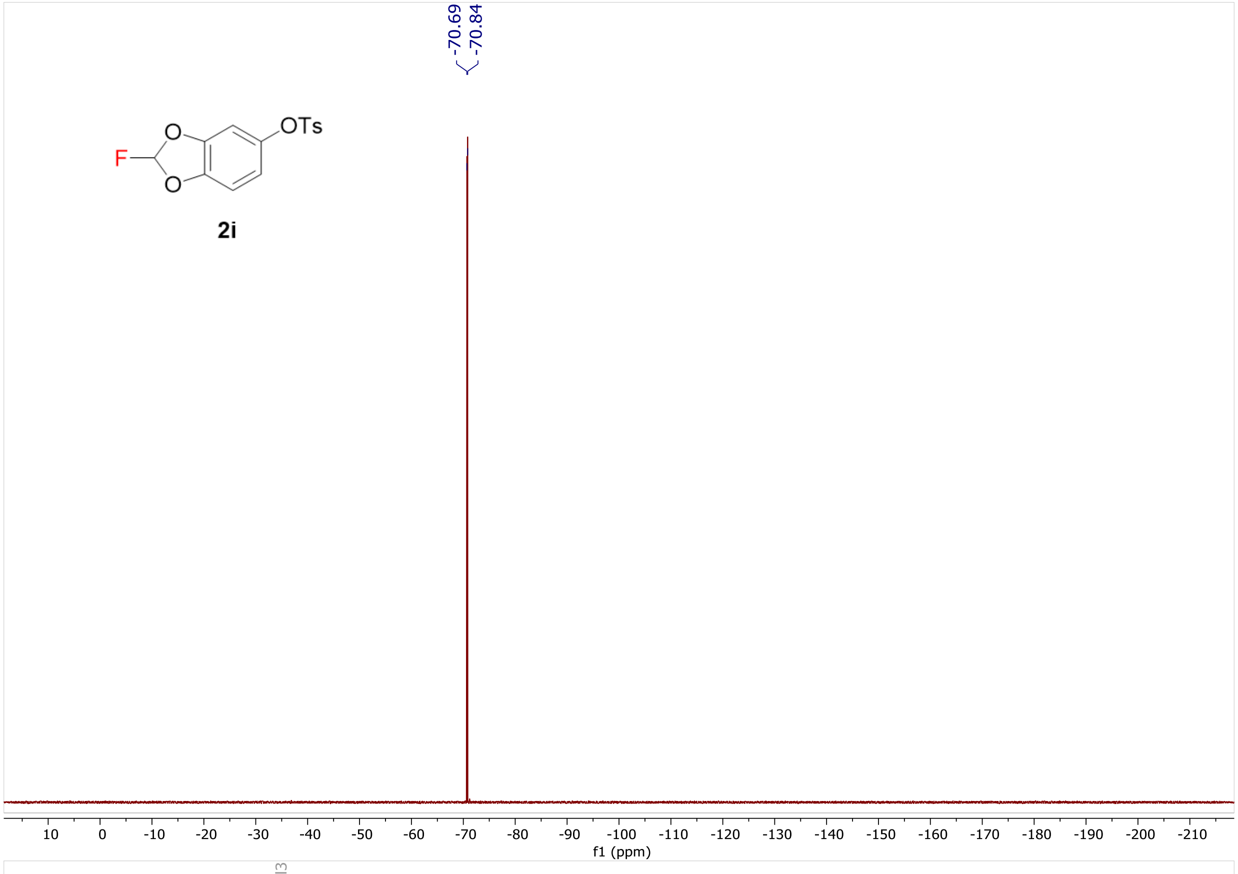

-70.69
-70.84
OTs
F
O
O
2i
f1 (ppm)

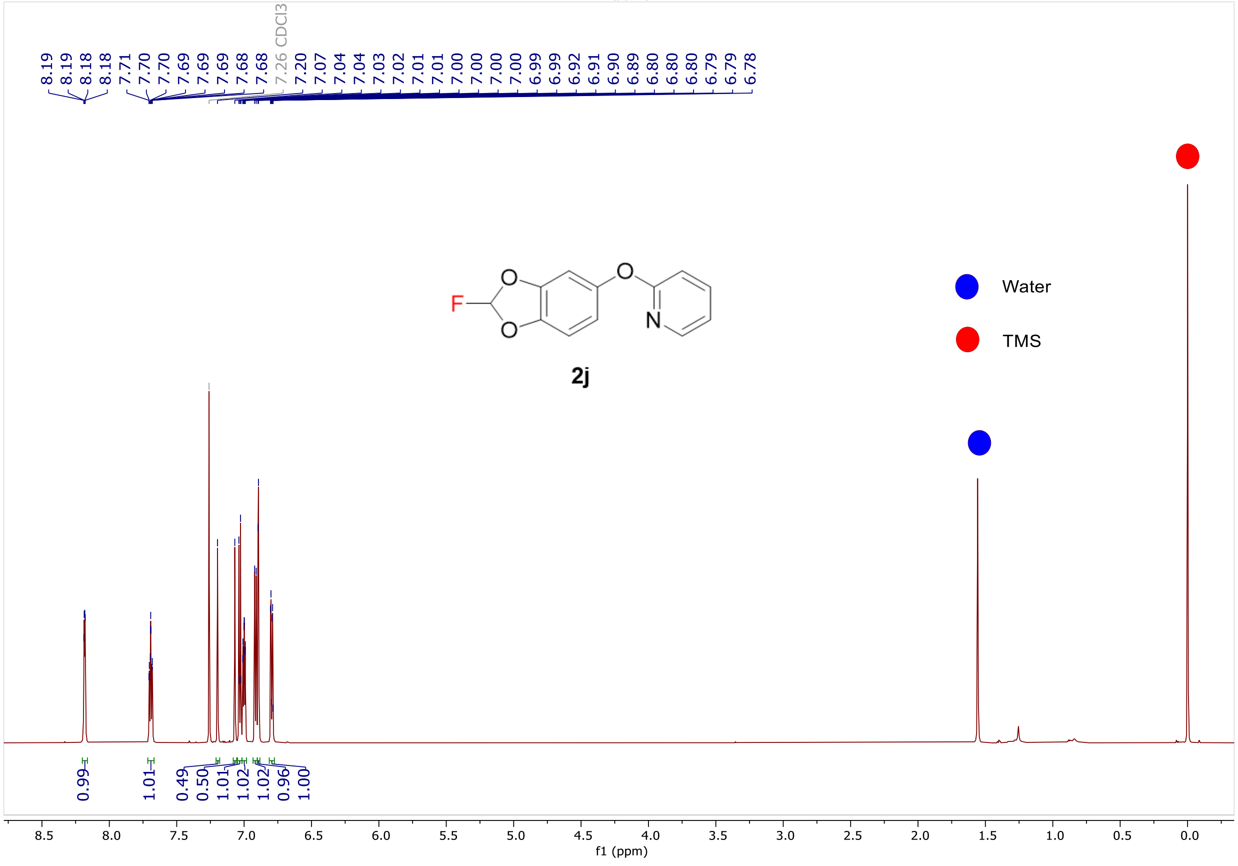

8.19
8.19
8.18
8.18
7.71
7.70
7.70
7.69
7.69
7.69
7.68
7.68
7.26 CDCl3
7.20
7.07
7.04
7.04
7.03
7.02
7.01
7.01
7.00
7.00
7.00
7.00
6.99
6.99
6.92
6.91
6.90
6.89
6.80
6.80
6.80
6.79
6.79
6.78
F
O
O
N
2j
Water
TMS
0.99
1.01
0.49
0.50
1.01
1.02
1.02
0.96
1.00
f1 (ppm)

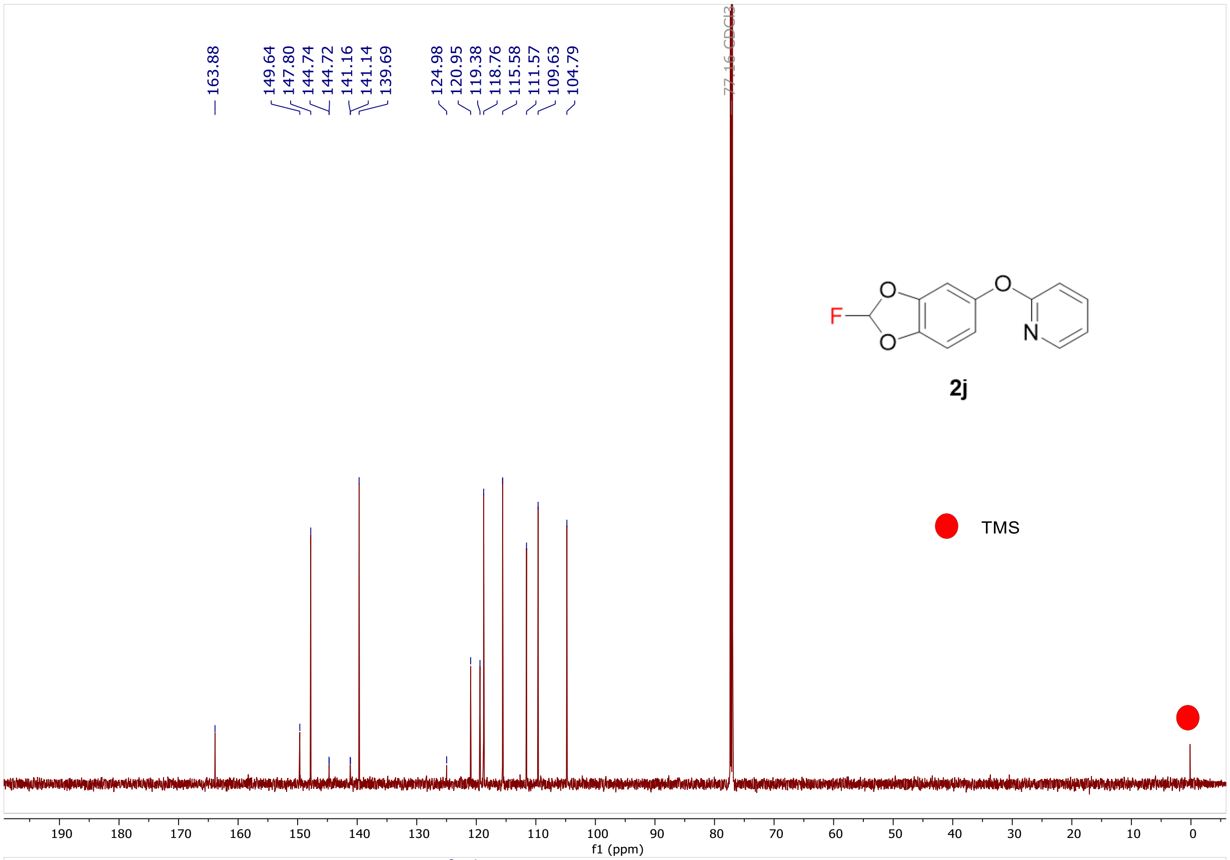
163.88
149.64
147.80
144.74
144.72
141.16
141.14
139.69
124.98
120.95
119.38
118.76
115.58
111.57
109.63
104.79
77.16 CDCl3
F
O
O
O
N
2j
TMS
190 180 170 160 150 140 130 120 110 100 90 80 70 60 50 40 30 20 10 0
f1 (ppm)

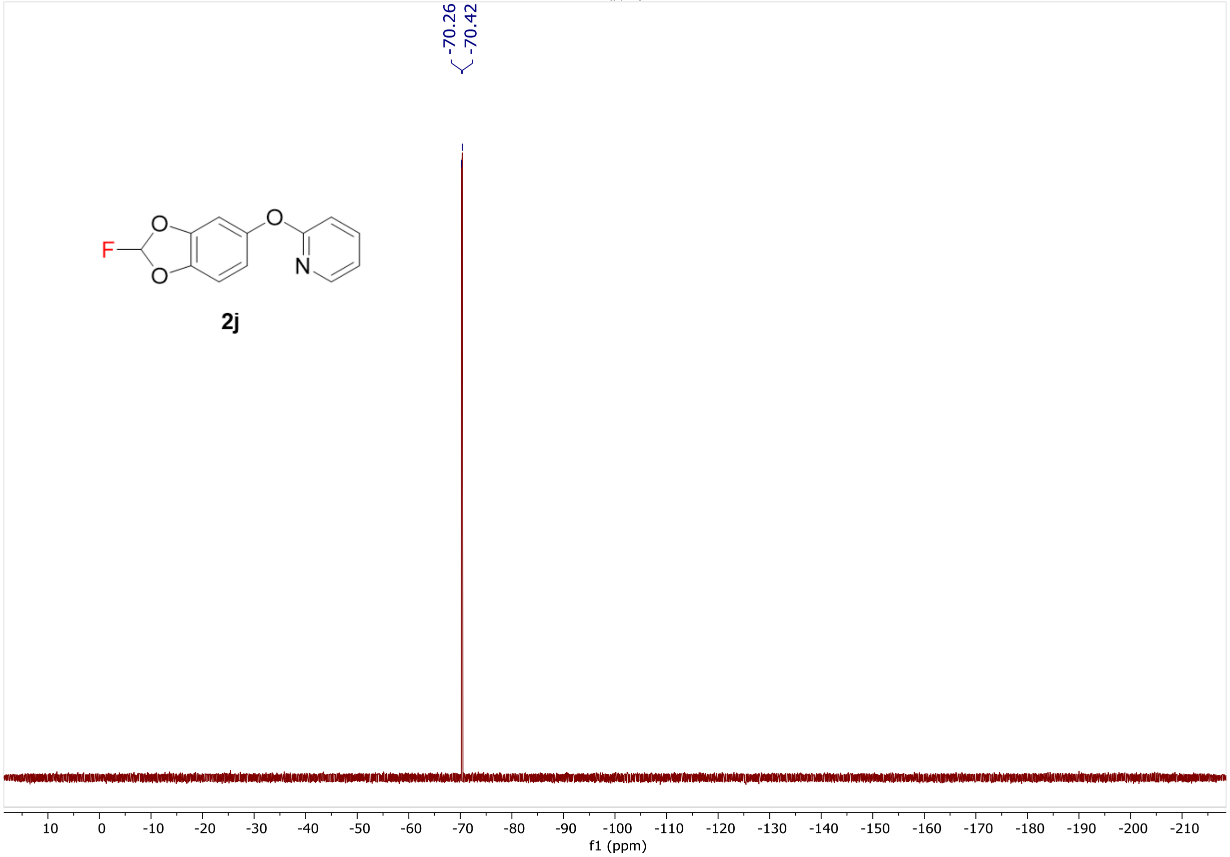
-70.26
-70.42
F
O
O
O
N
2j
10 0 -10 -20 -30 -40 -50 -60 -70 -80 -90 -100 -110 -120 -130 -140 -150 -160 -170 -180 -190 -200 -210
f1 (ppm)

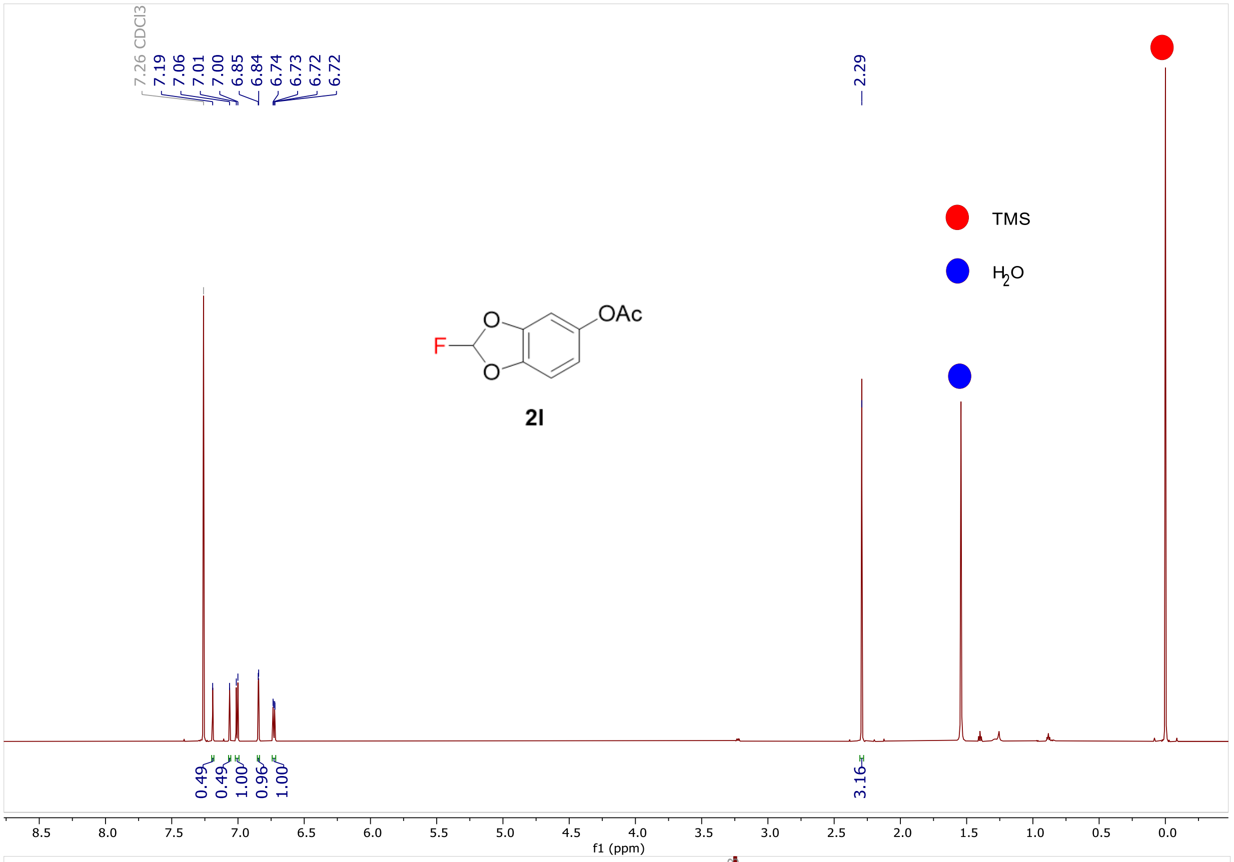
7.26 CDCl3
7.19
7.06
7.01
7.00
6.85
6.84
6.74
6.73
6.72
6.72
2.29
TMS
H2O
OAc
F
O
O
2l
0.49
0.49
1.00
0.96
1.00
3.16
f1 (ppm)

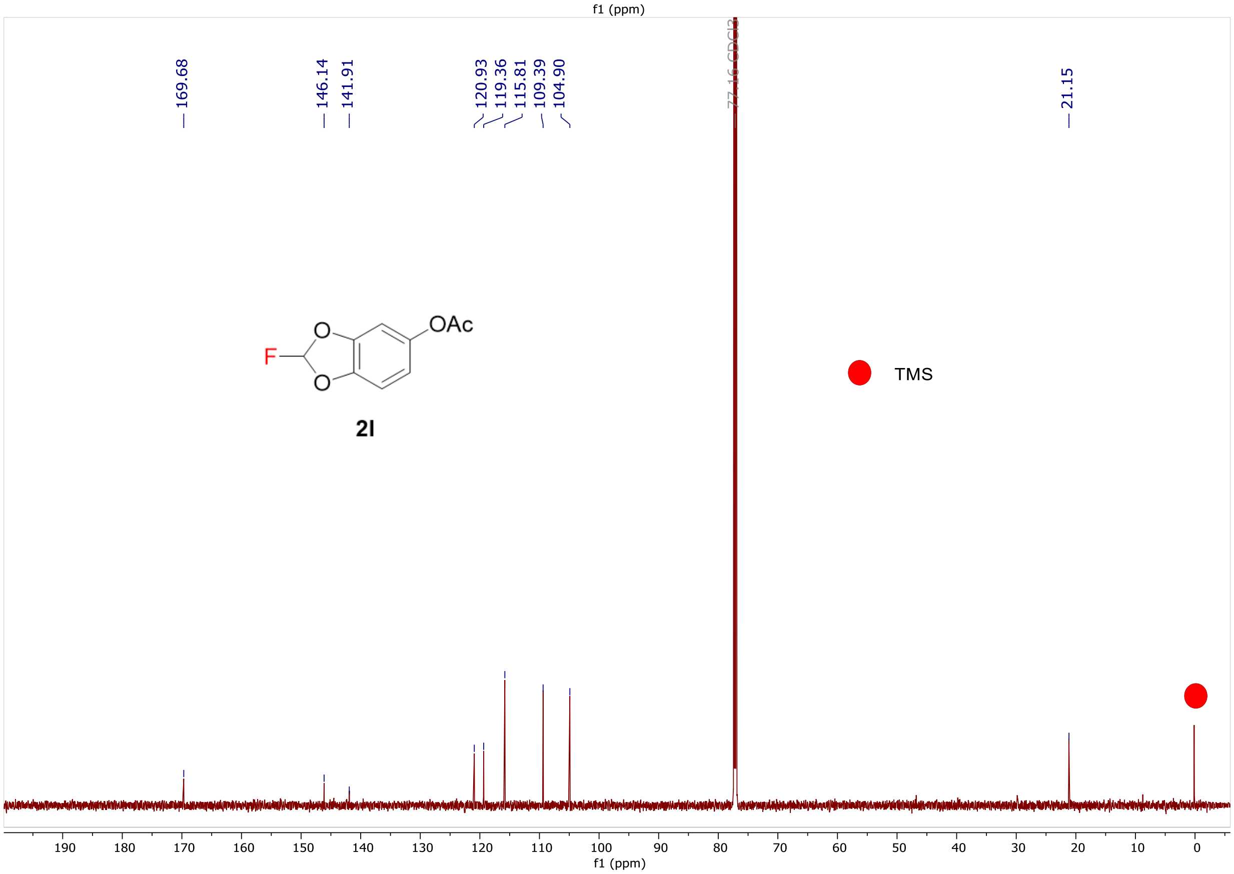
169.68
146.14
141.91
120.93
119.36
115.81
109.39
104.90
77.16 CDCl3
21.15
OAc
F
O
O
2l
TMS
f1 (ppm)

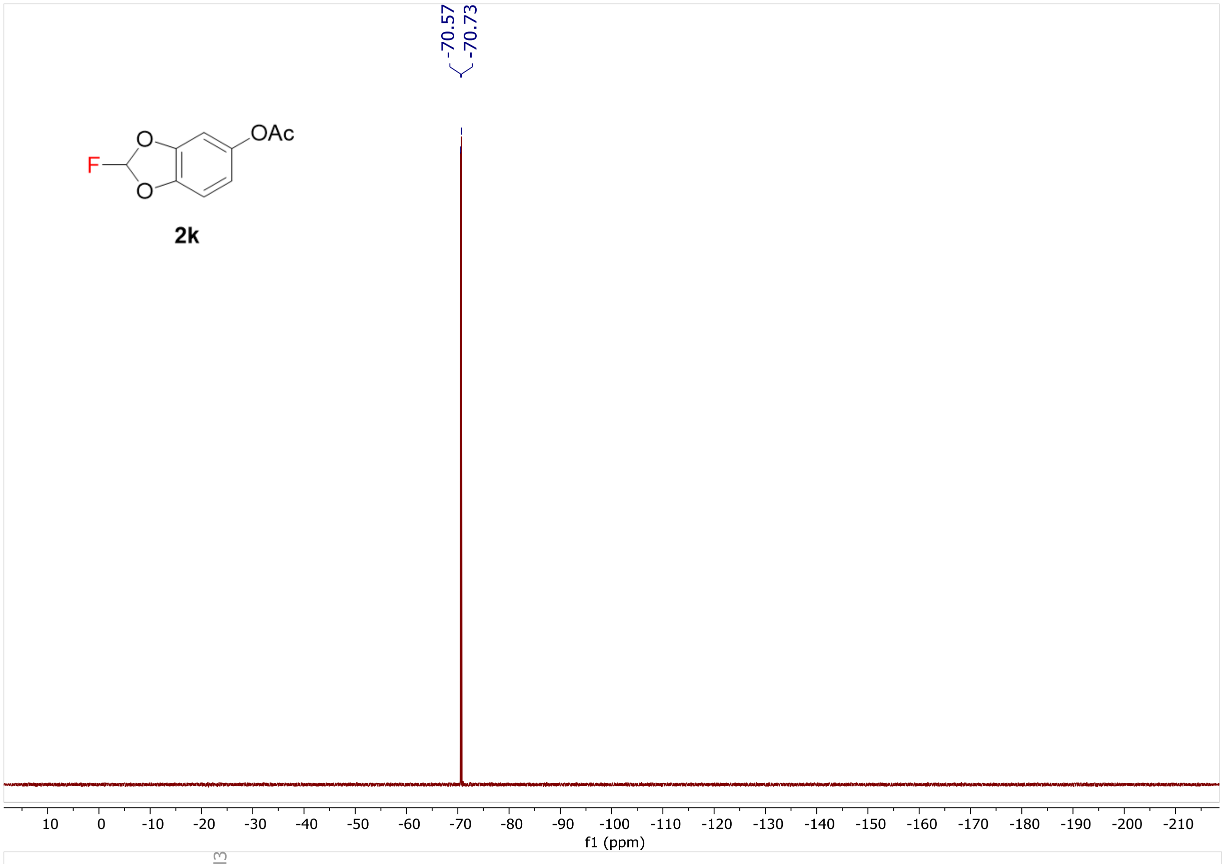
-70.57
-70.73
OAc
F
O
O
2k
10 0 -10 -20 -30 -40 -50 -60 -70 -80 -90 -100 -110 -120 -130 -140 -150 -160 -170 -180 -190 -200 -210
f1 (ppm)

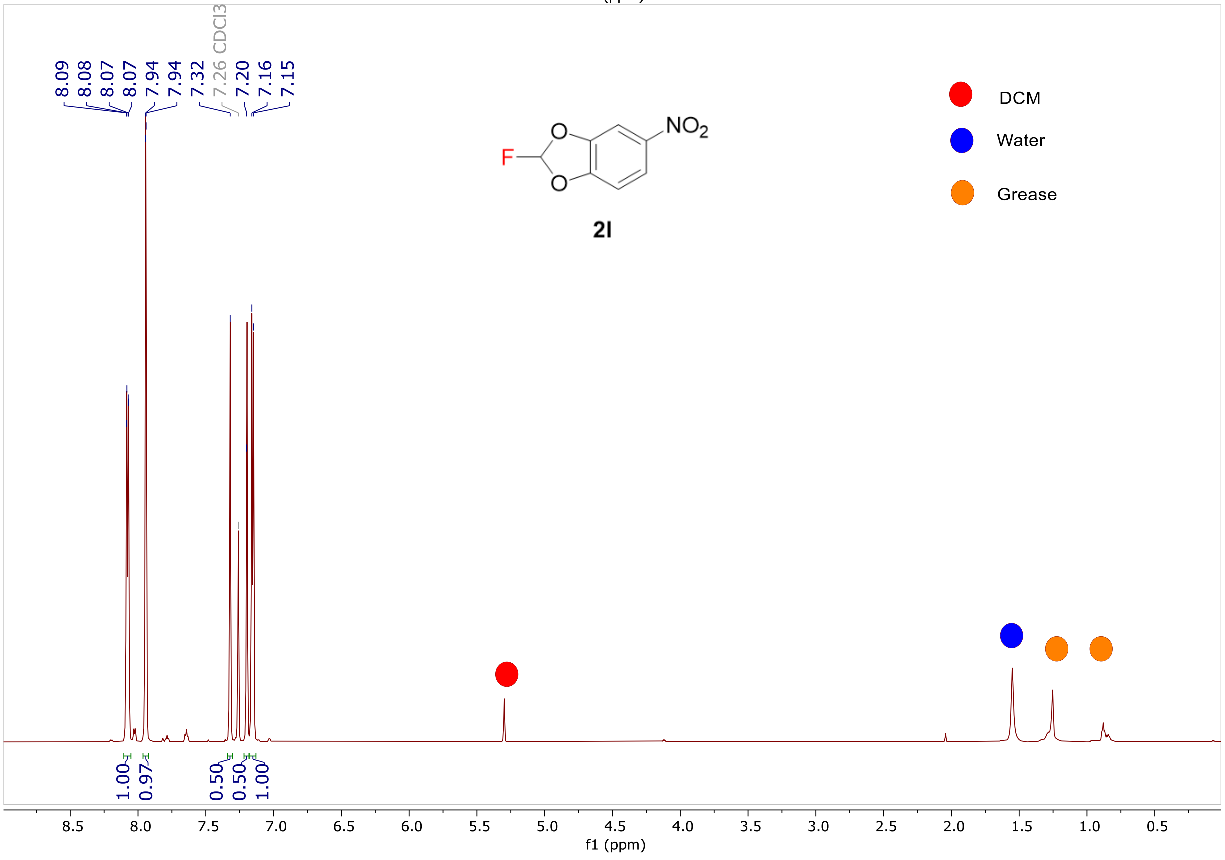
8.09
8.08
8.07
8.07
7.94
7.94
7.32
7.26 CDCl3
7.20
7.16
7.15
NO2
F
O
O
2l
DCM
Water
Grease
1.00
0.97
0.50
0.50
1.00
8.5 8.0 7.5 7.0 6.5 6.0 5.5 5.0 4.5 4.0 3.5 3.0 2.5 2.0 1.5 1.0 0.5
f1 (ppm)

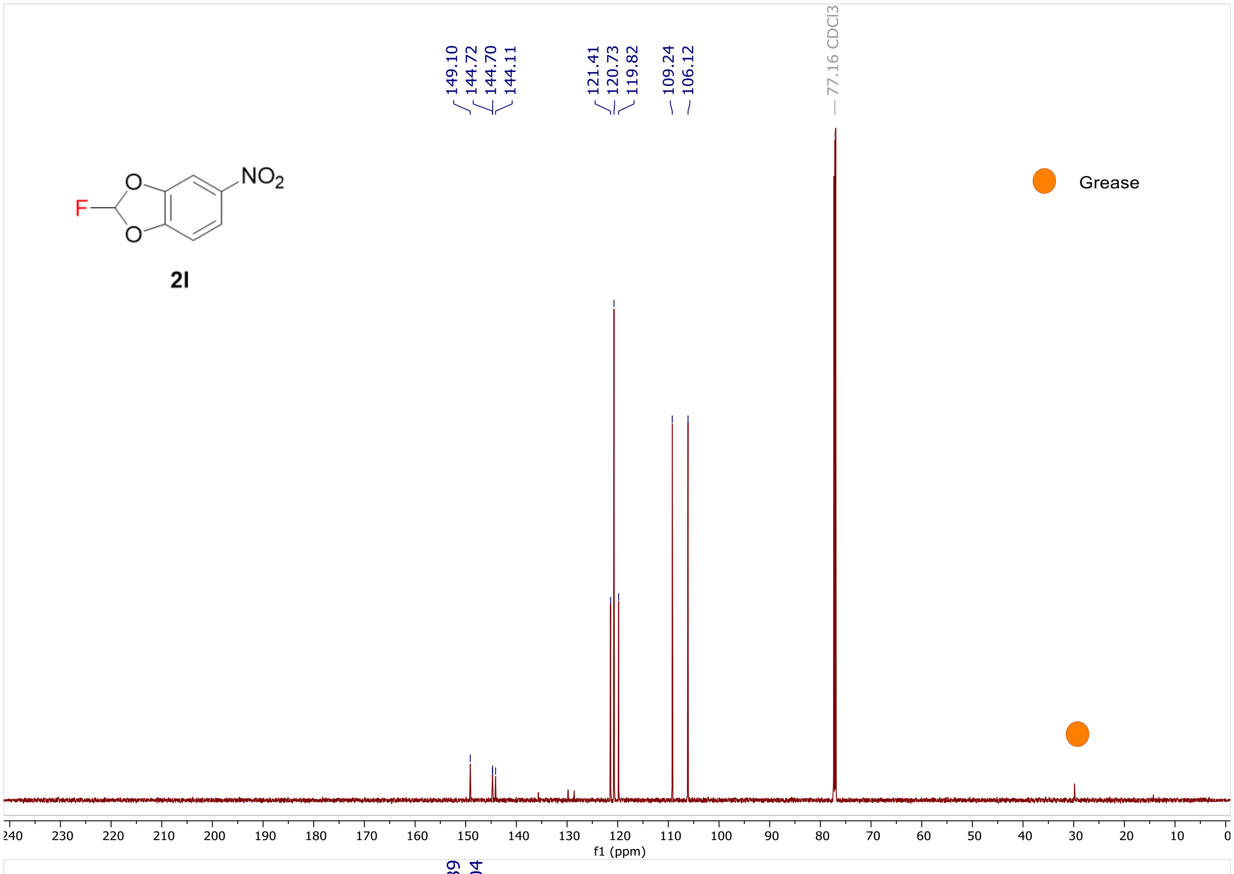
149.10
144.72
144.70
144.11
121.41
120.73
119.82
109.24
106.12
77.16 CDCl3
NO2
F
O
O
2l
Grease
f1 (ppm)

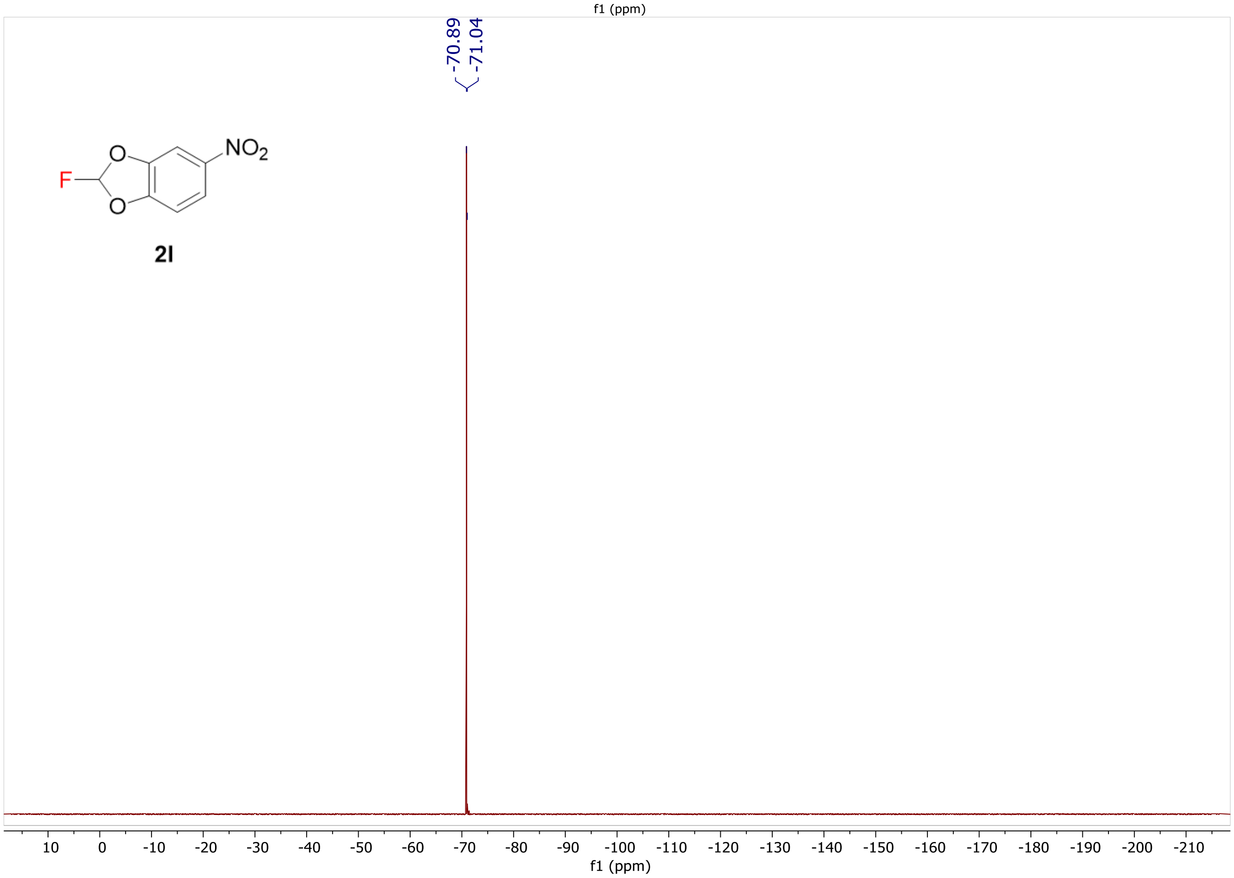
-70.89
-71.04
NO2
F
O
O
2l
f1 (ppm)

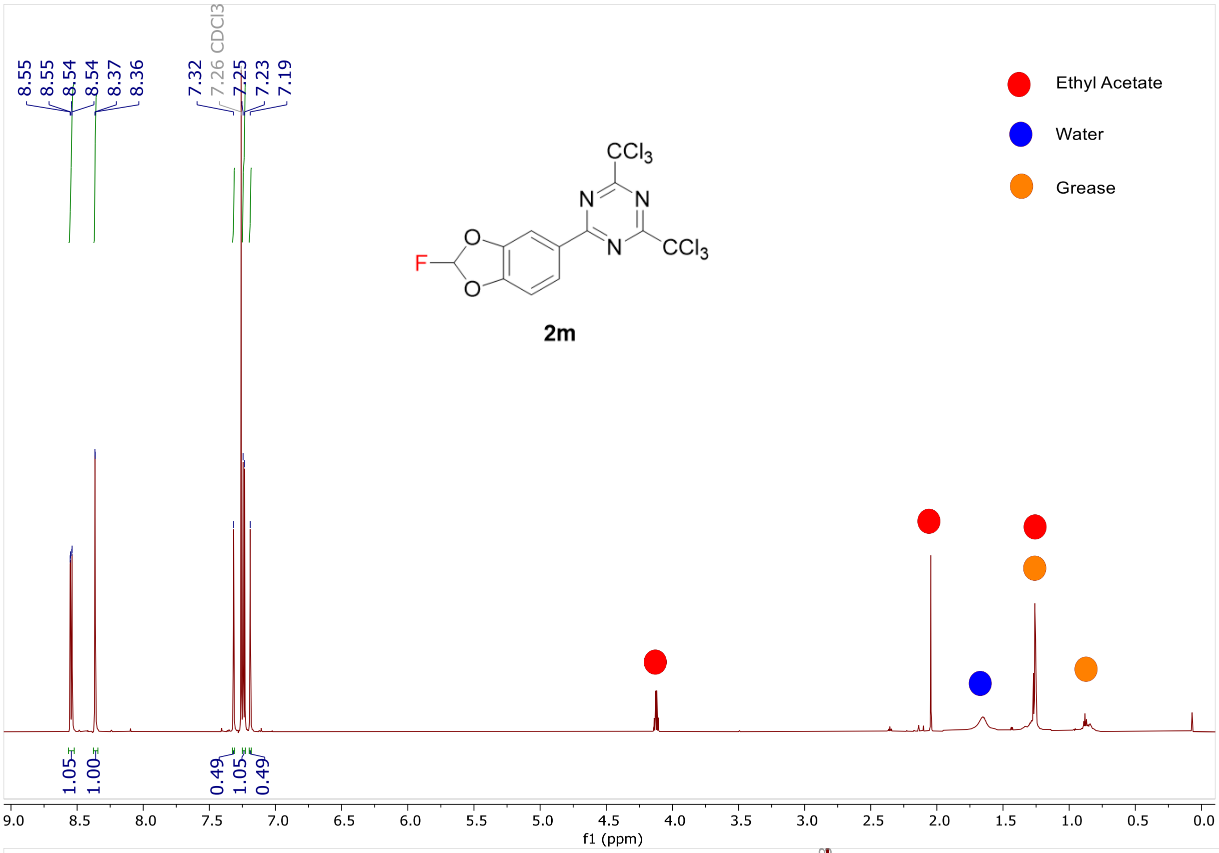
8.55
8.55
8.54
8.54
8.37
8.36
7.32
7.26 CDCl3
7.25
7.23
7.19
Ethyl Acetate
Water
Grease
CCl3
N
N
N
CCl3
O
O
F
2m
1.05
1.00
0.49
1.05
0.49
9.0
8.5
8.0
7.5
7.0
6.5
6.0
5.5
5.0
4.5
4.0
3.5
3.0
2.5
2.0
1.5
1.0
0.5
0.0
f1 (ppm)

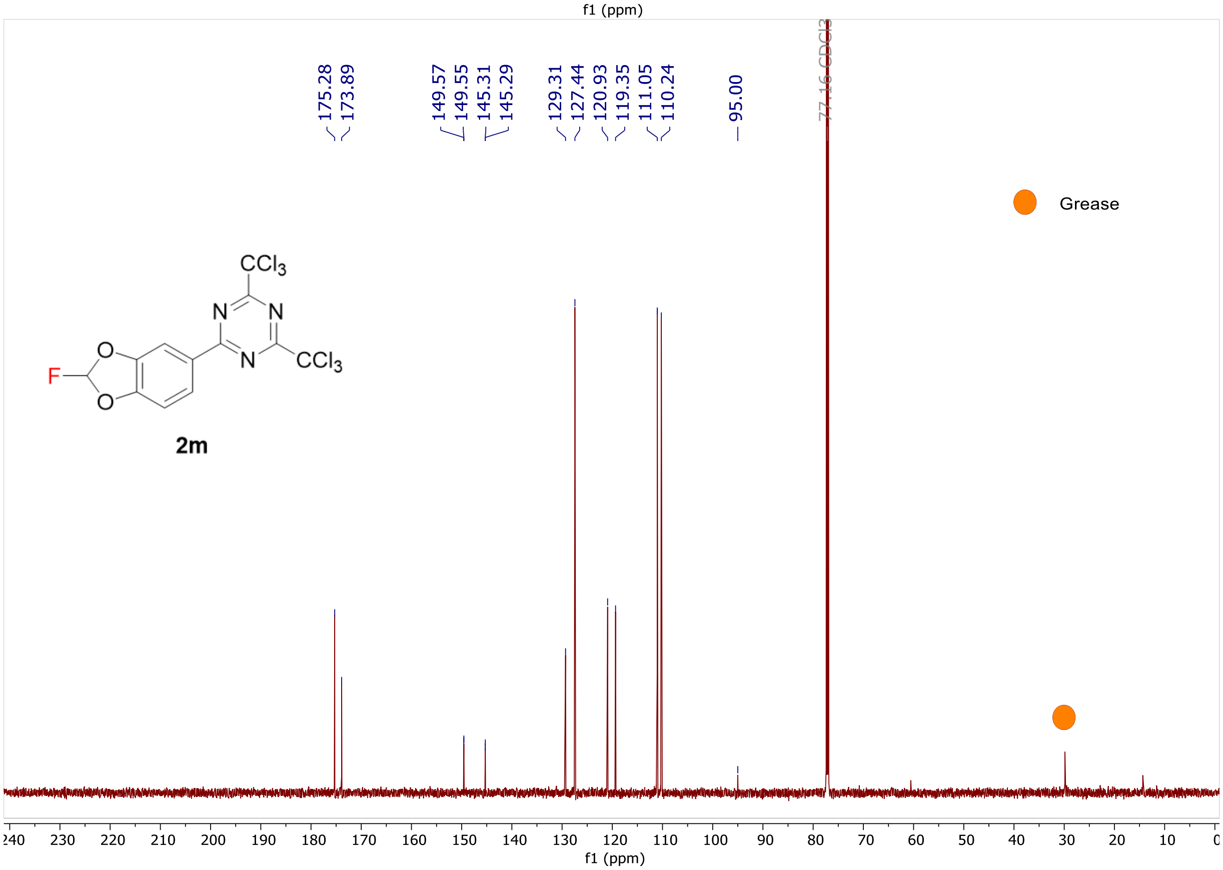
175.28
173.89
149.57
149.55
145.31
145.29
129.31
127.44
120.93
119.35
111.05
110.24
95.00
77.16 CDCl3
Grease
CCl3
N
N
N
CCl3
O
O
F
2m
230
220
210
200
190
180
170
160
150
140
130
120
110
100
90
80
70
60
50
40
30
20
10
f1 (ppm)

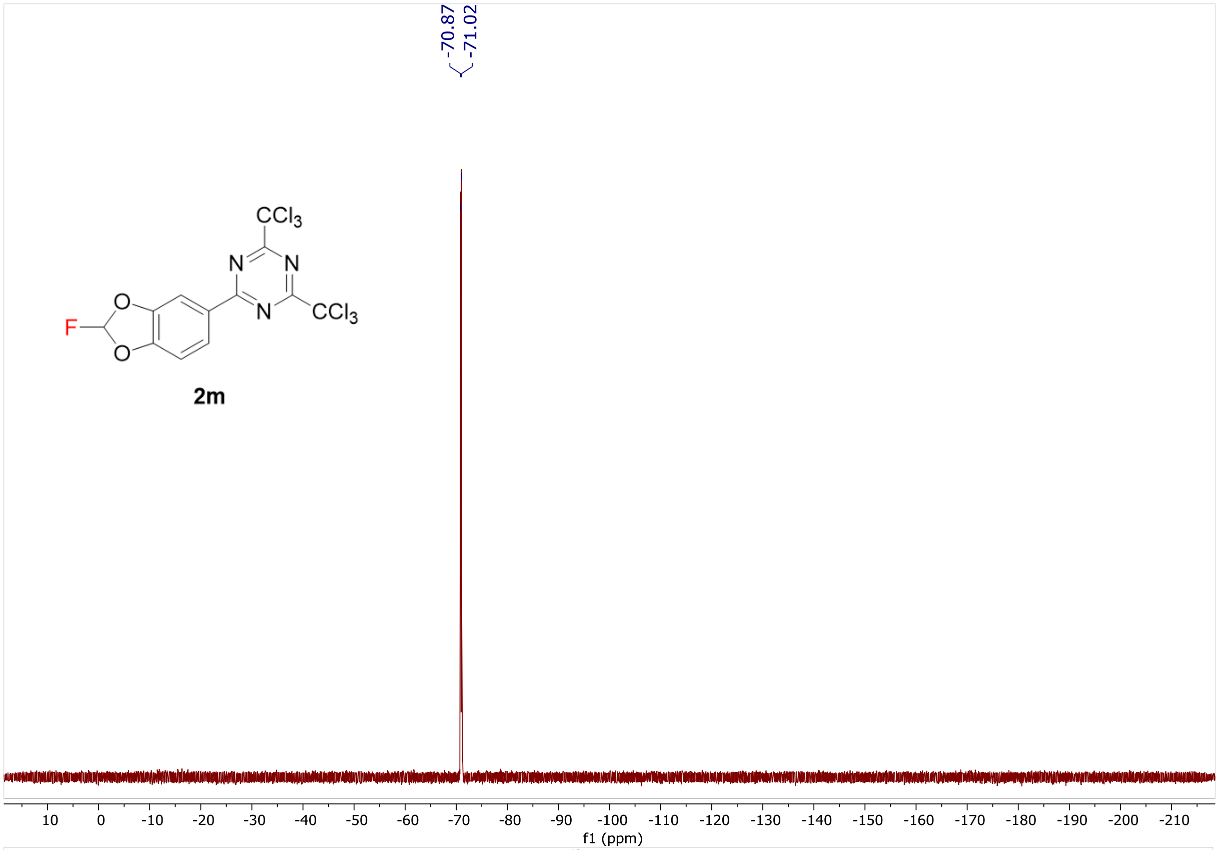
-70.87
-71.02
CCl3
N
N
N
O
O
F
CCl3
2m
10 0 -10 -20 -30 -40 -50 -60 -70 -80 -90 -100 -110 -120 -130 -140 -150 -160 -170 -180 -190 -200 -210
f1 (ppm)

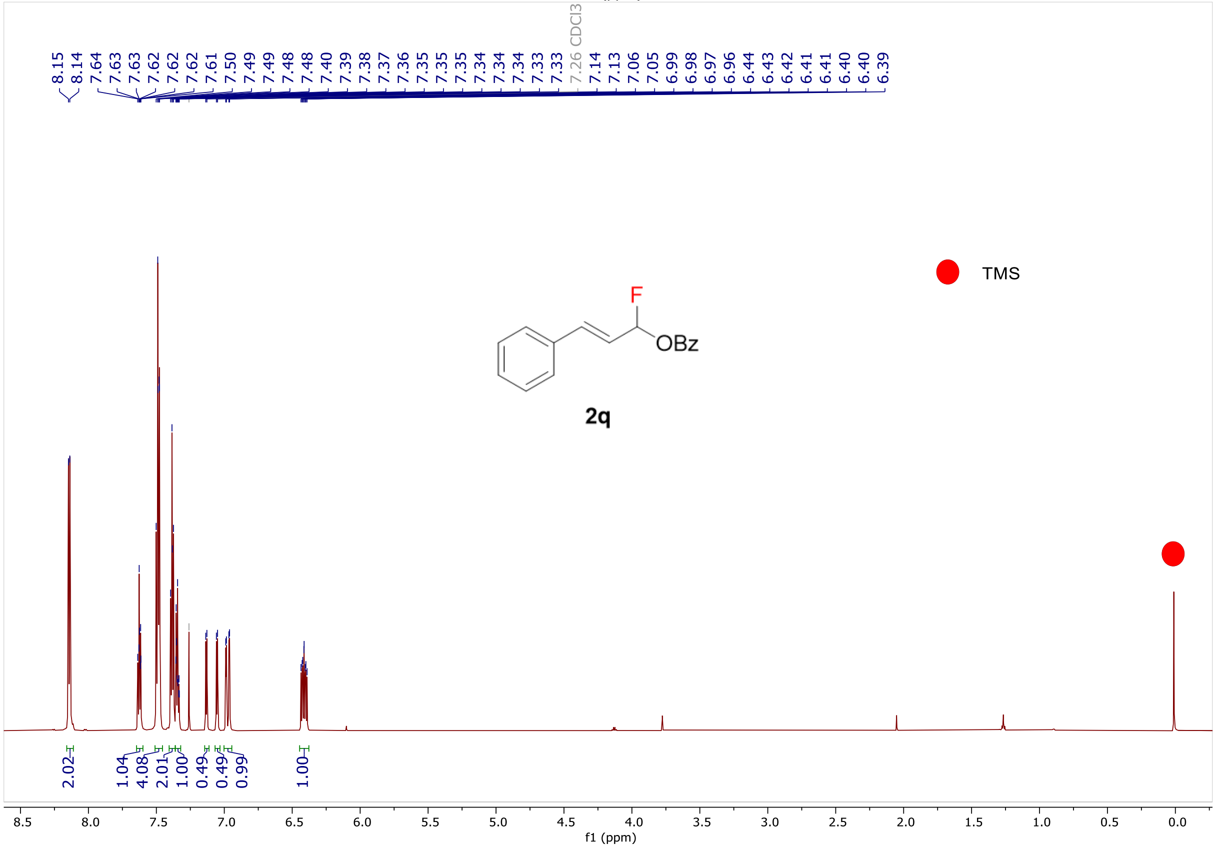
8.15
8.14
7.64
7.63
7.63
7.62
7.62
7.62
7.61
7.50
7.49
7.48
7.48
7.40
7.39
7.38
7.37
7.36
7.35
7.35
7.35
7.34
7.34
7.34
7.33
7.33
7.26 CDCl3
7.14
7.13
7.06
7.05
6.99
6.98
6.97
6.96
6.44
6.43
6.42
6.41
6.40
6.40
6.39
TMS
F
OBz
2q
2.02
1.04
4.08
2.01
1.00
0.49
0.49
0.99
1.00
8.5 8.0 7.5 7.0 6.5 6.0 5.5 5.0 4.5 4.0 3.5 3.0 2.5 2.0 1.5 1.0 0.5 0.0
f1 (ppm)

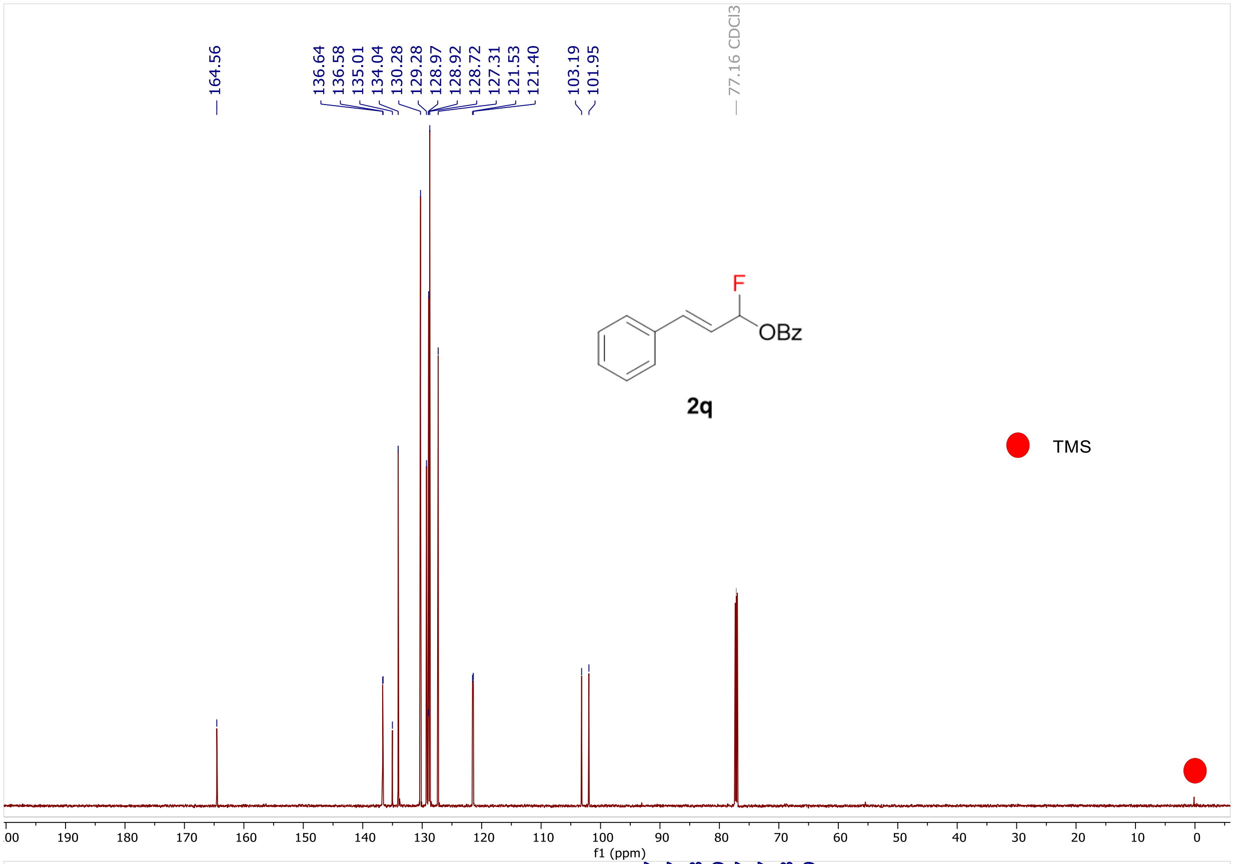
164.56
136.64
136.58
135.01
134.04
130.28
129.28
128.97
128.92
128.72
127.31
121.53
121.40
103.19
101.95
77.16 CDCl3
F
OBz
2q
TMS
f1 (ppm)

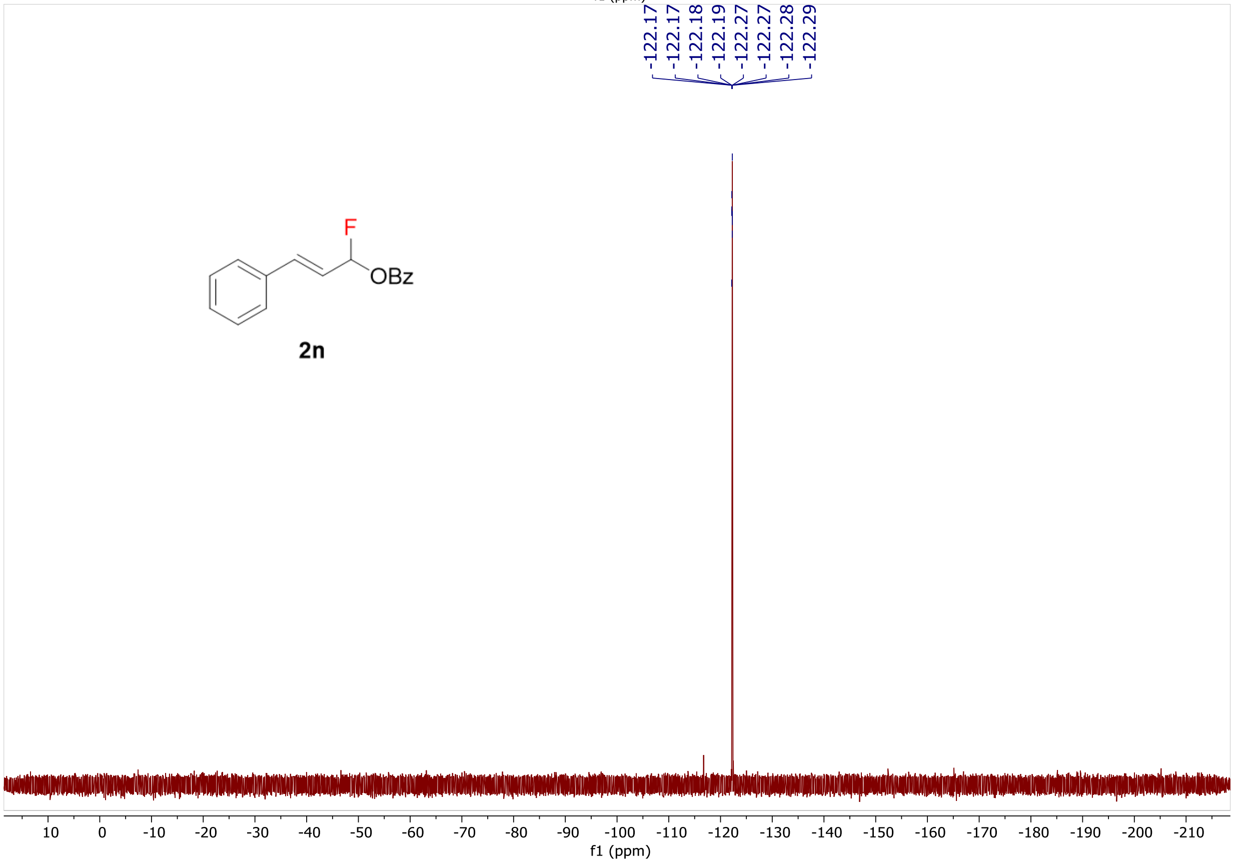
-122.17
-122.17
-122.18
-122.19
-122.27
-122.27
-122.28
-122.29
F
OBz
2n
f1 (ppm)

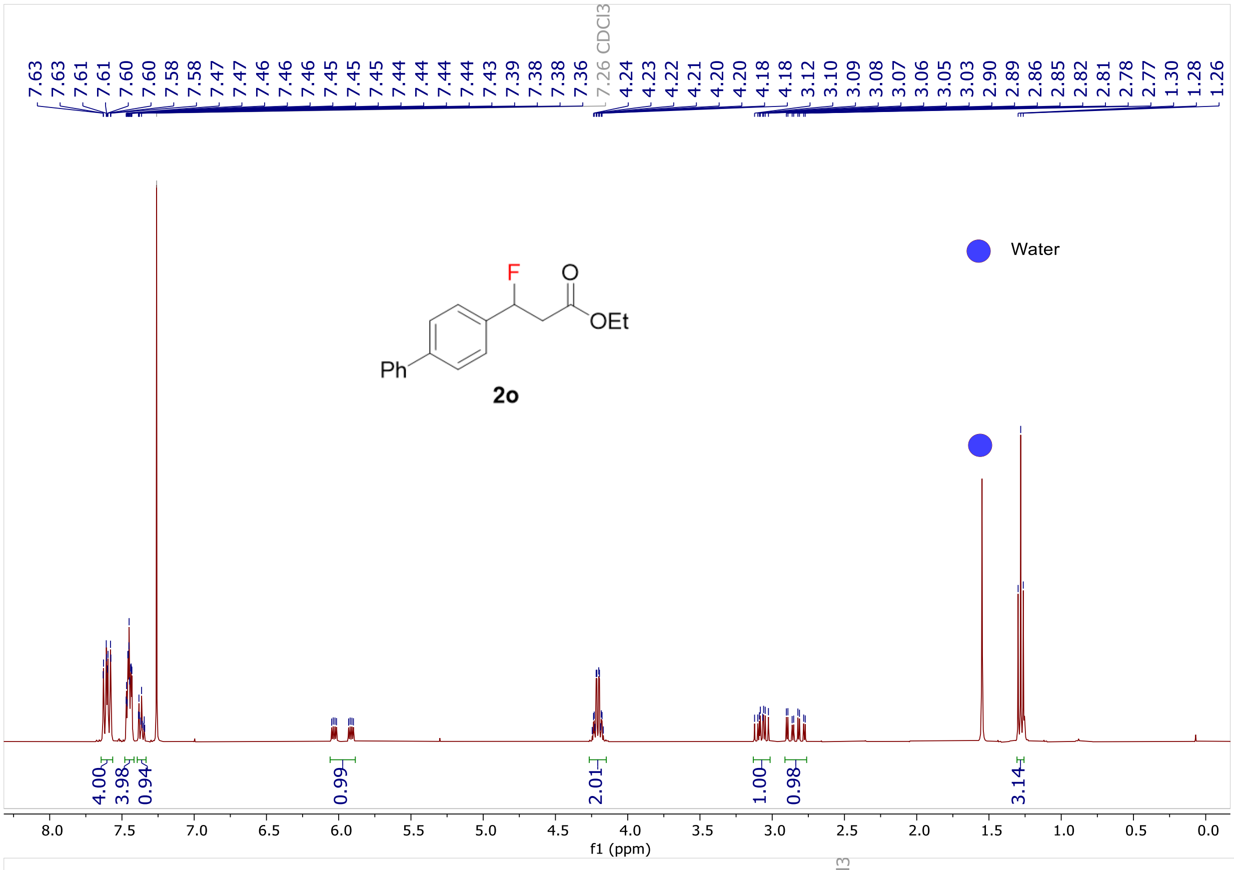
7.63
7.63
7.61
7.61
7.60
7.60
7.58
7.58
7.47
7.47
7.46
7.46
7.46
7.45
7.45
7.45
7.44
7.44
7.44
7.44
7.43
7.39
7.38
7.38
7.36
7.26 CDCl3
4.24
4.23
4.22
4.21
4.20
4.20
4.18
4.18
3.12
3.10
3.09
3.08
3.07
3.06
3.05
3.03
2.90
2.89
2.86
2.85
2.82
2.81
2.78
2.77
1.30
1.28
1.26
Water
F
O
OEt
Ph
2o
4.00
3.98
0.94
0.99
2.01
1.00
0.98
3.14
f1 (ppm)

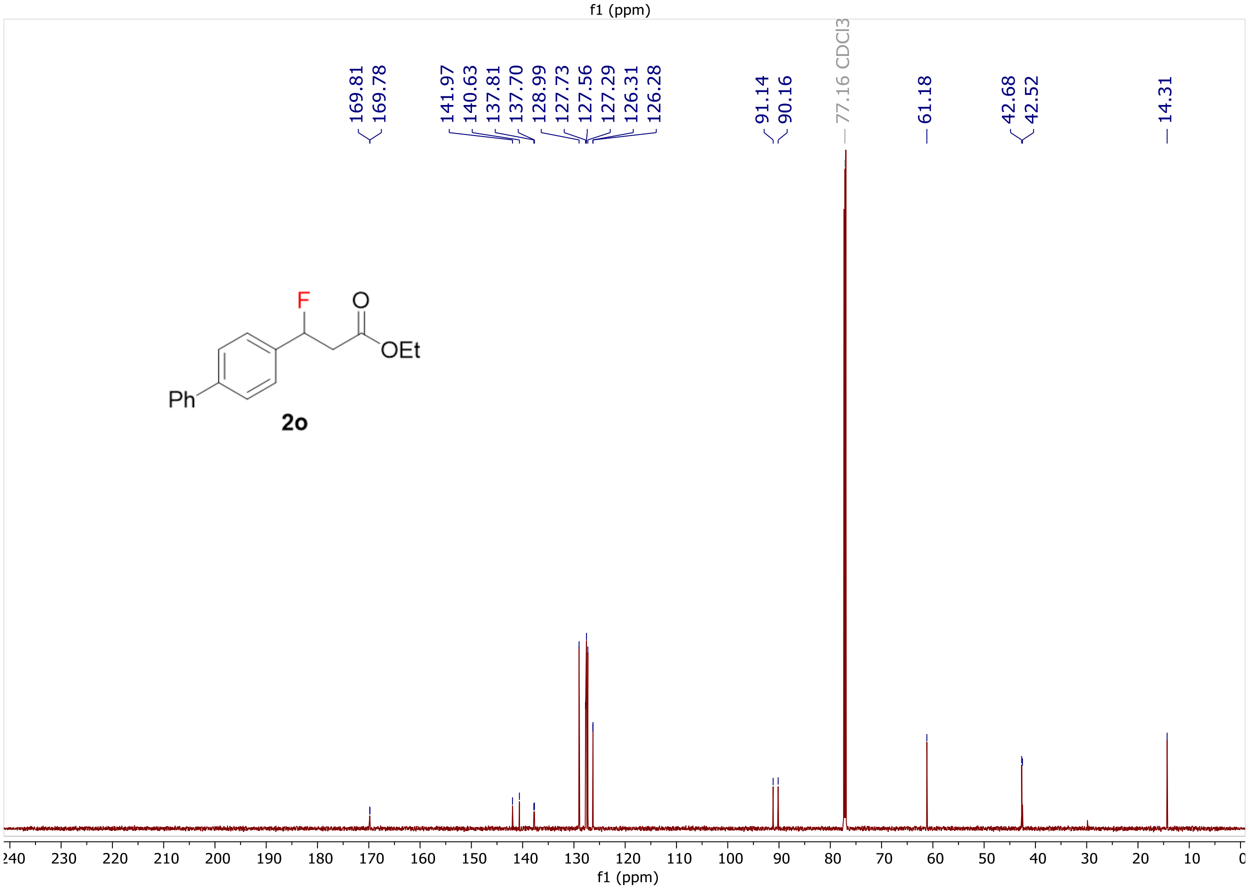
169.81
169.78
141.97
140.63
137.81
137.70
128.99
127.73
127.56
127.29
126.31
126.28
91.14
90.16
77.16 CDCl3
61.18
42.68
42.52
14.31
F
O
OEt
Ph
2o
f1 (ppm)

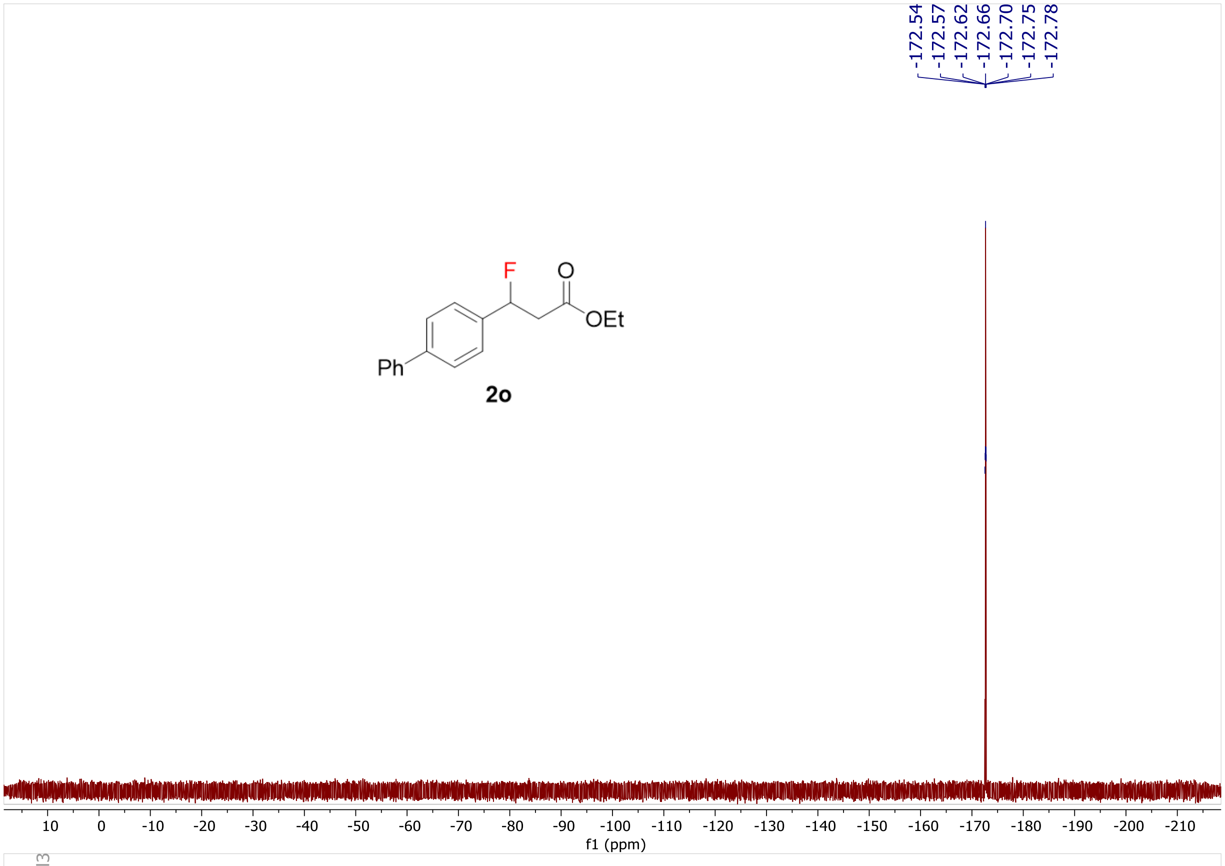

-172.54
-172.57
-172.62
-172.66
-172.70
-172.75
-172.78
F
O
OEt
Ph
2o
10 0 -10 -20 -30 -40 -50 -60 -70 -80 -90 -100 -110 -120 -130 -140 -150 -160 -170 -180 -190 -200 -210
f1 (ppm)

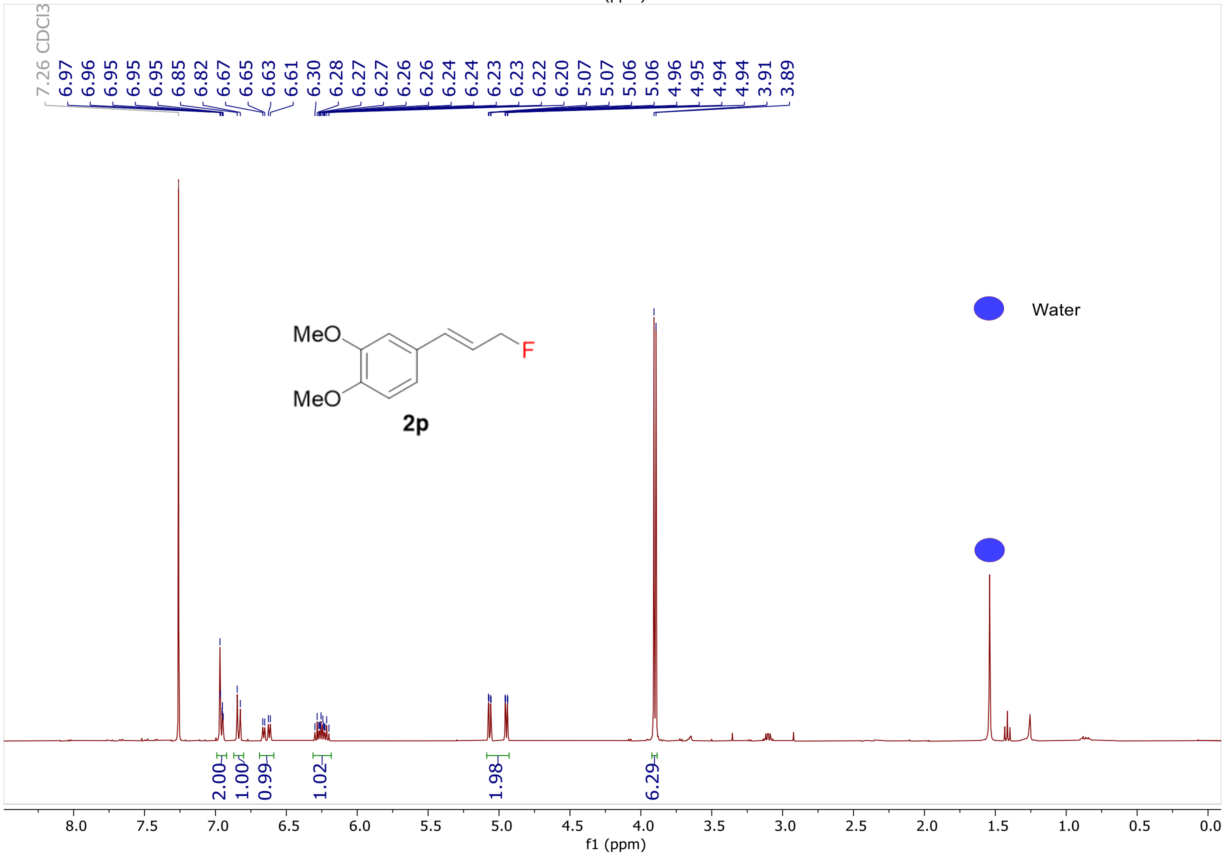

7.26 CDCl3
6.97
6.96
6.95
6.95
6.95
6.85
6.82
6.67
6.65
6.63
6.61
6.30
6.28
6.27
6.27
6.26
6.26
6.24
6.24
6.23
6.23
6.22
6.20
5.07
5.07
5.06
5.06
4.96
4.95
4.94
4.94
3.91
3.89
Water
MeO
MeO
F
2p
2.00
1.00
0.99
1.02
1.98
6.29
8.0 7.5 7.0 6.5 6.0 5.5 5.0 4.5 4.0 3.5 3.0 2.5 2.0 1.5 1.0 0.5 0.0
f1 (ppm)

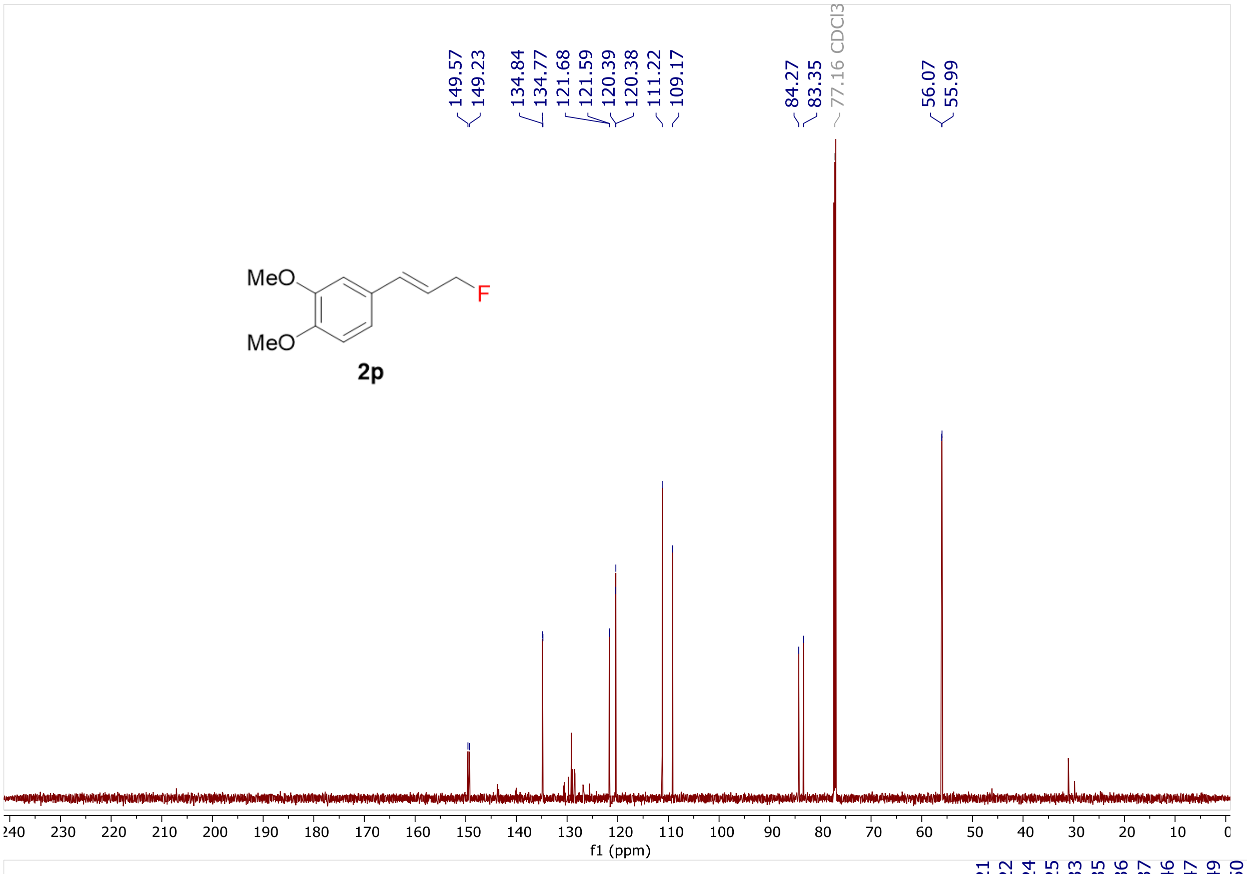
149.57
149.23
134.84
134.77
121.68
121.59
120.39
120.38
111.22
109.17
84.27
83.35
77.16 CDCl3
56.07
55.99
MeO
MeO
F
2p
240 230 220 210 200 190 180 170 160 150 140 130 120 110 100 90 80 70 60 50 40 30 20 10 0
f1 (ppm)

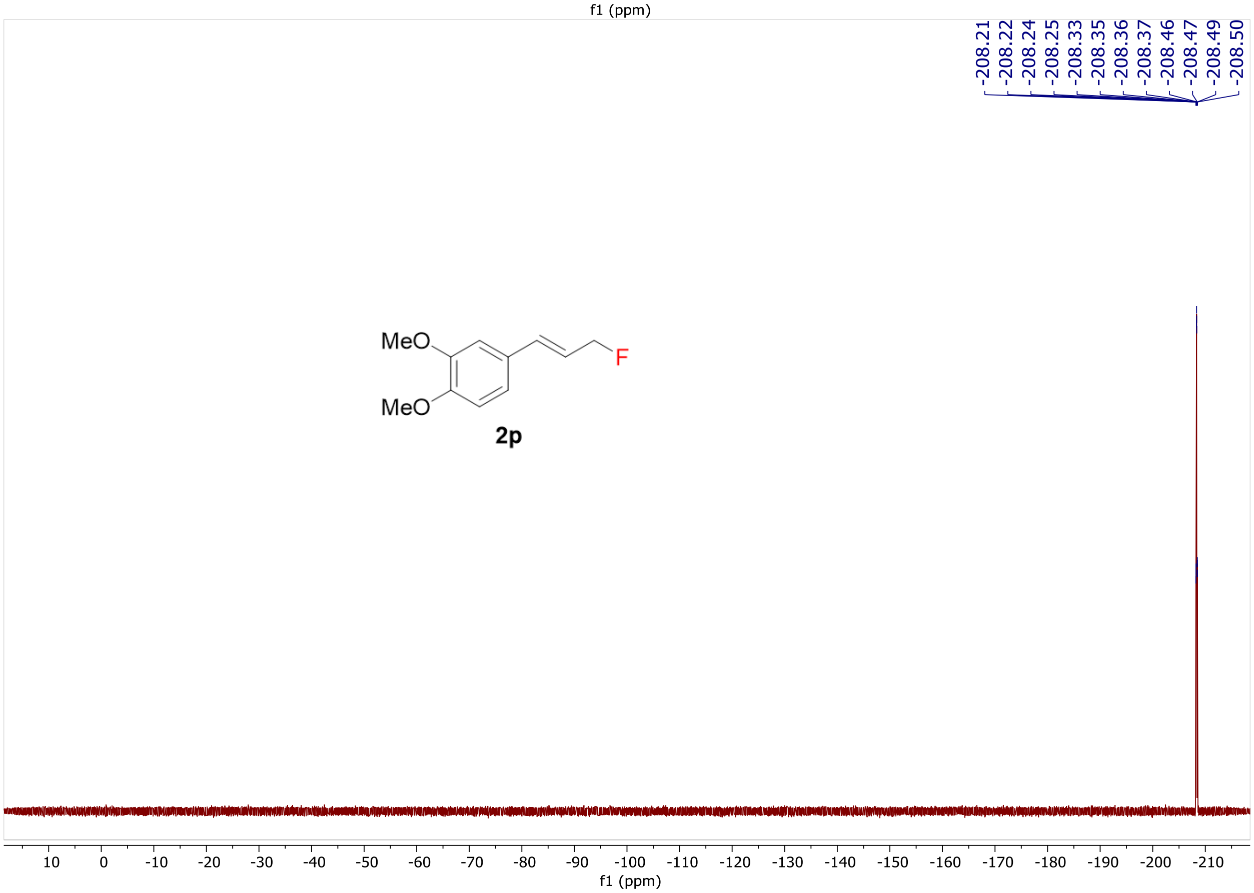
-208.21
-208.22
-208.24
-208.25
-208.33
-208.35
-208.36
-208.37
-208.46
-208.47
-208.49
-208.50
MeO
MeO
F
2p
10 0 -10 -20 -30 -40 -50 -60 -70 -80 -90 -100 -110 -120 -130 -140 -150 -160 -170 -180 -190 -200 -210
f1 (ppm)

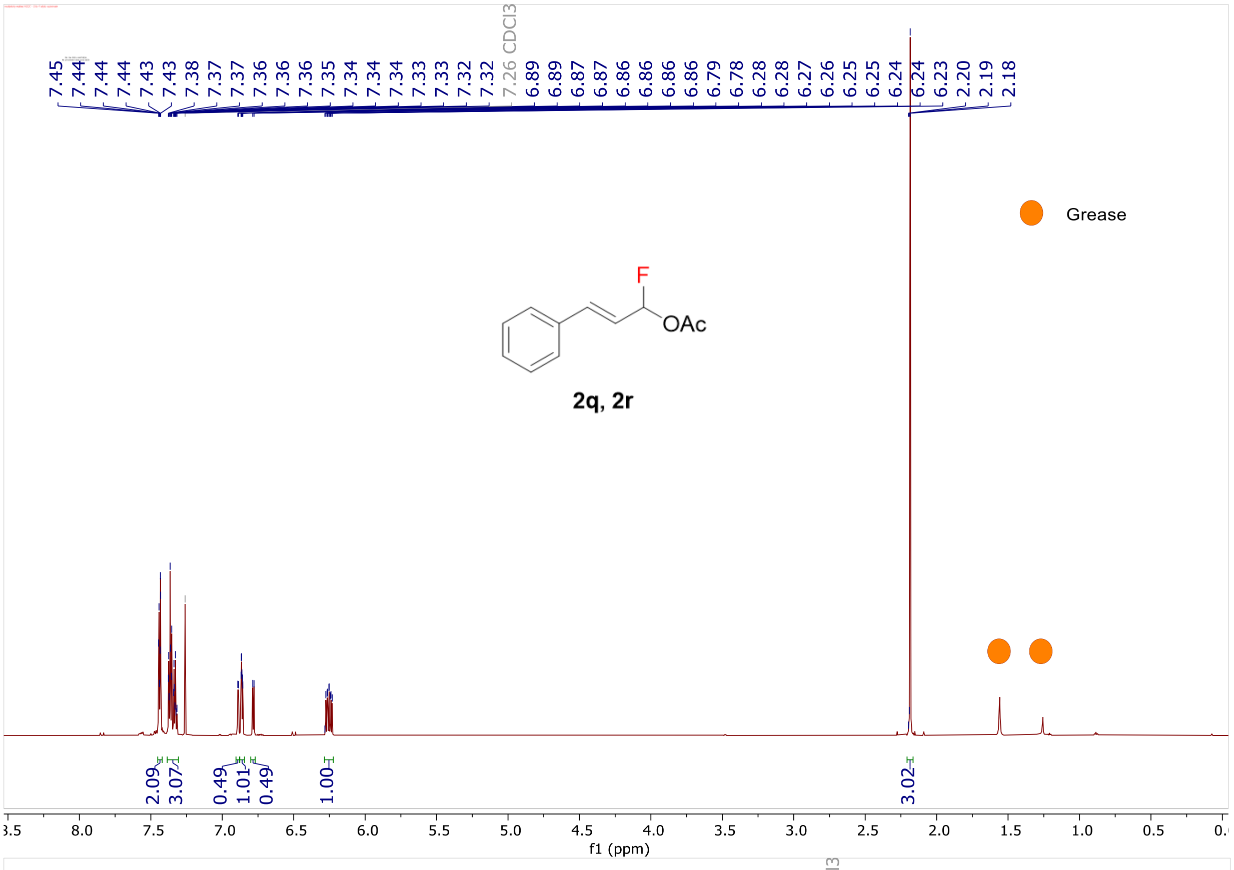
7.45
7.44
7.44
7.44
7.43
7.43
7.38
7.37
7.37
7.36
7.36
7.36
7.35
7.34
7.34
7.34
7.33
7.33
7.32
7.32
7.26 CDCl3
6.89
6.89
6.87
6.87
6.86
6.86
6.86
6.86
6.79
6.78
6.28
6.28
6.27
6.26
6.25
6.25
6.24
6.24
6.23
2.20
2.19
2.18
Grease
F
OAc
2q, 2r
2.09
3.07
0.49
1.01
0.49
1.00
3.02
f1 (ppm)

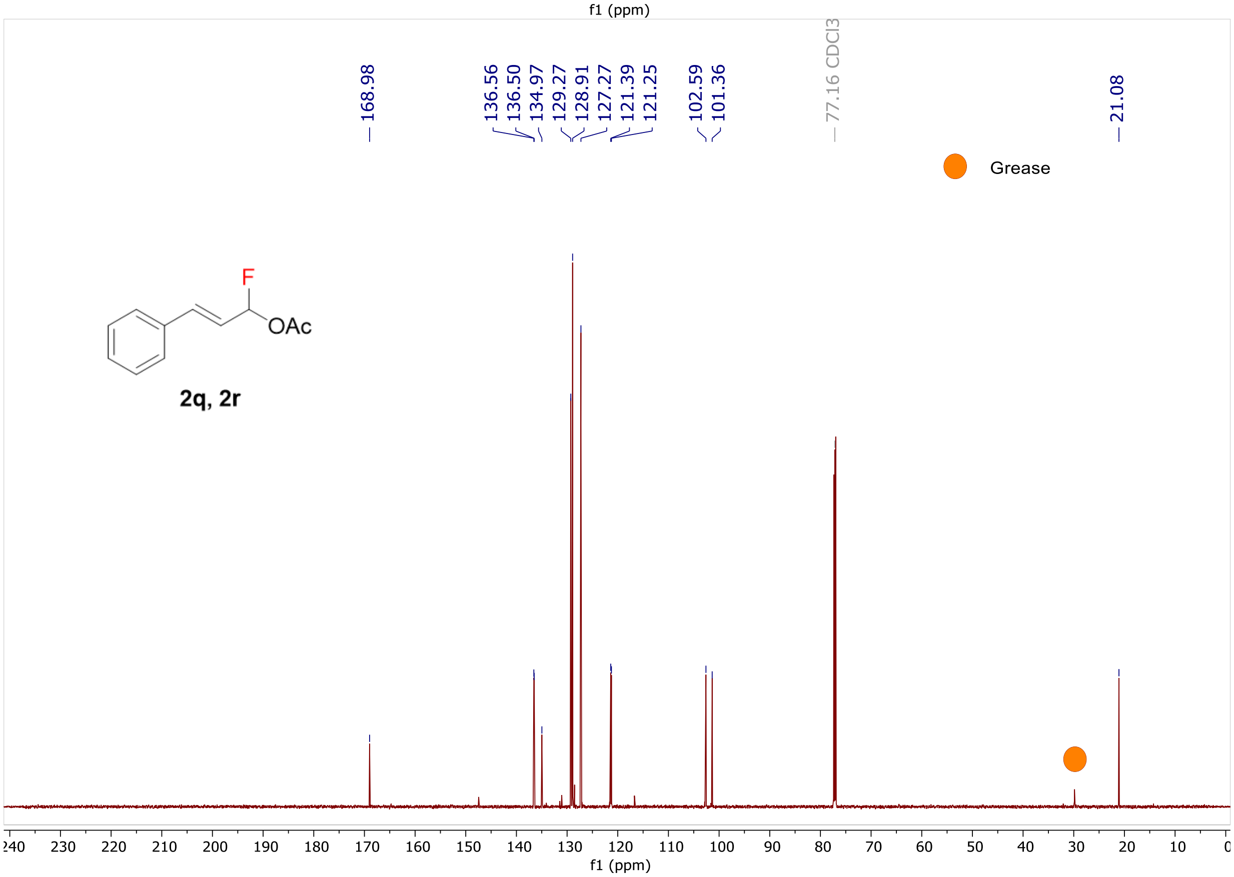
168.98
136.56
136.50
134.97
129.27
128.91
127.27
121.39
121.25
102.59
101.36
77.16 CDCl3
21.08
Grease
F
OAc
2q, 2r
f1 (ppm)

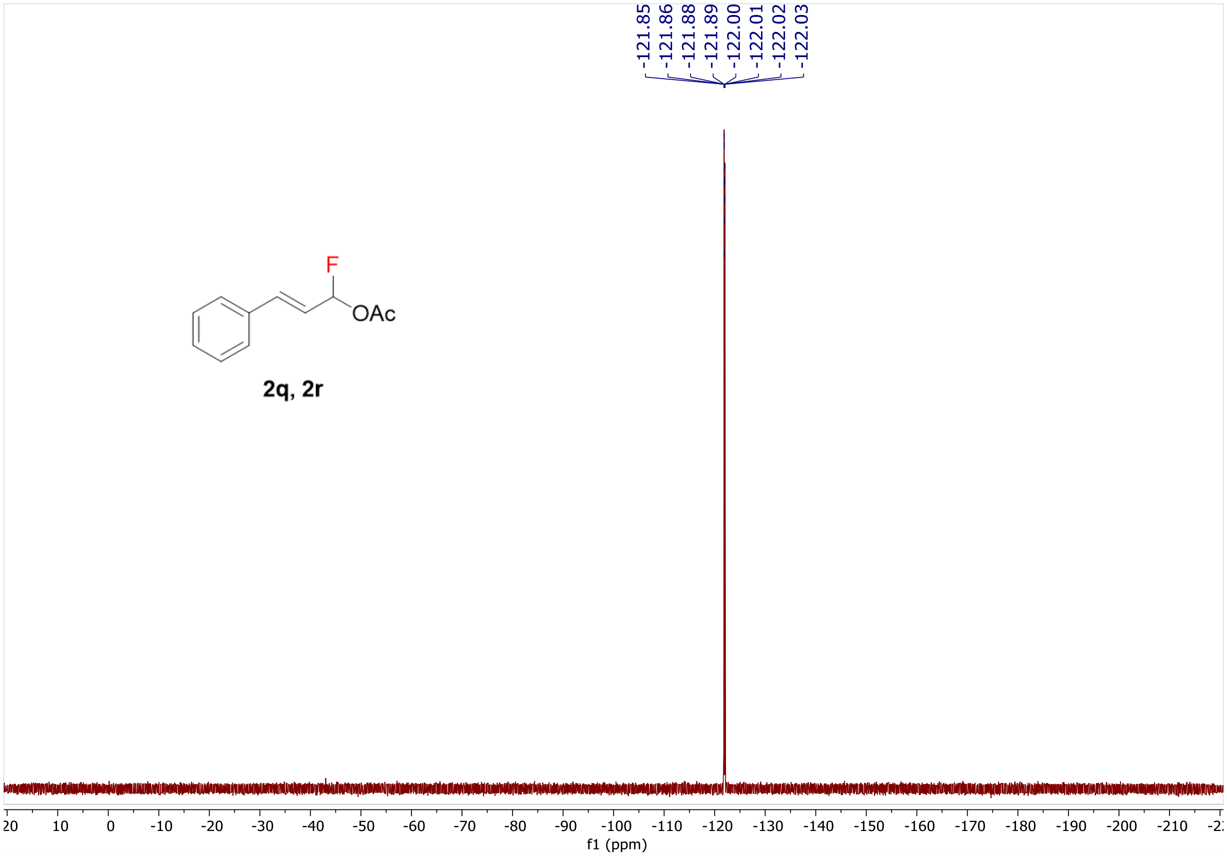
-121.85
-121.86
-121.88
-121.89
-122.00
-122.01
-122.02
-122.03
F
OAc
2q, 2r
f1 (ppm)

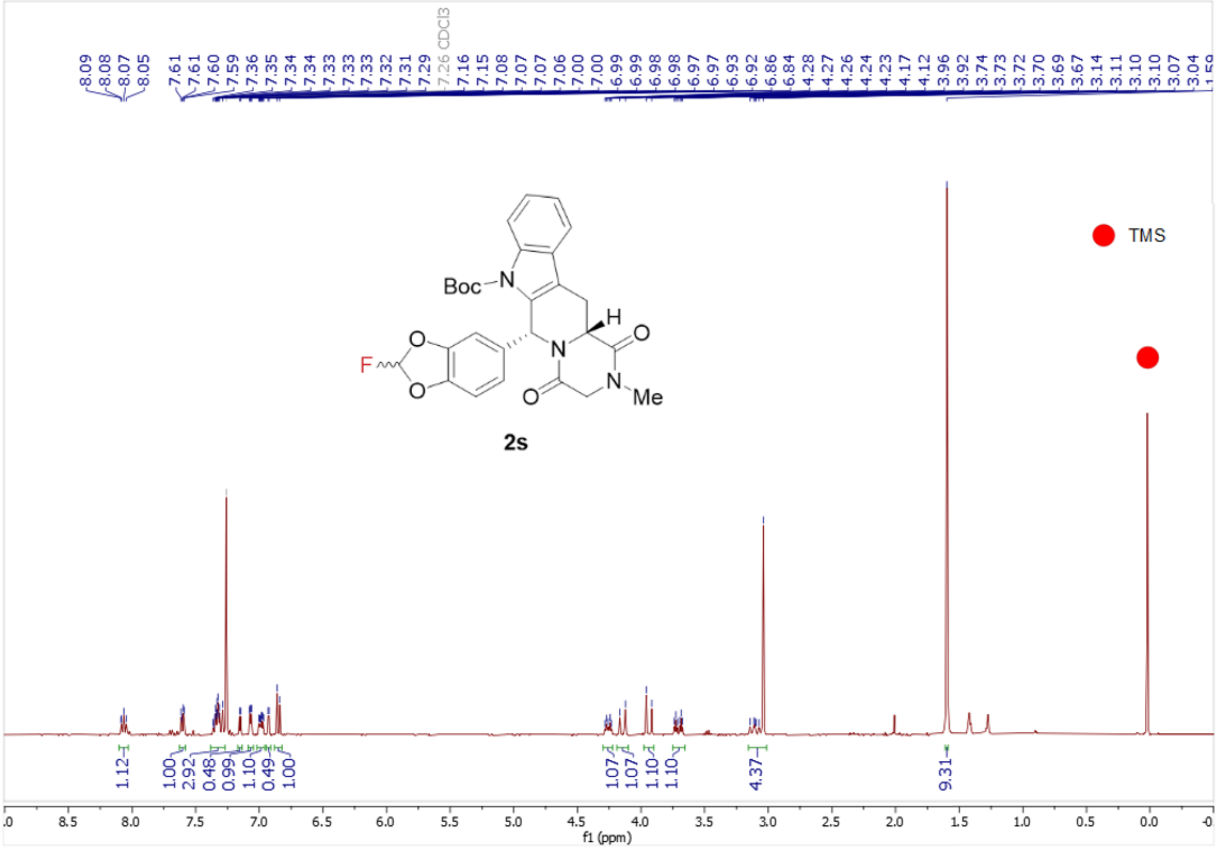
CDCl3
TMS
Boc
N
H
O
F
N
O
Me
2s
f1 (ppm)

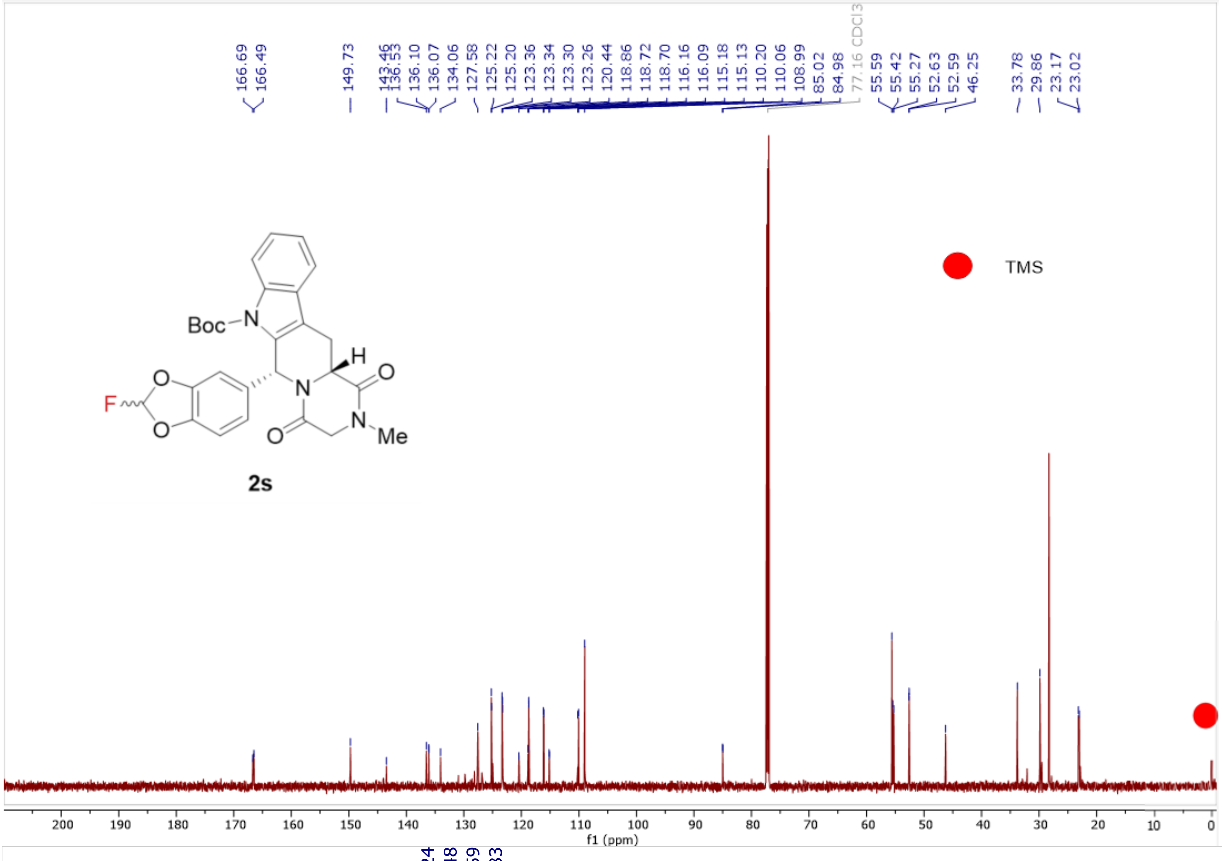
166.69
166.49
149.73
143.46
136.53
136.10
136.07
134.06
127.58
125.22
125.20
123.36
123.34
123.30
123.26
120.44
118.86
118.72
118.70
116.16
116.09
115.18
115.13
110.20
110.06
108.99
85.02
84.98
77.16 CDCl3
55.59
55.42
55.27
52.63
52.59
46.25
33.78
29.86
23.17
23.02
TMS
Boc
N
H
O
F
Me
2s
f1 (ppm)

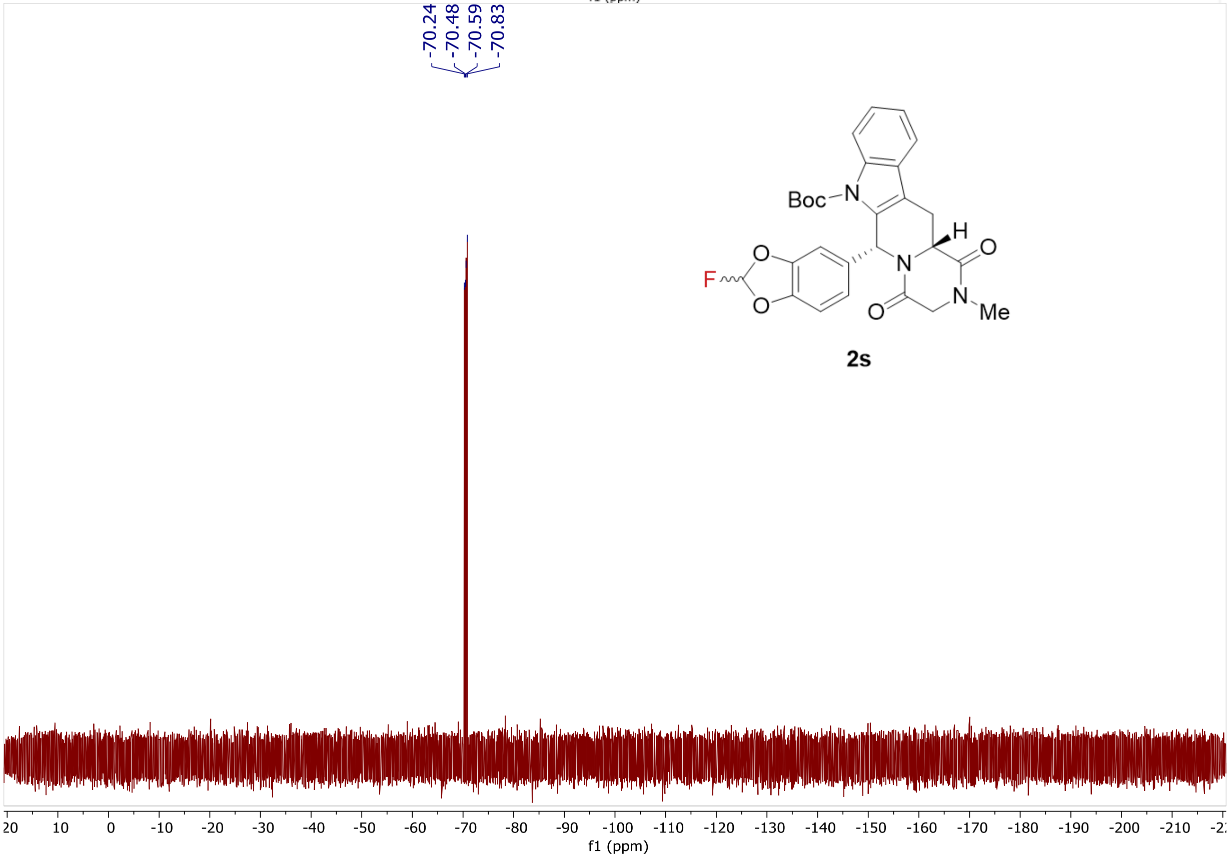
-70.24
-70.48
-70.59
-70.83
Boc
N
H
O
F
Me
2s
f1 (ppm)

References

(1) K. Bower, J.; D. Cypcar, A.; Henriquez, B.; Chantal E. Stieber, S.; Zhang, S. C(Sp^3)–H Fluorination with a Copper(II)/(III) Redox Couple. *J. Am. Chem. Soc.* **2020**, *142*, 8514–8521.

(2) J. Donoghue, P.; Tehranchi, J.; J. Cramer, C.; Sarangi, R.; I. Solomon, E.; B. Tolman, W. Rapid C–H Bond Activation by a Monocopper(III)–Hydroxide Complex. *J. Am. Chem. Soc.* **2011**, *133*, 17602–17605.

(3) LI ZHEN [CN]; TANG FENG [CN]; FU YAYUAN [CN]; LIU LIFENG [CN]; ZHAO CHUNYAN [CN]; TANG RENHONG [CN]; REN JINSHENG [CN]. Camptothecin Derivative, and Pharmaceutical Composition and Use Thereof. HAINAN SIMCERE ZAIMING PHARMACEUTICAL CO LTD 2024.

(4) Prashanth, M. K.; Revanasiddappa, H. D.; Lokanatha Rai, K. M.; Veeresh, B. Synthesis, Characterization, Antidepressant and Antioxidant Activity of Novel Piperamides Bearing Piperidine and Piperazine Analogues. *Bioorganic Med. Chem. Lett.* **2012**, *22*, 7065–7070.

(5) AZIZ-UR-REHMAN1,*, A. S.; ABBASI1, M. A.; RASOOL1, S.; AKHTAR2, M. NADEEMLODHI3, M. A.; NAFEESA1, K.; KHAN, and A. Synthesis, Characterization and Urease Inhibiting Derivatives of 5-(3,4-Methylenedioxyphenyl)-1,3,4-Oxadiazol-2-Thiol. *Asian J. Chem.* **2014**, *26*, 4605–4609.

(6) Che, Z. P.; Yang, J. M.; Shan, X. J.; Tian, Y. E.; Liu, S. M.; Lin, X. M.; Jiang, J.; Hu, M.; Chen, G. Q. Synthesis and Insecticidal Activity of Sulfonate Derivatives of Sesamol against *Mythimna Separata in Vivo*. *J. Asian Nat. Prod. Res.* **2020**, *22*, 678–688.

(7) Kinuta, H.; Tobisu, M.; Chatani, N. Rhodium-Catalyzed Borylation of Aryl 2-Pyridyl Ethers through Cleavage of the Carbon-Oxygen Bond: Borylative Removal of the Directing Group. *J. Am. Chem. Soc.* **2015**, *137*, 1593–1600.

(8) Yang, J.; Xu, F.; Shi, S.; Nie, J. Influence of Structure of Benzodioxole Derivatives on Photoinitiation Efficiency of Benzophenone. *Photochem. Photobiol. Sci.* **2012**, *11*, 1377–1382.

(9) Radomkit, S.; Sarnpitak, P.; Tummatorn, J.; Batsomboon, P.; Ruchirawat, S.; Ploypradith, P. Pt(IV)-Catalyzed Generation and [4+2]-Cycloaddition Reactions of o-Quinone Methides. *Tetrahedron* **2011**, *67*, 3904–3914.

(10) Deng, W.; Hu, Y.; Hu, J.; Li, X.; Li, Y.; Huang, Y. Electrochemically Induced Markovnikov-Type Selective Hydro/Deuterophosphonylation of Electron-Rich Alkenes. *Chem. Commun.* **2022**, *58*, 12094–12097.

(11) Xu, Y.; Chen, L.; Yang, Y. W.; Zhang, Z.; Yang, W. Vinylethylene Carbonates as *α,β*-Unsaturated Aldehyde Surrogates for Regioselective [3 + 3] Cycloaddition. *Org. Lett.* **2019**, *21*, 6674–6678.

(12) Naef, R.; Tenor, H.; Koch, G.; Ludin, C. Novel Dual Mode of Action Soluble Guanylate Cyclase Activtors And Phosphodiesterase Iinhibitors And Uses Thereof, 2021.

(13) Bower, J. K.; Cypcar, A. D.; Henriquez, B.; Stieber, S. C. E.; Zhang, S. C(Sp^3)-H Fluorination with a Copper(II)/(III) Redox Couple. *J. Am. Chem. Soc.* **2020**, *142*, 8514–8521.

(14) Bloom, S.; McCann, M.; Lectka, T. Photocatalyzed Benzylic Fluorination: Shedding “Light” on the Involvement of Electron Transfer. *Org. Lett.* **2014**, *16*, 6338–6341.

(15) Moriyama, K.; Nakamura, Y.; Togo, H. Oxidative Debenzylation of N-Benzyl Amides and O-

Benzyl Ethers Using Alkali Metal Bromide. *Org. Lett.* **2014**, *16*, 3812–3815.

(16) Kokubo, T.; Takadama, H. How Useful Is SBF in Predicting in Vivo Bone Bioactivity? *Biomaterials* **2006**, *27*, 2907–2915.

(17) Yilmaz, B.; Pazarceviren, A. E.; Tezcaner, A.; Evis, Z. Historical Development of Simulated Body Fluids Used in Biomedical Applications: A Review. *Microchem. J.* **2020**, *155*, 104713.

(18) Neese, F. Software Update: The ORCA Program System, Version 4.0. *Wiley Interdiscip. Rev. Comput. Mol. Sci.* **2018**, *8*, e1327.

(19) Frisch, M. J.; Trucks, G. W.; Schlegel, H. B.; Scuseria, G. E.; Robb, M. a.; Cheeseman, J. R.; Scalmani, G.; Barone, V.; Petersson, G. a.; Nakatsuji, H.; Li, X.; Caricato, M.; Fox, D. J.; et al. G16_C01. 2016, p Gaussian 16, Revision C.01, Gaussian, Inc., Wallin.

(20) Mandal, M.; Elwell, C. E.; Bouchey, C. J.; Zerk, T. J.; Tolman, W. B.; Cramer, C. J. Mechanisms for Hydrogen-Atom Abstraction by Mononuclear Copper(III) Cores: Hydrogen-Atom Transfer or Concerted Proton-Coupled Electron Transfer? *J. Am. Chem. Soc.* **2019**, *141*, 17236–17244.

(21) Bím, D.; Maldonado-Domínguez, M.; Rulísek, L.; Srnec, M. Beyond the Classical Thermodynamic Contributions to Hydrogen Atom Abstraction Reactivity. *Proc. Natl. Acad. Sci. U. S. A.* **2018**, *115*, E10287–E10294.
